# A New Supervised Over-Sampling Algorithm with Application to Protein-Nucleotide Binding Residue Prediction

**DOI:** 10.1371/journal.pone.0107676

**Published:** 2014-09-17

**Authors:** Jun Hu, Xue He, Dong-Jun Yu, Xi-Bei Yang, Jing-Yu Yang, Hong-Bin Shen

**Affiliations:** 1 School of Computer Science and Engineering, Nanjing University of Science and Technology, Nanjing, Jiangsu, China; 2 Institute of Image Processing and Pattern Recognition, Shanghai Jiao Tong University, Shanghai, China; 3 Changshu Institute, Nanjing University of Science and Technology, Changshu, Jiangsu, China; 4 School of Computer Science and Engineering, Jiangsu University of Science and Technology, Zhenjiang, Jiangsu, China; University of Michigan, United States of America

## Abstract

Protein-nucleotide interactions are ubiquitous in a wide variety of biological processes. Accurately identifying interaction residues solely from protein sequences is useful for both protein function annotation and drug design, especially in the post-genomic era, as large volumes of protein data have not been functionally annotated. Protein-nucleotide binding residue prediction is a typical imbalanced learning problem, where binding residues are extremely fewer in number than non-binding residues. Alleviating the severity of class imbalance has been demonstrated to be a promising means of improving the prediction performance of a machine-learning-based predictor for class imbalance problems. However, little attention has been paid to the negative impact of class imbalance on protein-nucleotide binding residue prediction. In this study, we propose a new supervised over-sampling algorithm that synthesizes additional minority class samples to address class imbalance. The experimental results from protein-nucleotide interaction datasets demonstrate that the proposed supervised over-sampling algorithm can relieve the severity of class imbalance and help to improve prediction performance. Based on the proposed over-sampling algorithm, a predictor, called TargetSOS, is implemented for protein-nucleotide binding residue prediction. Cross-validation tests and independent validation tests demonstrate the effectiveness of TargetSOS. The web-server and datasets used in this study are freely available at http://www.csbio.sjtu.edu.cn/bioinf/TargetSOS/.

## Introduction

Protein-ligand interactions are ubiquitous in virtually all biological processes [1]–[3], and the prediction of protein-ligand interactions using automated computational methods has been an area of intense research in bioinformatics fields [4]–[15]. As important ligand types, nucleotides (e.g., ATP, ADP, AMP, GDP, and GTP) play critical roles in various metabolic processes, such as providing chemical energy, signaling, and replication and transcription of DNA [10]–[15]. The residues in a protein to which nucleotides bind are called protein-nucleotide binding residues. By interacting with the binding residues in a protein, nucleotides can carry out their specific biological functions. Furthermore, protein-nucleotide (e.g., protein-ATP) binding residues are considered valuable targets of therapeutic drugs [12]. Hence, accurate identification of nucleotide-binding residues in protein sequences is of significant importance for protein function analysis and drug design [16], especially in the post-genomic era, as large volumes of protein data have not been functionally annotated.

Much effort has been made to identify and characterize nucleotide-binding residues from protein sequences. In the early stages, motif-based methods [17]–[21] dominated this field. For most motif-based methods, conserved motifs in known nucleotide-binding protein sequences or structures are first identified; then, the identified motifs are further utilized to uncover potential binding residues in those un-annotated proteins. Although considerable progress has been achieved in motif-based methods, challenges remain. As Chen et al. [14] reported, motif-based methods often characterize the protein-nucleotide interaction motifs within a relatively narrow range, usually only for a selected interaction mode for a single nucleotide type; in addition, some motif-based methods require tertiary protein structure as the input, which substantially limits their utility, as it is very common in many realistic application scenarios for a given protein target to only have sequence information and no corresponding tertiary structure information [22], [23].

The above-mentioned challenges have motivated researchers in this field to develop machine-learning-based methods for predicting protein-ligand binding residues solely from protein sequences [4]–[6], [13], [14], [22], [24]–[26]. In pioneering work, Chauhan et al. [13] designed a predictor, called ATPint, specifically for predicting protein-ATP binding residues. This group also designed a GTP-specific predictor for protein-GTP binding residue prediction [27], and their earlier studies demonstrated the feasibility of predicting protein-nucleotide binding residues solely from protein sequence information [13], [27]. Later, researchers tended to design predictors that covered a wide range of nucleotide types. For example, Firoz et al. [15] implemented a method of performing binding residue predictions for six nucleotide types, i.e., AMP, GMP, ADP, GDP, ATP and GTP. Recently, Chen et al. [14] presented a predictor, called NsitePred, that could also be used to perform binding residue predictions for multiple nucleotides based on much larger training datasets. All in all, great success has been achieved in this field.

Machine-learning-based protein-nucleotide binding residue prediction is, in fact, a typical imbalanced learning problem because the number of negative samples (i.e., non-binding residues) is significantly larger than that of positive samples (i.e., binding residues). Previous studies in the machine-learning field have shown that direct application of traditional machine-learning algorithms tends to result in a bias toward the majority class [28]. Unfortunately, most of the existing machine-learning-based predictors, including ATPint [13], ATPsite [24], and NsitePred [14], have not carefully considered this serious class imbalance phenomenon.

Considerable effort has been made to develop effective solutions for imbalanced learning [28]. Roughly speaking, the existing solutions for imbalanced learning can be grouped into three categories: sample rescaling-based methods [29], [30], learning-based methods (e.g., cost-sensitive learning [31], [32], active learning [33], [34], kernel learning [35], [36]), and hybrid methods, which combine both the sampling rescaling and learning methods [37], [38].

Among the above-mentioned solutions, the sample rescaling strategy (e.g., over-sampling [39] and under-sampling [40]) is the basic technique, and it attempts to balance the sizes of different classes by changing the numbers and distributions within them; this strategy has been demonstrated to be effective for imbalanced learning problems [29], [30]. For example, we recently investigated class imbalance in the protein-nucleotide binding prediction problem and found that prediction performance could be improved by balancing the number of samples in different classes via an under-sampling technique [22], [25], [26].

In this study, we seek to overcome the problem of class imbalance via an over-sampling technique. In contrast to the under-sampling technique, which reduces the size of the majority class, an over-sampling technique attempts to balance the sizes of different classes by generating additional samples for the minority class. To date, many over-sampling techniques have emerged, including random over-sampling (ROS), the synthetic minority over-sampling technique (SMOTE) [39], and adaptive synthetic sampling (ADASYN) [41]. Motivated by these existing over-sampling techniques, in this study, we propose a new supervised over-sampling (SOS) algorithm that synthesizes new additional samples for minority classes using a supervised process to guarantee the validity of the synthesized samples. Additionally, a new predictor, called TargetSOS, is developed based on the proposed SOS for performing protein-nucleotide binding residue prediction. The experimental results from two benchmark datasets demonstrate the effectiveness of TargetSOS. TargetSOS and the datasets used in this study are freely available at http://www.csbio.sjtu.edu.cn/bioinf/TargetSOS/.

## Materials and Methods

### Benchmark Datasets

Two benchmark datasets were chosen to evaluate the efficacy of the proposed SOS algorithm and of the implemented predictor, TargetSOS. The first dataset [13], ATP168, consists of 168 non-redundant, ATP-interacting protein sequences, of which the maximal pairwise sequence identity is less than 40%. In total, ATP168 includes 3104 and 59226 residues for ATP binding and ATP non-binding, respectively. The second dataset [14], NUC5, is a multiple nucleotide-interacting dataset that consists of five training sub-datasets, each for a specific type of nucleotide; more specifically, NUC5 consists of 227, 321, 140, 56, and 105 protein sequences that interact with five types of nucleotides, i.e., ATP, ADP, AMP, GTP, and GDP, respectively, and the maximal pairwise identity of the sequences of each of the five sub-datasets is less than 40%. In addition, for each nucleotide type, Chen et al. [14] constructed a corresponding, independent validation dataset to evaluate the generalization capability of a prediction model. For each independent validation dataset, the maximal pairwise sequence identity is culled to 40%. Furthermore, any sequence in the independent validation dataset shares less than 40% identity to sequences in the corresponding training sub-dataset. Table 1 summarizes the detailed compositions of the two benchmark datasets. All data listed in Table 1 can be found in Supporting Information S1. Further details regarding the construction of the datasets can be found in [13] and [14].

**Table 1:** Compositions of the two benchmark datasets.

Dataset	Ligand Type	Cross-Validation Dataset (Training Dataset)			Independent Validation Dataset			Total No. of Sequences
		No. of Sequences	(numP, numN)*	Ratio^△^	No. of Sequences	(numP, numN)*	Ratio^△^	
ATP168 [13]	ATP	168	(3104, 59226)	19	–	–	–	168
	ATP	227	(3393, 80409)	24	17	(248, 6974)	28	244
	ADP	321	(4688, 121158)	26	26	(405, 10553)	26	347
NUC5 [14]	AMP	140	(1756, 44009)	25	20	(263, 6057)	23	160
	GTP	56	(875, 21401)	24	7	(134, 2678)	20	63
	GDP	105	(1577, 36561)	23	7	(94, 2420)	26	112

* Figures numP, numN in 2-tuple (numP, numN) represent the number of positive (binding residues) and negative (non-binding residues) samples, respectively; ^△^ Ratio = numN/numP.

### Feature Representation and Classifier

The main purpose of this study is to demonstrate the feasibility of the proposed SOS algorithm and its effectiveness in protein-nucleotide binding residue prediction. To fulfill the aforementioned purpose, only the most commonly used feature representation methods and classifiers in the field of protein-nucleotide binding residue prediction are used. More specifically, the position-specific scoring matrix (PSSM) and predicted protein secondary structure (PSS), both of which have been demonstrated to be especially useful for protein-nucleotide binding residue prediction [13], [14], [25], [26], are taken to extract discriminative feature vectors. Support vector machine (SVM) [42] is used as a classifier for constructing a prediction model.

#### A. Extract Feature Vector from the Position-Specific Scoring Matrix

Position-specific scoring matrix (PSSM) derived features have been widely used in bioinformatics including intrinsic disorder prediction [43]–[45], protein secondary structure prediction [46], transmembrane helix prediction [47]–[49], protein 3D structure prediction [50], and protein-ligand binding prediction [14], [51]. In this study, we obtain the PSSM of a query protein sequence by performing PSI-BLAST [52] to search the Swiss-Prot database through three iterations and with 0.001 as the *E*-value cutoff against the query sequence. To facilitate the subsequent computation, we further normalize each score, denoted as 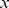, that is contained in the PSSM using the logistic function 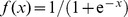. Based on the normalized PSSM, the feature vector, denoted *LogisticPSSM*, for each residue in the protein sequence can be extracted by applying a sliding-window technique, as follows [25], [26]: for a residue at position 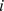 along the query sequence, its *LogisticPSSM* feature vector consists of the normalized PSSM scores of the query sequence that correspond to a sequence segment of length 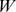 that is centered on 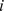. It has been demonstrated that *W* = 17 is a better choice for several protein-ligand binding residue prediction studies [25], [26]. Consequently, the dimensionality of the *LogisticPSSM* feature vector of a residue is 17×20 = 340-D.

#### B. Extract Feature Vector from the Predicted Protein Secondary Structure

PSIPRED [53], which has been widely used in bioinformatics [54], [55], can predict the probabilities of each residue in a query protein sequence belonging to three secondary structure classes, i.e., coil, helix, and strand. We obtained the predicted protein secondary structure by performing PSIPRED against the query sequence. The obtained predicted secondary structure is an *L*×3 probability matrix, where *L* is the length of the protein sequence. Similar to the *LogisticPSSM* feature extraction, we can extract a 17×3 = 51-D feature vector, denoted as PSS, for each residue in the protein by applying a sliding window of size 17.

The final discriminative feature vector of a residue is formed by serially combining its *LogisticPSSM* feature with the corresponding *PSS* feature, and the dimensionality of the obtained feature vector for the residue is 340+51 = 391-D.

#### C. Support Vector Machine

Support vector machine (SVM), which was proposed by Vapnik [42], has been widely used in a variety of bioinformatics fields, including the protein-nucleotide binding residue prediction [13], [14] considered in this study. In view of this, we will also use SVM as the base-learning model to evaluate the efficacy of the proposed SOS algorithm. Here, we will briefly introduce the basic idea of SVM.

Let 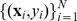 be the set of samples, where 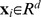 and 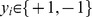 are the feature vector and the corresponding label of the *i*-th sample, respectively, and +1 and −1 are the labels of positive class and negative class, respectively.

In linearly separable cases, SVM constructs a hyperplane that separates the samples of two classes with a maximum margin. The optimal separating hyperplane (OSH) is constructed by finding another vector, 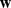, and a parameter, 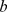, that minimizes 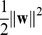 and satisfies the following conditions:
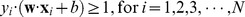
(1)where 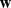 is a vector normal to the hyperplane, and 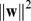 is the Euclidean norm of 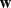.

The solution is a unique, globally optimized result with the following expansion:
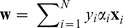
(2)


Support vectors are those 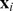, whose corresponding 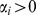.

Once the 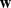 and 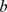 are found, a query input 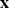 can be classified as follows:
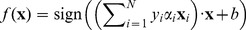
(3)


To allow for mislabeled examples, Corinna Cortes and Vladimir N. Vapnik suggested a modified maximum margin idea, i.e., “soft margin” technique [56].

For each training sample, a corresponding slack variable is introduced: 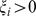, 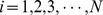. Accordingly, the relaxed separation constraint is given as:
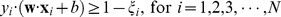
(4)


Then, the OSH can be solved by minimizing.
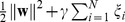
(5)where 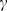 is the regularization parameter.

Furthermore, to address non-linearly separable cases, the “kernel substitution” technique is introduced as follows: first, the input vector 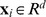 is mapped into a higher dimensional Hilbert space, *H,* by a non-linear kernel function, 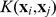; then, the OSH in the mapped space, *H*, is solved using a procedure similar to that for a linear case, and the decision function is given by:
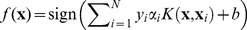
(6)


To train a SVM on a given data set, the kernel function and the regularity parameter 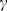 need to be specified in advance. In this study, LIBSVM [57] (http://www.csie.ntu.edu.tw/~cjlin/libsvm/) is taken. The Gaussian kernel 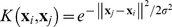, which is one of the most commonly used kernel functions, is chosen as the kernel function. The regularization parameter 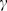 and the kernel width parameter 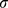 are optimized based on 10-fold cross-validation using a grid search strategy in the LIBSVM [57] software.

### Dealing with Class Imbalance: A New Supervised Over-Sampling Method

As described in the introduction section, protein-nucleotide binding residue prediction is a typical imbalanced learning problem. By revisiting Table 1, we can easily find that a severe class imbalance phenomenon does exist among both training datasets and independent validation datasets: the ratio of the number of non-binding residues to that of binding residues is often larger than 20.

In this study, we propose a new SOS algorithm for relieving the severity of class imbalance to facilitate the subsequent statistical machine learning methods. To demonstrate the effectiveness of the proposed SOS, several popular over-sampling methods, including ROS, SMOTE [39], and ADASYN [41], are used to perform comparisons with the proposed SOS.

#### A. Random Over-sampling

In the ROS technique, the minority set 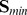 is augmented by replicating randomly selected samples within the set.

Although ROS is simple and easy to perform, a potential problem is that the resulting dataset tends to be over-fitted because ROS simply appends replicated samples to the original dataset; thus, multiple instances of certain samples become “tied” [58]. In view of this issue, several improved over-sampling techniques, e.g., SMOTE [39] and ADASYN [41], have been proposed and have shown promising results in various imbalanced applications. In this study, two improved over-sampling techniques, i.e., SMOTE [39] and ADASYN [41], were considered.

#### B. Synthetic Minority Over-sampling Technique

The SMOTE method [39] augments the minority class set 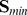 by creating artificial samples based on the feature space similarities between existing minority samples. The SMOTE procedure is briefly described below.

For each sample 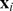 in 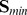, let 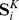 be the set of the *K*-nearest neighbors of 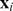 in 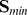 under the Euclidian distance metric. To synthesize a new sample, an element in 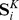, denoted as 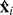, is selected and then multiplied by the feature vector difference between 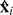 and 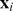 and by a random number between [0, 1]. Finally, this vector is added to 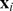:
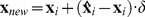
(7)where 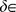
[0, 1] is a random number.

These synthesized samples help break the ties introduced by ROS and augment the original dataset in a manner that, in general, significantly improves subsequent learning [28].

#### C. Adaptive Synthetic Sampling

SMOTE creates the same number of synthetic samples for each original minority sample without considering the neighboring majority samples, which increases the occurrence of overlapping between classes [28]. In view of this limitation, various adaptive over-sampling methods, e.g., ADASYN [41], have been proposed.

ADASYN uses a systematic method to adaptively create different numbers of synthetic samples for different original minority samples according to their distributions. The ADASYN procedure is briefly described below.

The number of samples that must be synthesized for the entire minority class is computed first:
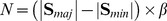
(8)where 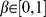 is a parameter that determines the balance level after the ADASYN process.

Then, for each original sample, 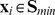, its *K*-nearest neighbors are found according to the Euclidean distance metric, and the distribution function, 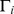, which is defined as:
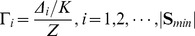
(9)is calculated, where 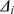 is the number of samples in the *K*-nearest neighbors of 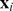 that belong to 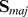, and 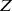 is a normalization constant so that 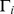 is a distribution function, i.e., 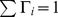.

Next, the number of synthetic samples that must be generated for each 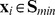 is computed:
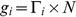
(10)


Finally, for each 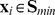, 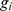 synthetic samples are generated according to Eq. (7), as in SMOTE.

The key difference between ADASYN and SMOTE is that the former uses a density distribution, 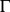, as a criterion to automatically decide the number of synthetic samples that must be generated for each minority sample by adaptively changing the weights of the different minority samples to compensate for the skewed distributions [28], [41]. The latter generates the same number of synthetic samples for each original minority sample.

#### D. Proposed Supervised Over-sampling

Let 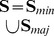 be the training dataset, where 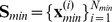 is the minority class sample set, and 
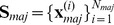
 is the majority class sample set. The purpose of the proposed SOS algorithm is to obtain a relatively balanced dataset, denoted as 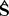, by synthesizing additional minority class samples under a supervised process.

Let 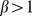 be the parameter of the over-sampling coefficient, which is a scalar quantity that measures the ratio of the size of the minority class sample set after over-sampling to that of the original minority class sample set. In other words, 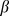 controls how many additional minority samples will be generated. More additional minority samples will be synthesized with larger values of 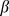.

The process of the proposed SOS is described as follows:

Step I: Training an initial classifier model, denoted as 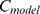, on the original training dataset 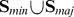:
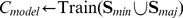
(11)


The trained classifier model will be used to judge whether a synthesized minority class sample is valid.

Step II: Synthesizing an additional minority sample:

First, two samples, denoted as 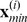 and 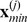, will be randomly selected from the minority class sample set 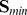:
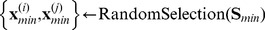
(12)


According to the two randomly selected minority class samples, an additional sample can be synthesized:
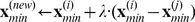
(13)where 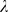 is a random value ranging from 0 to 1.

Then, the confidence of the synthesized sample, 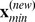, being a minority class sample is predicted using the trained initial classifier model 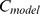:
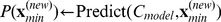
(14)


The validity of the synthesized sample depends on its confidence. More specifically, the synthesized sample is a valid minority class sample if and only if 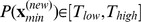, i.e., its confidence lies within the prescribed confidence interval 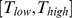.

Step II is repeated until the 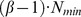 valid minority class samples have been synthesized.

Algorithm 1 summarizes the proposed SOS. Note that the three parameters, i.e., 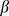, 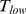, and 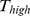, are problem-dependent. In this study, we set 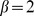, 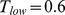, and 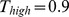.

Note that in Step II, it is straightforward and reasonable that a synthesized sample will not be considered valid when its confidence is less than the prescribed lower confidence, 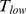. However, a synthesized sample will also be considered invalid if its confidence is larger than the prescribed upper confidence, 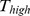. The underlying reason for this choice is that we believe that a synthesized sample with confidence that is too high tends to become “tied” with those true minority class samples, thus potentially leading to an over-fitting problem.

**Algorithm 1.** Supervised Over-Sampling (SOS)

**INPUT**: 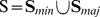
- The training dataset, where 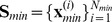 is the minority class sample set and 
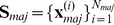
 is the majority class sample set; 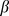
- The over-sampling coefficient, which is the size of the minority class after over-sampling, divided by that of the original minority class; 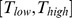
- The confidence interval, which is used to determine whether a synthetic sample belongs to the minority class.

**OUTPUT:** 
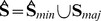
- The over-sampled training dataset, where 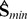 is the minority class sample set after over-sampling.

1. Training a classifier model, denoted as 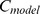, using the original training set 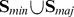:
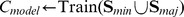



2. 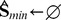


3. WHILE 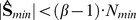


4. Randomly select two samples, denoted as 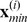 and 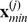, from 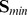:
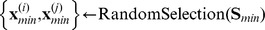



5. Synthesize a new sample:
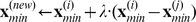



where 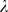 is a random value ranging 0 from 1;

6. Predict the confidence of 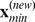 being a minority class sample:
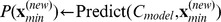



7. IF 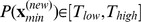


8. 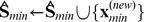


9. END IF

10. END WHILE

11. 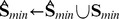


12. 
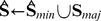


13. RETURN 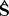


### Evaluation Indexes

Let 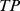, 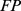, 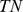, and 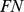 be the abbreviations for true positive, false positive, true negative, and false negative, respectively. Then, 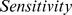
(*Sen*), 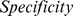
(*Spe*), 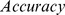
(*Acc*), and the Matthews correlation coefficient (*MCC*) can be defined as follows:
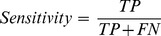
(15)




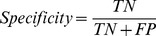
(16)




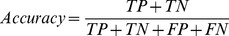
(17)




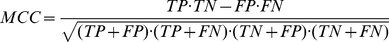
(18)


However, these four evaluation indexes are threshold-dependent, i.e., the values of these indexes vary with the threshold that is used in the prediction model. Considering that the *MCC* measures the overall quality of the binary predictions, we reported these threshold-dependent evaluation indexes by choosing the threshold that maximizes the value of the *MCC* of the predictions (termed *MaxMCC Evaluation* in this study).

It has not escaped our notice that several predictors reported their performances by selecting the threshold that balances the values of *Sen* and *Spe* [13], [25], [26] (termed *Balanced Evaluation* in this study). For the purpose of a fair comparison, we also used *Balanced Evaluation* when comparing the proposed method with these predictors.

In addition, the *A*rea *U*nder the receiver operating characteristic (ROC) *C*urve (*AUC*), which is threshold-independent and increases in direct proportion to prediction performance, was used to evaluate the overall prediction qualities of the considered prediction models.

## Experimental Results and Analysis

### Supervised Over-Sampling Helps to Enhance Prediction Performance

**Figure 1:**
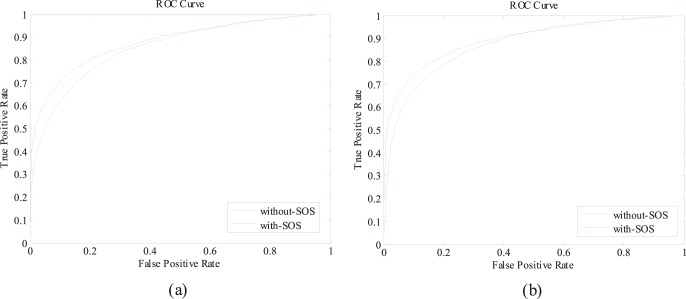
ROC curves of with-SOS and without-SOS predictions for ATP168 and ATP227 over five-fold cross-validation. (a) ROC curves for ATP168; (b) ROC curves for ATP227.

In this section, we empirically demonstrate that the performance of protein-nucleotide binding residue prediction can be further improved by applying the proposed SOS algorithm. Tables 2 and 3 summarize the performance comparisons between with-SOS and without-SOS for ATP168 and ATP227 over five-fold cross-validation under *Balanced Evaluation* and *MaxMCC Evaluation*, respectively. Figure 1 (a) and (b) illustrate the ROC curves of with-SOS and without-SOS for ATP168 and ATP227 over five-fold cross-validation. The results listed in Tables 2 and 3 show that the prediction performances are remarkably improved after SOS is applied. An improvement in the *AUC* of over 2% is observed for both the ATP168 and ATP227 datasets. In addition, the other four indexes, i.e., *Sen*, *Spe*, *Acc*, and *MCC*, of the with-SOS predictions are consistently higher than that of the without-SOS predictions. Taking *MCC* as an example, improvements of 5% and 4% are observed for ATP168 and ATP227, respectively, under *Balanced Evaluation,* whereas improvements of 12% and 8% are achieved for ATP168 and ATP227, respectively, under *MaxMCC Evaluation*.

**Table 2:** Performance comparisons of with-SOS and without-SOS predictions for ATP168 and ATP227 over five-fold cross-validation under *Balanced Evaluation*.

Dataset	Upper-Sampling	*Sen* (%)	*Spe* (%)	*Acc* (%)	*MCC*	*AUC*
ATP168	with-SOS	**80.0**	**80.1**	**80.1**	**0.311**	**0.878**
	without-SOS	75.2	77.2	77.1	0.262	0.843
ATP227	with-SOS	**81.3**	**81.7**	**81.7**	**0.306**	**0.893**
	without-SOS	79.0	79.1	79.1	0.266	0.871

**Table 3:** Performance comparisons of with-SOS and without-SOS predictions for ATP168 and ATP227 over five-fold cross-validation under *MaxMCC Evaluation*.

Dataset	Upper-Sampling	*Sen* (%)	*Spe* (%)	*Acc* (%)	*MCC*	*AUC*
ATP168	with-SOS	**42.3**	**99.2**	**96.3**	**0.536**	**0.878**
	without-SOS	35.2	98.5	95.3	0.415	0.843
ATP227	with-SOS	**46.3**	**99.2**	**97.0**	**0.553**	**0.893**
	without-SOS	40.1	98.9	96.5	0.473	0.871

### Comparisons with Other Over-Sampling Methods

In this section, we compare the proposed SOS with several other popular over-sampling methods, including ROS, SMOTE [39], and ADASYN [41].

Table 4 shows comparisons of the performance of SOS, ROS, SMOTE, and ADASYN for ATP168 and ATP227 over five-fold cross-validation under *MaxMCC Evaluation*. The results for the four other types of nucleotide ligands, i.e., ADP, AMP, GTP, and GDP, can be found in Supporting Information S2.

**Table 4:** Performance comparisons between SOS and ROS, SMOTE, and ADASYN for ATP168 and ATP227 over five-fold cross-validation under *MaxMCC Evaluation*.

Dataset	Over-Sampling Method	*Sen* (%)	*Spe* (%)	*Acc* (%)	*MCC*	*AUC*
	SOS	**42.3**	**99.2**	**96.3**	**0.536**	**0.878**
ATP168	ADASYN [41]	41.7	99.0	96.1	0.512	0.877
	SMOTE [39]	41.4	99.0	96.1	0.511	0.860
	ROS	39.2	98.8	95.8	0.474	0.846
	SOS	46.3	**99.2**	**97.0**	**0.553**	0.893
ATP227	ADASYN [41]	**46.5**	98.9	96.8	0.537	**0.896**
	SMOTE [39]	44.7	99.0	96.8	0.526	0.880
	ROS	42.9	99.1	96.9	0.522	0.876

From Table 4, it is clear that the proposed SOS significantly outperforms ROS for both ATP168 and ATP227. Taking *AUC* and *MCC*, which are two overall measurements of prediction quality, as examples, average improvements of approximately 3% and 5% are observed. We also found that the proposed SOS achieves comparable performance to ADASYN and slightly outperforms SMOTE for ATP168 and ATP227. Similar phenomenon could also be found for the four other types of nucleotide ligands (refer to Supporting Information S2).

The results listed in Table 4 and Supporting Information S2 show that the proposed SOS performs much better than ROS and can achieve comparable performances to ADASYN and SMOTE, which demonstrates the efficacy of the proposed SOS.

### Comparisons with Existing Predictors

In this section, we compare the proposed predictor, called TargetSOS, to the existing popular protein-nucleotide binding residue predictors to demonstrate its efficacy. TargetSOS performs predictions using a SVM model, which is trained with the proposed SOS algorithm in the NUC5 dataset and uses the *LogisticPSSM*+*PSS* feature as the model input. The comparisons are performed for both the cross-validation test and the independent validation test. Note that when cross-validation comparisons are performed for ATP168, only the *Balanced Evaluation* results are reported because the results for most existing predictors that are constructed from ATP168 are reported under *Balanced Evaluation*. For the same reason, cross-validation comparisons for the NUC5 dataset are reported under *MaxMCC Evaluation*.

#### A. Cross-Validation Test

Table 5 lists the performance comparisons of the proposed TargetSOS, TargetATP [26], TargetATPsite [25], and ATPint [13] for ATP168 over five-fold cross-validation under *Balanced Evaluation*. By observing Table 5, we find that the proposed TargetSOS significantly outperforms ATPint and is the best performer among the four considered predictors that were specifically designed for protein-ATP binding residue prediction. An over 5% improvement is observed for each of the five considered evaluation indexes, i.e., *Sen*, *Spe*, *Acc*, *MCC*, and *AUC*. In addition, TargetSOS performs better, although not significantly better, than the two most recently released predictors, i.e., TargetATP [26] and TargetATPsite [25].

**Table 5:** Performance comparisons between the proposed TargetSOS, TargetATP, and TargetATPsite for ATP168 over five-fold cross-validation under *Balanced Evaluation*.

Predictor	*Sen* (%)	*Spe* (%)	*Acc* (%)	*MCC*	*AUC*
TargetSOS	**80.0**	**80.1**	**80.1**	**0.311**	**0.878**
TargetATP [26]	79.1	79.8	79.8	0.308	0.873
TargetATPsite [25]	78.2	78.4	78.4	0.290	0.860
ATPint [13]	74.4	75.8	75.1	0.249	0.823

Table 6 summarizes the performance comparisons between the proposed TargetSOS and several other popular protein-nucleotide binding residue predictors for the NUC5 dataset over five-fold cross-validation under *MaxMCC Evaluation*. It is found that the proposed TargetSOS almost always achieves the best performance, with only one exception for ATP concerning *MCC* and *AUC*, which are two evaluation indexes that measure the overall prediction quality of a predictor. Taking *MCC* as an example, TargetSOS achieves improvements of approximately 3%, 8%, 6%, 7%, and 3% for ATP, ADP, AMP, GDP, and GTP, respectively, compared with the second-best performer (i.e., TargetATPsite [25] for ATP and NsitePred [14] for ADP, AMP, GDP, and GTP). The underlying reason for the improvement in *MCC* is that the TargetSOS can achieve much higher performance with respect to the true positive rate (i.e., *Sen*) while simultaneously achieving comparable or even slightly better performances for the true negative rate (i.e., *Spe*). We believe that this improvement may be a result of the SOS technique.

**Table 6:** Performance comparisons between the proposed TargetSOS and other popular predictors for the NUC5 dataset over five-fold cross-validation under *MaxMCC Evaluation*.

Ligand Type	Predictor	*Sen* (%)	*Spe* (%)	*Acc* (%)	*MCC*	*AUC*
	TargetSOS	**46.3**	**99.2**	**97.0**	**0.553**	0.893
	TargetATP [26]	41.2	99.0	96.6	0.501	**0.895**
ATP	TargetATPsite [25]	44.5	98.9	96.6	0.520	0.881
	NsitePred*	44.4	98.2	96.0	0.460	0.861
	SVMPred*	36.1	98.8	96.2	0.433	0.854
	TargetSOS	**60.5**	99.1	**97.7**	**0.653**	**0.914**
ADP	NsitePred*	54.4	98.8	97.1	0.572	0.893
	SVMPred*	45.8	**99.3**	97.3	0.555	0.885
	TargetSOS	**38.1**	98.8	**96.4**	**0.440**	**0.850**
AMP	NsitePred*	30.4	98.8	96.2	0.377	0.829
	SVMPred*	20.8	**99.6**	96.6	0.360	0.820
	TargetSOS	**66.1**	**99.5**	**98.2**	**0.744**	**0.923**
GDP	NsitePred*	64.6	99.1	97.6	0.675	0.910
	SVMPred*	62.3	98.9	97.7	0.655	0.905
	TargetSOS	**47.3**	99.5	**97.4**	**0.598**	**0.850**
GTP	NsitePred*	47.3	99.1	96.8	0.562	0.844
	SVMPred*	37.3	**99.7**	97.0	0.551	0.836

* Data excerpted from [14].

#### B. Independent Validation Test

It has been routine procedure to evaluate the generalization capability of a predictor using an independent validation test because evaluating a newly developed predictor by only comparing it to existing predictors and by using the same datasets may potentially lead to optimistically biased results, in the sense that the new predictor’s characteristics over-fit the used datasets [59]. Considering this potential bias, we also performed independent validation tests for the proposed TargetSOS and compared their performances with those of several other popular sequence-based protein-nucleotide binding residue predictors, as shown in Table 7.

**Table 7:** Performance comparisons between the proposed TargetSOS and other popular predictors for the independent validation dataset of NUC5.

Ligand Type	Predictor	*Sen* (%)	*Spe* (%)	*Acc* (%)	*MCC*	*AUC*
	TargetSOS	**53.6**	**99.2**	**97.6**	**0.603**	**0.912**
	TargetATP [26]	48.9	98.9	96.9	0.542	**0.912**
ATP	TargetATPsite [25]	45.8	99.1	97.2	0.530	0.882
	NsitePred*	46.0	98.5	96.7	0.476	0.875
	SVMPred*	36.7	99.1	96.9	0.451	0.868
	TargetSOS	**60.0**	98.5	97.0	**0.585**	**0.912**
ADP	NsitePred*	47.4	98.7	96.8	0.512	0.893
	SVMPred*	38.8	**99.3**	**97.1**	0.500	0.886
	TargetSOS	**45.6**	98.9	96.7	**0.522**	**0.880**
AMP	NsitePred*	42.3	98.7	**96.9**	0.501	0.876
	SVMPred*	33.5	**99.4**	96.7	0.478	0.870
	TargetSOS	49.1	**99.1**	97.2	0.562	0.866
GDP	NsitePred*	**58.5**	98.5	97.0	**0.576**	**0.867**
	SVMPred*	51.1	98.8	**97.1**	0.553	0.855
	TargetSOS	**61.9**	98.8	**97.1**	**0.655**	0.900
GTP	NsitePred*	60.4	98.8	96.9	0.640	**0.909**
	SVMPred*	48.5	**99.3**	96.9	0.602	0.887

*Data excerpted fdrom [14].

From Table 7, we find that the *AUC*s for ATP, ADP, AMP, GDP, and GTP when using TargetSOS in the corresponding independent validation datasets are 0.912, 0.912, 0.880, 0.866, and 0.900, respectively. By revisiting Table 6, it is found that the *AUC*s of TargetSOS for ATP, ADP, AMP, GDP, and GTP on the training datasets are 0.893, 0.914, 0.850, 0.923, and 0.850, respectively. In other words, TargetSOS achieves similar overall prediction performances (measured by *AUC*s) on the training dataset and the corresponding independent validation dataset for all five nucleotide ligands, indicating that the generalization capability of the TargetSOS that is derived from the knowledge buried in the training datasets has not been under- or over-estimated.

In addition, we find that the proposed TargetSOS achieves comparable overall performance (*AUC*) to the state-of-the-art sequence-based predictors considered in this study. On the other hand, TargetSOS almost always achieves the best performances for *MCC,* with only one exception for GDP, and an average improvement of approximately 3% is observed compared with the second-best performer (i.e., TargetATP [26] for ATP and NsitePred [14] for ADP, AMP, GDP, and GTP).

## Conclusion

In this study, a new SOS algorithm that balances the samples of different classes by synthesizing additional samples for minority class with a supervised process is proposed to address imbalanced learning problems. We apply the proposed SOS algorithm to protein-nucleotide binding residue prediction, and a web-server, called TargetSOS, is implemented. Cross-validation tests and independent validation tests on two benchmark datasets demonstrate that the proposed SOS algorithm helps to improve the performance of protein-nucleotide binding residue prediction. The findings of this study enrich the understanding of class imbalance learning and are sufficiently flexible to be applied to other bioinformatics problems in which class imbalance exists, such as protein functional residue prediction and disulfide bond prediction.

## Supporting Information

Supporting Information S1 **Datasets used in this study.**
(DOC)

Supporting Information S2 **Performance comparisons between different over-sampling techniques on the ADP, AMP, GTP, GDP sub-datasets in NUC5.**
(DOC)
