# Supplementary material for: A New Supervised Over-Sampling Algorithm with Application to Protein-Nucleotide Binding Residue Prediction
Source: PLoS One. 2014 Sep 17;9(9):e107676. doi: 10.1371/journal.pone.0107676 (PMC4168127; doi:10.1371/journal.pone.0107676)
Supplement: Supporting Information S1 — Datasets used in this study. (DOC) [file pone.0107676.s001.doc]

Supporting Information S1: Datasets Used in This Study

**A New Supervised Over-Sampling Algorithm with Application to Protein-Nucleotide Binding Residue Prediction**

Jun Hu^1^, Xue He^1^, Dong-Jun Yu^1, 3,*^, Xi-Bei Yang^1, 4^, Jing-Yu Yang^1^, and Hong-Bin Shen^2,*^

^1^ School of Computer Science and Engineering, Nanjing University of Science and Technology, Xiaolingwei 200, Nanjing, China, 210094

^2^ Institute of Image Processing and Pattern Recognition, Shanghai Jiao Tong University, Dongchuan Road 800, Shanghai, China, 200240

^3^ Changshu Institute, Nanjing University of Science and Technology, Changshu 215513, PR China

^4^ School of Computer Science and Engineering, Jiangsu University of Science and Technology, Huanchenlu 200, Zhenjiang, China, 212003

* Address correspondence to D.J. Yu [njyudj@njust.edu.cn](mailto:njyudj@njust.edu.cn) or H.B. Shen at [hbshen@sjtu.edu.cn](mailto:hbshen@sjtu.edu.cn)

Tel: +86-21-34205320

Fax: +86-21-34204022

Contents

[Readme 2](#__RefHeading___Toc396642134)

[Cross-Validation Datasets 3](#__RefHeading___Toc396642135)

[#ATP168 3](#__RefHeading___Toc396642136)

[#ATP of NUC5 48](#__RefHeading___Toc396642137)

[#ADP of NUC5 110](#__RefHeading___Toc396642138)

[#AMP of NUC5 201](#__RefHeading___Toc396642139)

[#GDP of NUC5 235](#__RefHeading___Toc396642140)

[#GTP of NUC5 263](#__RefHeading___Toc396642141)

[Independent Validation Datasets 280](#__RefHeading___Toc396642142)

[#ATP of NUC5 280](#__RefHeading___Toc396642143)

[#ADP of NUC5 285](#__RefHeading___Toc396642144)

[#AMP of NUC5 293](#__RefHeading___Toc396642145)

[#GDP of NUC5 298](#__RefHeading___Toc396642146)

[#GTP of NUC5 300](#__RefHeading___Toc396642147)

# Readme

Each protein sequence and the labels of the residues in it are represented as follows:

>Protein Name

Protein Sequence Line

Protein Label Line

For example:

>1JWA_D

MIKVLFFAQVRELVGTDATEVAADFPTVEALRQHMAAQSDRWALALEDGKLLAAVNQTLVSFDHPLTDGDEVAFFPPVTgg

222222222222222222222222222222222222222222222222222222222222222222222222222222211

where the label '1' denote that the corresponding residue in the sequence is a binding residue; while the label '2' represents that the corresponding residue in the sequence is a non-binding residue.

# Cross-Validation Datasets

## #ATP168

>1JWA_D

MIKVLFFAQVRELVGTDATEVAADFPTVEALRQHMAAQSDRWALALEDGKLLAAVNQTLVSFDHPLTDGDEVAFFPPVTgg

222222222222222222222222222222222222222222222222222222222222222222222222222222211

>1N5I_A

MLIAIEGVDGAGKRTLVEKLSGAFRAAGRSVAtlaFPrYGqSVAADIAAEALHGEHGDLASSVYAMATLFALDRAGAVhtIQglCRGyDVVILDRYVASNAAYSAARLHENAAGKAAAWVQRIEFARLGLPKPDWQVLLAVSAELAGERSRGRAQRDPGRARDNYERDAELQQRTGAVYAELAAQGWGGRWLVVGADVDPGRLAATLAPPDVPS

2222222222222222222222222222222211122122122222222222222222222222222222222222221122112221222222222222222222222222222222222222222222222222222222222222222222222222222222222222222222222222222222222222222222222222222222

>2ARU_A

MEGRLLLLETPGNTRMSLAYDEAIYRSFQYGDKPILRFYRHDRSVIIGYFQVAEEEVDLDYMKKNGIMLARrYTGggavyhDLGdLNFSVVRSSDDMDITSMFRTMNEAVVNSLRILGLDARPGELNDvsipvNkKtDIMAGEKkImgaaGAMRKGAKLWhAaMlVHTDLDMlSAVlKvPDEKFRDKIAKStRERvANVTDFVDVSIDEVRNALIRGFSETLHIDFREDTITEKEESLARELFDKKYSTEEWNMGLLRKEVV

2222222222222222222222222222222222222222222222222222222222222222222222212221111112221222222222222222222222222222222222222222222211111212122222221211112222222222121212222222122212122222222222212221222222222222222222222222222222222222222222222222222222222222222222

>2QK4_A

MHHHHHHSSGVDLGTENLYFQSMAARVLIIGSGGREHTLAWKLAQSHHVKQVLVAPGNAGTACSEKISNTAISISDHTALAQFCKEKKIEFVVVGPEAPLAAGIVGNLRSAGVQCFGPTAEAAQLESSKRFAKEFMDRHGIPTaQWKAFTKPEEACSFILSADFPALvVkASGLAAGKGvivAkSKEEACKAVQEIMQEKAFGAAGETIVIeellDGEeVSCLCFTDGKTVAPMPPAQDHkrLLEGDGGpnTGGMGAYCPAPQVSNDLLLKIKDTVLQRTVDGMQQEGTPYTGILYAGImLTKNGPKVleFNCRFGDPECQVILPLLKSDLYEVIQSTLDGLLCTSLPVWLENHTALTVVMASKGYPGDYTKGVEITGFPEAQALGLEVFHAGTALKNGKVVTHGGRVLAVTAIRENLISALEEAKKGLAAIKFEGAIYRKDIGFRAIAFLQ

22222222222222222222222222222222222222222222222222222222222222222222222222222222222222222222222222222222222222222222222222222222222222222222222122222222222222222222222121222222222111212222222222222222222222222221111222122222222222222222222211222222211222222222222222222222222222222222222222222222222122222222112222222222222222222222222222222222222222222222222222222222222222222222222222222222222222222222222222222222222222222222222222222222222222222222

>2RD5_C

MQISSDYIPDSKFYKVEAiVRPWRIQQVSSALLKIGIRGVTVSDvrgfgAqGGsTeRHGGSEFSEDKFVAkVKMEIVVKKDQVESVINTIIEGARTGEigdgkIfVLPVSDVIRVRTGERGEKAEKMTGDMLSPS

222222222222222222122222222222222222222222221111121221212222222222222212222222222222222222222222221111121222222222222222222222222222222

>1GTR_A

SEAEARPTNFIRQIIDEDLASGKHTTVHTRfppePnGYLhIghAksICLNFGIAQDYKGQCNLRFDDTNPVKEDIEYVESIKNDVEWLGFHWSGNVRYSSDYFDQLHAYAIELINKGLAYVDELTPEQIREYRGTLTQPGKNSPYRDRSVEENLALFEKMRAGGFEEGKACLRAKIDMASPFIVMRDPVLYRIKFAEHHQTGNKWCIYPMYDFTHCISDALEGITHSlctLefQDNRRLYDWVLDNITIPVHPRQYEfSrlNLEYTVmskrKLNLLVTDKHVEGWDDPRMPTISGLRRRGYTAASIREFCKRIGVTKQDNTIEMASLESCIREDLNENAPRAMAVIDPVKLVIENYQGEGEMVTMPNHPNKPEMGSRQVPFSGEIWIDRADFREEANKQYKRLVLGKEVRLRNAYVIKAERVEKDAEGNITTIFCTYDADTLSKDPADGRKVKGVIHWVSAAHALPVEIRLYDRLFSVPNPGAADDFLSVINPESLVIKQGFAEPSLKDAVAGKAFQFEREGYFCLDSRHSTAEKPVFNRTVGLRDTWAKVGE

2222222222222222222222222222221111212221211211222222222222222222222222222222222222222222222222222222222222222222222222222222222222222222222222222222222222222222222222222222222222222222222222222222222222222222222222222222222222211121122222222222222222222222212112222221111222222222222222222222222222222222222222222222222222222222222222222222222222222222222222222222222222222222222222222222222222222222222222222222222222222222222222222222222222222222222222222222222222222222222222222222222222222222222222222222222222222222222222222222222222222222222222222

>1DV2_B

GSHMLDKIVIANRGEIALRILRACKELGIKTVAVHSSADRDLKHVLLADETVCIGPAPSVKSYLNIPAIISAAEITGAVAIHPGYGFLSENANFAEQVERSGFIFIGPKAETIRLMGDkVSAIAAMKKAGVPCvPGSDGPLGDDMDKNRAIAKRIGYPViIkASGggggRGmRVVRGDAELAQSISMTRAEAKAAFSNDMVYMekylENpRhVEIQVLADGQGNAIYLAERDCSMqRRhQkVVEEAPAPGITPELRRYIGERCAKACVDIGYRGAGTFeFlFENGEFYFikMNTrIQVEHPVTEMITGVDLIKEQLRIAAGQPLSIKQEEVHVRGHAVECRINAEDPNTFLPSPGKITRFHAPGGFGVRWESHIYAGYTVPPYYDSMIGKLICYGENRDVAIARMKNALQELIIDGIKTNVDLQIRIMNDENFQHGGTNihYLEKKLGLQEK

22222222222222222222222222222222222222222222222222222222222222222222222222222222222222222222222222222222222222222222221222222222222221222222222222222222222222212122211112212222222222222222222222222222222111122121222222222222222222222221221212222222222222222222222222222222222222121222222221122212222222222222222222222222222222222222222222222222222222222222222222222222222222222222222222222222222222222222222222222222222222222222222222222221122222222222

>2NPI_B

MGSSHHHHHHSQDPNSASLPGIDEHTTSEELITGDNEWHKLVIPKGSdWqiDLKAEGKLIVKVNSGIVEIfGTELAVDDEYTFQNwkfpIYAVEETELLWKCPDLTTNTITVKPnHTmKYIYNLHFMLEKIRMSNFEGPRVVIVGGsqtgktsLSRTLCSYALKFNAYQPLYINLdPQqPIFTVPGCISATPISDILDAQLPTWGQSLTSGATLLHNKQPMVKNFGLERINENKDLYLECISQLGQVVGQRLHLDPQVRRSGCIVDtpsISQLDENLAELHHIIEKLNVNIMLVLCSETDPLWEKVKKTFGPELGNNNIFFIPKLdgvsAvDDVykRSLQRTSIREYFYGSLDTALSPYAIGVDYEDLTIWKPSNVFDNEVGRVELFPVTITPSNLQHAIIAITFAERRADQATVIKSPILGFALITEVNEKRRKLRVLLPVPGRLPSKAMILTSYRYLE

2222222222222222222222222222222222222222222222212112222222222222222222122222222222222111122222222222222222222222221221222222222222222222222222222211111112222222222222222222222122122222222222222222222222222222222222222222222222222222222222222222222222222222222222222211122222222222222222222222222222222222222222222222222222222111121222112222222222222222222222222222222222222222222222222222222222222222222222222222222222222222222222222222222222222222222222222222

>2A3Z_A

DEDETTALVCDNgsglVkAGFAGDDAPRAVFPSIVGRPRHQGVMVGMGQKDSYVGDEAQSKRGILTLKYPIEHgIITNWDDMEKIWHHTFYNELRVAPEEHPTLLTEAPLNPKANREKMTQIMFETFNVPAMYVAIqAVLSLYASGRTTGIVLdSgdgvTHNVPIYEGYALPHAIMRLDLAgRDLtDYLMKILTERGYSFVTTAEREIVrDIkeKLCYVALDFENEMATAASSSSLEKSYELPDGQVITIGNERFRCPETLFQPSFIGMESAGIHETTYNSIMKCDIDIRKDLYANNVMSggtTmypGIADRMQKEITALAPSTMKIKIIAPPERkySVWIGGSILASLSTFQQMWITKQEYDEAGPSIVHRKCF

222222222222111121222222222222222222222222222222222222222222222222222222212222222222222222222222222222222222222222222222222222222222222212222222222222222121111222222222222222222222212221222222222222222222222221221122222222222222222222222222222222222222222222222222222222222222222222222222222222222222111211122222222222222222222222222221122222222222222222222222222222222222222

>2I4O_A

MGSSHHHHHHSSGLVPRGSHMRLSRFFLPILKENPKEAEIVSHRLMLRAGMLRQEAAGIYAWLPLGHRVLKKIEQIVREEQNRAGAIELLMPTLQLADLWRESGRYDAYGPEMLRIADRHKRELLYGPTNEEMITEIFRAYIKSYKSLPLNLYHIQWKFrDeQRPRFGVMrGREfLmKDAYSFDVDEAGARKSYNKMFVAYLRTFARMGLKAIPMRAETGPIGGDLSHEFIVLAETGESGVYIDRDVLNLPVPDENVDYDGDLTPIIKQWTSVYAATEDVHEPARYESEVPEANRLNTRGIevgqIFYFGTKYSDSMKANVTGPDGTDAPIHGGSYgVgVSrLLGAIIEACHDDNGIIWPEAVAPFRVTILNLKQGDAATDAACDQLYRELSAKGVDVLYDDTDQRAGAKFATADLIGIPWQIHVGPRGLAEGKVELKRRSDGARENLALADVVARLT

22222222222222222222222222222222222222222222222222222222222222222222222222222222222222222222222222222222222222222222222222222222222222222222222222222222222222212122222222122212122222222222222222222222222222222222222222222222222222222222222222222222222222222222222222222222222222222222222222222222222221111222222222222222222222222222222212122122222222222222222222222222222222222222222222222222222222222222222222222222222222222222222222222222222222222222222222

>1XSC_A

GPLGSMALRACGLIIFRRCLIPKVDNNAIEFLLLQasdGiHhWtPPkGHVEPGEDDLETALRATQEEAGIEAGQLTIIEGFKRELNyVarNKPkTVIYWLAEVKDYDVEIRLSHeHQAYRWLGLEEACQLAQfKemKAALQEGHQFLCSIEAL

222222222222222222222222222222222221112121212212222222222222222222222222222222222222221211222122222222222222222222122222222222222222121122222222222222222

>2Z1U_A

GAMSGETLLLDYGSGGRASHRLISDLFLRHFDNPILGTLNDAARLDLTGPLAMSTdSYTVDPIFFPGGDIGTLAVHGTVNdVSMLGARPRYLSCGFILEEGLDMDILERVVASMGKAAREAGVFIVTGDTKVVPRGACDKMFINTTGIGEILVDPAPSGDRARPGDAILISGSMGDHGLTILSQRQGLNFAADVCSDSASLNRVVEKLVLEVGDIHVLrdPtrGGLATTLNeIAGQSQAVCHVLETAVPVRESVRNGCSFLGLDPLYLANeGKLICILPEERAEAALAVLREGPHGEHAARIGSVKSVGELGAARAGQVVMETALGGHRLLSMLEGEQLPRIc

2222222222222222222222222222222222222222222222222222222122222222222222222222222212222222222222222222222222222222222222222222222222222222222222222222222222222222222222222222222222222222222222222222222222222222222222222211211222222221222222222222222222222222222222222222221222222222222222222222222222222222222222222222222222222222222222222222221

>1QHX_A

MTTRMIILNGGssagksgIVrCLQSVLPEPWLAFGvdSLIEAMPLKMQSAEGGIEFDADGGVSIGPEFRALEGAWAEGVVAMARAGARIIIDDvFLGGAAAQERWRSFVGDLDVLWVGVRCDGAVAEGrETarGDrVAGmAAKQAYVVHEGVEYDVEVDtTHkesIECAWAIAAHVVP

2222222222211111112212222222222222211222222222222222222222222222222222222222222222222222222221222222222222222222222222222222222212211221222122222222222222222221221112222222222222

>2FSG_B

VFGSRNDRTLRRMRKVVNIINAMEPEMEKLSDEELKGKTAEFRARLEKGEVLENLIPEAFAVVREASKRVFgMrHfDVqLLGGMVLNERCIAEMRtgegktlTATLPAYLNALTGKGVHVVTVNDYLAQrDAENNRPLFEFLGLTVGINLPGMPAPAKREAYAADITYGTNNEYGFDYLRDNMAFSPEERVQRKLHYALVdeVDSILIDEARTPLIISGPAEDSSEMYKRVNKIIPHLIRQEKEDSETFQGEGHFSVDEKSRQVNLTERGLVLIEELLVKEGIMDEGESLYSPANIMLMHHVTAALRAHALFTRDVDYIVKDGEVIIVDEHTGRTMQGRRWSDGLHQAVEAKEGVQIQNENQTLASITFQNYFRLYEKLAGMTGTADTEAFEFSSIYKLDTVVVPTNRPMIRKDLPDLVYMTEAEKIQAIIEDIKERTAKGQPVLVGTISIEKSELVSNELTKAGIKHNVLNAKFHANEAAIVAQAGYPAAVTIATNMAGRGTDIVLGGSWQAEVAALENPTAEQIEKIKADWQVRHDAVLEAGGLHIIGTERHESRRIDNQLRGRSGRQGDAGSSRFYLSMEDALMRIFASDRVSGMMRKLGMKPGEAIEHPWVTKAIANAQRKVESRNFDIRKQLLEYDDVANDQRRAIYSQRNELLDVSDVSETINSIREDVFKATIDAYIPPQSLEEMWDIPGLQERLKNDFDLDLPIAEWLDKEPELHEETLRERILAQSIEVYQRKEEVVGAEMMRHFEKGVMLQTLDSLWKEHLAAMDYLRQGIHLRGYAQKDPKQEYKRESFSMFAAMLESLKYEVISTLSKVQVRMPEEVEELEQQRRMEAERLAQMQQLSHQD

2222222222222222222222222222222222222222222222222222222222222222222222212121221222222222222222211111112222222222222222222222222221222222222222222222222222222222222222222222222222222222222222222222222211222222222222222222222222222222222222222222222222222222222222222222222222222222222222222222222222222222222222222222222222222222222222222222222222222222222222222222222222222222222222222222222222222222222222222222222222222222222222222222222222222222222222222222222222222222222222222222222222222222222222222222222222222222222222222222222222222222222222222222222222222222222222222222222222222222222222222222222222222222222222222222222222222222222222222222222222222222222222222222222222222222222222222222222222222222222222222222222222222222222222222222222222222222222222222222222222222222222222222222222222222222222222222222222222222222222222222222222222222

>1O93_A

MNGPVDGLCDHSLSEEGAFMFTSESvGEGhpDKICDQISDAVLDAHLKQDPNAKVACETVCKTGMVLLCGEITSMAMIDYQRVVRDTIKHIGYDDSAKGFDFKTCNVLVALEQQSPDIAQCVHLDRNEEDVGAGDQGLMFGYATDETEECMPLTIVLAHKLNTRMADLRRSGVLPWLRPdSkTQVTVQYVQDNGAVIPVRVHTIVISVQHNEDITLEAMREALKEQVIKAVVPAKYLDEDTIYHLQPSGRFVIGgPQgdaGVTGrkIIVDTYGGWGAHGGGAFSGKDYTKVDRSAAYAARWVAKSLVKAGLCRRVLVQVSYAIGVAEPLSISIFTYGTSKKTERELLEVVNKNFDLRPGVIVRDLDLKKPIYQKTACYGHFGRSEFPWEVPKKLVF

222222222222222222222222212221122222222222222222222222222222222222222222222222222222222222222222222222222222222222222222222222222222222222222222222222222222222222222222222222222221212222222222222222222222222222222222222222222222222222222222222222222222221221112222112222222222222222222222222222222222222222222222222222222222222222222222222222222222222222222222222222222222222222222222222222222222

>1YID_B

MPFVDLEVPTMTTPTPAATPARPRVLTgdrPtGALhLghLAgsLQNrVRLQDEAELFVLLADVQALTdhFDRPEQVRENVLAVALDYLAAGLDPQKTTCVVQSAVPELAELTVYFLNLVTVSHLRQNPTVKAeIAQkGYGERVPAGFFVYPVSQAADIAAFGATLVPVGDDqLPMLEQTREIVRRFNALYAPVLAEPQAQLSRVPrlPGLDGQAkmsksLGNAIALGDSADEVARKVMGMYTDPGHLRASDPGRVEGNPVFTFLDAFDPDPARVQALKDQYRAGGLGDVKVKKHLIDVLNGVLAPIRTRRAEYERDPDAVLRFVTEGTARGREVAAQTLGQVRRAMRLFGH

222222222222222222222222222111212221211221122212222222222222222222211222222222222222222222222222222222222222222222222222222222222222122212222222222222222222222222222222222122222222222222222222222222222222211222222211111222222222222222222222222222222222222222222222222222222222222222222222222222222222222222222222222222222222222222222222222222222222222

>1OL6_A

ESKKRQWALEDFEIGRPlgkGkfgNvYLAREKQSKFILaLkVlFKAQLEKAGVEHQLRReVEIQSHLRHPNIlRLYGYFHDATRVYLIleyaPLgtVYRELQKLSKFDEQRTATYITELANALSYCHSKRVIHRDIKPenLlLGSAGELKIanFGWSVHAPSSRRTTLCGTLDYLPPEMIEGRMHDEKVDLWSLGVLCYEFLVGKPPFEANTYQETYKRISRVEFTFPDFVTEGARDLISRLLKHNPSQRPMLREVLEHPWITANSSKPSNCQNKESASKQS

222222222222222221112111212222222222221212122222222222222221222222222222122222222222222211112211222222222222222222222222222222222222222222112122222222211222222222222222222222222222222222222222222222222222222222222222222222222222222222222222222222222222222222222222222222222222222222

>1TYQ_A

MAGRLPACVVDCgtgyTKLGYAGNTEPQFIIPSCIAIKESAKVGDQAQRRVMKGVDDLDFFIGDEAIEKPTYATKWPIRHgIVEDWDLMERFMEQVIFKYLRAEPEDHYFLLTEPPLNTPENREYTAEIMFESFNVPGLYIAVQAVLALAASWTSRQVGERTLTGTVIdSgdgvThVIPVAEGYVIGSCIKHIPIAgRDITYFIQQLLRDREVGIPPEQSLETAkAVkeRYSYVCPDLVKEFNKYDTDGSKWIKQYTGINAISKKEFSIDVGYERFLGPEIFFHPEFANPDFTQPISEVVDEVIQNCPIDVRRPLYKNIVLSggsTmfRDFGRRLQRDLKRTVDARLKLSEELSGGRLKPKPIDVQVITHHMQrYAVWFGGSMLASTPEFYQVCHTKKDYEEIGPSICRHNPVFGVMS

2222222222221111222222222222222222222222222222222222222222222222222222222222222212222222222222222222222222222222222222222222222222222222222222222222222222222222222222221211112122222222222222222222122222222222222222222222222212211222222222222222222222222222222222222222222222222222222222222222222222222222222222222222222222111211222222222222222222222222222222222222222222222122222222222222222222222222222222222222222222

>2IAJ_A

PISPIETVPVKLKPGMDGPKVKQWPLTEEKIKALVEICTEMEKEGKISKIGPENPYNTPVFAIKKKDSTKWRKLVDFRELNKRTQDFWEVQLGIPHPAGLKKNKSVTVLdVgdayFSVPLDEDFRKYTAFTIPSINNETPGIRYQYNVLPqGWKGSPAIFQSSMTKILEPFKKQNPDIVICQymddLYVGSDLEIGQHRTKIEELRQHLLRWGLTTPDkKHQKEPPFLWmGYELHPDKWTVQPIVLPEKDSWTVNDIQKLVGKLNwASQIYPGIKVRQLSKLLRGTKALTEVIPLTEEAELELAENREILKEPVHGVYYDPSKDLIAEIQKQGQGQWTYQIYQEPFKNLKTGKYARMRGAHTNDVKQLTEAVQKITTESIVIWGKTPKFKLPIQKETWETWWTEYWQATWIPEWEFVNTPPLVKLWYQLEKEPIVGAETFYVDGAANRETKLGKAGYVTNKGRQKVVPLTNTTNQKTELQAIYLALQDSGLEVNIVTDSQYALGIIQAQPDKSESELVNQIIEQLIKKEKVYLAWVPAHKGIGGNEQVDKLVSAGIRKIL

22222222222222222222222222222222222222222222222222222222222222222222222222222222222222222222222222222222222221211112222222222222222222222222222222222212222222222222222222222222222222111122222222222222222222222222222222122222222221222222222222222222222222222222222221222222222222222222222222222222222222222222222222222222222222222222222222222222222222222222222222222222222222222222222222222222222222222222222222222222222222222222222222222222222222222222222222222222222222222222222222222222222222222222222222222222222222222222222222222222222222222222222222222222

>1FMW_A

MNPIHDRTSDYHKYLKVKQGDSDLFKLTVSDKRYIWYNPDPKERDSYECGEIVSETSDSFTFKTVDGQDRQVKKDDANQRNPIKFDGVEDMSELSYLNEPAVFHNLRVRYNQDLiyTYSGLFLVAVnpfkriPIyTQEMVDIFKGRRRNEVAPHIFAISDVAYRSMLDDRQNQSLLITGesgagktenTKKVIQYLASVAGRNQANGSGVLEQQILQANPILEAFGNAKTTRnNnssrFGKFIEIQFNNAGFISGASIQSYLLEKSRVVFQSETERNYHIFYQLLAGATAEEKKALHLAGPESFNYLNQSGCVDIKGVSDSEEFKITRQAMDIVGFSQEEQMSIFKIIAGILHLGNIKFEKGAGEGAVLKDKTALNAASTVFGVNPSVLEKALMEPRILAGRDLVAQHLNVEKSSSSRDALVKALYGRLFLWLVKKINNVLCQERKAYFIGVLDIsGFEIFKVNSFEQLCINYTNEKLQQFFNHHMFKVEQEEYLKEKINWTFIDFGLDSQATIDLIDGRQPPGILALLDEQSVFPNATDNTLITKLHSHFSKKNAKYEEPRFSKTEFGVTHYAGQVMYEIQDWLEKNKDPLQQDLELCFKDSSDNVVTKLFNDPNIASRAKKGANFITVAAQYKEQLASLMATLETTNPHFVRCIIPNNKqLPAKLEDKVVLDQLRCNGVLEGIRITRKGFPNRIIYADFVKRYYLLAPNVPRDAEDSQKATDAVLKHLNIDPEQYRFGITKIFFRAGQLARIEEAREPN

22222222222222222222222222222222222222222222222222222222222222222222222222222222222222222222222222222222222222222211222222222211111122122222222222222222222222222222222222222222222111111111222222222222222222222222222222222222222222221211112222222222222222222222222222222222222222222222222222222222222222222222222222222222222222222222222222222222222222222222222222222222222222222222222222222222222222222222222222222222222222222222222222222222222222222222222122222222222222222222222222222222222222222222222222222222222222222222222222222222222222222222222222222222222222222222222222222222222222222222222222222222222222222222222222222222222222222222222222222222222221222222222222222222222222222222222222222222222222222222222222222222222222222222222222222222222222222

>1TYQ_B

MDSQGRKVVVCDNGTGFVKCGYAGSNFPEHIFPALVGRPIIRSTTKVGNIEIKDLMVGDEASELRSMLEVNYPMENGIVRNWDDMKHLWDYTFGPEKLNIDTRNCKILLTEPPMNPTKNREKIVEVMFETYQFSGVYVAIQAVLTLYAQGLLTGVVVdSgdgvTHICPVYEGFSLPHLTRRLDIAgRDITRYLIKLLLLRGYAFNHSADFETVrMIkeKLcYVGYNIEQEQKLALETTVLVESYTLPDGRIIKVGGERFEAPEALFQPHLINVEGVGVAELLFNTIQAADIDTRSEFYKHIVLSggsTmypGLPSRLERELKQLYLERVLKGDVEKLSKFKIRIEDPPRRKHMVFLGGAVLADIMKDKDNFWMTRQEYQEKGVRVLEKLGVTVR

2222222222222222222222222222222222222222222222222222222222222222222222222222222222222222222222222222222222222222222222222222222222222222222222222222222222222121111222222222222222222222212222222222222222222222222221221122122222222222222222222222222222222222222222222222222222222222222222222222222222222222111211122222222222222222222222222222222222222222222222222222222222222222222222222222222222

>1KO5_B

MSTTNHDHHIYVLMGVsgsgksavASEVAHQLHAAFLDGDFLHPRRNIEKMASGEPLNDDDRKPWLQALNDAAFAMQRTNKVSLIVCsALKKHYRDLLREGNPNLSFIYLKGDFDVIESrlKArKGHFFKTQMLVTQFETLQEPGADETDVLVVDiDqplEGvVASTIEVIKKGK

2222222222222222111111112222222222222222222222222222222222222222222222222222222222222221222222222222222222222222222222211221222222222222222222222222222222212111221222222222222

>1MA9_B

DEDETTALVCDngsglVkAGFAGDDAPRAVFPSIVGRPRHQGVMVGMGQKDSYVGDEAQSKRGILTLKYPIEHgIITNWDDMEKIWHHTFYNELRVAPEEHPTLLTEAPLNPKANREKMTQIMFETFNVPAMYVAIQAVLSLYASGRTTGIVLdSgdgvTHNVPIYEGYALPHAIMRLDLAgrDLtDYLMKILTERGYSFVTTAEREIVrDIkeKLCYVALDFENEMATAASSSSLEKSYELPDGQVITIGNERFRCPETLFQPSFIGMESAGIHETTYNSIMKCDIDIRKDLYANNVMSggtTmypGIADRMQKEITALAPSTMKIKIIAPPERkySVWIGGSILASLSTFQQMWITKQEYDEAGPSIVHRKCF

222222222221111121222222222222222222222222222222222222222222222222222222212222222222222222222222222222222222222222222222222222222222222222222222222222222121111222222222222222222222211221222222222222222222222221221122222222222222222222222222222222222222222222222222222222222222222222222222222222222222111211122222222222222222222222222221122222222222222222222222222222222222222

>1NSF_A

GAHMGDFLASLEQDIKPAFGTNQEDYASyimngiiKwGDPvTRVLDDGELLVQQTKNSDRTPLVSVLLEGPphsgktalAAKIAEESNFPFIKICSPDKMIGFSETAKCQAMKKIFDDAYKSQLSCVVVDdIERLLDYVPIGPRFSNLVLQALLVLLKKAPPQGRKLLIIGTTsRKDVLQEMEMLNAFSTTIHVPNIATGEQLLEALELlGNFKDKERTTIAQQVKGKKVWIGikKLlMLIEMSLQMDPEYRVRKFLALLREEGASPLDFDLE

222222222222222222222222222211111112122212222222222222222222222222222221111111122222222222222222222222222222222222222222222222222212222222222222222222222222222222222222222221222222222222222222222222222222222221222222222222222222222221122122222222222222222222222222222222222

>3C5E_A

MGHHHHHHSSGVDLGTENLYFQSMSLQWGHQEVPAKFNFASDVLDHWADMEKAGKRPPSPALWWVNGKGKELMWNFRELSENSQQAANVLSGACGLQRGDRVAVVLPRVPEWWLVILGCIRAGLIFMPGTIQMKSTDILYRLQMSKAKAIVAGDEVIQEVDTVASECPSLRIKLLVSEKSCDGWLNFKKLLNEASTTHHCVETGSQEASAIYFtsgtsGLpkMAEHSYSSLGLKAKMDAGWTGLQASDIMWTISDTGWILNILCSLMEPWALGACTFVHLLPKFDPLVILKTLSSYPIKSMMGAPIVYRMLLQQDLSSYKFPHLQNCVTVgesLLPETLENWRAQTGLDIResygqteTGLTCMVSKTMKIKPGYmGTAASCYDVQIIDDKGNVLPPGTEGDIGIRVKPIRPIGIFSGYVDNPDKTAANIRGDFWLLGdRGIKDEDGYFQfMGrADDIINSSGYRIGPSEVENALMEHPAVVETAVISSPDPVRGEVVKAFVVLASQFLSHDPEQLTKELQQHVKSVTAPYKYPRKIEFVLNLPKTVTGkIQRAKLRDKEWKMSGKARAQ

222222222222222222222222222222222222222222222222222222222222222222222222222222222222222222222222222222222222222222222222222222222222222222222222222222222222222222222222222222222222222222222222222222222222222222222111112211222222222222222222222222222222222222222222222222222222222222222222222222222222222222222222222222222222222222111222222222222222222111111122222222222222222122222222222222222222222222222222222222222222222222222222222222122222222222122122222222222222222222222222222222222222222222222222222222222222222222222222222222222222222222222122222222222222222222

>2R7L_A

MISKDEILEIFDKYNKDEITIATLGSHTSLHILKGAKLEGFSTVCITMKGRDVPYKRFKVADKFIYVDNFSDIKNEEIQEKLRELNSIVVPHGSFIAYCGLDNVENSFLVPMFGNRRILRWESErSLEGKLLREAGLRVpKKYESPEDIDGTViVkFPGARGGRGyFIASSTEEFYKKAEDLKKRGILTDEDIANAHIeeyvVGTnFCIHYFYSPLKDEVELLGMDKrYeSNIDGLVRIPAKDQLEMNINPSyViTGNIPVVIRESLLPQVFEMGDKLVAKAKELVPPGMIGPFCLqSlCNENLELVVfeMSARVdGGTNSFMNGGPYSFLYNGEPLSMGQRIAREIKMALQLDMIDKIIS

2222222222222222222222222222222222222222222222222222222222222222222222222222222222222222222222222222222222222222222222222222122222222222222122222222222221212222222221222222222222222222222222222222221111222122222222222222222222212122222222222222222222221212222222222222222222222222222222222222222212122222222211222221222222222222222222222222222222222222222222222

>2Q66_A

KVFGITGPVSTVGATAAENKLNDSLIQELKKEGSFETEQETANRVQVLKILQELAQRFVYEVSKKKNMSDGMARDAGGKIFTygsYRlGVHGPGsdIdTLVVVPKHVTREDFFTVFDSLLRERKELDEIAPVPDAFVPIIKIKFSGISIALICARLDQPQVPLSLTLSDKNLLRNLDEKDLRALngTRvtDEILELVPKPNVFRIALRAIkLWAqRRAVyAnIFGFPggvAWAMLVARICQLYPNACSAVILNRFFIILSEWNWPQPVILKPIEDGPLQVRVWNPKIYAQDRSHRMPVItPAYPSmcaThNITESTKKVILQEFVRGVQITNDIFSNKKSWANLFEKNDFFFRYKFYLEITAYTRGSDEQHLKWSGLVESKVRLLVMKLEVLAGIKIAHPFTKPFESSYCCPTEDDYEMIQDKYGSHKTETALNALKLVTDENKEEESIKDAPKAYLSTMYIGLDFNIENKKEKVDIHIPCTEFVNLCRSFNEDYGDHKVFNLALRFVKGYDLPDEVFDENEKRP

222222222222222222222222222222222222222222222222222222222222222222222222222222222211122122222211212222222222222222222222222222222222222222222222222222222222222222222222222222222222222211221122222222222222222222122212222121222221112222222222222222222222222222222222222222222222222222222222222222222221222221112122222222222222222222222222222222222222222222222222222222222222222222222222222222222222222222222222222222222222222222222222222222222222222222222222222222222222222222222222222222222222222222222222222222222222222222222

>1ESQ_A

MRGSHHHHHHGSMDAQSAAKCLTAVRRHSPLVHSITNNVVTNFTANGLLALGASPVMAYAKEEVADMAKIAGALVLNIGTLSKESVEAMIIAGKSANEHGVPVILDPVGAGATPFRTESARDIIREVRLAAIrGnAAeIAHTVGVTDWLIKGVDAGEGGGDIIRLAQQAAQKLNTVIAItgeVdVIADTSHVYTLHnghkLltKVTGagSLlTSVVGAFCAVEENPLFAAIAAiSSygVAAQLAAQQTADKGPGSFQIELLNKLSTVTEQDVQEWATIERVTVS

22222222222222222222222222222222222222222222222222222222222222222222222222222222222222222222222222222222222222222222222222222222222212122122222222222222222222222222222222222222222111212222222222221111211222211221222222222222222222222122112222222222222222222222222222222222222222222222

>1MO8_A

QNPMTVAHMWFDNQIHEADTTENQSGVSFDKTSATWFALSRIAGLCNrAVFQANQENLPILKRAVAGdasESALLKCIEVCCGSVMEMREKYTKIVEIPfNsTNkyqLSIHKNPNASEPKHLLVMkgaPERILDRCSSILLHGKEQPLDEELKDAFQNAYLELGGLGeRVlGFCHLLLPDEQFPEGFQFDTDEVNFPVDNLCFVGLISMIDPP

222222222222222222222222222222222222222222222221222222222222222222211122222222222222222222222222222121221112222222222222222221112222222222222222222222222222222222222221221222222222222222222222222222222222222222222

>1VC9_B

MeLGAGGVVFNAKREVLLLRdRmGfWvFPkghPEPGESLEEAAVREVWEQTGVRAEVLLPLYPTRyvnpkGVErEVHWFLMRGEGAPRLEEGMTGAGWFSPEEARALLAfpedLGLLEVALERLPL

212222222222222222221212121221112222222222222222222222222222222221111122212222222222222222222222222222222222211112222222222222

>1PJ4_D

IKEKGKPLMLNPRTNKGMAFTLQERQMLGLQGLLPPKIETQDIQALRFHRNLKKMTSPLEKYIYIMGIQERNEKLFYRILQDDIESLMPIVYTPTVGLACSQYGHIFRRPKGLFISISDRGHVRSIVDNWPENhVkAVVVTDGERILGLGDLGVYGMGIPVGKLCLYTACAgirPDrCLPVCIDVGTDNIALLKDPFYMGLYQKRDRTQQYDDLIDEFMKAITDRYGRNTLIQFEDFGNHNAFRFLRKYREKYCTFNDDIQGTAAVALAGLLAAQKVISKPISEHKILFLGAGEAALGIANLIVMSMVENGLSEQEAQKKIWMFDKYGLLVKGRKAKIDSYQEPFTHSAPESIPDTFEDAVNILKPSTIIGVAGAGRLFTPDVIRAMASINERPVIFALSNPTAQAECTAEEAYTLTEGRCLFASGSPFGPVKLTDGRVFTPGQGNNVYIFPGVAlAVilCnTRHISDSVFLEAAKALTSQLTDEELAQGRLYPPLANIQEVSINIAIKVTEYLYANKMAFrYPEPEDKAKyVKErTwRSEYDSLLPDVYEWPESASSPPVITE

222222222222222222222222222222222222222222222222222222222222222222222222222222222222222222222222222222222222222222222222222222222222212122222222222222222222222222222222222111221222222222222222222222222222222222222222222222222222222222222222222222222222222222222222222222222222222222222222222222222222222222222222222222222222222222222222222222222222222222222222222222222222222222222222222222222222222222222222222222222222222222222222222222222222222222222221221121222222222222222222222222222222222222222222222222222222222221222222222122212122222222222222222222222222

>2CJA_B

MGSSHHHHHHSSGLVPRGSHMKLQFNLKAYFKTSADPTPAKDAIAALFEEANSTLLTRGAPEGQGAKVTEWKLGEDRIELTLQSGRYVRVHDAIFRLRKQLAEALGKKYKIGIRGIEVESFIIKVPADHELRMLKVPYIKSMENIEGGIQLELEVGEAEMKNRVPDRILTLLEEKIEAAQYGAKAEHWNLLWQREPMEHPFKEDPTQAMMKEGWLKRGSSRGQWIHGPQSARIFRTFEKIVLEELLEPLGYREMIFPKLVTWEVWMKSGHAKGVYPEIYYVCPPQTRDPDYWEEVADYYKVTHEVPTKLIKEKIAEPIGGMCyAQCPPFWMYVAGETLPNEEIPVKVFDRSGTSHrYeSGGIHGiERVDEfHrIEIVWIGTKEEVLKCAEELHDRYMHIFNDILDIEWRKARVTPWFMAQEGLLGLAEENTVGTTdYEACLPYRGPDGEWlefqnVSINGDKYPKGFNVKLQSGDELWSGCSgVgLeRWAAVFLAQKGLDPANWPEEFRNRVGEMPKGIRFL

222222222222222222222222222222222222222222222222222222222222222222222222222222222222222222222222222222222222222222222222222222222222222222222222222222222222222222222222222222222222222222222222222222222222222222222222222222222222222222222222222222222222222222222222222222222222222222222222222222222222222222222222222222222212222222222222222222222222222222212122222212222212122222222222222222222222222222222222222222222222222222222222222122222222222222111112222222222222222222222222221212122222222222222222222222222222222222

>2IVP_A

MLALGIEGtahtLGIGIVSEDKVLANVFDTLTTEKGGIHPKEAAEHHARLMKPLLRKALSEAGVSLDDIDVIAFSQGPGLGPALRVVATAARALAVKYRKPIVGVNhCIAhVEITKMFGVKDPVGLyVsggnTQVLALEGGRYRVFGETLDIGIgNAidVFARELGLGfpggpKVeKLAEKGEKYIELPYAVKGMDLSFSGLLTEAIRKYRSGKYRVEDLAYSFqETAFAALVEVTERAVAHTEKDEVVLVggvAanNRLREMLRIMTEDRGIKFFVPPyDLCrdNGAMIAYTGLRMYKAGISFRLEETIVKQKFRTDEVEIVWHHHHHH

222222221111222222222222222222222222222222222222222222222222222222222222222222222222222222222222222222222212221222222222222222121111222222222222222222222212211222222222111112212222222222222222222222222222222222222222222222221222222222222222222222222221112112222222222222222222222122211222222222222222222222222222222222222222222222

>1Z7E_A

MKTVVFAYHDMGCLGIEALLAAGYEISAIFTHTDNPGEKAFYGSVARLAAERGIPVYAPDNVNHPLWVERIAQLSPDVIFSFYYRHLIYDEILQLAPAGAFNLHGSLLPKYRGRAPLNWVLVNGETETGVTLHRMVKRADAGAIVAQLRIAIAPDDIAITLHHKLCHAARQLLEQTLPAIKHGNILEIAQRENEATCFGRRTPDDSFLEWHKPASVLHNMVRAVADPWPGAFSYVGNQKFTVWSSRVHPHASKAQPGSVISVAPLLIACGDGALEIVTGQAGDGITMQGSQLAQTLGLVQGSRLNSQPACTARRRTRVLILgVngfigNHLTERLLREDHYEVYGldigSDAISRFLNHPHFHFVEgdisIHSEWIEYHVKKCDVVLPlvaiatPIEYTRNPLRVFElDFEENLRIIRYCVKYRKRIIFPSTSEVYGMCSDKYFDEDHSNLIVGPVNKPRWIySVSKQLLDRVIWAYGEKEGLQFTLFRpFNwMGPRldNLNAARIGSSrAITQLILNLVEGSPIKLIDGGKQKRCFTDIRDGIEALYRIIENAGNRCDGEIINIGNPENEASIEELGEMLLASFEKHPLRHHFPPFAGFRVVESSSYYGKGYQDVEHRKPSIRNAHRCLDWEPKIDMQETIDETLDFFLRTVDLTDKPS

222222222222222222222222222222222222222222222222222222222222222222222222222222222222222222222222222222222222222222222222222222222222222222222222222222222222222222222222222222222222222222222222222222222222222222222222222222222222222222222222222222222222222222222222222222222222222222222222222222222222222222222222222222222121111122222222222222222111122222222222222222111122222222222222222211111122222222222221222222222222222222222222222222222222222222222222222222122222222222222222222222222122122221122222222221222222222222222222222222222222222222222222222222222222222222222222222222222222222222222222222222222222222222222222222222222222222222222222222222222222

>1NGE_A

MSKGPAVGIdLgttySCVGVFQHGKVEIIANDQGNRtTPSYVAFTDTERLIGDAAKNQVAMNPTNTVFDAkRLIGRRFDDAVVQSDMKHWPFMVVNDAGRPKVQVEYKGETKSFYPEEVSSMVLTKMKEIAEAYLGKTVTNAVVTVPAYFNDSQRQATKDAGTIAGLNVLRIINEPTAAAIAYGLDKKVGAERNVLIFSLgggtFDVSILTIEDGIFEVKSTAGDTHLGgeDFdNRMVNHFIAEFKRKHKKDISENKRAVRRLRTACeRAkrTLsSSTQASIEIDSLYEGIDFYTSITRARFEELNADLFRGTLDPVEKALRDAKLDKSQIHDIVLVggsTriPKIQKLLQDFFNGKELNKSINPdEAVAYGAAVQAAILSGDKSE

22222222212111122222222222222222222212222222222222222222222222222222221222222222222222222222222222222222222222222222222222222222222222222222222222222222222222222222222222222222222222222222222222222222111122222222222222222222222221122122222222222222222222222222222222212211221222222222222222222222222222222222222222222222222222222222222221112112222222222222222222222122222222222222222222

>1YUN_B

MGSSHHHHHHSSGLVPRGSHMGKRIGLfggtfdPVhIghMRsAVEMAEQFALDELRLLPNARPPHRETPQVSAAQRLAMVERAVAGVERLTVDPRELQRDKPSYTIDTLESVRAELAADDQLFMligWDAFCGLPTWHRWEALLDHCHIVVlQrPDADSEPPESLRDLLAARSVADPQALKGPGGQITFVWqTplAVsatQIrALLGAGRSVRFLVPDAVLNYIEAHHLYRAPHLEHHHHHH

22222222222222222222222222211111122121122122222222222222222222222222222222222222222222222222222222222222222222222222222222221112222222222222222222222221212222222222222222222222222222222222222121122111221222222222222222222222222222222222222222

>1ZP9_A

MKDLKKIESYLDKLRIKEKDGEERKIYAEVLDGRTLKTLYKLSAKGYITAMGGVisTgKeaNvFYADGVFDGKPVAMaVkIyRIETSEFDKMDEYLYGDERFDMRRISPKEKVFIwTEKEFRNLERAKEAGVSVpQPYTYMKNVLLMEfiGEDELpAPtLVeLGRELKELDVEGIFNDVVENVKRLYQEAELVHAdLSEynIMYIDKVYFidMGqAVTLRHPMAESYLERDVRNIIRFFSKYGVKADFEEMLKEVKGE

222222222222222222222222222222222222222222222222222222112121121222222222222221212122222222222222222222222222222222212222222222222222221222222222222211222221221221222222222222222222222222222222222122211222222222112212222222222222222222222222222222222222222222

>1EE1_A

SMQEKIMRELHVKPSIDPKQEIEDRVNFLKQYVKKTGAKGFVlgisGgqdsTLAGRLAQLAVESIREEGGDAQFIAvrlPHGTqQDEDDAQLALKFIKPDKSWKFDIKSTVSAFSDQYQQETGDQLTDFNKGNVKARTrMIAqYAIGGQEGLLVLGtDhAAeAVTGFFTKYGdGGADLLPLTGLTkRQGRTLLKELGAPERLYLKEptadlLDEKPQQSdETELGISyDEIDDYLEGKEVSAKVSEALEKRYSMTEHKRQVPASMFDDWWK

2222222222222222222222222222222222222222221111211112222222222222222222222222111222212222222222222222222222222222222222222222222222222222221222122222222222221212212222222222122222222222212222222222222222222211111222222221222222212222222222222222222222222222222222222222222

>2E89_A

MNPESRVIRKVLALQNDEKIFSGERRVLIafsGGVdsVVLTDVLLKLKNYFSLKEVALahfNHMLRESAERDEEFCKEFAKERNMKIFVGKEDVRAFAKENRMSLeEAGrFLrYKFlKEILESEGFDCIAtahHLNdLLEtSLLFFTRGTGLDGLIGfLPKEEVIRRPLYYVKRSEIEEYAKFKGLRWVEDETNYEVSIPrNRIRHRVIPELKRINENLEDTFLKMVKVLRAEREFLEEEAQKLYKEVKKGNCLDVKKLKEKPLALQRRVIRKFIGEKDYEKVELVRSLLEKGGEVNLGKGKVLKRKERWLCFSPEV

22222222222222222222222222222111222112222222222222222222221112222222222222222222222222222222222222222222212221221222122222222222221112221222122222222222222221222222222222222222222222222222222222222222122222222222222222222222222222222222222222222222222222222222222222222222222222222222222222222222222222222222222222222

>2OH5_A

ADVAGTSNRDFRGREQRLFNSEQyNYNnSLNGEVSVWVYAYYSDGSVLVINKNSQYKVGISETFKALKEYRKGQHNDSYDEYEVNQSIYYPNGGDARKFHSNAKPRAIQIIFSPSVNVRTIKMAKGNAVSVPDEYLQRSHPWEATGIKYRkIkRDGeiVGySHYFELPHEYNSISLAVSGVHkNPSSYNVGSAHNVMDVFQSCDLALRFCNRYWAELELVNHYISPNAYPYLDINNHSYGVALSNRQ

2222222222222222222222212221222222222222222222222222222222222222222222222222222222222222222222222222222222222222222222222222222222222222222222222222221212221122122222222222222222222212222222222222222222222222222222222222222222222222222222222222222

>1Q97_A

DYRPGGYHPAFKGEPYKDARYILVRKlgwghfsTvWLAKDMVNNTHVaMkIVRGDKVYTEAAEDEIKLLQRVNDADNTKEDSMGANHIlKLLDHFNHKGPNGVHVVMVfEvlgenLLALIKKYEHRGIPLIYVKQISKQLLLGLDYMHRRCGIIHTdIkPenVlMEIVDSPENLIQIKIADLGNACWYDEHYTNSIQTREYRSPEVLLGAPWGCGADIWSTACLIFELITGDFLFEPDEGHSYTKDDDHIAQIIELLGELPSYLLRNGKYTRTFFNSRGLLRNISKLKFWPLEDVLTEKYKFSKDEAKEISDFLSPMLQLDPRKRADAGGLVNHPWLKDTLGMEEIRVPDRELYGSGSDIPGWFEEVRDHKRH

2222222222222222222222222211111112122222222222212122222222222222222222222222222222222222122222222222222222221211111222222222222222222222222222222222222222221212112122222222222222222222222222222222222222222222222222222222222222222222222222222222222222222222222222222222222222222222222222222222222222222222222222222222222222222222222222222222222222222222222222222222222222222

>2E5Y_B

MKTIHVSVVTPdGPVYEDDVEMVSVKAKSGELGILPGHIPLVAPLEISAARLKKGGKTQYIAVsGGFLEVRPDKVTILAqaaeRAEdidVLraKAaKErAERRLQSQQDDIDFKRAELaLKraMNrLSVAEMK

2222222222212222222222222222222222222222222222222222222222222221222222222222222111122211122112212212222222222222222222122112212222222

>1KP2_A

TTILKHLPVGQRIGIafsGGLDtSAALLWMRQKGAVPYAytaNlGqPDEEDyDAiPRRAMEYGAENARLIDCRKQLVAEGIAAIQCGAFHNTTGGLTYFNTTPLGrAVtGTMlVAAMKEDGVNIWGdgstYKgndiERfYRYGLLTNAELQIYKPWLDTDFIDELGGRHEMSEFMIACGFDYKMSVEKAYstdSNMLGATHeAKDLEYLNSSVKIVNPIMGVKFWDESVKIPAEEVTVRFEQGHPVALNGKTFSDDVEMMLEANRIGGRHGLGMSDQIENRIIEAKSRGIYEAPGMALLHIAYERLLTGIHNEDTIEQYHAHGRQLGRLLYQGRWFDSQALMLRDSLQRWVASQITGEVTLELRRGNDYSILNTVSENLTYKPERLTMEKGDSVFSPDDRIGQLTMRNLDITDTREKLFGYAKTGLLSSSAASGVPQVENLENKGQSVEHHHHHH

22222222222222211122221222222222222222211121212222212212222222222222222222222222222222222222222222222222212212221222222222222211112211112212222222222222222222222222222222222222222222222222221112222222212222222222222222222222222222222222222222222222222222222222222222222222222222222222222222222222222222222222222222222222222222222222222222222222222222222222222222222222222222222222222222222222222222222222222222222222222222222222222222222222222222222222222

>1M83_A

MKTIFsgiqPsGVItIgnYIgaLRQfVELQHEYNCYFCIVDQHAITVWQDPHELRQNIRRLAALYLAVGIDPTQATLFIQSEVPAHAQAAWMLQCIVYIGELERMTQFKEkSAGKEAVSAGLLTyPPLMAADILLYNTDIVpvgEdqKQHIELTRDLAERFNKRYGELFTIPEARIPKVgariMSLVDPTKkmsksDPNPKAYITLLDDAKTIEKKIKSAVTDSEGTIRYDKEAKPGISNLLNIYSTLSGQSIEELERQYEGKGYGVFKADLAQVVIETLRPIQERYHHWMESEELDRVLDEGAEKANRVASEMVRKMEQAMGLGRRR

2222211112122212112211222122222222222222222222222222222222222222222222222222222222222222222222222222222222222212222222222222122222222222222221112112222222222222222222222222222222211112222222211111222222222222222222222222222222222222222222222222222222222222222222222222222222222222222222222222222222222222222222222222222222222222

>1E8X_A

AASEETLAFQRQLNALIGYDVTDVSNVHDDELEFTRRRLVTPRMAEVAGRDPKLYAMHPWVTSKPLPEYLLKKITNNCVFIVIHRSTTSQTIKVSADDTPGTILQSFFTKMAKKKSLMDIPESQNERDFVLRVCGRDEYLVGETPIKNFQWVRQCLKNGEEIHLVLDTPPDPALDEVRKEEWPLVDDCTGVTGYHEQLTIHGKDHESVFTVSLWDCDRKFRVKIRGIDIPVLPRTADLTVFVEANIQYGQQVLCQRRTSPKPFTEEVLWNVWLEFSIKIKDLPKGALLNLQIYCGKAPALSGKTSAEMPSPESKGKAQLLYYVNLLLIDHRFLLRHGEYVLHMWQLSGKGEDQGSFNADKLTSATNPDKENSMSISILLDNYCHPIALPKHRPTPDPEGDRVRAEMPNQLRKQLEAIIATDPLNPLTAEDKELLWHFRYESLKDPKAYPKLFSSVKWGQQEIVAKTYQLLAKREVWDQSALDVGLTMQLLDCNFSDENVRAIAVQKLESLEDDDVLHYLLQLVQAVKFEPYHDSALARFLLKRGLRNKRIGHFLFWFLRSEIAQSRHYQQRFAVILEAYLRGCGTAMLHDFTQQVQVIDMLQKVTIDIKSLSAEKYDVSSQVISQLKQKLENLQNLNLPQSFRVPYDPGLKAGALVIEKCKVmAskkKpLwLEFKCADPTALSNETIGIiFkHGdDLRQDMLILQILRIMESIWETESLDLCLLPyGCISTGDKIGMieivKDaTtIAkIQQSTVGNTGAFKDEVLSHWLKEKCPIEEKFQAAVERFVYSCAGYCVATFVLGIGDRHNdnImISETGNLfHidFGHILGNYKSFLGINKERVPFVLTPDFLFVMGTSGKKTSLHFQKFQDVCVKAYLALRHHTNLLIILFSMMLMTGMPQLTSKEDIEYIRDALTVGKSEEDAKKYFLDQIEVCRDKGWTVQFNWFLHLVLGIKQGEKHSA

2222222222222222222222222222222222222222222222222222222222222222222222222222222222222222222222222222222222222222222222222222222222222222222222222222222222222222222222222222222222222222222222222222222222222222222222222222222222222222222222222222222222222222222222222222222222222222222222222222222222222222222222222222222222222222222222222222222222222222222222222222222222222222222222222222222222222222222222222222222222222222222222222222222222222222222222222222222222222222222222222222222222222222222222222222222222222222222222222222222222222222222222222222222222222222222222222222222222222222222222222222222222222222222222222222222222222222222222222222222222222212111212122222222222222222212122122222222222222222222222222222212222222222211112212122122222222222222222222222222222222222222222222222222222222222112122222221211222222222222222222222222222222222222222222222222222222222222222222222222222222222222222222222222222222222222222222222222222222222222222222

>2BEK_A

MLRAKVRRIALANQKggvgkttTAINLAAYLARLGKRVLLVDLaPqGnATSGLGVRAERGVYHLLQGEPLEGLVHPVDGFHLLPATPDLVGATVELAGAPTALREALRDEGYDLVLLDappSLSPLTLNALAAAEGVVVPVQAEYYALEGVAGLLATLEEVRAGLNPRLRLLGILVTmYDGRTLLAQQVEAQLRAHFGEKVFWTViprnvRlaEApSFGKTIAQHAPTSPGAHAyRRLAEEVMARVQEAGSHHHHHH

22222222222222211111112222222222222222222221212122222222222222222222222222222222222222222222222222222222222222222222221112222222222222222222222222222222222222222222222222222222212222222222222222222222222221111121122122222222222222222212222222222222222222222

>1R9T_B

MSDLANSEKYYDEDPYGFEDESAPITAEDSWAVISAFFREKGLVSQQLDSFNQFVDYTLQDIICEDSTLILEQLAQHTTESDNISRKYEISFGKIYVTKPMVNESDGVTHALYPQEARLRNLTYSSGLFVDVKKRTYEAIDVPGRELKYELIAEESEDDSESGKVFIGRLPIMLRSKNCYLSEATESDLYKLKECPFDMGGYFIINGSEKVLIAQERSAGNIVQVFKKAAPSPISHVAEIRSALEKGSRFISTLQVKLYGREGSSARTIKATLPYIKQDIPIVIIFRALGIIPDGEILEHICYDVNDWQMLEMLKPCVEDGFVIQDRETALDFIGRRGTALGIKKEKRIQYAKDILQKEFLPHITQLEGFESRKAFFLGYMINRLLLCALDRKDQDDRDHFGKKRLDLAGPLLAQLFKTLFKKLTKDIFRYMQRTVEEAHDFNMKLAINAKTITSGLKYALATGNWGEQKKAMSSRAGVSQVLNRYTYSSTLSHLRRTNTPIGRDGKLAKPRQLHNTHWGLVCPAETPEGQACGLVKNLSLMSCISVGTDPMPIITFLSEWGMEPLEDYVPHQSPDATRVFVNGVWHGVHRNPARLMETLRTLRRKGDINPEVSMIRDIREKELKIFTDAGRVYRPLFIVEDDESLGHKELKVRKGHIAKLMATEYQDIEGGFEDVEEYTWSSLLNEGLVEYIDAEEEESILIAMQPEDLEPAEANEENDLDVDPAKRIRVSHHATTFTHCEIHPSMILGVAASIIPFPDHNQSPrNTyQSAMGKQAMGVFLTNYNVRMDTMANILYYPQKPLGTTRAMEYLKFRELPAGQNAIVAIACYSGYNQEDSMIMNQSSIDRGLFRSLFFRSYMDQEKKYGMSITETFEKPQRTNTLRMKHGTYDKLDDDGLIAPGVRVSGEDVIIGKTTPISPDEEELGQRTAYHSKRDASTPLRSTENGIVDQVLVTTNQDGLKFVKVRVRTTKIPQIGDKFASRHGQkGTIGITYRREDMPFTAEGIVPDLIINPHAIPsrMTVAHLIECLLSKVAALSGNEGDASPFTDITVEGISKLLREHGYQSRGFEVMYNGHTGKKLMAQIFFGPTYYQRLRHMVDDKIHARARGPMQVLTRQPVEGRSRDGGLRFGEMERDCMIAHGAASFLKERLMEASDAFRVHICGICGLMTVIAKLNHNQFECKGCDNKIDIYQIHIPYAAKLLFQELMAMNITPRLYTDRSRDF

222222222222222222222222222222222222222222222222222222222222222222222222222222222222222222222222222222222222222222222222222222222222222222222222222222222222222222222222222222222222222222222222222222222222222222222222222222222222222222222222222222222222222222222222222222222222222222222222222222222222222222222222222222222222222222222222222222222222222222222222222222222222222222222222222222222222222222222222222222222222222222222222222222222222222222222222222222222222222222222222222222222222222222222222222222222222222222222222222222222222222222222222222222222222222222222222222222222222222222222222222222222222222222222222222222222222222222222222222222222222222222222222222222222222222222222222222222222222222222222222222222222222222222222222222222222222222222222122122222222222222222222222222222222222222222222222222222222222222222222222222222222222222222222222222222222222222222222222222222222222222222222222222222222222222222222222222222222222222222222222222222222222222222222222221222222222222222222222222222222211222222222222222222222222222222222222222222222222222222222222222222222222222222222222222222222222222222222222222222222222222222222222222222222222222222222222222222222222222222222222222222222222222222222222

>1NYR_B

MEQINIQFPDGNKKAFDKGTTTEDIAQSISPGLRKKAVAGKFNGQLVDLTKPLETDGSIEIVTPGSEEALEVLRHSTAHLMAHAIKRLYGNVKFGVGPVIEGGFYYDFDIDQNISSDDFEQIEKTMKQIVNENMKIERKVVSRDEAKELFSNDEYKLELIDAIPEDENVTLYSQGDFTDLCRGVHVPSTAKIKEFKLLSTAGAYWRGDSNNKMLQRIYGTAFFDKKELKAHLQMLEERKERDHRKIGKELELFTNSQLVGAGLPLWLPNGATIRREIERYIVDKEVSMGYDHVYTPVLANVDLYKTSGHWDHyQEdMFPPMQLDETESMVLRPmNCPHHMMIYANKPHSYRELPIRIAELGTmHrYeASGAVSGlqrvRGmTlNDSHIFVRPDQIKEEFKRVVNMIIDVYKDFGFEDYSFRLSYRDPEDKEKYFDDDDMWNKAENMLKEAADELGLSYEEAIGeAAFyGPkLdVQVKTAMGKEEtlstAqLDFLLPERFDLTYIGQDGEHHRPVVIHRGVVstMerFVAFLTEETKGAFPTWLAPKQVQIIPVNVDLHYDYARQLQDELKSQGVRVSIDDRNEKMGYKIREAQMQKIPYQIVVGDKEVENNQVNVRQYGSQDQETVEKDEFIWNLVDEIRLKKHR

222222222222222222222222222222222222222222222222222222222222222222222222222222222222222222222222222222222222222222222222222222222222222222222222222222222222222222222222222222222222222222222222222222222222222222222222222222222222222222222222222222222222222222222222222222222222222222222222222222222222222222222222122122222222222222222122222222222222222222222222221212122222221111221212222222222222222222222222222222222222222222222222222222222222222222222222222222212221221212222222222211112122222222222222222222222222222221121122222222222222222222222222222222222222222222222222222222222222222222222222222222222222222222222222222222222222222222222

>1R9T_A

MVGQQYSSAPLRTVKEVQFGLFSPEEVRAISVAKIRFPETMDETQTRAKIGGLNDPRLGSIDRNLKCQTCQEGMNECPGHFGHIDLAKPVFHVGFIAKIKKVCECVCMHCGKLLLDEHNELMRQALAIKDSKKRFAAIWTLCKTKMVCETDVPSEDDPTQLVSRGGCGNTQPTIRKDGLKLVGSWKKDRATGDADEPELRVLSTEEILNIFKHISVKDFTSLGFNEVFSRPEWMILTCLPVPPPPVRPSISFNESQRGEDDLTFKLADILKANISLETLEHNGAPHHAIEEAESLLQFHVATYMDNDIAGQPQALQKSGRPVKSIRARLKGKEGRIRGNLMGKRVDFSARTVISGDPNLELDQVGVPKSIAKTLTYPEVVTPYNIDRLTQLVRNGPNEHPGAKYVIRDSGDRIDLRYSKRAGDIQLQYGWKVERHIMDNDPVLFNRQPSLHKMSMMAHRVKVIPYSTFRLNLSVTSPYNAdFdGDEMNLHVPQSEETRAELSQLCAVPLQIVSPQSNKPCMGIVQDTLCGIRKLTLRDTFIELDQVLNMLYWVPDWDGVIPTPAIIKPKPLWSGKQILSVAIPNGIHLQRFDEGTTLLSPKDNGMLIIDGQIIFGVVEkKTVGSSNGGLIHVVTREKGPQVCAKLFGNIQKVVNFWLLHNGFSTGIGDTIADGPTMREITETIAEAKKKVLDVTKEAQANLLTAKHGMTLRESFEDNVVRFLNEARDKAGRLAEVNLKDLNNVKQMVMAGSkgSFINIAQMSACVGQQSVEGKRIAFGFVDRTLPHFSKDDYSPESKGFVENSYLRGLTPQEFFFHAMGGREGLIDTAVKTAETGYIQRRLVKALEDIMVHYDNTTRNSLGNVIQFIYGEDGMDAAHIEKQSLDTIGGSDAAFEKRYRVDLLNTDHTLDPSLLESGSEILGDLKLQVLLDEEYKQLVKDRKFLREVFVDGEANWPLPVNIRRIIQNAQQTFHIDHTKPSDLTIKDIVLGVKDLQENLLVLRGKNEIIQNAQRDAVTLFCCLLRSRLATRRVLQEYRLTKQAFDWVLSNIEAQFLRSVVHPGEMVGVLAAQSIGEPATQMTLNTFHFAGVASKKVTSGVPRLKEILNVAKNMKTPSLTVYLEPGHAADQEQAKLIRSAIEHTTLKSVTIASEIYYDPDPRSTVIPEDEEIIQLHFSLLDEEAEQSFDQQSPWLLRLELDRAAMNDKDLTMGQVGERIKQTFKNDLFVIWSEDNDEKLIIRCRVVRPKSLDAETEAEEDHMLKKIENTMLENITLRGVENIERVVMMKYDRKVPSPTGEYVKEPEWVLETDGVNLSEVMTVPGIDPTRIYTNSFIDIMEVLGIEAGRAALYKEVYNVIASDGSYVNYRHMALLVDVMTTQGGLTSVTRHGFNRSNTGALMRCSFEETVEILFEAGASAELDDCRGVSENVILGQMAPIGTGAFDVMIDEESLVKYMPEQKITEIEDGQDGGVTPYSNESGLVNADLDVKDELMFSPLVDSGSNDAMAGGFTAYGGADYGEATSPFGAYGEAPTSPGFGVSSPGFSPTSPTYSPTSPAYSPTSPSYSPTSPSYSPTSPSYSPTSPSYSPTSPSYSPTSPSYSPTSPSYSPTSPSYSPTSPSYSPTSPSYSPTSPSYSPTSPSYSPTSPSYSPTSPSYSPTSPAYSPTSPSYSPTSPSYSPTSPSYSPTSPSYSPTSPNYSPTSPSYSPTSPGYSPGSPAYSPKQDEQKHNENENSR

22222222222222222222222222222222222222222222222222222222222222222222222222222222222222222222222222222222222222222222222222222222222222222222222222222222222222222222222222222222222222222222222222222222222222222222222222222222222222222222222222222222222222222222222222222222222222222222222222222222222222222222222222222222222222222222222222222222222222222222222222222222222222222222222222222222222222222222222222222222222222222222222222222222222222222222222222222222222222222222222212122222222222222222222222222222222222222222222222222222222222222222222222222222222222222222222222222222222222222222222222222222222222222212222222222222222222222222222222222222222222222222222222222222222222222222222222222222222222222222222222222222222222222222222222222221122222222222222222222222222222222222222222222222222222222222222222222222222222222222222222222222222222222222222222222222222222222222222222222222222222222222222222222222222222222222222222222222222222222222222222222222222222222222222222222222222222222222222222222222222222222222222222222222222222222222222222222222222222222222222222222222222222222222222222222222222222222222222222222222222222222222222222222222222222222222222222222222222222222222222222222222222222222222222222222222222222222222222222222222222222222222222222222222222222222222222222222222222222222222222222222222222222222222222222222222222222222222222222222222222222222222222222222222222222222222222222222222222222222222222222222222222222222222222222222222222222222222222222222222222222222222222222222222222222222222222222222222222222222222222222222222222222222222222222222222222222222222222222222222222222222222222222222222222222222222222222222222222222222222222222222222222222222222222222222222222222222222222222222

>1DY3_A

TVAYIAIGSNLASPLEQVNAALKALGDIPESHILTVSSFYRTPPLGPQDQPDYLNAAVALETSLAPEELlNHTqRIELQQGrVrkAErwGPrTLdldiMLFGNEVINTerltvPhyDMKNrGFMLWPLFEIAPELVFPDGEMLRQILHTRAFDKLNKW

22222222222222222222222222222222222222222222222222222222222222222222212221222222212112211221221111222222222211111211222212222222222222222222222222222222222222

>1R4N_B

DWEGRWNHVKKFLErSGPFTHPDFEPSTESLQFLLDTCKVLVigagGLGCELLKNLALSGFRQIHVidmdTIDVsnLNrqFLFRPKDIGRPkAEVAAEFLNDRVPNCNVVPHFnkiqDFNDTFYRQFHIIVCgldsIIaRRWINGMLISLLNYEDGVLDPSSIVPLIDGGTEGFKGNARVILPGMTACIECTLELYPPQVNFPMATIASMPRLPEHCIEYVRMLQWPKEQPFGEGVPLDGDDPEHIQWIFQKSLERASQYNIRGVTYRLTQGVVkRiiPAVASTNAVIAAVCATEVFKIATSAYIPLNNYLVFNDVDGLYTYTFEAERKENCPACSQLPQNIQFSPSAKLQEVLDYLTNSASLQMKSPAITATLEGKNRTLYLQSVTSIEERTRPNLSKTLKELGLVDGQELAVADVTTPQTVLFKLHFTS

22222222222222122222222222222222222222222211112222222222222222222211112222112211222222222221222222222222222222222111122222222222222211112212222222222222222222222222222222222222222222222222222222222222222222222222222222222222222222222222222222222222222222222222222222222222221211222222222222222222222222222222222222222222222222222222222222222222222222222222222222222222222222222222222222222222222222222222222222222222222222222222222

>2HIX_A

MGSSHHHHHHSSGLVPRGSHMEFKVIAEYFDKLEKISSRLQLTALLADLLSKSDKTIIDKVVYIIQGKLWPDFLGYPELGIGEKFLIKAISIATNTDENSVENLYKTIGDLGEVARRLKSKQQSTGILGFLGTTSKESLTVDEVYSTLSKVALTTGEGSRDLKIRLLAGLLKKADPLEAKFLVRFVEGRLRVGIGDATVLDAMAIAFGGGQSASEIIERAYNLRADLGNIAKIIVEKGIEALKTLKPQVGIPIRPMlaERLSNPEEILKKMGGNAIVdykyDGErAQIHKKEDKIFIFSrRLENITSQYPDVVDYVSKYIEGKEFIIEGeIVAIDPESGEMRPFQELMHRKRKSDIYEAIKEYPVNVFLfDLMYYEDVDYTTKPLEARRKLLESIVKPNDYVKIaHHIQANNVEDLKSFFYRAISEGGEGVmVkAIGKDAIYQAGArGWLwIkLkRDYQSEMADTVDLVVVGGFYGKGKRGGKISSLLMAAYNPKTDSFESVCKVASGFSDEQLDELQKKLMEIKRDVKHPRVNSKMEPDIWVEPVYVAEIIGSEITISPLHTCCQDVVEKDAGLSIRFPRFIRWRDDKSPEDATTTDEILEMYNKQPKKKIESPAVDESV

222222222222222222222222222222222222222222222222222222222222222222222222222222222222222222222222222222222222222222222222222222222222222222222222222222222222222222222222222222222222222222222222222222222222222222222222222222222222222222222222222222222222222211222222222222222222211112221222222222222221222222222222222222222222222221222222222222222222222222222222222222222122222222222222222222222222222222221222222222222222222222222221212222222222221222121212222222222222222222222222222222222222222222222222222222222222222222222222222222222222222222222222222222222222222222222222222222222222222222222222222222222222222222222

>2NYJ_A

SVSAGEKPPRLYDRRSIFDAVaQSNCQELESLLPFLQRSKKRLTDSEFKDPETGkTCLLkAMlnLHNGQNDTIALLLDVARKTDSLKQFVNASyTDSYyKGqTALHiAIerRNMTLVTLLVENGADVQAAANGDfFKKTKGRPGFYFGELPLSLAACTNQLAIVKFLLQNSWQPADISARDSVGNTVLHALVEVADNTVDNTKFVTSMYNEILILGAKLHPTLKLEEITNRKGLTPLALAASSGKIGVLAYILQREIHEPECRHAAAHHHHHH

222222222222222222222122222222222222222222222222222222122221221122222222222222222222222222222122221221222212211222222222222222222222221222222222222222222222222222222222222222222222222222222222222222222222222222222222222222222222222222222222222222222222222222222222222222222

>1Q12_D

MASVQLQNVTKAwGEvVvSKDINLDIHEGEFVVFVGPsgcgkstLLRMIAGLETITSGDLFIGEKRMNDTPPAERGVGMVFQSYALYPHLSVAENMSFGLKLAGAKKEVINQRVNQVAEVLQLAHLLDRKPKALSGGQRQRVAIGRTLVAEPSVFLLDEPLSNLDAALRVQMRIEISRLHKRLGRTMIYVThDQVEAMTLADKIVVLDAGRVAQVGKPLELYHYPADRFVAGFIGSPKMNFLPVKVTATAIDQVQVELPMPNRQQVWLPVESRDVQVGANMSLGIRPEHLLPSDIADVILEGEVQVVEQLGNETQIHIQIPSIRQNLVYRQNDVVLVEEGATFAIGLPPERCHLFREDGTACRRLHKEPGVASASHHHHHH

222222222222122121222222222222222222211111112222222222222222222222222222222222222222222222222222222222222222222222222222222222222222222222222222222222222222222222222222222222222222222222222221222222222222222222222222222222222222222222222222222222222222222222222222222222222222222222222222222222222222222222222222222222222222222222222222222222222222222222222222222222222222222222222

>2BIY_A

MDGTAAEPRPGAGSLQHAQPPPQPRKKRPEDFKFGKIlgegsfsTvVLARELATSREYaIkILEKRHIIKENKVPYVTRERDVMSRLDHPFFvKLYFTFQDDEKLYFGlSyaKNgeLLKYIRKIGSFDETCTRFYTAEIVSALEYLHGKGIIHRdLkPeNIlLNEDMHIQItdFGTAKVLSPESKQARANAFVGTAQYVSPELLTEKSACKSSDLWALGCIIYQLVAGLPPFRAGNEYLIFQKIIKLEYDFPEKFFPKARDLVEKLLVLDATKRLGCEEMEGYGPLKAHPFFESVTWENLHQQTPPKLTA

2222222222222222222222222222222222222111111121222222222222121222222222222222222222222222222212222222222222221211221122222222222222222222222222222222222222121212212222222221122222222222222222222222222222222222222222222222222222222222222222222222222222222222222222222222222222222222222222222222222222222222222222

>2IDX_A

MPKIYTKTGDKGFSSTFTGERRPKDDQVFEAVGTTDELSSAIGFALELVTEKGHTFAEELQKIQCTLQDVGSALATPCSSAREAHLKYTTFKAGPILELEQWIDKYTSQLPPLTAFILPSGGKISSALHFCRaVCRrAErrVVPLVQMGETDANVAKFLNrlSDyLFTLARYAAMKEGNQEKIYMKNDPSAESEGL

2222222222222222222222222222222222222222222222222222222222222222222222222222222222222222222222222222222222222222222222222222222222221222122112222222222222222222112212222222222222222222222222222222

>1J7K_A

MSEFLTPERTVYDSGVQFlrpKSLDefiGQENVKKKLSLALEAAKMRGEVLDHVLLAGPpglgkttLAHIIASELQTNIHVTSGPVLVKQGDMAAILTSLERGDVLFIdEIHRLNKAVEELLYSAIEDFQIDIMIGKGPSAKSIRIDIQPFTLVGATTRSGLLSSPLRSRFGIILELDFyTVKELKEiIKraASlMDVEIEDAAAEMIAKRSRGTgrIAirLTKRVRDMLTVVKADRINTDIVLKTMEVLNIDDEGLDEFDRKILKTIIEIYRGGPVGLNALAASLGVEADTLSEVYEPYLLQAGFLARTPRGRIVTEKAYKHLKYEVPENRLF

2222222222222222221112222111222222222222222222222222222222211111112222222222222222222222222222222222222222221222222222222222222222222222222222222222222222222222222222222222222222212222222122112212222222222222222222211221122222222222222222222222222222222222222222222222222222222222222222222222222222222222222222222222222222222222222222

>2A5Y_C

MLCEIECRALSTAHTRLIHDFEPRDALTYLEGKNIFTEDHSELISKMSTRLERIANFLRIYRRQASELGPLIDFFNYNNQSHLADFLEDYIDFAINEPDLLRPVVIAPQFSRQMLDRKLLLGNVPKQmTCyIrEYHVDRVIKKLDEMCDLDSFFLFLHGRAgsgksviASqALSKSDQLIGINYDSIVWLKDSGTAPKSTFDLFTDILLMLKSEDDLLNFPSVEHVTSVVLKRMICNALIDRPNTLFVFDdVVQEETIRWAQELRLRCLVTTrDVEISNAASQTCEFIEVTSLEIDECYDfLEAyGMPMPVGEKEEDVLNKTIELSSGNpaTLmMFFKSCEPKTFEKMAQLNNKLESRGLVGVECItpySyKSLAMALQRCVEVLSDEDRSALAFAVVMPPGVDIPVKLWSCVIPVDICSNEEEQLDDEVADRLKRLSKRGALLSGKRMPVLTFKIDHIIHMFLKHVVDAQTIANGISILEQRLLEIGNNNVSVPERHIPSHFQKFRRSSASEMYPKTTEETVIRPEDFPKFMQLHQKFYDSLKNFACC

222222222222222222222222222222222222222222222222222222222222222222222222222222222222222222222222222222222222222222222222222222212212122222222222222222222222222221111111221222222222222222222222222222222222222222222222222222222222222222222222222222222212222222222222222222221222222222222222222222222222122212222222222222222222222221122122222222222222222222222222222222111212222222222222222222222222222222222222222222222222222222222222222222222222222222222222222222222222222222222222222222222222222222222222222222222222222222222222222222222222222222222

>1G5T_A

MSDERYQQRQQKVKDRVDARVAQAQEERGIIIVFTGngkgkttAAFGTAARAVGHGKNVGVVQFIKGTwPNGeRNLLEPHGVEFQVMATGFTWETQNREADTAACMAVWQHGKRMLADPLLDMVVLDeLTyMVAYDYLPLEEVISALNARPGHQTVIITGrGCHRDILDLADTVSELRpVKhAFDAGVKAQMGIDY

2222222222222222222222222222222222221111111222222222222222222222222212221222222222222222222222222222222222222222222222222222222122122222222222222222222222222222122222222222222222122122222222222222

>1UF9_C

MGHEAKHPIIIGITGnigsgkstVAALLRSWGYPVLDLDALAARARENKEEELKRLFPEAVVGGRLDRRALARLVFSDPERLKALEAVVHPEVRRLLMEELSRLEAPLVFLEIPLLFEKGWEGRLHGTLLVAAPLEERVRrvMArSGLSREEVLArERAQMPEEEKRKRATWVLEntgsLEdLERALKAVLAELTGGATEGRG

22222222222222211111111222222222222222222222222222222222222222222222222222222222222222222222222222222222222222222222222222222222222222222222112212222222222122222222222222222221111221222222222222222222222

>1KVK_A

MLSEVLLVSAPGkVILHGEHAVVHGKVALAVALNLRtFLVLRPQSNGKVSLNlPnvGIKQVWDVATLQLLDTGFLEQGDVPAPTLEQLEKLKKVAGLPRDCVGneGLsLLAFLYLYLAICRKQRTLPSLDIMvWsElPpgaglgssAAySVCVAAALLTACEEVTNPLKDRGSIGSWPEEDLKSINKWAYEGERVihGNPSGVdNSVSTWGGALRYQQGKMSSLKRLPALQILLTNTKVPRSTKALVAGVRSRLIKFPEIMAPLLTSIDAISLECERVLGEMAAAPVPEQYLVLEELMDMNQHHLNALGVGHASLDQLCQVTAAHGLHSKLTGAGGGGCGITLLKPGLERAKVEAAKQALTGCGFDCWETSIGAPGVSMHSATSIEDPVRQALGL

22222222222212222222222222222222222212222222222222221211222222222222222222222222222222222222222222222221122122222222222222222222222212121211111111221222222222222222222222222222222222222222222222211222222122222222222222222222222222222222222222222222222222222222222222222222222222222222222222222222222222222222222222222222222222222222222222222222222222222222222222222222222222222222222222222222222

>2YWW_B

MIPMEELkVKkiTNGTvIdhIDAGKALMVFKVLNVPKETSVMIAINVPSKkKGKKDiLkIEGIELKKEDVDKISLISPDVtInIiRNGKvVEkLKPQIPDEIEGTLKCTNPNCITNKEKVRGKFKIESKNPLKIRCYYCEKFLNEVIFE

22222221221122221211222222222222222222222222222222122222121222222222222222222222121212222122122222222222222222222222222222222222222222222222222222222

>1TQP_A

MNIAELYGKMGKHSWRIMDAIFKNLWDYEYVPLQLISSHARIGEEKARNILKYLSDLRVVQNRQKDYEGSTFTFIGLSLYSLHRLVRSGKVDAIGKLMgEgKesAvFNCYSEKFGECvVkFHKVGHTSFKKVKEKRDYGDLHFSVLAIRSARNEFRALQKLQGLAVpKVYAWEGNAVLMeliDaKeLYRVRVENPDEVLDMILEEVAKFYHRGIVHGdLSQynVlVSEEGIWIidFpQSVEVGEEGWREILERDVRNIITYFSRTYRTEKDINSAIDRILQE

222222222222222222222222222222222222222222222222222222222222222222222222222222222222222222222222221212112122222222222121222222222222222222222222222222222222222222222212222222222221112121222222222222222222222222222222212221121222222221121222222222222222222222222222222222222222222222

>1B8A_B

MYRTHYSSEITEELNGQKVKVAGWVWEVKDLGGIKFLWIRDRDGIVQITAPKKKVDPELFKLIPKLRSEDVVAVEGVVNFTPKAKLGFEILPEKIVVLNRAETPLPLDPTGKVKAELDTRLNNRFMDLRRPEVMAIFKIRSSVFKAVRDFFHENGFIEIHTPKIIATATEGGTELFPMKYFEEDAFLAESPQLYKEIMMASGLDRVYEIAPiFrAeEHNTTrhlNEaWsIDSEMAFIEDEEEVMSFLERLVAHAINYVREHNAKELDILNFELEEPKLPFPRVSYDKALEILGDLGKEIPWGEDIDTEGeRLLGKYMMENENAPLYFLYQYPSEAKPFYIMKYDNKPEICRAFDLEYRGVeissGGQREHRHDILVEQIKEKGLNPESFEFYLKAFRYGMPPHGGFgLgAerLIKQMLDLPNiREVILFPRDRRRLTP

222222222222222222222222222222222222222222222222222222222222222222222222222222222222222222222222222222222222222222222222222222222222222222222222222222222222222222222222222222222222222222222222222222222222222222212121222221112212122222222222222222222222222222222222222222222222222222222222222222222222222222222122222222222222222222222222222222222222222222222222111122222222222222222222222222222222222222222212121122222222221222222222222222

>2Z02_B

MEIKLEEILKKQPLySgkakSiYEIDDDKvLiEFRDDITAGNGAKHDVKQGKGYLNALISSKLFEALEENGVKThYIKYIEPRYMIakkvEiIPIeVIVRNIAAGSLCRRYPFEEGKELPFPIVQFDYkNDEYGDPMLNEDIAVALGLATREELNKIKEIALKVNEVLKKLFDEKGIILVDFkIeIGKDREGNLLVadeISPDTMRLWDKETRDVLDKDVFRKDLGDVIAKYRIVAERLGLL

22222222222222121111212222222121222222222222222222222222222222222222222222122222222222111121222122222222222222222222222222222222122222222222222222222222222222222222222222222222222222121222222222221112222222222222222222222222222222222222222222

>1E2Q_A

GSHMAARRGALIVLEGVdragkstqSrKLVEALCAAGHRAELLRFPERSTEIGKLLSSYLQKKSDVEDHSVHLLFSANRWEQVPLIKEKLSQGVTLVVDrYAFSGVAFTGAKENFSLDWCKQPDVGLPKPDLVLFLQLQLADAAKrGAFGHERYeNGAFQERALRCFHQLMKDTTLNWKMVDasksiEAvHEDIRVLSEDAIATATEKPLGELWK

22222222222222222111111112122222222222222222222222222222222222222222222222222222222222222222222222212222222222222222222222222222222222222222222221222222221222222222222222222222222222111112212222222222222222222222222

>1Z0S_A

MGSSHHHHHHDYDIPTTENLYFQGGGGGGMRAAVVYKTDGHVKRIEEALKRLEVEVELFNQPSEELENFDFIVSVGGDgTIlrILQKLKRCPPIFGINTGrVGLLTHASPENFEVELKKAVEKFEVERFPRVSCSAMPDVLALneIAVLSRKPAKMIDVALRVDGVEVDRIRCDGFIVATQIGSTgyAFsAGGPVVEPYLECFILIPIAPFRFGWKPYVVSMERKIEVIAEKAIVVAdgqKSVDFDGEITIEKSEFPAVFFKNEKRFRNLFGKVRSIG

22222222222222222222222222222222222222222222222222222222222222222222222222222212211222222222222222221222222222222222222222222222222222222222222112222222222222222222222222222222222222222112212222222222222222222222222222222222222222222222211122222222222222222222222222222222222222

>2IYW_A

MAPKAVLVGLpgsgkstiGrRLAKALGVGLLDTDVAIEQRTGRSIADIFATDGEQEFRRIEEDVVRAALADHDGVLSLGGGAVTSPGVRAALAGHTVVYLEISAAEGVrrtGGNTVRPLLAGPDRAEKYRALMAKRAPLYRRVATMRVDtnRrnpGAvVRHILSRLQVPSPSEAATLEHHHHHH

2222222222111111112122222222222222222222222222222222222222222222222222222222222222222222222222222222222222221112222222222222222222222222222222222222211211122122222222222222222222222222

>1H3E_A

MAGTGHTPEEALALLKRGAEEIVPEEELLAKLKEGRPLTVKlgadPtRPDLhLghAvvLRKMRQFQELGHKVVLIIGDFTGMIGDPSGRSKTrPPLTLEETRENAKTyVAQAGKILRQEPHLFELRYNSEWLEGLTFKEVVRLTSLMTVAQMLEREDFKKRYEAGIPISLHELLYPFAQAYDSVAIRADVEmggTdqRFNLLVGREVQRAYGQSPQVCFLMpllVGLDGREkmsksLDNYIGLTEPPEAMFKKLMRVPDPLLPSYFRLLTDLEEEEIEALLKAGPVPAHRVLARLLTAAYALPQIPPRIDRAFYESLGYAWEAFGRDKEAGPEEVRRAEARYDEVAKGGIPEEIPEVTIPASELKEGRIWVARLFTLAGLTPSNAEARRLIQNRGLRLDGEVLTDPMLQVDLSRPRILQRGKDRFVRVRLSD

222222222222222222222222222222222222222221111212222121121122222222222222222222222222222222221222222222222221222222222222222222222222222222222222222222222222222222222222222222222222222222222221112112222222222222222222222221112222222111112222222222222222222222222222222222222222222222222222222222222222222222222222222222222222222222222222222222222222222222222222222222222222222222222222222222222222222222222222222222222222222222222222

>1J1Z_A

MKIVLaysGgLdtSIILKWLKETYRAEVIAftaDiGqGEEVEEAREKALRTGASKAIALDLKEEFVRDFVFPMMRAGAVYEGYYLLGTSIArPLiAKHLVRIAEEEGAEAIAhgaTGKGNdQVRfELTAYALKPDIKVIAPWREWSFQGrKEMIAYAEAHGIPVPVTQEKPYsmdANLLHISYEGGVLEDPWAEPPKGMFRMTQDPEEAPDAPEYVEVEFFEGDPVAVNGERLSPAALLQRLNEIGGRHGVGRVDIVENRFVGMKSRGVYETPGGTILYHARRAVESLTLDREVLHQRDMLSPKYAELVYYGFWYAPEREALQAYFDHVARSVTGVARLKLYKGNVYVVGRKAPKSLYRQDLVSFDEAGGYDQKDAEGFIKIQALRLRVRALVEREGHGA

2222211121211222222222222222221112121222222222222222222222222222222222222222222222222222222122122222222222222222111222221222122222222222222222222222212222222222222222222222111222222222222222222222222222222222222222222222222222222222222222222222222222222222222222222222222222222222222222222222222222222222222222222222222222222222222222222222222222222222222222222222222222222222222222222222222222222222

>1BCP_A

DDPPATVYRYDSRPPEDVFQNGFTAWGNNDNVLEHLTGRSCQVGSSNSAFVSTSSSRRYTEVYLEHRMQEAVEAERAGRGTGHFIGYIYEVRADNNFYGAASSYFEYVDTYGDNAGRILAGALATYQSEYLAHRRIPPENIRRVTRVYHNGITGETTTTEYSNARYVSQQTRANPNPYTSRRSVASIVGTLVRMAPVVGACMARQAESSEAMAAWSERAGEAMVLVYYESIAYsf

2222222222222222222222222222222222222222222222222222222222222222222222222222222222222222222222222222222222222222222222222222222222222222222222222222222222222222222222222222222222222222222222222222222222222222222222222222222222222222211

>1GN8_B

MQKRAIypgtfdPItNghIDiVTRATQMFDHVILAIAASPSkKPMFTLEERVALAQQATAHLGNVEVVGFSDLMANFARNQHATVLIrgLrAVAdFEYeMQLAHMNRHLMPELESVFLMpSKEwSFisssLVkEVARHQGDVTHFLPENVHQALMAKLA

222222111111221211221222222222222222222221222222222222222222222222222222222222222222222112122212221222222222222222222221222122111122122222222222222222222222222

>2QRD_G

AMDVQETQKGALKEIQAFIRSRTSYDVLPTSFRLIVFDVTLFVKTSLSLLTLNNIVSAPLWDSEANKFAGLLTMADFVNVIKYYYQSSSFPEAIAEIDKFRLLGLREVERKIGAIPPETIYVHPMHSLMDACLAMSKSrArRIPLIDVDGETGSEMIVSVLTqYRILKFISMNCKETAMLRVPLNQMTIgtWSnlaTASMETKVYDVIKMLAEknisaVpIVNSEGTLLNVYESVDVMHLIQDGDYSNLDLSVGEALLKRPANFDGVHTCRATDRLDGIFDAIKHSRVHrLfVVDENLKLEGiLsladILNYIIYDKTTTPGVPEQTDNFESAV

2222222222222222222222222222222222222222222222222222222222222222222222222222222222222222222222222222222222222222222222222222222222222222221212222222222222222222221222222222222222222222222221122111222222222222222221111121222222222222222222222222222222222222222222222222222222222222222222222121222222222212111122222222222222222222222222

>1VJC_A

SLSNKLTLDKLDVKGKRVVMRVDFNVPMKNNQITNNQRIKAAIPSIKFCLDNGAKSVVLMSHLGRPDGIPMPDKYSLEPVAVELKSLLGKDVLFLKDCVGPEVEKACADPAAGSVILLENLRFHVEEEGKGKDASGSKVKADPAKIEAFRASLSKLGDVYVNDAFGTAHRAHSSMVGVNLPKKAGGFLMKKELNYFAKALESPERPFLAILGgakVAdkIQLINNMLDKVNEMIIGggMAfTFLKVLNNMEIGTslFDEEGSKIVKDLMSKAEKNGVKITLPVDFVTADKfDENAKTGQATVASGIPAGWmgldCGPESSKKYSEAVARAKQIVWngpvgvfeWEAFAQGTKALMDEVVKATSRGCITIIGGgdTATCCAKWNTEDKVSHVSTGGGASLELLEGKVLPGVDALSNV

22222222222222222222222222222222222222222222222222222222222222222222222222222222222222222222222222222222222222222222222222222222222222222222222222222222222222222222222222222222222222222222222222222222222222222222111221122222222222222222112212222222222222112222222222222222222222222222222222122222222222222222221111222222222222222222222111111112222222222222222222222222222211222222222222222222222222222222222222222222

>1JI0_A

MVSDIVLEVQSLHVYyGAiHaIKGIDLKVPRGQIVTLIGAngagkttTLSAIAGLVRAQKGKIIFNGQDITNKPAHVINRMGIALVPeGRRIFPELTVYENLMMGAYNRKDKEGIKRDLEWIFSLFPRLKERLKQLGGTLSGGEQQMLAIGRALMSRPKLLMMdePSlGLAPILVSEVFEVIQKINQEGTTILLVEQNALGALKVAHYGYVLEtGQIVLEGKASELLDNEMVRKAYLGVA

222222222222222122121222222222222222222211111112222222222222222222222222222222222222222122222222222222222222222222222222222222222222222222222222222222222222222222211221222222222222222222222222222222222222222222222122222222222222222222222222

>1H1V_A

DEDETTALVCDNgsglVkAGFAGDDAPRAVFPSIVGRPRHQGVMVGMGQKDSYVGDEAQSKRGILTLKYPIEHgIITNWDDMEKIWHHTFYNELRVAPEEHPTLLTEAPLNPKANREKMTQIMFETFNVPAMYVAIqAVLSLYASGRTTGIVLdSgdgvTHNVPIYEGYALPHAIMRLDLAgRDLTDYLMKILTERGYSFVTTAEREIVrDIkeKLCYVALDFENEMATAASSSSLEKSYELPDGQVITIGNERFRCPETLFQPSFIGMESAGIHETTYNSIMKCDIDIRKDLYANNVMSggtTmypGIADRMQKEITALAPSTMKIKIIAPPERkYSVWIGGSILASLSTFQQMWITKQEYDEAGPSIVHRKCF

222222222222111121222222222222222222222222222222222222222222222222222222212222222222222222222222222222222222222222222222222222222222222212222222222222222121111222222222222222222222212222222222222222222222222221221122222222222222222222222222222222222222222222222222222222222222222222222222222222222222111211122222222222222222222222222221222222222222222222222222222222222222222

>1G21_H

MAMRQCAIYGKggigkstTTQNLVAALAEMGKKVMIVGCdPkAdSTRLILHSKAQNTIMEMAAEAGTVEDLELEDVLKAGYGGVKCVESGGPEPGVGCAGRGVITAINFLEEEGAYEDDLDFVFYdVGdVVCGGFAMPIRENKAQEIYIVCSGEMMAMYAANNISKGIVKYANSGSVRLGGLICNSRNTDREDELIIALANKLGTQMIHFVprdNVvqRAeIRRMTVIEYDPKAKqADEyRALARKVVDNKLLVIPNPITMDELEELLMEFGIMEVEDESIVGKTAEEV

2222222222211111112222222222222222222221212122222222222222222222222222222222222222222222222222222222222222222222222222222222212212222222222222222222222222222222222222222222222222222222222222222222222222222222222111221122122222222222222122212222222222222222222222222222222222222222222222222

>2ILY_A

GEIQWMRPSKEVGYPIINAPSKTKLEPSAFHYVFEGVKEPAVLTKNDPRLKTDFEEAIFSkYVGNKITEVDEYMKEAVDHYAGQLMSLDINTEQMCLEDAMYGTDGLEALDLSTSAGYPYVAMGKKKRDILNKQTRDTKEMQKLLDTYGINLPLVTYVkDELrSKTkVEQGkSrliEASSLNDSVAMRMAFGNLYAAFHKNPGVITGSAVGCDPDLFWSKIPVLMEEKLFAFdytgydaSLSPAWFEALKMVLEKIGFGDRVDYIDYLNHSHHLYKNKTYCVKGGMPsGCSGTSIFNSMINNLIIRTLLLKTYKGIDLDHLKMIAYGdDVIASYPHEVDASLLAQSGKDYGLTMtPADkSATFETVTWENVTFLKRFFRADEKYPFLIHPVMPMKEIHESIRWTKDPRNTQDHVRSLCLLAWHNGEEEYNKFLAKIRSVPIGRALDLPEYSTLYDRWLDSF

22222222222222222222222222222222222222222222222222222222222212222222222222222222222222222222222222222222222222222222222222222222222222222222222222222222222222122212221222212111222222222222222222222222222222222222222222222222222222221111111222222222222222222222222222222222222222222222222122222222222222222222222222222222222222212222222222222222222222222212221222222222222222222222222222222222222222222222222222222222222222222222222222222222222222222222222222222

>1KJ8_A

TLLGTALRPAATRVMLLGSGELGKEVAIECQRLGVEVIAVDRYADAPAMHVAHRSHVINMLDGDALRRVVELEKPHYIVPEIeAIATDMLIQLEEEGLNVVPCARATKLTMNrEGIRRLAAEELQLPTsTYRFADSESLFREAVADIGYPCiVkPVMsssgkGqTfIRSAEQLAQAWKYAQQGGRAGAGRVIVegvvKfDFeITLLTVSAVDGVHFCAPVGhRqEDgDYRESWQPQQMSPLALERAQEIARKVVLALGGYGLFGVeLfVCGDEVIFseVSPRPhdTGMVTLISQDLSEFALHVRAFLGLPVGGIRQYGPAASAVILPQLTSQNVTFDNVQNAVGADLQIRLFGKPEIDGSRRLGVALATAESVVDAIERAKHAAGQVKVQG

2222222222222222222222222222222222222222222222222222222222222222222222222222222222122222222222222222222222222222122222222222222212222222222222222222222121222111112121222222222222222222222222222111121221222222222222222222212122122222222222222222222222222222222222222121222222221122222112222222222222222222222222222222222222222222222222222222222222222222222222222222222222222222222222222222222

>1BCP_I

VAPGIVIPPKALFTQQGGAYGRCPNGTRALTVAELRGNAELQTYLRQITPGWSIYGLYDGTYLGQAYGGIIKDAPPGAGFIYRETFCITTIYKTGQPAADHYYSKVTATRLLASTNSRLCAVFVRDGQSVIGACASPYEGRYRDMYdALrrLLYMIYMSGLAVRVHVSKEEQYYDYEDATFQTYALTGISLCNPAASIC

2222222222222222222222222222222222222222222222222222222222222222222222222222222222222222222222222222222222222222222222222222222222222222222222222212211222222222222222222222222222222222222222222222222

>1E79_F

AAQASPSPKAGATTGRIVAVIGAVVDVQFDEGLPPILNALEVQGRETRLVLEVAQHLGESTVRTIAMDGTEGLVRGQKVLDSGAPIRIPVGPETLGRIMNVIGEPIDERGPIKTKQFAAIHAEAPEFVEMSVEQEILVTGIKVVDLLAPYAKGGKIGLFGGAGVGKTVLIMELINNVAKAHGGYSVFAGVGERTREGNDLYHEMIESGVINLKDATSKVALVYGQMNEPPGARARVALTGLTVAEYFRDQEGQDVLLFIDNIFRFTQAGSEVSALLGRIPSAVGYQPTLATDMGTMQERITTTKKGSITSVQAIYVPADDLTDPAPATTFAHLDATTVLSRAIAELGIYPAVDPLDSTsrImdPNIVGSEHyDVARGVQKILQDYKSLQDIIAILGMDELSEEDKLTVSRARKIQRFLSQPFQVAEVFTGHLGKLVPLKETIKGFQQILAGEYDHLPEQAFYMVGPIEEAVAKADKLAEEHS

22222222222222222222222222222222222222222222222222222222222222222222222222222222222222222222222222222222222222222222222222222222222222222222222222222222222222222222222222222222222222222222222222222222222222222222222222222222222222222222222222222222222222222222222222222222222222222222222222222222222222222222222222222222222222222222222222222222222222222222221121122222222122222222222222222222222222222222222222222222222222222222222222222222222222222222222222222222222222222222222222

>1XDP_B

GQEKLYIEKELSWLSfNERvLQEAADKSNPLIERMRfLGiYSnnLDeFYKVrFAELKRRIIISEEQGSNSHSRHLLGKIQSRVLKADQEFDGLYNELLLEMARNQIFLINERQLSVNQQNWLRHYFKQYLRQHITPILINPDTDLVQFLKDDYTYLAVEIIRGDTIRYALLEIPSDKVPRFVNLPPEAPRRRKPMILLDNILRYCLDDIFKGFFDYDALNAYSMKMTRDAEYDLVHEMEASLMELMSSSLKQRLTAEPVRFVYQRDMPNALVEVLREKLTISRYDSIVPGGRYHNFKDFINFPNVGKANLVNKPLPRLRHIWFDKAQFRNGFDAIRERDVLLYYPYHTFEHVLELLRQASFDPSVLAIKINIyrVAKDSRIIDSMIHAAHNGKKVTVVVELQArFDEEANIHWAKRLTEAGVHVIFSAPGLKIhAkLFLISRKENGEVVRYAHIGTGnFNEKtARlyTDYSLLTADARITNEVRRVFNFIENPYRPVTFDYLMVSPQNSRRLLYEMVDREIANAQQGLPSGITLKLnNLVDKGLVDRLYAASSSGVPVNLLVrGMCSLIPNLEGISDNIRAISIvDRYlEhDrVYIFENGGDKKVYLSSADWMTRNIDYrIEVATPLLDPRLKQRVLDIIDILFSDTVKARYIDKELSNRYVPRGNRRKVRAQLAIYDYIKSLEQPE

222222222222222122212222222222222222122122112212222122222222222222222222222222222222222222222222222222222222222222222222222222222222222222222222222222222222222222222222222222222222222222222222222222222222222222222222222222222222222222222222222222222222222222222222222222222222222222222222222222222222222222222222222222222222222222222222222222222222222222222222222222222222112222222222222222222222222222212222222222222222222222222222212122222222222222222222212222122112222222222222222222222222222222222222222222222222222222222222222222221222222222222222222222222212222222222222222222221222121212222222222222222222222222212222222222222222222222222222222222222222222222222222222222222222222

>2V92_E

MESVAAESAPAPENEHSQETPESNSSVYTTFMKSHRCYDLIPTSSKLVVFDTSLQVKKAFFALVTNGVrAAPLWDSKKQSFVgmLtitdFInILHRYYKSALVQIYELEEHKIETWrEVYLQDSFkplvCISPNASLFDAVSSLIRNkihrLpVIDPESGNTLYILTHKRILKFLKLFITEFPKPEFMSKSLEELQIGTYANIAMVRTTTPVYVALGIFVQHRvsALPVVDEKGRVVDIYSkFDVINLAAEKTYNNLDVSVTKALQHRSHYFEGVLKCYLHETLEAIINRLVEAEVhRLVVVDEHDVVKGIVSLSDILQALVLTGGEKKP

222222222222222222222222222222222222222222222222222222222222222222221222222222222211211112212222222222222222222222221222222221111222222222222222222111121222222222222222222222222222222222222222222222222222222222222222222222211222222222222222212222222222222222222222222222222222222222222222222222221222222222222222222222222222222222

>1J09_A

MVVTRIApsPtGDPhVgtAyiALFNYAWARRNGGRFIVRIEDTDRARYVPGAEERILAALKWLGLSYDEGPDVGGPHGPYRQSERLPLYQKYAEELLKRGWAYRAFETPEELEQIRKEKGGYDGRARNIPPEEAEERARRGEPHVIRLKVPRPGTTEVKDELRGVVVYDNQEIPDVVLLKSDGYPTYHLANVVDDHLMGVTDVIRaEewLVSTPIHVLLYRAFGWEAPRFYHMPllRNPDKTkiskrKSHTSLDWYKAEGFLPEALRNYLCLMGFSMPDGREIFTLEEFIQAFTWERVSLGGPVFDLEKLRWMNGKYIREVLSLEEVAERVKPFLREAGLSWESEAYLRRAVELMRPRFDTLKEFPEKARYLFTEDYPVSEKAQRKLEEGLPLLKELYPRLRAQEEWTEAALEALLRGFAAEKGVKLGQVAQPLRAALTGSLETPGLFEILALLGKERALRRLERALA

222222211212221211211222222222222222222222222222222222222222222222222222222222222222222222222222222222222222222222222222222222222222222222222222222222222222222222222222222222222222222222222222222222222222212112222222222222222222222222112222221111122222222222222222222222222222222222222222222222222222222222222222222222222222222222222222222222222222222222222222222222222222222222222222222222222222222222222222222222222222222222222222222222222222222222222222222222222222

>1BCP_K

DVPYVLVKTNMVVTSvAmKPYEVTPTRMLvCGIAAKLGAAASSPDAHVPFCFGkDLKRPgsSPMevMlrAVfmQQRPLRMFLGPKQLTFEGKPALELIRMVECSGKQDCP

22222222222222212122222222222122222222222222222222222122222112221121122112222222222222222222222222222222222222

>1BCP_L

GLPTHLYKNFTVQELALKLKGKNQEFCLTAFMSGRSLVRACLSDAGHEHDTWFdtMLgfAIsAYAlKSRIALTVEDSPYPGTPGDLLELQiCPLNGYCE

222222222222222222222222222222222222222222222222222221122112212221222222222222222222222222122222222

>1X01_B

MLGLKTSIIGRRVIYFQEITSTNEFAKTSYLEEGTVIVADKQTMGHgrlNrKweSpEGGLwLSIVLSPKVPQKDLPKIVFLGAVGVVETLKEFSIDGRIkWPndVLVNYKkIAGVLVEGKGDKIVLGIGLnVNnkVpNGaTSMKLELGSEVPLLSVFRSLITNLDRLYLNFLKNPMDILNLVRDNMILGVRVKILGDGSFEGIAEDIDDFGRLIIRLDSGEVKKVIYGDVslRFL

2222222222222222222222222222222222222222222222111212112122221222222222222222222222222222222222222221221122222212222222222222222222122112122122222222222222222222222222222222222222222222222222222222222222222222222222222222222222222211222

>3BJU_D

MSVDPNQYYKIRSQAIHQLKVNGEDPYPHKFHVDISLTDFIQKYSHLQPGDHLTDITLKVAGRIHAKRASGGKLIFYDLRGEGVKLQVMANSRNYKSEEEFIHINNKLRRGDIIGVQGNPGKTKKGELSIIPYEITLLSPCLHMLPHLHFGLKDKETRYRQRYLDLILNDFVRQKFIIRSKIITYIRSFLDELGFLEIETPMMNIIPGGavAKPFITYHNELDMNLYMRIAPELYHKMLVVGGIDRVYEIGRQFrNeGIDLThNPEfTtCEFYMAYADYHDLMEITEKMVSGMVKHITGSYKVTYHPDGPEGQAYDVDFTPPFRRINMVEELEKALGMKLPETNLFETEETRKILDDICVAKAVECPPPRTTARLLdKLVGEFLeVTCINPTFICDHPQIMSPLAKWHRSKEGLTERFELFVMKKeICnAYTELNDPMRQRQLFEEQAKAKAAGDDEAMFIDENFCTALEYGLPPTAGWgmgIdrVAMFLTDSNNiKEVLLFPAMKPEDKKENLEHHHHHH

22222222222222222222222222222222222222222222222222222222222222222222222222222222222222222222222222222222222222222222222222222222222222222222222222222222222222222222222222222222222222222222222222222222222222222112222222222222222222222222222222222222222222121222221222121222222222222222222222222222222222222222222222222222222222222222222222222222222222222222222222222222222222221222222212222222222222222222222222222222222222222122122222222222222222222222222222222222222222222222222111211222222222212222222222222222222222222

>2VHQ_C

MIHLYDAKSFAKLRAAQYAAFHTDAPGSWFDHTSGVLESVEDGTPVLAIGVESGDAIVFDKNAQRIVAYKEKSVKAEDGSVSVVQVENGFMKQGHRGWLVDLTGELVGCSPVVAEFGGHRYASGMVIVTGKGnsgktpLVHALGEALGGKDKYATVRFGePLSGYNTDFNVFVDDIARAMLQHRVIVIDSLkNVIGAAGGNTTSGGISRGAFDLLSDIGAMAASRGCVVIASLnPTSNDDKIVELVKEASRANSTSLVISTDVDGEWQVLTRTGEGLQRLTHTLQTSygEHsVLTIHTSKQSGGKQASGKAIQTVIKNDELESVLRRLTSN

2222222222222222222222222222222222222222222222222222222222222222222222222222222222222222222222222222222222222222222222222222222222221111112222222222222222222221222222222222222222222222222222212222222222222222222222222222222222222222212222222222222222222222222222222222222222222222222222211221222222222222222222222222222222222222222

>1U5V_A

MNLRAAGPGWLFCPADAPEAFAAAAAAADVVILDLEDGVAAAQKPAARNALRDTPLDPERTVVRINAGGTADQARDLEALAGTAYTTVMLPKAESAAQVIELAPRDVIALVETARGAVCAAEIAAADPTVGMMWGAEDLIATLGGSSSRrAdGAYRdVArHVRSTILLAASAFGRLALDAVHLDILDVEGLQEEARDAAAVGFDVTVCIHPSQIPVVRKAYAASHEKLAWARRVLAASRSERGAFAFEGQMVDSPVLTHAETMLRRAGEATSE

222222222222222222222222222222222222222222222222222222222222222222222222222222222222222222222222222222222222222222222222222222222222222222222222222221212222122122222222222222222222222222222222222222222222222222222222222222222222222222222222222222222222222222222222222222222

>1N48_A

MIVLFVdFdyFyAQVEEVLNPSLKGKPVVVCvFSGRFEDSGAvatANyEArKFGVKagIPIVEAKKILPNAVYLPmRKEVYQQVSSRIMNLLREYSEKIEIASidEAYLDISDKVRDYREAYNLGLEIKNKILEKEKITVTVGISKNKVFAkIAAdMAkPNGIKVIDDEEVKRLIRELDIADVPGIGNITAEKLKKLGINKLVDTLSIEFDKLKGMIGEAKAKYLISLARDEYNEPIRTRVRKSIGRIVTMKRNSRNLEEIKPYLFRAIEESYYKLDKRIPKAIHVVAVTEDLDIVSRGRTFPHGISKETAYSESVKLLQKILEEDERKIRRIGVRFSKFIEAIGLDKFFDT

2222221211212222222222222222222122222222221112212212222211222222222222222221222222222222222222222222222112222222222222222222222222222222222222222222222122212212222222222222222222222222222222222222222222222222222222222222222222222222222222222222222222222222222222222222222222222222222222222222222222222222222222222222222222222222222222222222222222222222

>4AT1_D

MTHDNKLGveaiKRGTvIdhIPAQIGFKLLSLFKLTETDQRITIGLNLPSGeMGRkDlIkIENTFLSEDQVDQLALYAPQAtVnRiDNyevVGkSRPSLPERIDNVLVCPNSNCISHAEPVSSSFAVRKRANDIALKCKYCEKEFSHNVVLAN

222222221111222212112222222222222222222222222222222122212121222222222222222222222121212211122122222222222222222222222222222222222222222222222222222222222

>1KP8_N

AAKDVKFGNDAGVKMLRGVNVLADAVKVtlgpKGRNVVLDKSFGAPTITkdgvSVAREIELEDKFENMGAQMVKEVASKAnDAAGdgtttATVLAQAIITEGLKAVAAGMNPMDLKRGIDKAVTVAVEELKALSVPCSDSKAIAQVGTISANSDETVGKLIAEAMDKVGKEGVITVEDGTGLQDELDVVEGMQFDRGYLSPYFINKPETGAVELESPFILLADKKISNIREMLPVLEAVAKAGKPLLIIAEDVEGEALATLVVNTMRGIVKVAAVKAPGFGDRRKAMLQDIATLTGGTVISEEIGMELEKATLEDLGQAKRVVINKDTTTIIDGVGEEAAIQGRVAQIRQQIEEATSDYDREKLQERVAKLAGGVAVIKVGAATEVEMKEKKARVEDALHATRAAVEEGVVAgggVALIRVASKLADLRGQNADQNVGIKVALRAMEApLRQiVLNCGEEPSVVANTVKGGDGNYGynaaTEEYGNMIDMGildPTKVTRSALQYAASVAGLMITTECMVTDLPKNDAADLGAAGGMGGMGGMGGMM

2222222222222222222222222222111122222222222222222111122222222222222222222222222212222111112222222222222222222222222222222222222222222222222222222222222222222222222222222222222222222222222222222222222222222222222222222222222222222222222222222222222222222222222222222222222222222222222222222222222222222222222222222222222222222222222222222222222222222222222222222222222222222222222222222222222222222222222222222222111222222222222222222222222222222222122212222222222222222222222211112222222222211122222222222222222222222222222222222222222222222222222

>1XDN_A

GHMDQSDFSPyieiDLPSESRIQSLHKSGLAAQEWVAcekvhGtnFGIYLINQGDHEVVRFAKrSGIMDPNENFFGYHILIDEFTAQIRILNDLLKQKYGLSRVGRLVLNGeLFGAKYKHPLVPKSEKWCTLPNGKKFPIAGVQIQREPFPQYSPELHFfAfDIKYSVSGAEEDFVLLGYDEFVEFSSKVPNLLYaRALVRGTLDECLAFDVENFMTPLPALLGLGNYPLEGNLAEGVvIrHVRRGDPAVEKHNVSTiIkLrCSSFMELKHPGKQKE

2222222222111122222222222222222222222111112112222222222222222221222222222222222222222222222222222222222222222221222222222222222222222222222222222222222222222221212222222222222222222222222222222221222222222222222222222222222222222222222222121222222222222222212121222222222222222

>2EWW_A

MFEKQEVEQKKELKILEIIKEAIELGASDIHLTAGAPPAVRIDGYIKFLKDFPRLTPEDTQKLAYSVMSEKHRQKLEENGQVDFSFGVRGVGRFRANVFYQRGSVAAALrSLPAEIpEFKklGLPDKVLELCHRKMGLILVTGPtgsgksttIASMIDYINQTKSYHIITIEDPIeyVFKHKKSIVNQREVGEDTKSFADALRAALREDPDVIFVGeMRDLETVETALRAAETGHLVFGtLhTNTAIDTIHRIVDIFPLNQQEQVRIVLSFILQGIISQRlLPKIGGGrvlAYELLIPNTAIRNLIRENKLQQVYSLMQSGQAETGMQTMNQTLYKLYKQGLITLEDAMEASPDPKELERMIRGGRHHHHHH

222222222222222222222222222222222222222222222222222222222222222222222222222222222222222222222222222222222222212222221222112222222222222222222222111111112222222222222222222222211222222222222222222222222222222222222222122222222222222222222221212222222222222222222222222222222222222212222222111222222222222222222222222222222222222222222222222222222222222222222222222222222222

>1TF7_A

MTSAEMTSPNNNSEHQAIAKMRTMIEGFDDISHGGLPIGRSTLVSGTSGTGKTLFSIQFLYNGIIEFDEPGVFVTFEETPQDIIKNARSFGWDLAKLVDEGKLFILDASPDPEGQEVVGGFDLSALIERINYAIQKYRARRVSIDSVTSVFQQYDASSVVRRELFRLVARLKQIGATTVMTTERIEEYGPIARYGVEEFVSDNVVILRNVLEGERRRRTLEILKLRGTSHMKGEYPFTITDHGINIFPLGAMRLTQRSSNVRVSSGVVRLDEMCGGGFFKDSIILATGAtgtgktlLVSRFVENACANKERAILFAYeeSRAQLLRnAYswGMDFEEMERQNLLKIVCAYPESAGLEDHLQIIKSEINDFKPARIAIDSLSALARGVSNNAFRQFVIGVTGYAKQEEITGLFtNtSDQFMGAHSITDSHISTITDTIILLQYVEIRGEMSrAINVFKMRGSWHDKAIREFMisdKGPDIKDSFRNFERIISGSPTRITVDEKSELSRIVRGVQEKGPESHHHHHH

222222222222222222222222222222222222222222222222222222222222222222222222222222222222222222222222222222222222222222222222222222222222222222222222222222222222222222222222222222222222222222222222222222222222222222222222222222222222222222222222222222222222222222222222222222222222222222222222211111112222222222222222222221122222221221122222222222222222222222222222222222222222222222222222222222222222222222222222222212122222222222222222222222222222222222122222222222222222222111222222222222222222222222222222222222222222222222222

>2HMU_B

MGRIKNKQFAViglgrfGGSIVKELHRMGHEVLAvdinEEkVNAYASYATHAVIAnatEENELLSLGIRNFEYVIVAiganIQaSTLtTLLLKELDIPNIWVkAQNYYHHKVLEKIGADRIIHPEKDMGVKIAQSLSDENVLNY

222222222221111112222222222222222211112212222222222222211122222222222222222221111221222122222222222222122222222222222222222222222222222222222222

>1H4Q_A

MAKEKGLTPQSQDFSEWYLEVIQKAELADYGPVRGTIVVRPYGYAIWENIQQVLDRMFKETGHQNAYFPLFIPMSFLRKEAEHVEGFSPELAVVTHAGGEELEEPLAVRPTSETVIGYMWSKWIRSWRDLPQLLNQWGNVVrWeMRTRPflrtSEfLwQEGHTAHATREEAEEEVRRMLSIYARLAREYAAIPVIEGLkTEKeKfAGAVYTTTIEALMKDGKALqagtShYLGENFARAFDIKFQDRDLQVKYVHTTSWgLswrFIGAIIMTHGDDRGLVLPPRLAPIQVVIVPIYKDESRERVLEAAQGLRQALLAQGLRVHLDDRDQHTPGYKFHEWELKGVPFRVELGPKDLEGGQAVLASRLGGKETLPLAALPEALPGKLDAFHEELYRRALAFREDHTRKVDTYEAFKEAVQEGFALAFHCGDKACERLIQEETTATTrCVPFEAEPEEGFCVRCGRPSAYGKRVVFAKAy

222222222222222222222222222222222222222222222222222222222222222222222222222222222222222222222222222222222222222222222222222222222222222222222121222221111221212222222222222222222222222222222222222222122212122222222222222222221111212222222222222222222222222222212111222222222222222222222222222222222222222222222222222222222222222222222222222222222222222222222222222222222222222222222222222222222222222222222222222222222222222222222222222222222222122222222222222222222222222222221

>1ATP_E

GNAAAAKKGSEQESVKEFLAKAKEDFLKKWETPSQNTAQLDQFDRIKTlgtgsfgRvMLVKHKESGNHYaMkILDKQKVVKLKQIEHTLNEKRILQAVNFPFLvKLEFSFKDNSNLYMVmeyvAGGeMFSHLRRIGRFSEPHARFYAAQIVLTFEYLHSLDLIYRDLkPenLlIDQQGYIQVtdFGFAKRVKGRTWTLCGTPEYLAPEIILSKGYNKAVDWWALGVLIYEMAAGYPPFFADQPIQIYEKIVSGKVRFPSHFSSDLKDLLRNLLQVDLTKRFGNLKNGVNDIKNHKWFATTDWIAIYQRKVEAPFIPKFKGPGDTSNfDDYEEEEIRVSINEKCGKEFTEF

22222222222222222222222222222222222222222222222211111112122222222222212122222222222222222222222222222221222222222222222111122212222222222222222222222222222222222222222121121222222222112222222222222222222222222222222222222222222222222222222222222222222222222222222222222222222222222222222222222222222222222222222222222222222222122222222222222222222222

>1MJH_B

MSVMYKKILyptdFSETaEIALKHVKAFKTLKAEEVILlhvIdEREIKKRDIFSLLLGVAGLNKSVEEFENELKNKLTEEAKNKMENIKKELEDVGFKVKDIIVVGIpHEEiVKIAEDEGVDIIImgshgktnlKEiLLgsvtENVIKKSNKPVLVVKRKNS

222222222111122221222222222222222222221112122222222222222222222222222222222222222222222222222222222222222221222122222222222221111111112212211112222222222222222222

>2PBZ_C

MSLIVSTIASHSSLQILLGAKKEGFKTRLYVSPKRRPFYSSLPIVDDLVVAEEMTSILNDDGIVVPHGSFVAYLGIEAIEKAKARFFGNRRFLKWETTFELQDKALEGAGIPRvEVVEPEDAKPDELYfVriEGPRGGSGHFIVeGSELEERLSTLEEPYRVeRfiPGVyLyVHFFYSPILERLELLGVDErVlIADGNARWPVKPLPyTiVGNRAIALRESLLPQLYDYGLAFVRTMRELEPPGVIGPFALhFAYDGSFKAiGIASrIDGGSNADHWYSELYWGERLSMGRRIARELRLAEEEDRLEEVVTEGHHHHHH

22222222222222222222222222222222222222222222222222222222222222222222222222222222222222222222222222222222222222222122222222222222121122222222222212222222222222222212112221212222222222222222222121222222222222221212222222222222222222222222222222222222222212222222221222212222222222222222222222222222222222222222222222222222

>1JKN_A

GPLGSMDSPPEGYRRnVGICLMNNDKKIFAASrLDiPDawqMPqggIDEGEDPRNAAIRELREETGVTSAEVIAEVPYWLTyDfPPKvREklNIQwGSDWKGqAQKWFLFKFTGQDQEINLLGDGSEKPeFGEWSWVTPEQLIDltvEfkKPVYKEVLSVFAPHL

222222222222222122222222222222221221221112211122222222222222222222222222222222222121222122112221222222122222222222222222222222222122222222222222111211222222222222222

>8ICN_A

MSKRKAPQETLNGGITDMLTELANFEKNVSQAIHKYNAYRKAASVIAKYPHKIKSGAEAKKLPGVGTKIAEKIDEFLATGKLRKLEKIRQDDTSSSINFLTRVSGIGPSAARKFVDEGIKTLEDLRKNEDKLNHHQRIGLKYFGDFEKrIPREEmLQMQDIVLNEVKKVDSEYIATVCgsfRrGAESsgdMDVLLTHPSFTSESTKQPKLLHQVVEQLQKVHFITDTLSKGETKFMGVCQLPSKNDEKEYPHRRIDIRLIPKDQYYCGVLYFTGSDIFNKNMRAHALEKGFTINEYTIRPLGVTGVAGEPLPVDSEKDIFDYIQWKYREPKDRSE

22222222222222222222222222222222222222222222222222222222222222222222222222222222222222222222222222222222222222222222222222222222222222222222222222221222221222222222222222222222221112122221112222222222222222222222222222222222222222222222222222222222222222222222222222222222222222222222222222222222222222222222222222222222222222222222222

>1GOL_A

HHHHHHMAAAAAAGPEMVRGQVFDVGPRYTNLSYigeGAyGMvCSAYDNLNKVRVaIrKISPFEHQTYCQRTLREIKILLRFRHENIiGINDIIRAPTIEQMKDVYIVqdlmetdLYkLLKTQHLSNDHICYFLYQILRGLKYIHSANVLHRDLkPsNLlLNTTCDLKIcdFGLARVADPDHDHTGFLTEYVATRWYRAPEIMLNSKGYTKSIDIWSVGCILAEMLSNRPIFPGKHYLDQLNHILGILGSPSQEDLNCIINLKARNYLLSLPHKNKVPWNRLFPNADSKALDLLDKMLTFNPHKRIEVEQALAHPYLEQYYDPSDEPIAEAPFKFDMELDDLPKEKLKELIFEETARFQPGYRS

2222222222222222222222222222222222111221221222222222222121222222222222222222222222222221222222222222222222221111111221222222222222222222222222222222222222121221222222222112222222222222222222222222222222222222222222222222222222222222222222222222222222222222222222222222222222222222222222222222222222222222222222222222222222222222222222222222222222222222222222222222

>1UA2_D

MALDVKSRAKRYEKLDFlgegqfaTvYKARDKNTNQIVaIkKIKLGHRSEAKDGINRTALREIKLLQELSHPNIiGLLDAFGHKSNISLVfdfmetdLEVIIKDNSLVLTPSHIKAYMLMTLQGLEYLHQHWILHRdLkPnnLlLDENGVLKLAdFGLAksFGSPNRAYTHQVVTRWYRAPELLFGARMYGVGVDMWAVGCILAELLLRVPFLPGDSDLDQLTRIFETLGTPTEEQWPDMCSLPDYVTFKSFPGIPLHHIFSAAGDDLLDLIQGLFLFNPCARITATQALKMKYFSNRPGPTPGCQLPRPNCPVETLKEQSNPALAIKRKRTEALEQGGLPKKLIF

2222222222222222211111112122222222222212122222222222222222222222222222222212222222222222221111111222222222222222222222222222222222222222121211212222222222122221122222222222222222222222222222222222222222222222222222222222222222222222222222222222222222222222222222222222222222222222222222222222222222222222222222222222222222222222222222222222222222

>1A0I_A

VNIKTNPfkaVSFVESAIKKALDNAGYLIAeikyDGVrGNICVDNTANSYWLSrVSKTIPALEHLNGFDVRWKRLLNDDRCFYKDGFMLDGeLMVKGVDFNTGSGLLRTKWTDTKNQEFHEELFVEPIRKKDKVPFKLHTGHLHIKLyAILPLHIVESGEDCDVMTLLMQEHVKNMLPLLQEYFPEIEWQAAESYEVYDMVELQQLYEQKRAEGHEGLiVkDPMCIYKRGKKSGwWkMkPeNEADGIIQGLVWGTKGLANEGKVIGFEVLLESGRLVNATNISRALMDEFTETVKEATLSQWGFFSPYGIGDNDACTINPYDGWACQISYMEETPDGSLRHPSFVMFR

222222211122222222222222222222111122212222222222222221222222222222222222222222222222222222212222222222222222222222222222222222222222222222222222222122222222222222222222222222222222222222222222222222222222222222222222221212222222222222121212122222222222222222222222222222222222222222222222222222222222222222222222222222222222222222222222222222222222

>2C01_X

MKPPQFTwAQwfETqhINMTSQQCTNAMQVINNYqRrCkNQNTFLLTTFANVVNVCGNPNMTcPsnKTrKnCHHSGSQVPLIHCNLTTPSPQNISNCRYAQTPANMFYIVaCDNRDQRRDPPQYPVVPvhldRII

222222212211221122222222222222222212121222222222222222222222221211221212222222222222222222222222222222222222221222222222222222221111222

>2Z08_A

MFKTILLaydGsEHaRRAAEVAKAEAEAHGARLIVVhaYEPVPDYLGEPFFEEALRRRLERAEGVLEEARALTGVPKEDALLLEGVpAEAiLQAARAEKADLIVmgtrgLgaLGSlFLgsqsQRVVAEAPCPVLLVR

22222221112122122222222222222222222211222222222222222222222222222222222222222222222222122212222222222222111112112221221111222222222222222

>1SU2_B

MEHDERTHVPVElraAGVVLLNERGDILLVQEKGIPGHPEKAGLWHIPsgaVEDGENPQDAAVREACEETGLRVRPVKFLGAYLGRfpdGvLiLrHVWLAEPEPGQTLAPAFTDEIAEASFVSREDFAQLYAAGQIRmYQTKLFYADALREKGFPALPV

222222222222111222222222222222222222222222222222111222222222222222222222222222222222221112121212222222222222222222222222222222222222222221222222222222222222222

>1JJV_A

MTYIVGLTGGigsgkttIANLFTDLGVPLVDADVVAREVVAKDSPLLSKIVEHFGAQILTEQGELNRAALRERVFNHDEDKLWLNNLLHPAIRERMKQKLAEQTAPYTLFVVpLLIENKLTALCDRILVVDVSPQTQLArsAQRDNNNFEQIQRIMNSQVSQQERLKWADDVINnDaelAQNLPHlQQKVLELHQFYLQQAENKNA

22222222221111111222222222222222222222222222222222222222222222222222222222222222222222222222222222222222222222221222222222222222222222222221122222222222222222222222222222222212111222222122222222222222222222

>1F9A_A

LRGFiigrfqPFhKghLEvIKKIAEEVDEIIIGIGSAQKSHTLENPFTAGERILMITQSLKDYDLTYYPIPIKDIEFNSIWVSYVESLTPPFDIVysgNPLVRVLFEERGYEVKRpemfNrKEysgtEIrRRMLNGEKWEHLVPKAVVDVIKEIKGVERLRKLAQTDK

222211111122121122122222222222222222222222222222222222222222222222222222222222222222222222222221112222222222222222211112122111122122222222222222222222222222222222222222

>2NT8_A

MGSSHHHHHHSSGLVPRGSHMASMTGGQQMGRDRSVKiytkngdKGqtriIGKQILYkNDPRvaAygEVdELNSWVGYTKSLINSHTQVLSNELEEIQQLLFDCGHDLaTPADDERHSFKFKQEQPTVWLEEKIDNYTQVVPAVKKFILPGGTQLASALHVARTITRRAERQIVQLMREEQINQDVLIFINRLSDYFFAAARYANYLEQQPDMLYRNSKDVFR

2222222222222222222222222222222222222111111122111122222221222211211221222222222222222222222222222222222222221222222222222222222222222222222222222222222222222222222222222222222222222222222222222222222222222222222222222222222

>3C4W_B

MDFGSLETVVANSAFIAARGSFDASSGPASRDRKYLARLKLPPLSKCEALRESLDLGFEGMCLEQPIGKRLFQQFLRTHEQHGPALQLWKDIEDYDTADDALRPQKAQALRAAYLEPQAQLFCSFLDAETVARARAGAGDGLFQPLLRAVLAHLGQAPFQEFLDSLYFLRFLQWKWLEAQPMGEDWFLDFRVlgrggfgEvFACQMKATGKLYaCkKlNKKRLKKRKGYQGAMVeKKILAKVHSRFIvSLAYAFETKTDLCLVmtImNGGdIRYHIYNVDEDNPGFQEPRAIFYTAQIVSGLEHLHQRNIIYRdLkPeNVlLDDDGNVRIsdLGLAVELKAGQTKTKGYAGTPGFMAPELLLGEEYDFSVDYFALGVTLYEMIAARGPFRARGEKVENKELKQRVLEQAVTYPDKFSPASKDFCEALLQKDPEKRLGFRDGSCDGLRTHPLFRDISWRQLEAGMLTPPFVPDSRTVYAKNIQDVGAFSTVKGVAFEKADTEFFQEFASGTCPIPWQEEMIETGVFGDLNVWRPDGVDHHHHHH

222222222222222222222222222222222222222222222222222222222222222222222222222222222222222222222222222222222222222222222222222222222222222222222222222222222222222222222222222222222222222222222222111111121222222222222121212222222222222222122222222222212222222222222221121222122222222222222222222222222222222222222222212121221222222222112222222222222222222222222222222222222222222222222222222222222222222222222222222222222222222222222222222222222222222222222222222222222222222222222222222222222222222222222222222222222222222222222222222222222222222

>1OJL_E

GSHmiGSSPAMQHLLNEIAMVAPSDATVLIHGDsgtgkelVARALHACSARSDRPLVTLNCAALNESLLESELFGHEKGAFTGADKRREGRFVEADGGTLFLdeIGDISPLMQVRLLRAIQEREVQRVGSNQTISVDVRLIAATHRDLAEEVSAGRFRQDLYYRLNVVAIEMPSLRQrREDIPLlADhfLRrFAERNRKVVKGFTPQAMDLLIHYDWPGNirELeNAIERAVVLLTGEYISERELPLAIAATPIKTEYSGEIQPLVDVEKEVILAALEKTGGNKTEAARQLGITRKTLLAKLSR

2221122222222222222222222222222221111111222222222222222222222222222222222222222222222222222222222222221122222222222222222222222222222222222222222222222222222222222222222222222221222222122112212222222222222222222222222222112212222222222222222222222222222222222222222222222222222222222222222222222222222222

>1G3I_F

MSEMTPREIVSELDQhiiGQADAKRAVAIALRNRWRRMQLQEPLRHEVTPKNILMIGPtgvgkteIARRLAKLANAPFIkVEATKFTEVGYVGKEVDSIIRDLTDSAMKLVRQQEIAKNRARAEDVAEERILDALLPPAKNQWGEVENHDSHSSTRQAFRKKLREGQLDDKEIEIDVSAGVSMGVEIMAPPGMEEMTNQLQSLFQNLGSDKTKKRKMKIKDALKALIDDEAAKLINPEELKQKAIDAVEQNGIVFIdeIDKICKKGEYSGADVSREGVQRDLLPLVEGSTVSTKHGMVKTDHILFIASgaFQVARPSDLIPELQGRLPIRVELTAlSAADFERiLTEpHASLTEQYKALMATEGVNIAFTTDAVKKIAEAAFRVNEKTENIGarRLhTVMERLMDKISFSASDMNGQTVNIDAAYVADALGEVVENEDLSRFIL

222222222222222111222222222222222222222222222222222222222211111112222222222222212222222222222222222222222222222222222222222222222222222222222222222222222222222222222222222222222222222222222222222222222222222222222222222222222222222222222222222222222222222211222222222222222222222222222222222222222222222222221122222222222222222222222221222222212221222222222222222222222222222222222222222222221122122222222222222222222222222222222222222222222222

>1YFR_B

MSLSAHEIRELFLSFFEKKGHTRVKSAPLVPENDPTLLFVNAGMVPFKNVFLGLEKRPYKRATSCQKCLrVSGKHNdLEQVGYTSrhhTFfEmLGNFSFGDYFKKEAIEYAWEFVTEVLKLPKEKLYVSVYKDDEEAYRIWNEHIGIPSERIWRLGEEDNFWQMGDVGPCGPSSEIYVDRGEEYEGDERYLeIWnLVFMQYNRDENGVLTPLPHPNIDTgmgLerIASVLQGKNSnFEIDIIFPLIQFGEEVSGKKYGEKFETDVALRVIADHLRAITFAISDGVIPSNEGRGYVIRRILRRAMRFGYKLGIENPFLYKGVDLVVDIMKEPYPELELSREFVKGIVKGEEKRFIKTLKAGMEYIQEVIQKALEEGRKTLSGKEVFTAYDTYGFPVDLIDEIAREKGLGIDLEGFQCELEEQRERARKHFKVEAKKVKPVYSHLKELGKTSAFVGAAALEHHHHHH

222222222222222222222222222222222222222222222222222222222222222222222122222212222222211122121222222222222222222222222222222222222222222222222222222222222222222222222222222222222222222222222221221222222222222222222222222111211222222222212222222222222222222222222222222222222222222222222222222222222222222222222222222222222222222222222222222222222222222222222222222222222222222222222222222222222222222222222222222222222222222222222222222222222222222222222222222222222

>1A82_A

SKRYFVTGTDtevgktvASCALLQAAKAAGYRTAGYkPVASGSEKTPEGLRNSdALALQRNSSLQLDYATVNPYTFAEPTSPHIISAQEGRPIESLVMSAGLRALEQQADWVLVeGAgGWFTPLSDTFTFADWVTQEQLPVILVVGVKLGCINHAMLTAQVIQHAGLTLAGWVAndvTPPGKRHAEYMTTLTRMIPAPLLGEipwlaENpeNAATGKYINLALL

22222222221111111222222222222222222212222222222222222122222222222222222222222222222222222222222222222222222222222212212222222222222222222222222222222222222222222222222222222211122222222222222222222222221111122112222222222222

>1AYL_A

MRVNNGLTPQELEAYGISDVHDIVYNPSYDLLYQEELDPSLTGYERGVLTNLGAVAVDTGIFTGRSPKDKYIVRDDTTRDTFWWADKGKGKNDNKPLSPETWQHLKGLVTRQLSGKRLFVVDAFCGANPDTRLSVRFITEVAWQAHFVKNMFIRPSDEELAGFKPDFIVMNGAKCTNPQWKEQGLNSENFVAFNLTERMQLIGGTWYGGEMKkGMFSMMNYLLPLKGIASMhCSANVGEKGDVAVFFGlsgtgkttlSTDPKRRLIGDdEHGWDDDGVFNFEGGCyAkTikLSKEAePEIYNAIRRDALLENVTVREDGTIDFDDGSKTENTrVSYPIYHIDNIVKPVSKAGHATKVIFLTADAFGVLPPVSRLTADQTQYHFLSGFTAKLAGTERGITEPTPTFSACFGAAFLSLHPTQYAEVLVKRMQAAGAQAYLVNtGWNGTGKrisiKDtRAIIDAILNGSLDNAETFTLPMFNLAIPTELPGVDTKILDPRNTYASPEQWQEKAETLAKLFIDNFDKYTDTPAGAALVAAGPKLS

2222222222222222222222222222222222222222222222222222222222222222222222222222222222222222222222222222222222222222222222222222222222222222222222222222222222222222222222222222222222222222222222222222222222222222222212222222222222222221222222222222222211111111122222222222122222222222222221212112222212222222222222222222222222222222222212222222222222222222222222222222222222222222222222222222222222222222222222222222222222222222222222222222222212222222111122122222222222222222222222222222222222222222222222222222222222222222222222222222222222222

>1II0_B

MQFLQNIPPYLFFTGKGGVGKTSISCATAIRLAEQGKRVLLVSTDPASNVGQVFSQTIGNTIQAIASVPGLSALEIDPQAAAQQYRARIVDPIKGVLPDDVVSSINEQLSGACTTEIAAFDEFTGLLTDASLLTRFDHIIFDTAPTGHTIRLLQLPGAWSSFIDSNPEGASCLGPMAGLEKQREQYAYAVEALSDPKRTRLVLVARLqksTLQEVARTHLELAAIGLKNQYLVINGVLPKTEAANDTLAAAIWErEQEALANLPADLAGLPTDTLFLQPVNMVGVSALSRLLSTQPVASPSSDEYLQQRPDIPSLSALVDDIARNEHGLIMLMGKggvgkttMAAAIAVRLADMGFDVHLTTsdPAAHLSMTLNGSLNNLQVSRIDPHEETERYRQHVLETKGKELDEAGKRLLEEDLRSPCTEEIAVFQAFSRVIREAGKRFVVMDTAPTGHTLLLLDATGAYHREIAKKMGEKGHFTTPMMLLQDPERTKVLLVTLPETTPVLEAANLQADLERAGIHPWGWIInnSLSIADTRSPLLRMRAQQELPQIESVKRQHASRVALVPVLASEPTGIDKLKQLAGHHHHHH

2222222222222222222222222222222222222222222222222222222222222222222222222222222222222222222222222222222222222222222222222222222222222222222222222222222222222222222222222222222222222222222222222222222222222221112222222222222222222222222222222222222222222212222222222222222222222222222222222222222222222222222222222222222222222222222222211111112222222222222222222211222222222222222222222222222222222222222222222222222222222222222222222222222222222222222222222222222222222222222222222222222222222222222222222222222222222222222222112222222222222222222222222222222222222222222222222222222222222

>1LHR_B

MEEECRVLSIQSHVVRGYVGNRAATFPLQVLGFEVDAVNSVQFSNHTGYSHWKGQVLNSDELQELYDGLKLNHVNQYDYVLTGYTRDKSFLAMVVDIVQELKQQNPRLVYVCDPvMGdQRNGEGAMyVPDDLLPVYREKVVPVADIITPnQFEAELLTGRKIHSQEEALEVMDMLHSMGPDTVVItsSNLLSPRGSDYlMALGSQRTRAPDGSVVTQRIRMEmhkvDaVfvgtgdLfAAMLLAWTHKHPNNLKVACEKTvSAmHHVlQRTIKCAKAKSGEGVKPSPAQLELRMVQSKKDIESPEIVVQATVL

222222222222222222222222222222222222222222222222222222222222222222222222222222222222222222222222222222222222222222122122222222122222222222222222222221222222222222222222222222222222222221122222222222122222222222222222222222111121211111121222222222222222222222212212221222222222222222222222222222222222222222222222

>1ZFN_D

GSMNDRDFMRYSRQILLDDIALDGQQKLLDSQVLIiglgglGTPAALYLAGAGVGTLVLadDdDVhLsNLQrqILFTTEDIDRPkSQVSQQRLTQLNPDIQLTALQqrlTGEALKDAVARADVVLDctdnMAtRQEINAACVALNTPLITASAVGFGGQLMVLTPPWEQGCYRCLWPDNQEPERNCRTAGVVGPVvGVMGTLQALEAIKLLSGIETPAGELRLFDGKSSQWRSLALRRASGCPVCGGSNADPV

2222222222222222222222222222222222211111122222222222222222211212212122211222222222221222222222222222222222111222222222222222221111221222222222222222222222222222222222222222222222222222222222222221222222222222222222222222222222222222222222222222222222222

>1XNG_B

MQKDYQKLIVYLCDFLEKEVQKRGFKKVVYglsGgldsAVVGVLCQKVFKENAHAllmpSSVsMPENKTDALNLCEKFSIPYTEYSIAPYDAIFSSHFKDASLTRKGNFCARLrMAFlYDYSLKSDSLVIGtSNKSeRMLGYGTLFGdLACAINPIGELFkTEVYELARRLNIPKKILNkppsadlFVGQSdEKDLGYPYSVIDPLLKDIEALFQTKPIDTETLAQLGYDEILVKNITSRIQKNAFKLELPAIAKRFNPELEHHHHHH

2222222222222222222222222222221112111122222222222222222111122212222222222222222222222222222222222222222222222222212221222222222222212222122222222221222222222222122222222222222222211111112222212222222222222222222222222222222222222222222222222222222222222222222222222222

>2QXL_B

STPFGLDLgnnnSVLAVARNRGIDIVVNEVSNRSTPSVVGFGPKNRYLGETGKNKQTSNIKNTVANLkRIIGLDYHHPDFEQESKHFTSKLVELDDKKTGAEVRFAGEKHVFSATQLAAMFIDKVKDTVKQDTKANITDVCIAVPPWYTEEQRYNIADAARIAGLNPVRIVNdVTAAGVSYGIFKTDLPEGEEKPRIVAFVdIghssYTCSIMAFKKGQLKVLGTACDKHFGgRDFdLAITEHFADEFKTKYKIDIRENPKAYNRILTAAeKLkkVLsANTNAPFSVESVMNDVDVSSQLSREELEELVKPLLERVTEPVTKALAQAKLSAEEVDFVEIIggtTriPTLKQSISEAFGKPLSTTLNqdEAIAKGAAFICAIHSPTLRVRPFKFEDIHPYSVSYSWDKQVEDEDHMEVFPAGSSFPSTKLITLNRTGDFSMAASYTDITQLPPNTPEQIANWEITGVQLPEGQDSVPVKLKLRCDPSGLHTIEEAYTIEDIEVEEPIPLPEDAPEDAEQEFKKVTKTVKKDDLTIVAHTFGLDAKKLNELIEKENEMLAQDKLVAETEDRKNTLEEYIYTLRGKLEEEYAPFASDAEKTKLQGMLNKAEEWLYDEGFDSIKAKYIAKYEELASLGNIIRGRYLAKEEEKKQAIRSKQEA

2222222211112222222222222222222222222222222222222222222222222222222122222222222222222222222222222222222222222222222222222222222222222222222222222222222222222222222222222222122222222222222222222222222221211112222222222222222222222222122212222222222222222222222222222222221221122122222222222222222222222222222222222222222222222222222222222222111211222222222222222222221122222222222222222222222222222222222222222222222222222222222222222222222222222222222222222222222222222222222222222222222222222222222222222222222222222222222222222222222222222222222222222222222222222222222222222222222222222222222222222222222222222222222222222222222222222222222222222222222222

>1V1B_D

MLEVVTAGEPLVALVPQEPGHLRGKRLLEVYVGGaEVNVAVALARLGVKVGFVGRVGEDELGAMVEERLRAEGVDLTHFRRAPGFTGLYLREYLPLGQGRVFYYRKGSAGSALAPGAFDPDYLEGVRFLHLSGITPALSPEARAFSLWAMEEAKRRGVRVSLDVnYrQTLWSPEEARGFLERALPGVDLLFLsEeEAELLFGRVEEALRALSAPEVVLkRgaKgAWAFVDGRRVEGSaFAvEaVDpvgagdAfAAGYLAGAVWGLPVEERLRLAnLLGASVAASRGDHEGAPYREDLEVLLKATQTFMR

222222222222222222222222222222222212222222222222222222222222222222222222222222222222222222222222222222222222222222222222222222222222222222222222222222222222222222221212222222222222222222222222121222222222222222222222221211212222222222222122121221111112122222222222222222222212222222222222222222222222222222222

>2QB8_A

MSPLRKTVPEFLAHLKSLPISKIASNDVLTICVGNESAdMDSIASAITYSYCQYIYNEGTYSEEKKKGSFIVPIIDIPREDLSLRRDVMYVLEKLKIKEEELFFIEDLKSLKQNVSQGTELNSYLVDNNDTPKNLKNYIDNVVGIIDHhFDLQKHLDAEPRIVKVSGSCSSLVFNYWYEKLQGDREVVMNIAPLLMGAILIDTSNMRRkVEESDKLAIERCQAVLSGAVNEVSAQGLEDSSEFYKEIKSRkNDIKGFSVSDILKKdYkQFNFQGKGHKGLEIGLSsIVKRMSWLFNEHGGEADFVNQCRRFQAERGLDVLVLlTsWRKAGDSHrELVILGDSNVVRELIERVSDKLQLQLFGGNLDGGVAMFKQLNVEAtrkQVVPYLEEAYSNLEE

2222222222222222222222222222222222222212222222222222222222222222222222222222222222222222222222222222222222222222222222222222222222222222222222222222122222222222222222222222222222222222222222222222222222222222122222222222222222222222222222222222222222122222222222222121222222222222222221222222222222222222222222222222222222121222222221222222222222222222222222222222222222222222222111222222222222222

>1B0U_A

MMSENKLHVIDLHKRyGGhEvLKGVSLQARAGDVISIIGssgsgkstFLRCINFLeKPSEGAIIVNGQNINLVRDKDGQLKVADKNQLRLLRTRLTMVFqHFNLWSHMTVLENVMEAPIQVLGLSKHDARERALKYLAKVGIDERAQGKYPVHLSGGQQQRVSIARALAMEPDVLLFDePTSALDPELVGEVLRIMQQLAEEGKTMVVVThEMGFARHVSSHVIFLHQGKIEEEGDPEQVFGNPQSPRLQQFLKGSLKKLEH

2222222222222221221212222222222222222221111111122222222122222222222222222222222222222222222222222221222222222222222222222222222222222222222222222222222222222222222222222222222222122222222222222222222222222222221222222222222222222222222222222222222222222222222222

>2HS0_A

MKLRYLNILKEKLGREPTFVELQAFSVMWSEHCGYSHTKKYIRRLPKTGFEGNAGVVNLDDYYSVAFKIESHNHPSAIEPYNGAATGVGGIIRDVLAMGARPTAIfdSlHMSRIIDGIIEGIADYGNSIGVPtvggelrISSLYAHNPLVNVLAAGVVRNDMLVDSKASRPGQVIVIFGGATGRDGIHGASFASEDLTGDKATKLSIQVGDPFAEKMLIEAFLEMVEEGLVEGAQDLGAGGVLSATSELVAKGNLGAIVHLDRVPLREPDMEPWEILISESQERMAVVTSPQKASRILEIARKHLLFGDVVAEVIEEPVYRVMYRNDLVMEVPVQLLANAPEEDIVEYTPGKIPEFKRVEFEEvNaREVfEQYDHMVGTDTVVPpgFgAAVMRIKRDGGYSLvthSRADLALQDTYWGTLIAVLESVRkTLSVGAEPLAITNCVNYGDPDVDPVGLSAMMTALKNACEFSGVPVASGNASLYNTYQGKPIPPTLVVGMLGKVNPQKVAKPKPSKVFAVGWNDFELEREKELWRAIRKLSEEGAFILSssqLLtRthVETFREYGLKIEVKLPEVRPAHQMVLVFSERTPVVDVPVKEIGTLSR

222222222222222222222222222222222222222222222222222222222222222222222222222222222222222222222222222222222112122222222222222222222222111111122222222222222222222222222222222222222222222222222222222222222222222222222222222222222222222222222222222222222222222222222222222222222222222222222222222222222222222222222222222222222222222222222222222222222222222222222222222121222122222222222222112122222222222222111222222222222222222222221222222222222222222222222222222222222222222222222222222222222222222222222222222222222222222222222222222222222222222222211122121122222222222222222222222222222222222222222222222

>1HP1_A

YEQDKTYKITVLHTNDHHGHFWRNEYGEYGLAAQKTLVDGIRKEVAAEGGSVLLLSGGDINTGVPESDLQDAEPDFRGMNLVGYDAMAIGNHEFDNPLTVLRQQEKWAKFPLLSANIYQKSTGERLFKPWALFKRQDLKIAVIGLTTDDTAKIGNPEYFTDIEFRKPADEAKLVIQELQQTEKPDIIIAATHMGHYDNGEHGSNAPGDVEMARALPAGSLAMIVGGHSQDPVCMAAENKKQVDYVPGTPCKPDQQNGIWIVQAHEWGKYVGRADFEFRNGEMKMVNYQLIPVNLKKKRVLYTPEIAENQQMISLLSPFQNKGKAQLEVKIGETNGRLEGDRDKVRFVQTnMGRlILAAQMDRTGADFAVMsGggIrDSIEAGDISYKNVLKVQPfGnVVVYADMTGKEVIDYLTAVAQMKPDSgaYPQFANVSFVAKDGKLNDLKIKGEPVDPAKTYRMATLNfNATGGdGYPRLDNKPGYVNTGFIDAEVLKAYIQKSSPLDVSVYEPKGEVSWQ

222222222222222222222222222222222222222222222222222222222222222222222222222222222222222222222222222222222222222222222222222222222222222222222222222222222222222222222222222222222222222222222222222222222222222222222222222222222222222222222222222222222222222222222222222222222222222222222222222222222222222222222222222222222222222222222222222222222222212221222222222222222212112122222222222222222212122222222222222222222222222112222222222222222222222222222222222222212222212222222222222222222222222222222222222222222222

>2P09_A

MDYKDDDDKKTNWLKRIYRVRPCVKCKVAPRDWkVkNkHLRIyNmcktcFNnSIDIGDDTYHghvdWLMYADSKEISN

222222222222222222222222222222222121212222121111122122222222221111222222222222

>1MIW_B

MKPPFQEALGIIQQLKQHGYDAYFVggAVrdLLLGrPIgdVdIATSALPEDVMAIFPKTIDVGSKHGTVVVVHKGKAYEVTTFKTdGDYEDYRRPESVTFVRSLEEDLKrrdFTMnAIAMDEYGTIIDPFGGREAIRRRIIRTVGEAEKRFREdAlrMMrAVrfVSeLGFALAPDTEQAIVQNAPLLAHISVErMTMEMEkLLGGPFAARALPLLAETGLNAYLPGLAGKEKQLRLAAAYRWPWLAAREERWALLCHALGVQESRPFLRAWKLPNKVVDEAGAILTALADIPRPEAWTNEQLFSAGLERALSVETVRAAFTGAPPGPWHEKLRRRFASLPIKTKGELAVNGKDVIEWVGKPAGPWVKEALDAIWRAVVNGEVENEKERIYAWLMERNRTREKNC

22222222222222222222222221122112222122112122222222222222222222222222222222222222222221222222222222222222222221112221222222222222222222222222222222222222212112212211221222222222222222222222222221222222122222222222222222222222222222222222222222222222222222222222222222222222222222222222222222222222222222222222222222222222222222222222222222222222222222222222222222222222222222222222222222222222222222222222

>1TIL_A

MRNEMHLQFSARSENESFARVTVAAFVAQLDPTMDELTEIKTVVSeAVTnaIIhgYNNDPNGIVSISVIIEDGVVHLTVRdEGvgiPDIEEaRQPLfttkPELErsgmgfTIMENFMDEVIVESEVNKGtTvYLKKHIVKSKALSN

22222222222222222222222222222222222222222222212221122112222222222222222222222222122111222221222211112222111111222222222222222222212122222222222222

>1HI1_C

PRRAPAFPLSDIKAQMLFANNIKAQQASKRSFKEGAIETYEGLLSVDPRFLSFKNELSRYLTDHFPANVDEYGRVYGNGVRTNFFGMRHMNGFPMIPATWPLASNLKKRADADLADGPVSERDNLLFRAAVRLMFSDLEPVPLKIRKGSSTCIPYFSNDMGTKIEIAERALEKAEEAGNLMLQGKFDDAYQLHQMGGAYYVVYRAqStDAITLDPKTGKFVSkDrMVADFEYAVTGGEQGSLFAASKDASRLKEQYGIDVPDGFFCErRrTAMGGPFALNAPIMAVAQPVRNKIYSKYAYTFHHTTRLNKEEKVKEWSLCVATdvsdhDTFWPGWLRDLICDELLNMGYAPWWVKLFETSLKLPVYVGAPAPEQGHTLLGDPSNPDLEVGLSSGQGATDLMGTLLMSITYLVMQLDHTAPHLNSRIKDMPSACRFLDSYWQGHEEIRQISKSdDAMLGWTKGRALVGGHRLFEMLKEGKVNPSPYMKISYEHGGAFLGDILLYDSRREPGSAIFVGNINsMLnnQFSPEYGVQSGVRDRSKRKRPFPGLAWASMKDTYGACPIYSDVLEAIERCWWNAFGESYRAYREDMLKRDTLELSRYVASMARQAGLAELTPIDLEVLADPNKLQyKWTEADVSANIHEVLMHGVSVEKTERFLRSVMPR

2222222222222222222222222222222222222222222222222222222222222222222222222222222222222222222222222222222222222222222222222222222222222222222222222222222222222222222222222222222222222222222222222222222222222121222222222222221212222222222222222222222222222222222222222221212222222222222222222222222222222222222222222222222222211111222222222222222222222222222222222222222222222222222222222222222222222222222222222222222222222222222222222222222222222222222212222222222222222222222222222222222222222222222222222222222222222221221122222222222222222222222222222222222222222222222222222222222222222222222222222222222222222222222222222222212222222222222222222222222222222222

>1R0X_A

STTGIIMENVTAFwEEGFGELleKVQQSNGDRKHSSDENNVSfSHlCLVGNPvLKNINLNIEKGEMLAITGStgsgktsLLMLILGELEASEGIIKHSGRVSFCSqFSWIMPGTIKENIIFGVSYDEYRYKSVVKACQLQQDITKFAEQDNTVLGEGGVTLSGGQRARISLARAVYKDADLYLLDsPFGYLDVFTEEQVFESCVCKLMANKTRILVTSKMEHLRKADKILILHQGSSYFYGTFSELQSLRPDFSSKLMGYDTFDQFTEERRsSILTETLRRFSVDD

2222222222222122222221122222222222222222221221222222122222222222222222221111111222222222222222222222222221222222222222222222222222222222222222222222222222222222222222222222222222222222212222222222222222222222222222222222222222222222222222222222222222222222222222222222222122222222222222

>2J3M_A

MKQSKMLIPTLREVPNDAEVLSHQILLRAGYIRQVAAGIYSYLPLANRVLEKLKTIMREEFEKIDAVEMLMPALLPAELWKESGRYETYGPNLYRLKDRNDRDYILGPTHEETFTELIRDEINSYKRLPLNLYQIQTKYrDeKRSRSGllrgREfimKDGYSFHADEASLDQSYRDYEKAYSRIFERCGLEFRAIIGDGGAMGGKDSKEFMAISEIGedTICYSTESDYAANLEMATSLYTPKKSHETQLDLEKIATPEVGTIAEVANFFEVEPQRIIKSVLFIADEEPVMVLVRGDHDVNDVKLKNFLGADFLDEATEEDARRVLGAGFGSIGPVNVSEDVKIYADLAVQDLANAIVGANEDGYHLTNVNPDRDFQPISYEDLRFVQEGDPSPDGNGVLAFTKGIEIGHIFKLGTRYSDAMGATVLDENGREKSVIMGCYGIGVSRLLSAIVEQNADERGINWPTGIAPFDLHVVQMNVKDEYQTKLSQEVEAMMTEAGYEVLVDDRNERAGVKFADADLIGCPIRITVGKKAVDGVVEVKIKRTGEMLEVRKEELESTLSILMNTTSEVE

22222222222222222222222222222222222222222222222222222222222222222222222222222222222222222222222222222222222222222222222222222222222222222221212222221111221112222222222222222222222222222222222222222222222222222222222221122222222222222222222222222222222222222222222222222222222222222222222222222222222222222222222222222222222222222222222222222222222222222222222222222222222222222222222222222222222222222222222222222222222222222222222222222222222222222222222222222222222222222222222222222222222222222222222222222222222222222222222222222222222222222222222222222222222222222222

>2A84_A

MAIPAFHPGELNVYSAPGDVADVSRALRLTGRRVMLVptmgALhEghLAlVRAAKRVPGSVVVVSIFVNPMQFGAGGDLDAYPRTPDDDLAQLRAEGVEIAFTPTTAAMYPDGLRTTVQPGPLAAELEGGPRPTHFAGVLTVVLKLLQIVRPDRVffgekdYQqLVLIRQLVADFNLDVAVVGvptvREADGLAmssrNRyLDPAQRAAAVALSAALTAAAHAATAGAQAALDAARAVLDAAPGVAVDYLELRDIGLGPMPLNGSGRLLVAARLGTTRLLDNIAIEIGTFAGTDRPDGYR

222222222222222222222222222222222222211112212112212222222222222222222222222222222222222222222222222222222222222222222222222222222222222222222222222222222221111112212222222222222222222111122222221111221222222222222222222222222222222222222222222222222222222222222222222222222222222222222222222222222222

>1XEX_A

MPYIEKLELKGFksYGNKKVVIPFSKGFTAIVGAngSgksnIGDAILFVLGGLSAKAMrASRISDLIfaGSKNEPPAKYAEVAIYFNNEDRGFPIDEDEVVIRRRVYPDGRSSYWLNGRRATRSEILDILTAAMISPDGYNIVLqGDITKFIKMSPLERRLLIDDISGIAEYDSKKEKALEE

22222222222211222222222222222222221121111222222222222222221222222221122222222222222222222222222222222222222222222222222222222222222222222222222212222222222222222222222222222222222222

>1R8B_A

MKVEEILEKALELVIPDEEEVRKGREAEEELRRRLDELGVEYVFvgsYArNTwlKGsLeIdVfLLFPEEFSKEELRERGLEIGKAVLDSYEIRYaeHPyVHGVVKGVEVDVvPCYKLKEPKNIKSavDRtPFhHKWLEGRIKGKENEVRLLkGFLKANGIyGAEYKVRGFsgyLCeLLIVFYGSFLETVKNARRWTRRTVIDVAKGEVRKGEEFFVVDPVDEKRNVAANLSLDNLARFVHLCREFMEAPSLGFFKPKHPLEIEPERLRKIVEERGTAVFAVKFRKPDIVDDNLYPQLERASRKIFEFLERENFMPLRSAFKASEEFCYLLFECQIKEISRVFRRMGPQFEDERNVKKFLSRNRAFRPFIENGRWWAFEMRKFTTPEEGVRSYASTHWHTLGKNVGESIREYFEIISGEKLFKEPVTAELCEMMGVKD

22222222222222222222222222222222222222222222111221221122121212122222222222222222222222222222221122122222222222212222222222222112212212222222222222222221222222221222222222111221222222222222222222222222222222222222222222222222222222222222222222222222222222222222222222222222222222222222222222222222222222222222222222222222222222222222222222222222222222222222222222222222222222222222222222222222222222222222222222222222222222222222222222222

>1PK8_H

GSNYLRRRLSDSNFMANLPNGYMTDLQRPQPPPPPPSAASPGATPGSAAASAERASTAAPVASPAAPSPGSSGGGGFFSSLSNAVKQTTAAAAATFSEQVGGGSGGAGRGGAAARVLLVIDEPHTDWAKYFKGKKIHGEIDIKVEQAEFSDLNLVAHANGGFSVDMEVLRNGVKVVRSLKPDFVLIRQhAFSMARNGDYRSLVIGLQYAGIPSVNSLHSVYNFCDkPWVFAQMVRLHKKLGTEEFPLiDQTFYPNHKEMLSSTTYPVvVkMGHAhsgMGkVKVDNQHDFQDIASVVALTKTYATAepfIDaKYdVRVQKIGQNYKAYMrTSVSGNwkTNTGSAMLEQIAMSDRYKLWVDTCSEIFGGLDICAVeAlHGKDGRDHIieVvGSSMpLIGDHQDEDKQLIVELVVNKMTQALPRQ

22222222222222222222222222222222222222222222222222222222222222222222222222222222222222222222222222222222222222222222222222222222222222222222222222222222222222222222222222222222222222222222122222222222222222222222222222222222212222222222222222222221222222222222222222212122221112212222222222222222222222222111221221222222222222221222222112222222222222222222222222222222222221212222222221121222212222222222222222222222222222

>1XEX_B

EKEKKNVFMRTFEAISRNFSEIFAKLSPGGSARLILENPEDPFSGGLEIEAKPAGKDVKRIEAMSGGEKALTALAFVFAIQKFKPAPFYLFDqIDAHLDDANVKRVADLIKESSKESQFIVITlRDVMMANADKIIGVSmRDGVSKVVSLSLEKAMKILEEIRKKQGWEHGN

2222222222222222222222222222222222222222222222222222222222222222222222222222222222222222222212222222222222222222222222222221222222222222222122222222222222222222222222222222

>1MV5_D

MLSARHVDFAYdDseQILRDISFEAQPNSIIAFAGPSGGGKSTIFSLLERFYQPTAGEITIDGQPIDNISLENWRSQIGFVSQDSAIMAGTIRENLTYGLEGDYTDEDLWQVLDLAFARSFVENMPDQLNTEVGERGVKISGGQRQRLAIARAFLRNPKILMLDEATASLDSESESMVQKALDSLMKGRTTLVIAHRLSTIVDADKIYFIEKGQITGSGKHNELVATHPLYAKYVSEQLTVGQ

222222222221211222222222222222222222222222222222222222222222222222222222222222222222222222222222222222222222222222222222222222222222222222222222222222222222222222222222222222222222222222222222222222222222222222222222222222222222222222222222222

>1Y8Q_D

MALSRGLPRELAEAVAGGRVLVvgAggiGCELLKNLVLTGFSHIDLidldTIdVsnLNrqFLFQKKHVGRSkAQVAKESVLQFYPKANIVAYHDsimNPDYNVEFFRQFILVMNAldnRAaRNHVNRMCLAADVPLIESGTAGYLGQVTTIKKGVTECYECHPKPTQRTFPGATIRNTPSEPIHCIVWAKYLFNQLFGEEDADQEVSPDRADPEAAWEPTEAEARARASNEDGDIKRISTKEWAKSTGYDPVKLFTKLFKDDIRYLLTMDKLWRKRKPPVPLDWAEVQSQGEETNASDQQNEPQLGLKDQQVLDVKSYARLFSKSIETLRVHLAEKGDGAELIWDkdDPSAMDFVTSAANLRMHIFSMNMKSRFDIKSMAGNiIPAIATTnAVIAGLIVLEGLKILSGKIDQCRTIFLNKQPNPRKKLLVPCALDPPNPNCYVCASKPEVTVRLNVHKVTVLTLQDKIVKEKFAMVAPDVQIEDGKGTILISSEEGETEANNHKKLSEFGIRNGSRLQADDFLQDYTLLINILHSEDLGKDVEFEVVGDAPEKVGPKQAEDAAKSITNGSDDGAQPSTSTAQEQDDVLIVDSDEEDSSNNADVSEEERSRKRKLDEKENLSAKRSRIEQKEELDDVIALD

2222222222222222222222112111222222222222222222111122121122112222222222212222222222222222222222111222222222222222222111221222222222222222222222222222222222222222222222222222222222222222222222222222222222222222222222222222222222222222222222222222222222222222222222222222222222222222222222222222222222222222222222222222222222222222222222222222222221122222222222222222222222222222222222122222221222222222222222222222222222222222222222222222222222222222222222222222222222222222222222222222222222222222222222222222222222222222222222222222222222222222222222222222222222222222222222222222222222222222222222222222222222222222222222222222222222222222

>1YP3_A

MAVSDSQNSQTCLDPDASRSVLGIIlGggAGTrLYPLTKKRAkPAVPLGANYRLIDIPVSNCLNSNISKIYVlTqFNSASLNRHLSRAYASNMGGYKNEGFVEVLAAQQSPENPDWFqgtADaVRQYLWLFEEHTVLEYLILagdhLYRMDYEKFIQAHRETDADITVAALPMDEKRATAFGLMKIDEEGRIIEFAeKPQGEQLQAMKVDTTILGLDDKRAKEMPFIAsMgIyVISKDVMLNLLRDKFPGANdfgsEVIPGATSLGMRVQAYLYDGYWEdIGTIEAFYNANLGITKKPVPDFSFYDRSAPIYTQPRYLPPSKMLDADVTDSVIGEGCVIKNCKIHHSVVGLRSCISEGAIIEDSLLMGADYYETDADRKLLAAKGSVPIGIGKNCHIKRAIIDKNARIGDNVKIINKDNVQEAARETDGYFIKSGIVTVIKDALIPSGIII

2222222222222222222222222121122212222222221222222222222222222222222222221212222222222222222222222222222222222222222221112212222222222222222222111122222222222222222222222222222222222222222222222222122222222222222222222222222222221212122222222222222222221111222222222222222222222221222222222222222222222222222222222222222222222222222222222222222222222222222222222222222222222222222222222222222222222222222222222222222222222222222222222222222222222222222

>3C9R_A

MGSHHHHHHDITSLYKKAGSAAAVLEENLYFQGSFTMRLKELGEFGLIDLIKKTLESKVIGDDTAPVEYCSKKLLLTTDVLNEGVHFLRSYIPEAVGWKAISVNVSDVIANGGLPKWALiSlNLPEDLEVSYVERFyIGVKRACEFYKCEvVggniSKSEKIGISVFLVGETERFVGRDGARLGDSVFVSGTLGDSRAGLELLLMEKEEYEPFELALIQRHLRPTARIDYVKHIQKYANASMDISDGLVADANHLAQRSGVKIEILSEKLPLSNELKMYCEKYGKNPIEYALFGGEDYQLLFTHPKERWNPFLDMTEIGRVEEGEGVFVDGKKVEPKGWKHF

222222222222222222222222222222222222222222222222222222222222222222222222222222222222222222222222222222222222222222222221212222222222222212222222222222121111222222222222222222222222222222222222222222222222222222222222222222222222222222222222222222222222222222222222222222222222222222222222222222222222222222222222222222222222222222222222222222

>2B6F_A

GAPEGPGPSGGAQGGSIHSGRIAAVHNVPLSVLIRPLPSVLDPAkVQslVDtIREDPDsvpPiDVLWIKGAQGGDYFYSFggchrYAAYQQLQRETIPAKLVQSTLSDLRVYLGASTPDLQ

2222222222222222222222222222222222222222222212211221222222111212222222222222222211111222222222222222222222222222222222222

>2JAX_A

MSSGNSSLGIIVGIDDSPAAQVAVRWAARDAELRKIPLTLVHAVSPEVATWLEVPLPPGVLRWQQDHGRHLIDDALKVVEQASLRAGPPTVHSEIVPAAAVPTLVDMSKDAVLMVVGCLGSGRWPGRLLGSVSSGLLRHAHCPVVIIHDEDSVMPHPQQAPVLVgvdGSSAsELATAIAFDEASRRNVDLVAlHaWSdVDVSEWPGIDWPATQSMAEQVLAERLAGWQERYPNVAITRVVVRDQpARQlVQRSEEAQLVVvgSrgrggyAGmLVgsvGETVAQLARTPVIVARESLTLEHHHHHH

22222222222222222222222222222222222222222222222222222222222222222222222222222222222222222222222222222222222222222222222222222222222222222222222222222222222222222222111222212222222222222222222212122122222222222222222222222222222222222222222222221222122222222222112111111221221112222222222222222222222222222

>2O0H_A

MGSSHHHHHHSSGLVPRGSHMLEDPMEQPINVLNDFHPLNEAGKILIKHPSLAERKDEDGIHWIKSQWDGKWYPEKFSDYLRLHKIVKIPNNSDKPELFQTYKDKNNKRSRYMGLPNLKRANIKTQWTREMVEEWKKCRDDIVYFAETYCAitHIDYGVIKvqLrDYqRDMLKIMSSKRMTVCNLSrqlgkttVVAIFLAHFVCFNKDKAVGILAHKGSMSAEVLDrTKQAIELLPDFLQPGIVEWNKGSIELDNGSSIGAYASSPDAVRGNSFAMIYIEDCAFIPNFHDSWLAIQPVISSGRRSKIIITTTPNGLNHFYDIWTAAVEGKSGFEPYTAIWNSVKERLYNDEDIFDDGWQWSIQTINGSSLAQFRQEHTAAFEGTS

2222222222222222222222222222222222222222222222222222222222222222222222222222222222222222222222222222222222222222222222222222222222222222222222222222222112222222211212212222222222222222221111111222222222222222222222222222222222122222222222222222222222222222222222222222222222222222222222222222222222222222222222222222222222222222222222222222222222222222222222222222222222222222222222222

>1D9Z_A

VEGRFQLVAPyePqGDqPQAIAKLVDGLRRGVKHQTLLGatgtgktfTISNVIAQVNKPTLVIAHNKTLAGQLYSeLKEFFPHNAVEYFVSYYDYYQPEAYVPQTDTYIEKDAKINDEIDKLRHSATSALFERRDVIIVASVSCIYGLGSPEEYRELVVSLRVGMEIERNALLRRLVDIQYDRNDIDFRGTFRVRGDVVEIFPASRDEHCIRVEFFGDEIERIREVDALTGKVLGEREHVAIFPASHFVTREEKMRLAIQNIEQELEERLAELRAQGKLLEAQRLEQRTRYDLEMMREMGFCSGIENYSRHLALRPPGSTPYTLLDYFPDDFLIIVDeSHVTLPQLRGMYNGDRARKQVLVDHGFRLPSALDNRPLTFEEFEQKINQIIYVSaTPGPYELEHSPGVVEQIIRpTGLLDPTIDVRPTKGQIDDLIGEIRERVERNERTLVTTLTKKMAEDLTDYLKEAGIKVAYLHSEIKTLERIEIIRDLRLGKYDVLVGINLLREGLdIPEVSLVAILDADKEGFLRSERSLIQTIGrAArNANGHVIMYADTITKSMEIAIQETKRRRAIQEEYNRKHGIVPRTVKkEIRDVIRATYAAEETEMYEAKPAAAMTKQEREELIRTLEAEMKEAAKALDFERAAQLRDIIFELKAEG

222222222211212212222222222222222222222111111112222222222222222222222222222122222222222222222222222222222222222222222222222222222222222222222222222222222222222222222222222222222222222222222222222222222222222222222222222222222222222222222222222222222222222222222222222222222222222222222222222222222222222222222222222222222222222222222222212222222222222222222222222222222222222222222222222222221222222222222222222212222222222222222222222222222222222222222222222222222222222222222222222222222222222222222222222212222222222222222222222222222212212222222222222222222222222222222222222222222222122222222222222222222222222222222222222222222222222222222222222222222

>1WKL_B

MERTFVMIkPDGVRRGLVGEILARFERKGFRIAALKLMQISQELAERhyAEhREKPfFPGlVRfITSGPVVAMVLEGPGVVAEVrKMMGATHPKDALPGTIrGDFATTidEnVIhGSaTLEDAQREIALFFRPEELL

22222222122222222222222222222222222222222222222112212222122212212222222222222222222212222222222222222122222211212212212222222222222222222

>1B76_B

AASSLDELVALCKRRGFIFQSSEIYGGLQGVYDYGPLGVELKNNLKQAWWRRNVYERDDMEGLDASVLTHRLVLHYSGHEATFADPMVDNAKARYWTPPRYFNMMFQDLRGPRGGRGLLAYLRPeTAQGIFVNFKNVLDATSRKLGFGIAQIGKAFrNeITPRNFIfrvREfEqMEIEYFVRPGEDEYWHRYWVEERLKWWQEMGLSRENLVPYQQPPESSAHyAKATVdILYRFPHGSleleGIAQRTDFDLGSHTKDQEALGITARVLRNEHSTQRLAYRDPETGKWFVPYVIePsagVdrGVLALLAEAFTREELPNGEERIVLKLKPQLAPIKVAVIPLVKNRPEITEYAKRLKARLLALGLGRVLYEDTGNIGKAYRRHDEVGTPFAVTVDYDTIGQSKDGTTRLKDTVTVRDRDTMEQIRLHVDELEGFLRERLRW

2222222222222222222222222222222222222222222222222222222222222222222222222222222222222222222222222222222222222222222222222222122222222222222222222222222222221212222222111221212222222222222222222222222222222222222222222222222122222122222222211112222222222222222222222222222222222222222222222222222121112112222222222222222222222222222222222222222222222222222222222222222222222222222222222222222222222222222222222222222222222222222222222222222222

>1PHK_A

TRDAALPGSHSTHGFYENYEPKEIlgrgvssVvRRCIHKPTCKEYaVkIIDVTGGGSFSAEEVQELREATLKEVDILRKVSGHPNIiQLKDTYETNTFFFLVfdlmKKgeLFdYLTEKVTLSEKETRKIMRALLEVICALHKLNIVHRdLkPenIlLDDDMNIKLtdFGfSCQLDPGEKLREVCGTPSYLAPEIIECSMNDNHPGYGKEVDMWSTGVIMYTLLAGSPPFWHRKQMLMLRMIMSGNYQFGSPEWDDYSDTVKDLVSRFLVVQPQKRYTAEEALAHPFFQQYVVEEVRHF

2222222222222222222222221111111212222222222221212222222222222222222222222222222222222212222222222222221111221122122222222222222222222222222222222222121211212222222221122122222222222222222222222222222222222222222222222222222222222222222222222222222222222222222222222222222222222222222222222222222222

>2R9V_A

MGSDKIHHHHHHLRINPGEITKVLEEKIKSFEEKIDLEDTGKVIQVGDGIARAYGLNKVMVSELVEFVETGVKGVAFNLEEDNVGIIILGEYKDIKEGHTVRRLKRIIEVPVGEELLGRVVNPLGEPLDGKGPINAKNFRPIEIKAPGVIYRKPVDTPLQTGIKAIDSMIPIGRGQRELIIGDrqtgktaiAIdTIINQKGQGVYCIYVAIGqKKSAIARIIDKLRQYGAMEYTTVVVASASDPASLQYIAPYAGCAMGEYFAYSGRDALVVYDdLSkHAVAYRQLSLLMRRPPGREAYPGDIFYLHSRLLERAVRLNDKLGGGSLTALPIVeTQANDISAYIPTNVISITDGQIYLEPGLfYAGQrpAINVGLSVSRVGGSAQIKAMKQVAGMLRIDLAQYRELETFAQFATELDPATRAQIIRGQRLMELLKqeqySPMPVEEQVVVLFAGVRGYLDDLPVEEVrRFEKEFLRFMHEKHQDILDDIKTKKELTSETEEKLKKAIEEFKTTFRV

22222222222222222222222222222222222222222222222222222222222222222222222222222222222222222222222222222222222222222222222222222222222222222222222222222222222222222222222222222222222222211111111221222222222222222222122222222222222222222222222222222222222222222222222222222222221221222222222222222222222222222222222222222222222222222222122222222222222222222222222221222211222222222222222222222222222222222222222222222222222222222222222222111122222222222222222222222222221222222222222222222222222222222222222222222222222

>1OBD_A

SITKTELDGILPLVargkvrDiYEVDAGTlLfVATDRISAYDVIMENSIPEKGILLTKLSEFWFKFLSNDVRNhLVDIAPGKTIFDYLPAKLSEPKYKTQLEDRSLLvhkhKlIPLEVIVRGYITGSAWKEYVKTGTVHGLKQPQGLKESQEFPEPIFTPSTkAEQGEHDENISPAQAAELVGEDLSRRVAELAVKLYSKCKDYAKEKGIIIADTkFeFGIDEKTNEIILvdEVLTPDSSRFWNGASYKVGESQDSYDKQFLRDWLTANKLNGVNGVKMPQDIVDRTRAKYIEAYETLTGSKWSH

22222222222222111111212222222121222222222222222222222222222222222222222221222222222222222222222222222222222111121222222222222222222222222222222222222222222222222212222222222222222222222222222222222222222222222222222121222222222222112222222222222222222222222222222222222222222222222222222222222222222222222

>1QHG_A

MNFLSEQLLAhlnKEqQEAVRTTEGPLLIMAgagsgktrVLTHRIAYLMAEKHVAPWNILAITFTNKAAREMRErVQSLLGGAAEDVWISTFHSMCVRILRRDIDRIGINRNFSILDPTDQLSVMKTILKEKNIDPKKFEPRTILGTISAAKNELLPPEQFAKRASTYYEKVVSDVYQEYQQRLLRNHSLDFDDLIMTTIQLFDRVPDVLHYYQYKFQYIHIDeYQDTNRAQYTLVKKLAERFQNICAVGDADqSIYRWRGADIQNILSFERDYPNAKVILLEQNyrSTKRILQAANEVIEHNVNRKPKRIWTENPEGKPILYYEAMNEADEAQFVAGRIREAVERGERRYRDFAVLYRTNAQSRVMEEMLLKANIPYQIVGGLKFYDRKEIKDILAYLRVIANPDDDLSLLRIINVPKRGIGASTIDKLVRYAADHELSLFEALGELEMIGLGAKAAGALAAFRSQLEQWTQLQEYVSVTELVEEVLDKSGYREMLKAERTIEAQSRLENLDEFLSVTKHFENVSDDKSLIAFLTDLALISDLDELDGTEQAAEGDAVMLMTLHAAkgLeFPVVFLIGMEEGIFPHNRSLEDDDEMEEERRLAYVGITrAEEELVLTSAQMRTLFGNIQMDPPSRFLNEIPAHLLETASRRQAGASRPAVSRPQASGAVGSWKVGDRANHRKWGIGTVVSVRGGGDDQELDIAFPSPIGIKRLLAKFAPIEKV

2222222222111221222222222222222111111112222222222222222222222222222222222212222222222222222222222222222222222222222222222222222222222222222222222222222222222222222222222222222222222222222222222222222222222222222222222222222122222222222222222222222222222122222222222222222222222222222221122222222222222222222222222222222222222222222222222222222222222222222222222222222222222222222222222222222222222222222222222222222222222222222222222222222222222222222222222222222222222222222222222222222222222222222222222222222222222222222222222222222222222222222222222222222222222221121222222222222222222222222222222222222221222222222222222222222222222222222222222222222222222222222222222222222222222222222222222222222222222222222222222222

>2F02_B

MSLIVTVTMNPSIDISYLLDHLKLDTVNRTSQVTKTPGGkGLNVTRVIHDLGGDVIATGVLGGFHGAFIANELKKANIPQAFTSIKEETRDSIAILHEGNQTEILEAGPTVSPEEISNFLENFDQLIKQAEIVTISGSLAKGLPSDFYQELVQKAHAQEVKVLLDTSGDSLRQVLQGPWKPYLIkPnLEELEGLLGQDFSENPLAAVQTALTKPMFAGIEWIVIsLGkDgAIAKHHDQFYRVKipTiQaKNpvgsgdAtIAGLAYGLAKDAPAAELLKWGMAAgMANaQERMTGHVDVENVKKHLMNIQVVEIAKEGHHHHHH

22222222222222222222222222222222222222212222222222222222222222222222222222222222222222222222222222222222222222222222222222222222222222222222222222222222222222222222222222222222222222221212222222222222222222222222222222222222122121222222222222211212122111111212222222222222222222222221222122222222222222222222222222222222222

>1E4G_T

MIDLSKTVFYTSIDIgsryIkGLVLGKRDQEWEALAFSSVKSRGLDeGEIKDAIAFKESVNTLLKELEEQLQKSLRSDFVISFsSVSFEREDTVIERDFGEEKRSITLDILSEMQSEALEKLKENGKTPLHIFSKRYLLDDERIVFNPLDMKASKIAIEYTSIVVPLKVYEMFYNFLQDTVKSPFQLKSsLVSTAEGVLTTPEKDRGVVVVnlgynfTGLIAYKNGVPIKISYVPVGmKHViKDVSAVLDTSFEESeRLiiTHGNAVYNDLKEEEIQYRGLDGNTIKTTTAKKLSVIIHARLREIMSKSKKFFREVEAKIVEEGEIGIPGGVVLTgggAkipRINELATEVFKSPVRTGCYANSDRPSIINADEVANDPsFAAAFGNVFAVSENPYEETPVKSENPLKKIFRLFKELME

22222222222222211112122222222222222222222222221222222222222222222222222222222222222122222222222222222222222222222222222222222222222222222222222222222222222222222222222222222222222222222222212222222222222222222221111112222222222222222222212221222222222222221221122222222222222222222222222222222222222222222222222222222222222222222222222111211122222222222222222222222222222222222221222222222222222222222222222222222222222

>2HVY_B

MEKQGEKMKRLGKVLHYAkqGFLIVRTNWVPSLNDRVVDKRLQFVGIVKDVFGPVKMPYVAIKPKVSNPEIYVGEVLYVDERKRKESPKKNKEKRMKKKKRLNR

22222222222222222211222222222222222222222222222222222222222222222222222222222222222222222222222222222222

>1KMN_A

MAKNIQAIRGMNDYLPGETAIWQRIEGTLKNVLGSYGYSEIRLPIVEQTPLFKRAIGEVTDVVEKEMYTFEDRNGDSLTLRPEGTAGCVRAGIEHGLLYNQEQRLWYIGPMFrHeRPQKgryRQfHqLGCEVFGLQGPDIDAELIMLTARWWRALGISEHVTLELNSIGSLEARANYRDALVAFLEQHKEKLDEDCKRRMYTNPLRVLDSKNPEVQALLNDAPALGDYLDEESREHFAGLCKLLESAGIAYTVNQRLVrglDYYNRTVFEWVTNSLGSqGtvCaGGRYDGLVEQLGGRATPAVGFaMgLerLVLLVQAVNPEFKADPVVDIYLVASGADTQSAAMALAERLRDELPGVKLMTNHGGGNFKKQFARADKWGARVAVVLGESEVANGTAVVKDLRSGEQTAVAQDSVAAHLRTLLG

2222222222222222222222222222222222222222222222222222222222222222222222222222222222222222222222222222222222222222121222211122121222222222222222222222222222222222222222222222222222222222222222222222222222222222222222222222222222222222222222222222222222222222221112222222222222222212112122222222222222222222212121122222222222222222222222222222222222222222222222222222222222222222222222222222222222222222222222222222222222222222

>2Q0D_A

MGSSHHHHHHSSGLVPRGSHMPPSPAVVGRSLVNSFKQFVSKDLHTRHVDATYRLVLDCVAAVDPLMRLYTfgsTVvYGVHEKGsdVdFVVLNKTDVEDGKGGDAATQVAKGLQADILAKLARVIRQKHLSWNVEEVRRTrVPVVRVKGGGAVDFDITAYRRNgVRnsALLRAYFEQNPPCRWLSMSIkRWSkQTGLNASVIGGSItsyGFnLMVVYYLLQRNHLQFVPPSTIDVSRVEPLPPHLPLEEPADEGLELGTQVLDFLHFFLHEFDSDKQVISLNRPGITTKEELDWTKSAEDFARMNGEKVHYQWCIEdPYeLNlNvGrnVTPLKRDFLRRHLEKARDTALLTIV

22222222222222222222222222222222222222222222222222222222222222222222222111221222222211212222222222222222222222222222222222222222222222222222122222222222222222222221221122222222222222222222122212222222222222111221222222222222222222222222222222222222222222222222222222222222222222222222222222222222222222222222222222221221221212112222222222222222222222222

>2DDO_A

MSSLLLFNDKSRALQADIVAVQSQVVYGSVGNSIAVPAIKQNGLNVFAVPTVLLSNTPHYDTFYGGAIPDEWFSGYLRALQERDALRQLRAVTTGYMGTASQIKILAEWLTALRKDHPDLLIMVdPvIgdIDSGIyVKPDLPEAYRQYLLPLAQGItPnIFeLEILTGKNCRDLDSAIAAAKSLLSDTLKWVVVtsASGNEENQEmQvVVVTADSVNVIShsrvKtDlKGtgDLfcAQLISGLLKGKALTDAVHRAgLRvlEVmRYTQQHESDELILPPLAEA

2222222222222222222222222222222222222222222222222222222222222222222222222222222222222222222222222222222222222222222222222222121211222221222222222222222222221212212222222222222222222222222222222211222222222121222222222222111121212211221122222222222222222222122112212222222222222222222

>1F2U_C

MKLERVTVKNFrsHSDTVVEFKEGINLIIGqngsgkssLLDAILVGLYWPLRIKDIKKDeFtkvGARDTYIDLIFEKDGTKYRITRRFLKGYSSGEIHAMKRLVGNEWKHVTEPSSKAISAFMEKLIPYNIFLNAIYIRqGQIDAILES

22222222222112222222222222222211111111222222222222222222222121112222222222222222222222222222222222222222222222222222222222222222222222222221222222222

>1F2U_D

KYKALAREAALSKIGELASEIFAEFTEGkySEVVVRAEENKVRLFVVwEGKERPLTflsggeRIALGLAFRLAMSLYLAGEISLLILDEPTPyLDEERRRKLITIMERYLKKIPQVILVSHDEELKDAADHVIRISLENGSSKVEVVS

2222222222222222222222222222112222222222222222212222222211111122222222222222222222222222222212222222222222222222222222222222222222222222222222222222

>1S9I_B

EAFLTQKAKVGELKDDDFERISElgagnGgVvTKVQHRPSGLIMaRkLIHLEIKPAIRNQIIRELQVLHECNSPYIVGFYGAFYSDGEISICmehmDGgsLdqVLKEAKRIPEEILGKVSIAVLRGLAYLREKHQIMHRdVkPsnIlVNSRGEIKLcdFGVSGQLIDSMANSFVGTRSYMAPERLQGTHYSVQSDIWSMGLSLVELAVGRYPIPPPDAKELEAIFGRPVVDGEEGEPHSISPRPRPPGRPVSGHGMDSRPAMAIFELLDYIVNEPPPKLPNGVFTPDFQEFVNKCLIKNPAERADLKMLTNHTFIKRSEVEEVDFAGWLCKTLRLNQPGTPTRTAVLEHHHHHH

222222222222222222222221111121212222222222221212222222222222222222222222222222222222222222221111221121122222222222222222222222222222222222212121121222222222112222222222222222222222222222222222222222222222222222222222222222222222222222222222222222222222222222222222222222222222222222222222222222222222222222222222222222222222222222222222222222222222222222

>2CG9_B

MASETFEFQAEITQLMSLIINTVYSNKEIFLRelISnaSdaLDKIRYKSLSDPKQLETEPDLFIRITPKPEQKVLEIRdSGigmTKAELINnLGTIAksgTkAFMEALSAGADVSMIgqfgvgfySLFLVADRVQVISKSNDDEQYIWESNAGGSFTVTLDEVNERIGRGtIlRLFLKDDQLEYLEEKRIKEVIKRHSEFVAYPIQLVVTKEVEKEVPIPEEEKKDEEKKDEEKKDEDDKKPKLEEVDEEEEKKPKTKKVKEEVQEIEELNKTKPLWTRNPSDITQEEYNAFYKSISNDWEDPLYVKHFSVEGQLEFRAILFIPKRAPFDLFESKKKKNNIKLYVRRVFITDEAEDLIPEWLSFVKGVVDSEDLPLNLSREMLQQNKIMKVIRKNIVKKLIEAFNEIAEDSEQFEKFYSAFSKNIKLGVHEDTQNRAALAKLLRYNSTKSVDELTSLTDYVTRMPEHQKNIYYITGESLKAVEKSPFLDALKAKNFEVLFLTDPIDEYAFTQLKEFEGKTLVDITKDFELEETDEEKAEREKEIKEYEPLTKALKEILGDQVEKVVVSYKLLDAPAAIRTGQFGWSANMERIMKAQALRDSSMSSYMSSKKTFEISPKSPIIKELKKRVDEGGAQDKTVKDLTKLLYETALLTSGFSLDEPTSFASRINRLISLGLN

22222222222222222222222222222222112211211222222222222222222222222222222222222212211122222221222221112122222222222222211111111222222222222222222222222222222222222222222222121222222222222222222222222222222222222222222222222222222222222222222222222222222222222222222222222222222222222222222222222222222222222222222222222222222222222222222222222222222222222222222222222222222222222222222222222222222222222222222222222222222222222222222222222222222222222222222222222222222222222222222222222222222222222222222222222222222222222222222222222222222222222222222222222222222222222222222222222222222222222222222222222222222222222222222222222222222222222222222222222222222222222222222222222

>1Y8P_A

GGSHHHHHHGMARLENLYFQGKQPVPKQIERYSRFSPSPLSIKQFLDFGRDNACEKTSYMFLRKELPVRLANTMREVNLLPDNLLNRPSVGLVQSWYMQSFLELLEYENKSPEDPQVLDNFLQVLIKVRNRHNDVVPTMAQGVIEYKEKFGFDPFISTNIQYFLDRFYTNRISFRMLINQHTLLFGGDTNPVHPKHIGSIDPTCNVADVVKDAYETAKMLCEQYYLVAPELEVEEFNAKAPDKPIQVVYVPSHLFHMLFelFknsMraTVELYEDRKEGYPAVKTLVTLGKEDLSIKISdLgGgvPLRKIDRlFNYMystAPRPSLEPTRAAPLagfgyglPISRLYARYFQGDLKlYSMEGVGtDAVIYLKALSSESFERLPVFNKSAWRHYKTTPEADDWSNPSSEPRDASKYKAKQ

22222222222222222222222222222222222222222222222222222222222222222222222222222222222222222222222222222222222222222222222222222222222222222222222222222222222222222222222222222222222222222222222222222222222222222222222222222222222222222222222222222222222222222221121112112222222222222222222222222222222121211222222212222111222222222222221111111222222222222222122222221222222222222222222222222222222222222222222222222222222

>3R1R_C

MNQNLLvTkrDGSTerinLDkiHRvLDWAAEGLHNVSISQVELRSHIQFYDGIKtSDihETiIKAAADLISRDAPDYQYLAARLAIfHLRkKAYGQFEPPALYDHVVKMVEMGKYDNHLLEDYTEEEFKQMDTFIDHDRDMTFSYAAVKQLEGKYLVQNRVTGEIYESAQFLYILVAACLFSNYPRETRLQYVKRFYDAVSTFKISLPTPIMSGVRTPTRQFSSCVLIECGDSLDSINATSSAIVKYVSQRAGIGINAGRIRALGSPIRGGEAFHTGCIPFYKHFQTAVKSCSQGGVRGGAATLFYPMWHLEVESLLVLKNNRGVEGNRVRHMDYGVQINKLMYTRLLKGEDITLFSPSDVPGLYDAFFADQEEFERLYTKYEKDDSIRKQRVKAVELFSLMMQERASTGRIYIQNVDHCNTHSPFDPAIAPVRQSNLCLEIALPTKPLNDVNDENGEIALCTLSAFNLGAINNLDELEELAILAVRALDALLDYQDYPIPAAKRGAMGRRTLGIGVINFAYYLAKHGKRYSDGSANNLTHKTFEAIQYYLLKASNELAKEQGACPWFNETTYAKGILPIDTYKKDLDTIANEPLHYDWEALRESIKTHGLRNSTLSALMPSETSSQISNATNGIEPPRGYVSIKASKDGILRQVVPDYEHLHDAYELLWEMPGNDGYLQLVGIMQKFIDQSISANTNYDPSRFPSGKVPMQQLLKDLLTAYKFGVKTLYYQNTRDGAEDAQDDLVPSIQDDGCESGACKI

22222212112222111122112212222222222222222222222222222212211221222222222222222222222222122212222222222222222222222222222222222222222222222222222222222222222222222222222222222222222222222222222222222222222222222222222222222222222222222222222222222222222222222222222222222222222222222222222222222222222222222222222222222222222222222222222222222222222222222222222222222222222222222222222222222222222222222222222222222222222222222222222222222222222222222222222222222222222222222222222222222222222222222222222222222222222222222222222222222222222222222222222222222222222222222222222222222222222222222222222222222222222222222222222222222222222222222222222222222222222222222222222222222222222222222222222222222222222222222222222222222222222222222222222222222222222222222

>2HVY_A

MARDEVRRILPADIKREVLIKDENAETNPDWGFPPEKRPIEMHIQFGVINLDKPPGPTSHEVVAWIKKILNLEKAGHGGTLDPKVSGVLPVALEKATRVVQALLPAGKEYVALMhLhGDVPEDKIIQVMKEFEGEIIQRPPLRSAVKRRLRTRKVYYIEVLEIEGrdVLFRVGVEAGTYIRSLIHHIGLALGVGAhMSELRRTRSGPFKEDETLITLHDLVDYYYFWKEDGIEEYFRKAIQPMEKAVEHLPKVWIKDSAVAAVTHGADLAVPGIAKLHAGIKRGDLVAIMTLKDELVALGKAMMTSQEMLEKTKGIAVDVEKVFMPRDWYPKLWEKRDRSHHHHHH

2222222222222222222222222222222222222222222222222222222222222222222222222222222222222222222222222222222222222222221212222222222222222222222222222222222222222222222221122222222222222222222222222221222222222222222222222222222222222222222222222222222222222222222222222222222222222222222222222222222222222222222222222222222222222222222222222222222222

>1U5R_B

MSYYHHHHHHDYDIPTTENLYFQGAMDPMPAGGRAGSLKDPDVAELFFKDDPEKLFSDLREighgsfGAvYFARDVRNSEVVaIkKMSYSGKQSNEKWQDIIKEVRFLQKLRHPNTiQYRGCYLREHTAWLVmeycLgsASdLLEVHKKPLQEVEIAAVTHGALQGLAYLHSHNMIHRDVkAgnIlLSEPGLVKLGdFGSASIMAPANSFVGTPYWMAPEVILAMDEGQYDGKVDVWSLGITCIELAERKPPLFNMNAMSALYHIAQNESPALQSGHWSEYFRNFVDSCLQKIPQDRPTSEVLLKHRFVLRERPPTVIMDLIQRTKDAVRELDNLQYRKMKKILFQEA

222222222222222222222222222222222222222222222222222222222222211111122122222222222212122222222222222222222222222222221222222222222222111121122122222222222222222222222222222222222222121121222222222212222222222222222222222222222222222222222222222222222222222222222222222222222222222222222222222222222222222222222222222222222222222222222222222222222222

>1W7A_B

MSAIENFDAHTPMMQQYLRLKAQHPEILLFYRMGDFYELFYDDAKRASQLLDISLTKRGASAGEPIPMAGIPYHAVENYLAKLVNQGESVAICEQIGDPATSKGPVERKVVRIVTPGTISDEALLQERQDNLLAAIWQDSKGFGYATLDISSGRFRLSEPADRETMAAELQRTNPAELLYAEDFAEMSLIEGRRGLRRRPLWEFEIDTARQQLNLQFGTRDLVGFGVENAPRGLCAAGCLLQYAKDTQRTTLPHIRSITMEREQDSIIMDAATRRNLEITQNLAGGAENTLASVLDCTVTPMGSRMLKRWLHMPVRDTRVLLERQQTIGALQDFTAGLQPVLRQVGDLERILARLALRTARPRDLARMRHAFQQLPELRAQLETVDSAPVQALREKMGEFAELRDLLERAIIDTPPVLVRDGGVIASGYNEELDEWRALADGATDYLERLEVRERERTGLDTLKVGFNAVHGYYIQISRGQSHLAPINYMRRQTLKNAERYIIPELKEYEDKVLTSKGKALALEKQLYEELFDLLLPHLEALQQSASALAELDVLVNLAERAYTLNYTCPTFIDKPGIRITEGRHPVvEQVlNepfiAnPLNLSPQRRMLIITGpnmGgkstYmrqTALIALMAYIGSYVPAQKVEIGPIDRIFTRVGAADDLASGRSTFMVEMTETANILHNATEYSLVLMdeIGRGTSTYDGLSLAWACAENLANKIKALTLFaTHYFELTQLPEKMEGVANVHLDALEHGDTIAFMhSVQDGAASKSYGLAVAALAGVPKEVIKRARQKLRELESIS

22222222222222222222222222222222222222222222222222222222222222222222222222222222222222222222222222222222222222222222222222222222222222222222222222222222222222222222222222222222222222222222222222222222222222222222222222222222222222222222222222222222222222222222222222222222222222222222222222222222222222222222222222222222222222222222222222222222222222222222222222222222222222222222222222222222222222222222222222222222222222222222222222222222222222222222222222222222222222222222222222222222222222222222222222222222222222222222222222222222222222222222222222222222222222222222222222222222222122212111121222222222222222111211112111222222222222222222222222222222222222222222222222222222222222222222112222222222222222222222222222222122222222222222222222222222222222212222222222222222222222222222222222222222

## #ATP of NUC5

>1MJHA

MSVMYKKILYPTDFSETAEIALKHVKAFKTLKAEEVILLHVIDEREIKKRDIFSLLLGVAGLNKSVEEFENELKNKLTEEAKNKMENIKKELEDVGFKVKDIIVVGIPHEEIVKIAEDEGVDIIIMGSHGKTNLKEILLGSVTENVIKKSNKPVLVVKRKNS

222222222211122221222222222222222222221112222222222222222222222222222222222222222222222222222222222222222221222122222222222221121111122222211112222222222222222222

>1NSFA

GAHMGDFLASLEQDIKPAFGTNQEDYASYIMNGIIKWGDPVTRVLDDGELLVQQTKNSDRTPLVSVLLEGPPHSGKTALAAKIAEESNFPFIKICSPDKMIGFSETAKCQAMKKIFDDAYKSQLSCVVVDDIERLLDYVPIGPRFSNLVLQALLVLLKKAPPQGRKLLIIGTTSRKDVLQEMEMLNAFSTTIHVPNIATGEQLLEALELLGNFKDKERTTIAQQVKGKKVWIGIKKLLMLIEMSLQMDPEYRVRKFLALLREEGASPLDFDLE

222222222222222222222222222221111112122212222222222222222222222222222221111111122222222222222222222222222222222222222222222222222222222222222222222222222222222222222222222221222222222222222222222222222222222222222222222222222222222221122122222222222222222222222222222222222

>3BLQA

GPAKQYDSVECPFCDEVSKYEKLAKIGQGTFGEVFKARHRKTGQKVALKKVLMENEKEGFPITALREIKILQLLKHENVVNLIEICRTKASPYNRCKGSIYLVFDFCEHDLAGLLSNVLVKFTLSEIKRVMQMLLNGLYYIHRNKILHRDMKAANVLITRDGVLKLADFGLARAFSLAKNSQPNRYUNRVVTLWYRPPELLLGERDYGPPIDLWGAGCIMAEMWTRSPIMQGNTEQHQLALISQLCGSITPEVWPNVDNYELYEKLELVKGQKRKVKDRLKAYVRDPYALDLIDKLLVLDPAQRIDSDDALNHDFFWSDPMPSDLKGMLST

2222222222222222222222222121222222222222222222121222222222222222222222222222222222222222222222222222222111122222222222222222222222222222222222222222212122121222222222212222222222222222222222222222222222222222222222222222222222222222222222222222222222222222222222222222222222222222222222222222222222222222222222222222222222222222222

>1OJLE

GSHMIGSSPAMQHLLNEIAMVAPSDATVLIHGDSGTGKELVARALHACSARSDRPLVTLNCAALNESLLESELFGHEKGAFTGADKRREGRFVEADGGTLFLDEIGDISPLMQVRLLRAIQEREVQRVGSNQTISVDVRLIAATHRDLAEEVSAGRFRQDLYYRLNVVAIEMPSLRQRREDIPLLADHFLRRFAERNRKVVKGFTPQAMDLLIHYDWPGNIRELENAIERAVVLLTGEYISERELPLAIAATPIKTEYSGEIQPLVDVEKEVILAALEKTGGNKTEAARQLGITRKTLLAKLSR

2222122222222222222222222222222221111112222222222222222222222222222222222222222222222222222222222222221222222222222222222222222222222222222222222222222222222222222222222222222221222222122212212222222222222222222222222222112212222222222222222222222222222222222222222222222222222222222222222222222222222222

>1PK8A

GSNYLRRRLSDSNFMANLPNGYMTDLQRPQPPPPPPSAASPGATPGSAAASAERASTAAPVASPAAPSPGSSGGGGFFSSLSNAVKQTTAAAAATFSEQVGGGSGGAGRGGAAARVLLVIDEPHTDWAKYFKGKKIHGEIDIKVEQAEFSDLNLVAHANGGFSVDMEVLRNGVKVVRSLKPDFVLIRQHAFSMARNGDYRSLVIGLQYAGIPSVNSLHSVYNFCDKPWVFAQMVRLHKKLGTEEFPLIDQTFYPNHKEMLSSTTYPVVVKMGHAHSGMGKVKVDNQHDFQDIASVVALTKTYATAEPFIDAKYDVRVQKIGQNYKAYMRTSVSGNWKTNTGSAMLEQIAMSDRYKLWVDTCSEIFGGLDICAVEALHGKDGRDHIIEVVGSSMPLIGDHQDEDKQLIVELVVNKMTQALPRQ

22222222222222222222222222222222222222222222222222222222222222222222222222222222222222222222222222222222222222222222222222222222222222222222222222222222222222222222222222222222222222222222222222222222222222222222222222222222212222222222222222222222222222222222222222212122211112212222222222222222222222222111122221222222222222221222222111122222222222222222222222222222222221212222222222122222222222222222222222222222222222

>3H1QA

SNAUELEQKLNLLNDLIVREIVNPLPPPYKVGVDLGTADIVLVVTDQEGIPVAGALKWASVVKDGLVVDYIGAIQIVRELKAKVERLLGSELFQAATAIPPGTVGRNAEACGHVVAGAGLELVTLVDEPVAAARALGINDGIVVDIGGGTTGIAVIEKGKITATFDEPTGGTHLSLVLAGSYKIPFEEAETIKKDFSRHREIURVVRPVIEKUALIVKEVIKNYDQTLPVYVVGGTAYLTGFSEEFSRFLGKEVQVPIHPLLVTPLGIALFG

22222222222222222222222222222222212111122222222222222222212222222222222222222222222222222222222222222222222222222222222222222221222222222222222212112222222222222222222222122222222222222222212211212212222222222222222222222222222222222111212222222222222222222222122222222222

>1Q12A

MASVQLQNVTKAWGEVVVSKDINLDIHEGEFVVFVGPSGCGKSTLLRMIAGLETITSGDLFIGEKRMNDTPPAERGVGMVFQSYALYPHLSVAENMSFGLKLAGAKKEVINQRVNQVAEVLQLAHLLDRKPKALSGGQRQRVAIGRTLVAEPSVFLLDEPLSNLDAALRVQMRIEISRLHKRLGRTMIYVTHDQVEAMTLADKIVVLDAGRVAQVGKPLELYHYPADRFVAGFIGSPKMNFLPVKVTATAIDQVQVELPMPNRQQVWLPVESRDVQVGANMSLGIRPEHLLPSDIADVILEGEVQVVEQLGNETQIHIQIPSIRQNLVYRQNDVVLVEEGATFAIGLPPERCHLFREDGTACRRLHKEPGVASASHHHHHH

222222222222122221222222222222222222211111112222222222222222222222222222222222222222222222222222222222222222222222222222222222221221111111222222222222222222222222122222222222222222222222222221222222222222222222222222222222222222222222222222222222222222222222222222222222222222222222222222222222222222222222222222222222222222222222222222222222222222222222222222222222222222222222222

>1PJ4A

IKEKGKPLULNPRTNKGUAFTLQERQULGLQGLLPPKIETQDIQALRFHRNLKKUTSPLEKYIYIUGIQERNEKLFYRILQDDIESLUPIVYTPTVGLACSQYGHIFRRPKGLFISISDRGHVRSIVDNWPENHVKAVVVTDGERILGLGDLGVYGUGIPVGKLCLYTACAGIRPDRCLPVCIDVGTDNIALLKDPFYUGLYQKRDRTQQYDDLIDEFUKAITDRYGRNTLIQFEDFGNHNAFRFLRKYREKYCTFNDDIQGTAAVALAGLLAAQKVISKPISEHKILFLGAGEAALGIANLIVUSUVENGLSEQEAQKKIWUFDKYGLLVKGRKAKIDSYQEPFTHSAPESIPDTFEDAVNILKPSTIIGVAGAGRLFTPDVIRAUASINERPVIFALSNPTAQAECTAEEAYTLTEGRCLFASGSPFGPVKLTDGRVFTPGQGNNVYIFPGVALAVILCNTRHISDSVFLEAAKALTSQLTDEELAQGRLYPPLANIQEVSINIAIKVTEYLYANKUAFRYPEPEDKAKYVKERTWRSEYDSLLPDVYEWPESASSPPVITE

222222222222222222222222222222222222222222222222222222222222222222222222222222222222222222222222222222222222222222222222222222222222212122222222122222222222222222222222222111221222222222222222222222222222222222222222222222212222222222222212222222222222222222222222222222222222222222222222211111122222222222222222222222222222112222222222222222222222222222222222222222222221111222222222222222222222222122222222222222222222222222222222222222222222222222222222221121222222222222222222222222222222222222222222222222222222222221222222222122212222222222222222222222222222

>1M83A

MKTIFSGIQPSGVITIGNYIGALRQFVELQHEYNCYFCIVDQHAITVWQDPHELRQNIRRLAALYLAVGIDPTQATLFIQSEVPAHAQAAWMLQCIVYIGELERMTQFKEKSAGKEAVSAGLLTYPPLMAADILLYNTDIVPVGEDQKQHIELTRDLAERFNKRYGELFTIPEARIPKVGARIMSLVDPTKKMSKSDPNPKAYITLLDDAKTIEKKIKSAVTDSEGTIRYDKEAKPGISNLLNIYSTLSGQSIEELERQYEGKGYGVFKADLAQVVIETLRPIQERYHHWMESEELDRVLDEGAEKANRVASEMVRKMEQAMGLGRRR

2222221112122222112211222222222222222222222222222222222222222222222222222222222222222222222222222222222222222212222222222222222222222222222222112112222222222222222222222222222222211112222222211111222222222222222222222222222222222222222222222222222222222222222222222222222222222222222222222222222222222222222222222222222222222222

>2HMUA

MGRIKNKQFAVIGLGRFGGSIVKELHRMGHEVLAVDINEEKVNAYASYATHAVIANATEENELLSLGIRNFEYVIVAIGANIQASTLTTLLLKELDIPNIWVKAQNYYHHKVLEKIGADRIIHPEKDMGVKIAQSLSDENVLNY

222222222222111112222222222222222221112212222222222222111122222222222222222221112221222222222222222222122222222222222222222212222222222222222222

>3BU5A

VFPSSVYVPDEWEVSREKITLLRELGQGSFGMVYEGNARDIIKGEAETRVAVKTVNESASLRERIEFLNEASVMKGFTCHHVVRLLGVVSKGQPTLVVMELMAHGDLKSYLRSLRPEAENNPGRPPPTLQEMIQMAAEIADGMAYLNAKKFVHRDLAARNCMVAHDFTVKIGDFGMTRDIUETDUURKGGKGLLPVRWMAPESLKDGVFTTSSDMWSFGVVLWEITSLAEQPYQGLSNEQVLKFVMDGGYLDQPDNCPERVTDLMRMCWQFNPNMRPTFLEIVNLLKDDLHPSFPEVSFFHSEENK

222222222222222222222222222222222222222222222222221212222222222222222122222222222222222222222222221111222122222222222222222222222222222222222222222222222222221121222222222212122222222222222222222222222222222222222222222222222222222222222222222222222222222222222222222222222222222222222222222222222222222222

>2B6FA

GAPEGPGPSGGAQGGSIHSGRIAAVHNVPLSVLIRPLPSVLDPAKVQSLVDTIREDPDSVPPIDVLWIKGAQGGDYFYSFGGCHRYAAYQQLQRETIPAKLVQSTLSDLRVYLGASTPDLQ

2222222222222222222222222222222222222222222212212221222222222222222222222222222211111222222222222222222222222222222222222

>1KMNA

MAKNIQAIRGMNDYLPGETAIWQRIEGTLKNVLGSYGYSEIRLPIVEQTPLFKRAIGEVTDVVEKEMYTFEDRNGDSLTLRPEGTAGCVRAGIEHGLLYNQEQRLWYIGPMFRHERPQKGRYRQFHQLGCEVFGLQGPDIDAELIMLTARWWRALGISEHVTLELNSIGSLEARANYRDALVAFLEQHKEKLDEDCKRRMYTNPLRVLDSKNPEVQALLNDAPALGDYLDEESREHFAGLCKLLESAGIAYTVNQRLVRGLDYYNRTVFEWVTNSLGSQGTVCAGGRYDGLVEQLGGRATPAVGFAMGLERLVLLVQAVNPEFKADPVVDIYLVASGADTQSAAMALAERLRDELPGVKLMTNHGGGNFKKQFARADKWGARVAVVLGESEVANGTAVVKDLRSGEQTAVAQDSVAAHLRTLLG

2222222222222222222222222222222222222222222222222222222222222222222222222222222222222222222222222222222222222222121222211122121222222222222222222222222222222222222222222222222222222222222222222222222222222222222222222222222222222222222222222222222222222222221222222222222222222222112122222222222222222222222122122222222222222222222222222222222222222222222222222222222222222222222222222222222222222222222222222222222222222222

>3CISA

MRGSHHHHHHGSMSSGNSSLGIIVGIDDSPAAQVAVRWAARDAELRKIPLTLVHAVSPEVATWLEVPLPPGVLRWQQDHGRHLIDDALKVVEQASLRAGPPTVHSEIVPAAAVPTLVDMSKDAVLMVVGCLGSGRWPGRLLGSVSSGLLRHAHCPVVIIHDEDSVMPHPQQAPVLVGVDGSSASELATAIAFDEASRRNVDLVALHAWSDVDVSEWPGIDWPATQSMAEQVLAERLAGWQERYPNVAITRVVVRDQPARQLVQRSEEAQLVVVGSRGRGGYAGMLVGSVGETVAQLARTPVIVARESLT

222222222222222222222222111212212222222222222222222211122222222222222222222222222222222222222222222222222222222122222222222222211211111222122111122222222222222222222222222222221112222122222222222222222222111221222222222222222222222222222222222222222222222212222222222222221121111122212211112222222222222222222

>1KP8A

AAKDVKFGNDAGVKMLRGVNVLADAVKVTLGPKGRNVVLDKSFGAPTITKDGVSVAREIELEDKFENMGAQMVKEVASKANDAAGDGTTTATVLAQAIITEGLKAVAAGMNPMDLKRGIDKAVTVAVEELKALSVPCSDSKAIAQVGTISANSDETVGKLIAEAMDKVGKEGVITVEDGTGLQDELDVVEGMQFDRGYLSPYFINKPETGAVELESPFILLADKKISNIREMLPVLEAVAKAGKPLLIIAEDVEGEALATLVVNTMRGIVKVAAVKAPGFGDRRKAMLQDIATLTGGTVISEEIGMELEKATLEDLGQAKRVVINKDTTTIIDGVGEEAAIQGRVAQIRQQIEEATSDYDREKLQERVAKLAGGVAVIKVGAATEVEMKEKKARVEDALHATRAAVEEGVVAGGGVALIRVASKLADLRGQNADQNVGIKVALRAMEAPLRQIVLNCGEEPSVVANTVKGGDGNYGYNAATEEYGNMIDMGILDPTKVTRSALQYAASVAGLMITTECMVTDLPKNDAADLGAAGGMGGMGGMGGMM

2222222222222222222222222222111122222222222222222221222222222222222222222222222222222111112222222222222222222222222222222222222222222222222222222222222222222222222222222222222222222222222222222222222222222222222222222222222222222222222222222222222222222222222222222222222222222222222222222222222222222222222222222222222222222222222222222222222222222222222222222222222222222222222222222222222222222222222222222222112222222222222222222222222222222222222222222222222222222222222211112222222222212122222222222222222222222222222222222222222222222222222

>1AYLA

MRVNNGLTPQELEAYGISDVHDIVYNPSYDLLYQEELDPSLTGYERGVLTNLGAVAVDTGIFTGRSPKDKYIVRDDTTRDTFWWADKGKGKNDNKPLSPETWQHLKGLVTRQLSGKRLFVVDAFCGANPDTRLSVRFITEVAWQAHFVKNMFIRPSDEELAGFKPDFIVMNGAKCTNPQWKEQGLNSENFVAFNLTERMQLIGGTWYGGEMKKGMFSMMNYLLPLKGIASMHCSANVGEKGDVAVFFGLSGTGKTTLSTDPKRRLIGDDEHGWDDDGVFNFEGGCYAKTIKLSKEAEPEIYNAIRRDALLENVTVREDGTIDFDDGSKTENTRVSYPIYHIDNIVKPVSKAGHATKVIFLTADAFGVLPPVSRLTADQTQYHFLSGFTAKLAGTERGITEPTPTFSACFGAAFLSLHPTQYAEVLVKRMQAAGAQAYLVNTGWNGTGKRISIKDTRAIIDAILNGSLDNAETFTLPMFNLAIPTELPGVDTKILDPRNTYASPEQWQEKAETLAKLFIDNFDKYTDTPAGAALVAAGPKLS

2222222222222222222222222222222222222222222222222222222222222222222222222222222222222222222222222222222222222222222222222222222222222222222222222222222222222222222222222222222222222222222222222222222222222222222222222222222222222221222222222222222211111111222222222222222222222222222222212122222212222222222222222222222222222222222212222222222222222222222222222222222222222222222222222222222222222222222222222222222222222222222222222222222212222222111122122222222222222222222222222222222222222222222222222222222222222222222222222222222222222

>2QK4A

MHHHHHHSSGVDLGTENLYFQSMAARVLIIGSGGREHTLAWKLAQSHHVKQVLVAPGNAGTACSEKISNTAISISDHTALAQFCKEKKIEFVVVGPEAPLAAGIVGNLRSAGVQCFGPTAEAAQLESSKRFAKEFMDRHGIPTAQWKAFTKPEEACSFILSADFPALVVKASGLAAGKGVIVAKSKEEACKAVQEIMQEKAFGAAGETIVIEELLDGEEVSCLCFTDGKTVAPMPPAQDHKRLLEGDGGPNTGGMGAYCPAPQVSNDLLLKIKDTVLQRTVDGMQQEGTPYTGILYAGIMLTKNGPKVLEFNCRFGDPECQVILPLLKSDLYEVIQSTLDGLLCTSLPVWLENHTALTVVMASKGYPGDYTKGVEITGFPEAQALGLEVFHAGTALKNGKVVTHGGRVLAVTAIRENLISALEEAKKGLAAIKFEGAIYRKDIGFRAIAFLQ

22222222222222222222222222222222222222222222222222222222222222222222222222222222222222222222222222222222222222222222222222222222222222222222222222222222222222222222222221222222222221222222222222222222222222222221111222122222222222222222222221222222221222222222222222222222222222222222222222222222222122222222122222222222222222222222222222222222222222222222222222222222222222222222222222222222222222222222222222222222222222222222222222222222222222222222

>2R9VA

UGSDKIHHHHHHLRINPGEITKVLEEKIKSFEEKIDLEDTGKVIQVGDGIARAYGLNKVUVSELVEFVETGVKGVAFNLEEDNVGIIILGEYKDIKEGHTVRRLKRIIEVPVGEELLGRVVNPLGEPLDGKGPINAKNFRPIEIKAPGVIYRKPVDTPLQTGIKAIDSUIPIGRGQRELIIGDRQTGKTAIAIDTIINQKGQGVYCIYVAIGQKKSAIARIIDKLRQYGAUEYTTVVVASASDPASLQYIAPYAGCAUGEYFAYSGRDALVVYDDLSKHAVAYRQLSLLURRPPGREAYPGDIFYLHSRLLERAVRLNDKLGGGSLTALPIVETQANDISAYIPTNVISITDGQIYLEPGLFYAGQRPAINVGLSVSRVGGSAQIKAUKQVAGULRIDLAQYRELETFAQFATELDPATRAQIIRGQRLUELLKQEQYSPUPVEEQVVVLFAGVRGYLDDLPVEEVRRFEKEFLRFUHEKHQDILDDIKTKKELTSETEEKLKKAIEEFKTTFRV

22222222222222222222222222222222222222222222222222222222222222222222222222222222222222222222222222222222222222222222222222222222222222222222222222222222222222222222222222222222222222111111112222222222222222222222222222222222222222222222222222222222222222222222222222222222222222222222222222222222222222222222222222222222222222222222222222222222222222222222222221222211222222222222222222222222222222222222222222222222222222222222222222121222222222222222222222222222222222222222222222222222222222222222222222222222222

>2Z08A

MFKTILLAYDGSEHARRAAEVAKAEAEAHGARLIVVHAYEPVPDYLGEPFFEEALRRRLERAEGVLEEARALTGVPKEDALLLEGVPAEAILQAARAEKADLIVMGTRGLGALGSLFLGSQSQRVVAEAPCPVLLVR

22222221112122222222222222222222222111222222222222222222222222222222222222222222222222122222222222222222112111112222221111222222222222222

>2VT3A

MNKDQSKIPQATAKRLPLYYRFLKNLHASGKQRVSSAELSDAVKVDSATIRRDFSYFGALGKKGYGYNVDYLLSFFRKTLDQDEMTDVILIGVGNLGTAFLHYNFTKNNNTKISMAFDINESKIGTEVGGVPVYNLDDLEQHVKDESVAILTVPAVAAQSITDRLVALGIKGILNFTPARLNVPEHIRIHHIDLAVELQSLVYFLKHYSVLEEIE

22222222222222222222222222222222222222222222222222222222222222222222222222222222222222222211111222222222222222222222211222122222222222212222222222222221112212221222222222222222222222222222222222222222222222222222222

>1YP3A

MAVSDSQNSQTCLDPDASRSVLGIILGGGAGTRLYPLTKKRAKPAVPLGANYRLIDIPVSNCLNSNISKIYVLTQFNSASLNRHLSRAYASNMGGYKNEGFVEVLAAQQSPENPDWFQGTADAVRQYLWLFEEHTVLEYLILAGDHLYRMDYEKFIQAHRETDADITVAALPMDEKRATAFGLMKIDEEGRIIEFAEKPQGEQLQAMKVDTTILGLDDKRAKEMPFIASMGIYVISKDVMLNLLRDKFPGANDFGSEVIPGATSLGMRVQAYLYDGYWEDIGTIEAFYNANLGITKKPVPDFSFYDRSAPIYTQPRYLPPSKMLDADVTDSVIGEGCVIKNCKIHHSVVGLRSCISEGAIIEDSLLMGADYYETDADRKLLAAKGSVPIGIGKNCHIKRAIIDKNARIGDNVKIINKDNVQEAARETDGYFIKSGIVTVIKDALIPSGIII

2222222222222222222222222121122222222222221222222222222222222222222222221212222222222222222222222222222222222222222221112212222222222222222222111222222222222222222222222222222222222222222222222222222222222222222222222222222222222222122222222222222222221111222222222222222222222222222222222222222222222222222222222222222222222222222222222222222222222222222222222222222222222222222222222222222222222222222222222222222222222222222222222222222222222222222

>1B8AA

MYRTHYSSEITEELNGQKVKVAGWVWEVKDLGGIKFLWIRDRDGIVQITAPKKKVDPELFKLIPKLRSEDVVAVEGVVNFTPKAKLGFEILPEKIVVLNRAETPLPLDPTGKVKAELDTRLNNRFMDLRRPEVMAIFKIRSSVFKAVRDFFHENGFIEIHTPKIIATATEGGTELFPMKYFEEDAFLAESPQLYKEIMMASGLDRVYEIAPIFRAEEHNTTRHLNEAWSIDSEMAFIEDEEEVMSFLERLVAHAINYVREHNAKELDILNFELEEPKLPFPRVSYDKALEILGDLGKEIPWGEDIDTEGERLLGKYMMENENAPLYFLYQYPSEAKPFYIMKYDNKPEICRAFDLEYRGVEISSGGQREHRHDILVEQIKEKGLNPESFEFYLKAFRYGMPPHGGFGLGAERLIKQMLDLPNIREVILFPRDRRRLTP

222222222222222222222222222222222222222222222222222222222222222222222222222222222222222222222222222222222222222222222222222222222222222222222222222222222222222222222222222222222222222222222222222222222222222222222121222221112212222222222222222222222222222222222222222222222222222222222222222222222222222222222222222222222222222222222222222222222222222222222222111122222222222222222222222222222222222222222212122122222222222222222222222222

>2ARUA

MEGRLLLLETPGNTRMSLAYDEAIYRSFQYGDKPILRFYRHDRSVIIGYFQVAEEEVDLDYMKKNGIMLARRYTGGGAVYHDLGDLNFSVVRSSDDMDITSMFRTMNEAVVNSLRILGLDARPGELNDVSIPVNKKTDIMAGEKKIMGAAGAMRKGAKLWHAAMLVHTDLDMLSAVLKVPDEKFRDKIAKSTRERVANVTDFVDVSIDEVRNALIRGFSETLHIDFREDTITEKEESLARELFDKKYSTEEWNMGLLRKEVV

2222222222222222222222222222222222222222222222222222222222222222222222212222111112221222222222222222222222222222222222222222222212112212212222221221112222222222221212222222122212222222222222222222222222222222222222222222222222222222222222222222222222222222222222

>2C01X

MKPPQFTWAQWFETQHINMTSQQCTNAMQVINNYQRRCKNQNTFLLTTFANVVNVCGNPNMTCPSNKTRKNCHHSGSQVPLIHCNLTTPSPQNISNCRYAQTPANMFYIVACDNRDQRRDPPQYPVVPVHLDRII

222222212222221122222222222222222222221222222222222222222222221222221212222222222222222222222222222222222222221212222222222222221112222

>2NT8A

MGSSHHHHHHSSGLVPRGSHMASMTGGQQMGRDRSVKIYTKNGDKGQTRIIGKQILYKNDPRVAAYGEVDELNSWVGYTKSLINSHTQVLSNELEEIQQLLFDCGHDLATPADDERHSFKFKQEQPTVWLEEKIDNYTQVVPAVKKFILPGGTQLASALHVARTITRRAERQIVQLMREEQINQDVLIFINRLSDYFFAAARYANYLEQQPDMLYRNSKDVFR

2222222222222222222222222222222222222211111222111222222221222212211222222222222222222222222222222222222222222222222222222222222222222222222222222222222222222222222222222222222222222222222222222222222222222222222222222222222

>1MIWA

UKPPFQEALGIIQQLKQHGYDAYFVGGAVRDLLLGRPIGDVDIATSALPEDVUAIFPKTIDVGSKHGTVVVVHKGKAYEVTTFKTDGDYEDYRRPESVTFVRSLEEDLKRRDFTUNAIAUDEYGTIIDPFGGREAIRRRIIRTVGEAEKRFREDALRUURAVRFVSELGFALAPDTEQAIVQNAPLLAHISVERUTUEUEKLLGGPFAARALPLLAETGLNAYLPGLAGKEKQLRLAAAYRWPWLAAREERWALLCHALGVQESRPFLRAWKLPNKVVDEAGAILTALADIPRPEAWTNEQLFSAGLERALSVETVRAAFTGAPPGPWHEKLRRRFASLPIKTKGELAVNGKDVIEWVGKPAGPWVKEALDAIWRAVVNGEVENEKERIYAWLUERNRTREKNC

22222222222222222222222221122122222222222222222222222222222222222222222222222222222222222222222222222222222221112222222222222222222222222222222222222222212212212211221222222222222222222222222222222222222222222222222222222222222222222222222222222222222222222222222222222222222222222222222222222222222222222222222222222222222222222222222222222222222222222222222222222222222222222222222222222222222222222222

>2IA6A

MIVLFVDFDYFYAQVEEVLNPSLKGKPVVVCVFSGRFEDSGAVATANYEARKFGVKAGIPIVEAKKILPNAVYLPMRKEVYQQVSSRIMNLLREYSEKIEIASIDEAYLDISDKVRDYREAYNLGLEIKNKILEKEKITVTVGISKNKVFAKIAADMAKPNGIKVIDDEEVKRLIRELDIADVPGIGNITAEKLKKLGINKLVDTLSIEFDKLKGMIGEAKAKYLISLARDEYNEPIRTRVRKSIGRIVTMKRNSRNLEEIKPYLFRAIEESYYKLDKRIPKAIHVVAVTEDLDIVSRGRTFPHGISKETAYSESVKLLQKILEEDERKIRRIGVRFSKFIEAIGLDKFFDT

2222221121122222222222222222222222222222222212212212222211222222222222222222222222222222222222222222222211222222222222222222222222222222222222222222222222222212222222222222222222222222222222222222222222222222222222222222222222222222222222222222222222222222222222222222222222222222222222222222222222222222222222222222222222222222222222222222222222222222

>1H4QB

MAKEKGLTPQSQDFSEWYLEVIQKAELADYGPVRGTIVVRPYGYAIWENIQQVLDRMFKETGHQNAYFPLFIPMSFLRKEAEHVEGFSPELAVVTHAGGEELEEPLAVRPTSETVIGYMWSKWIRSWRDLPQLLNQWGNVVRWEMRTRPFLRTSEFLWQEGHTAHATREEAEEEVRRMLSIYARLAREYAAIPVIEGLKTEKEKFAGAVYTTTIEALMKDGKALQAGTSHYLGENFARAFDIKFQDRDLQVKYVHTTSWGLSWRFIGAIIMTHGDDRGLVLPPRLAPIQVVIVPIYKDESRERVLEAAQGLRQALLAQGLRVHLDDRDQHTPGYKFHEWELKGVPFRVELGPKDLEGGQAVLASRLGGKETLPLAALPEALPGKLDAFHEELYRRALAFREDHTRKVDTYEAFKEAVQEGFALAFHCGDKACERLIQEETTATTRCVPFEAEPEEGFCVRCGRPSAYGKRVVFAKAY

222222222222222222222222222222222222222222222222222222222222222222222222222222222222222222222222222222222222222222222222222222222222222222222121222222111221212222222222222222222222222222222222222222222222222222222222222222221111212222222222222222222222222222212121222222222222222222222222222222222222222222222222222222222222222222222222222222222222222222222222222222222222222222222222222222222222222222222222222222222222222222222222222222222222222222222222222222222222222222222

>3DWLA

MASFNVPIIMDNGTGYSKLGYAGNDAPSYVFPTVIATRSAGASSGPAVSSKPSYMASKGSGHLSSKRATEDLDFFIGNDALKKASAGYSLDYPIRHGQIENWDHMERFWQQSLFKYLRCEPEDHYFLLTEPPLNPPENRENTAEIMFESFNCAGLYIAVQAVLALAASWTSSKVTDRSLTGTVVDSGDGVTHIIPVAEGYVIGSSIKTMPLAGRDVTYFVQSLLRDRNEPDSSLKTAERIKEECCYVCPDIVKEFSRFDREPDRYLKYASESITGHSTTIDVGFERFLAPEIFFNPEIASSDFLTPLPELVDNVVQSSPIDVRKGLYKNIVLSGGSTLFKNFGNRLQRDLKRIVDERIHRSEMLSGAKSGGVDVNVISHKRQRNAVWFGGSLLAQTPEFGSYCHTKADYEEYGASIARRYQIFGNSL

2222222222222221222222222222222222222222222222222222222222222222222222222222222222222222222222222222222222222222222222222222222222222222222222222222222222222222222222222222222222222222211122222222222222222222222212222222222222222222222221221122222222222222222222222222222222222222222222222222222222222222222222222222222222222222222221112112222222222222222222222222222222222222222222222222222222222222222222222222222222222222222

>2IXEA

MSPLSGSLAPLNMKGLVKFQDVSFAYPNHPNVQVLQGLTFTLYPGKVTALVGPNGSGKSTVAALLQNLYQPTGGKVLLDGEPLVQYDHHYLHTQVAAVGQEPLLFGRSFRENIAYGLTRTPTMEEITAVAMESGAHDFISGFPQGYDTEVGETGNQLSGGQRQAVALARALIRKPRLLILDNATSALDAGNQLRVQRLLYESPEWASRTVLLITQQLSLAERAHHILFLKEGSVCEQGTHLQLMERGGCYRSMVEALAAPSDAAAHHHHHH

2222222222222222222222222122122221222222222222222222111111112222222222222222222222222222222222222222222222222222222222222222222222222222222222222222222222211111122222222222222222222222222222222222222222222222222222122222222222222222222222222222222222222222222222222222222

>2IYWA

MAPKAVLVGLPGSGKSTIGRRLAKALGVGLLDTDVAIEQRTGRSIADIFATDGEQEFRRIEEDVVRAALADHDGVLSLGGGAVTSPGVRAALAGHTVVYLEISAAEGVRRTGGNTVRPLLAGPDRAEKYRALMAKRAPLYRRVATMRVDTNRRNPGAVVRHILSRLQVPSPSEAATLEHHHHHH

2222222221111111122222222222222222222222222222222222222222222222222222222222222222222222222222222222222222222122222222222222222222222222222222222222212211122122222222222222222222222222

>2EWWA

UFEKQEVEQKKELKILEIIKEAIELGASDIHLTAGAPPAVRIDGYIKFLKDFPRLTPEDTQKLAYSVUSEKHRQKLEENGQVDFSFGVRGVGRFRANVFYQRGSVAAALRSLPAEIPEFKKLGLPDKVLELCHRKUGLILVTGPTGSGKSTTIASUIDYINQTKSYHIITIEDPIEYVFKHKKSIVNQREVGEDTKSFADALRAALREDPDVIFVGEURDLETVETALRAAETGHLVFGTLHTNTAIDTIHRIVDIFPLNQQEQVRIVLSFILQGIISQRLLPKIGGGRVLAYELLIPNTAIRNLIRENKLQQVYSLUQSGQAETGUQTUNQTLYKLYKQGLITLEDAUEASPDPKELERUIRGGRHHHHHH

222222222222222222222222222222222222222222222222222222222222222222222222222222222222222222222222222222222222222222222222212222222222222222222221111111122222222222222222222222222222222222222222222222222222222222222222222222222222222222222222222222222222222222222222222222222222222212222222121222222222222222222222222222222222222222222222222222222222222222222222222222222222

>3H5NA

GSHMDYILGRYVKIARYGSGGLVGGGGKEQYVENLVLWENIIKTAYCFITPSSYTAALETANIPEKDFSNCFRFLKENFFIIPGEYNNSTENNRYSRNFLHYQSYGANPVLVQDKLKNAKVVILGCGGIGNHVSVILATSGIGEIILIDNDQIENTNLTRQVLFSEDDVGKNKTEVIKRELLKRNSEISVSEIALNINDYTDLHKVPEADIWVVSADHPFNLINWVNKYCVRANQPYINAGYVNDIAVFGPLYVPGKTGCYECQKVVADLYGSEKENIDHKIKLINSRFKPATFAPVNNVAAALCAADVIKFIGKYSEPLSLNKRIGIWSDEIKIHSQNMGRSPVCSVCGNRM

22222222222222222222222222222222222222222222222222222222222222222222222222222222222222222222222212222222222222222222222222221111222222222222222222221212222212211222222222221222222222222222222222111222222222222222222111222122222222222222222221222222222222222222222222222222222222222222222222222222222222222222222222222222222222222222222222222222222222222

>2KMXA

SFTMHGTPVVNQVKVLTESNRISHHKILAIVGTAESNSEHPLGTAITKYCKQELDTETLGTCIDFQVVPGCGISCKVTNIEGLLHKNNWNIEDNNIKNASLVQIDASNEQSSTSSSMIIDAQISNALNAQQYKVLIGNREWMIRNGLVINNDVNDFMTEHERKGRTAVLVAVDDELCGLIAIADT

22222222222222222222222222222222221222211112222222222222222222222222212122222222222222222222222222222222222222222222222222222222222222211122222222222222222222222222222122222222222222222

>1ZYDA

SLRYASDFEEIAVLGQGAFGQVVKARNALDSRYYAIKKIRHTEEKLSTILSEVMLLASLNHQYVVRYYAAWLERRNFVKPMTAVKKKSTLFIQMEYCENRTLYDLIHSENLNQQRDEYWRLFRQILEALSYIHSQGIIHRDLKPMNIFIDESRNVKIGDFGLAKNVHRSLDILKLDSQNLPGSSDNLTSAIGTAMYVATEVLDGTGHYNEKIDMYSLGIIFFEMIYPFSTGMERVNILKKLRSVSIEFPPDFDDNKMKVEKKIIRLLIDHDPNKRPGARTLLNSGWLPVKHQDEVIKEALKSL

222222222222212222222122222222222212122222222222222222222222222222222222222222222222222222222211122222222222222222222222222222222222222222221212212122222222221222222222222222222222222222222222222222222222222222222222222222222222222222222222222222222222222222222222222222222222222222222222222222222222222

>2AQXA

MVQWSPFVMSFKKKYPWIQLAGHAGSFKAAANGRILKKHCESEQRCLDRLMADVLRPFVPAYHGDVVKDGERYNQMDDLLADFDSPCVMDCKMGVRTYLEEELTKARKKPSLRKDMYQKMVEVDPEAPTEEEKAQRAVTKPRYMQWRETISSTATLGFRIEGIKKEDGSVNRDFKKTKTREQVTEAFREFTKGNQNILIAYRDRLKAIRATLEISPFFKCHEVIGSSLLFIHDKKEQAKVWMIDFGKTTPLPEGQTLQHDVPWQEGNREDGYLSGLDNLIDILTEMSQG

2222222222222222111111222212222222221222222222222222222222212222222222222221111222222222212122222222222222222222222222222222222222222222222222222222222222222222222222222222222222222222222222222222222222222222222222222222222222221222222222222211221222222222222222222222222222222222222222222

>3IN5A

MGLNDNKAGMEGLDKEKINKIIMEATKGSRFYGNELKKEKQVNQRIENMMQQKAQITSQQLRKAQLQVDRFAMELEQSRNLSNTIVHIDMDAFYAAVEMRDNPELKDKPIAVGSMSMLSTSNYHARRFGVRAAMPGFIAKRLCPQLIIVPPNFDKYRAVSKEVKEILADYDPNFMAMSLDEAYLNITKHLEERQNWPEDKRRYFIKMGSSVENDNPGKEVNKLSEHERSISPLLFEESPSDVQPPGDPFQVNFEEQNNPQILQNSVVFGTSAQEVVKEIRFRIEQKTTLTASAGIAPNTMLAKVCSDKNKPNGQYQILPNRQAVMDFIKDLPIRKVSGIGKVTEKMLKALGIITCTELYQQRALLSLLFSETSWHYFLHISLGLGSTHLTRDGERKSMSVERTFSEINKAEEQYSLCQELCSELAQDLQKERLKGRTVTIKLKNVNFEVKTRASTVSSVVSTAEEIFAIAKELLKTEIDADFPHPLRLRLMGVRISSFPNEEDRKHQQ

2222222122222222222222222222222222222222222222222222222222222222222222222222222222222222111111222222222222222222222222112212212222221222222222222222222222222222222222222222222222211222222222222222222222222222222222222222222222222222222222222222222222222222222222222222222222222222222222222222222222222222222221222222222222222222222222222222222222222222222222222222222222222222222222222222222222222222222222222222222222222222222222222222222222222222222222222222222222222222222222222222222222222222222222222222

>2BEKA

MLRAKVRRIALANQKGGVGKTTTAINLAAYLARLGKRVLLVDLAPQGNATSGLGVRAERGVYHLLQGEPLEGLVHPVDGFHLLPATPDLVGATVELAGAPTALREALRDEGYDLVLLDAPPSLSPLTLNALAAAEGVVVPVQAEYYALEGVAGLLATLEEVRAGLNPRLRLLGILVTMYDGRTLLAQQVEAQLRAHFGEKVFWTVIPRNVRLAEAPSFGKTIAQHAPTSPGAHAYRRLAEEVMARVQEAGSHHHHHH

22222222222222111111112222222222222222222222222222222222222222222222222222222222222222222222222222222222222222222222222212222222222222222222212121222222222222222222222222222222212222222222222222222222222221111221122222222222222222222222222222222222222222222

>2ZDQA

MRVLLIAGGVSPEHEVSLLSAEGVLRHIPFPTDLAVIAQDGRWLLGEKALTALEAKAAPEGEHPFPPPLSWERYDVVFPLLHGRFGEDGTVQGFLELLGKPYVGAGVAASALCMDKDLSKRVLAQAGVPVVPWVAVRKGEPPVVPFDPPFFVKPANTGSSVGISRVERFQDLEAALALAFRYDEKAVVEKALSPVRELEVGVLGNVFGEASPVGEVRYEAPFYDYETKYTPGRAELLIPAPLDPGTQETVQELALKAYKVLGVRGMARVDFFLAEGELYLNELNTIPGFTPTSMYPRLFEAGGVAYPELLRRLVELALT

2222222222222222222222222222222222222222222222222222222222222222222222222222222222222222222222222222222222222222222122222222222222122222222222222222221212221111221222222222222222222222222211112222122222222222222222222122211222212222222222222222222222222222222222222221212122222222112122222222222222222222222222222222222

>3IBQA

USLSTULVAEDLSAVGGISLSSALPVLTAUQYDVAALPTSLLSTHTSGYGTPAVVDLSTWLPQVFAHWTRAQLHFDQALIGYVGSVALCQQITTYLEQQTLSLLVVDPVLGDLGQLYQGFDQDYVAAURQLIQQADVILPNTTEAALLTGAPYQVTPDLEVILPALQAQLKTGAHAVITDVQRADQIGCAWLDEAGHVQYCGARRLPGHYNGTGDTLAAVIAGLLGRGYPLAPTLARANQWLNUAVAETIAQNRTDDRQGVALGDLLQAILALNEGHHHHHH

222222222222222222222222222222222222222222222222222222222222222222222222222222222222222222222222222222222222222222222222222222222222222222221222222222222222222222222222222222222211222222122222222222222222112121211122122222222222222222222212211222222222222222222222222222222222222222

>1DY3A

TVAYIAIGSNLASPLEQVNAALKALGDIPESHILTVSSFYRTPPLGPQDQPDYLNAAVALETSLAPEELLNHTQRIELQQGRVRKAERWGPRTLDLDIMLFGNEVINTERLTVPHYDMKNRGFMLWPLFEIAPELVFPDGEMLRQILHTRAFDKLNKW

22222222222222222222222222222222222222222222222222222222222222222222222221222222212122211221221211222222222221112211222212222222222222222222222222222222222222

>3LGXA

MSLKDMIDSIEQFAQTQADFPVYDCLGERRTYGQLKRDSDSIAAFIDSLALLAKSPVLVFGAQTYDMLATFVALTKSGHAYIPVDVHSAPERILAIIEIAKPSLIIAIEEFPLTIEGISLVSLSEIESAKLAEMPYERTHSVKGDDNYYIIFTSGTTGQPKGVQISHDNLLSFTNWMIEDAAFDVPKQPQMLAQPPYSFDLSVMYWAPTLALGGTLFALPKELVADFKQLFTTIAQLPVGIWTSTPSFADMAMLSDDFCQAKMPALTHFYFDGEELTVSTARKLFERFPSAKIINAYGPTEATVALSAIEITREMVDNYTRLPIGYPKPDSPTYIIDEDGKELSSGEQGEIIVTGPAVSKGYLNNPEKTAEAFFTFKGQPAYHTGDIGSLTEDNILLYGGRLDFQIKYAGYRIELEDVSQQLNQSPMVASAVAVPRYNKEHKVQNLLAYIVVKDGVKERFDRELELTKAIKASVKDHMMSYMMPSKFLYRDSLPLTPNGKIDIKTLINEVNNREGHHHHHH

22222222222222222222222222222222222222222222222222222222222222222222222222222222222222222222222222222222222222222222222222222222222222222222222222222222112222222222222222222222222222222222222222222222222222222222222222222222222222222222222222222222222222222222222222222221111222222222222222222211111122222222222222222222222122222222222222222222222222222222222222222222222222222222222121222222222221221222222222222222222222222222222222222222222222222222222222222222222222222222222222222222222222222121222222222222222222222

>2HIXA

MGSSHHHHHHSSGLVPRGSHMEFKVIAEYFDKLEKISSRLQLTALLADLLSKSDKTIIDKVVYIIQGKLWPDFLGYPELGIGEKFLIKAISIATNTDENSVENLYKTIGDLGEVARRLKSKQQSTGILGFLGTTSKESLTVDEVYSTLSKVALTTGEGSRDLKIRLLAGLLKKADPLEAKFLVRFVEGRLRVGIGDATVLDAMAIAFGGGQSASEIIERAYNLRADLGNIAKIIVEKGIEALKTLKPQVGIPIRPMLAERLSNPEEILKKMGGNAIVDYKYDGERAQIHKKEDKIFIFSRRLENITSQYPDVVDYVSKYIEGKEFIIEGEIVAIDPESGEMRPFQELMHRKRKSDIYEAIKEYPVNVFLFDLMYYEDVDYTTKPLEARRKLLESIVKPNDYVKIAHHIQANNVEDLKSFFYRAISEGGEGVMVKAIGKDAIYQAGARGWLWIKLKRDYQSEMADTVDLVVVGGFYGKGKRGGKISSLLMAAYNPKTDSFESVCKVASGFSDEQLDELQKKLMEIKRDVKHPRVNSKMEPDIWVEPVYVAEIIGSEITISPLHTCCQDVVEKDAGLSIRFPRFIRWRDDKSPEDATTTDEILEMYNKQPKKKIESPAVDESV

222222222222222222222222222222222222222222222222222222222222222222222222222222222222222222222222222222222222222222222222222222222222222222222222222222222222222222222222222222222222222222222222222222222222222222222222222222222222222222222222222222222222222222222222222222222222211112221222222222222221222222222222222222222222222221222222222222222222222222222222222222222122222222222222222222222222222222222222222222222222222222222221212222222222221222121212222222222222222222222222222222222222222222222222222222222222222222222222222222222222222222222222222222222222222222222222222222222222222222222222222222222222222222222

>3LSSA

GPGSMMVLDIQLFRDETGANIIRESQRRRFADPDIVDAIIEADKKWRRTQFLTEASKKLINICSKAVGAKKKAKEADGDTSEIPPQVKEAYENGTLKGEQVEQLCVLQLKQLSKDLSDQVAGLAKEAQQLEEERDKLMLNVGNILHESVPIAQDEETGNTVVRTFGNTTKRAKLNHVSIMERLGMMDTSKAVTSMAGGRSYVLKGGLVQLQVALVSYSLDFLVKRGYTPFYPPFFLNRDVMGEVAQLSQFDEELYQVSGDGDKKYLIATSEMPIAAYHRGRWFTELKEPLKYAGMSTCFRKEAGAHGRDTLGIFRVHQFDKIEQFVVCSPRQEESWRHLEDMITTSEEFNKSLGLPYRVVNICSGALNNAAAKKYDLEAWFPASGAFRELVSCSNCTDYQSQSVNCRYGPNLRGTAAQNVKEYCHMLNGTLCAITRTMCCICENYQTEEGVVIPDVLRPYMMGIEMIRFENNAQAEGTTPDKGE

2222222222222222222222222222222222222222222222222222222222222222222222222222222222222222222222222222222222222222222222222222222222222222222222222222222222222222222222222222222222222222222222222222222222222222222222222222222222222222222222222222222222222222222222222222222222222222222222222222222222212122222222222111221212222222222222222222222222222222222222222222222222222122222222222222111122222222222222222222222222222222222221221211222222222222222222222222222222222222222222222222

>1TILA

MRNEMHLQFSARSENESFARVTVAAFVAQLDPTMDELTEIKTVVSEAVTNAIIHGYNNDPNGIVSISVIIEDGVVHLTVRDEGVGIPDIEEARQPLFTTKPELERSGMGFTIMENFMDEVIVESEVNKGTTVYLKKHIVKSKALSN

22222222222222222222222222222222222222222222212221122112222222222222222222222222122211222221222221122222111111222222222222222222212222222222222222

>3E1YA

MRGSHHHHHHGMASMADDPSAADRNVEIWKIKKLIKSLEAARGNGTSMISLIIPPKDQISRVAKMLADEFGTASNIKSRVNRLSVLGAITSVQQRLKLYNKVPPNGLVVYCGTIVTEEGKEKKVNIDFEPFKPINTSLYLCDNKFHTEALTALLSDDSKFGFIVIDGSGALFGTLQGNTREVLHKFTVDLPKKHGRGGQSALRFARLRMEKRHNYVRKVAETAVQLFISGDKVNVAGLVLAGSADFKTELSQSDMFDQRLQSKVLKLVDISYGGENGFNQAIELSTEVLSNVKFIQEKKLIGRYFDEISQDTGKYCFGVEDTLKALEMGAVEILIVYENLDIMRYVLHCQGTEEEKILYLTPEQEKDKSHFTDKETGQEHELIESMPLLEWFANNYKKFGATLEIVTDKSQEGSQFVKGFGGIGGILRYRVDFQGMEYQGGDDEFFDLDDY

2222222222222222222222222222222222222222222222222222222222222222222212211221222222221222222222222222222222222222222222222222222222212212222222222222222222222222222222222222222222222222222222222222222222222222222222222222222222222222222222222222222222222222222222222222222222222222222222222222222222222222222222222222222222222222222222222222222222222222222222222222222222222222222222222222222222222222222222222222222222222222222222222222222222222222222

>1O93A

MNGPVDGLCDHSLSEEGAFMFTSESVGEGHPDKICDQISDAVLDAHLKQDPNAKVACETVCKTGMVLLCGEITSMAMIDYQRVVRDTIKHIGYDDSAKGFDFKTCNVLVALEQQSPDIAQCVHLDRNEEDVGAGDQGLMFGYATDETEECMPLTIVLAHKLNTRMADLRRSGVLPWLRPDSKTQVTVQYVQDNGAVIPVRVHTIVISVQHNEDITLEAMREALKEQVIKAVVPAKYLDEDTIYHLQPSGRFVIGGPQGDAGVTGRKIIVDTYGGWGAHGGGAFSGKDYTKVDRSAAYAARWVAKSLVKAGLCRRVLVQVSYAIGVAEPLSISIFTYGTSKKTERELLEVVNKNFDLRPGVIVRDLDLKKPIYQKTACYGHFGRSEFPWEVPKKLVF

222222222222222222222222222221122222222222222222222222222222222222222222222222222222222222222222222222222222222222222222222222222222222222222222222222222222222222222222222222222221212222222222222222222222222222222222222222222222222222222222222222222222222221122222212222222222222222222222222222222222222222222222222222222222222222222222222222222222222222222222222222222222222222222222222222222222

>3A8TA

MRGSHHHHHHMDYASVAMAAAPTTTTTTNVSLRRQRHRKEKLLVLMGATGTGKSRLSIDLAAHFPLEVINSDKMQVYKGLDITTNKISVPDRGGVPHHLLGEVDPARGELTPADFRSLAGKAVSEITGRRKLPVLVGGSNSFIHALLVDRFDSSGPGVFEEGSHSVVSSELRYDCCFLWVDVSVKVLTDYLAKRVDDMLELGMFDELAEFYSPEDEDHDEDSATRTGLRKAIGVPEFDRYFEKFRPGDVEGEDPGRDRVRRGAFEEAVRAIKENTCHLAKRQIGKILRLKGAGWDLRRLDATESFRAAMTSDSGEKCTEIWEKQVLEPSVKIVSRFLDE

222222222222222222222222222222222222222222222222222222222222222222222221112222222211122222222222222222222222222222222222222222222222222221111222222222222222222222222222222222222222222222222222222222222222222222222222222221222222211122222222222222222222222222222222222222222222212221221222222222222222222222222222222222222222222222222222222

>3EA0A

SNAKRVFGFVSAKGGDGGSCIAANFAFALSQEPDIHVLAVDISLPFGDLDUYLSGNTHSQDLADISNASDRLDKSLLDTUVQHISPSLDLIPSPATFEKIVNIEPERVSDLIHIAASFYDYIIVDFGASIDHVGVWVLEHLDELCIVTTPSLQSLRRAGQLLKLCKEFEKPISRIEIILNRADTNSRITSDEIEKVIGRPISKRIPQDEDAUQESLLSGQSVLKVAPKSQLSKTIVDWALHLNGV

22222222221211111111222222222222222222222222222122212222222222222222222222222222222222222222222222222222222222222222222222221222222222222222222222222222122222222222222222222222222112222222222222222222222221111222222122222222222222222222222222222

>1NGEA

MSKGPAVGIDLGTTYSCVGVFQHGKVEIIANDQGNRTTPSYVAFTDTERLIGDAAKNQVAMNPTNTVFDAKRLIGRRFDDAVVQSDMKHWPFMVVNDAGRPKVQVEYKGETKSFYPEEVSSMVLTKMKEIAEAYLGKTVTNAVVTVPAYFNDSQRQATKDAGTIAGLNVLRIINEPTAAAIAYGLDKKVGAERNVLIFSLGGGTFDVSILTIEDGIFEVKSTAGDTHLGGEDFDNRMVNHFIAEFKRKHKKDISENKRAVRRLRTACERAKRTLSSSTQASIEIDSLYEGIDFYTSITRARFEELNADLFRGTLDPVEKALRDAKLDKSQIHDIVLVGGSTRIPKIQKLLQDFFNGKELNKSINPDEAVAYGAAVQAAILSGDKSE

22222222222111122222222222222222222222222222222222222222222222222222222222222222222222222222222222222222222222222222222222222222222222222222222222222222222222222222222222222222222222222222222222222222111122222222222222222222222221222222222222222222222222222222222222212211221222222222222222222222222222222222222222222222222222222222222221112112222222222222222222222122222222222222222222

>2NVUB

MKLMKIEEGKLVIWINGDKGYNGLAEVGKKFEKDTGIKVTVEHPDKLEEKFPQVAATGDGPDIIFWAHDRFGGYAQSGLLAEITPDKAFQDKLYPFTWDAVRYNGKLIAYPIAVEALSLIYNKDLLPNPPKTWEEIPALDKELKAKGKSALMFNLQEPYFTWPLIAADGGYAFKYENGKYDIKDVGVDNAGAKAGLTFLVDLIKNKHMNADTDYSIAEAAFNKGETAMTINGPWAWSNIDTSKVNYGVTVLPTFKGQPSKPFVGVLSAGINAASPNKELAKEFLENYLLTDEGLEAVNKDKPLGAVALKSYEEELAKDPRIAATMENAQKGEIMPNIPQMSAFWYAVRTAVINAASGRQTVDAALAAAQTNAAADWEGRWNHVKKFLERSGPFTHPDFEPSTESLQFLLDTCKVLVIGAGGLGCELLKNLALSGFRQIHVIDMDTIDVSNLNRQFLFRPKDIGRPKAEVAAEFLNDRVPNCNVVPHFNKIQDFNDTFYRQFHIIVCGLDSIIARRWINGMLISLLNYEDGVLDPSSIVPLIDGGTEGFKGNARVILPGMTACIECTLELYPPQVNFPMCTIASMPRLPEHCIEYVRMLQWPKEQPFGEGVPLDGDDPEHIQWIFQKSLERASQYNIRGVTYRLTQGVVKRIIPAVASTNAVIAAVCATEVFKIATSAYIPLNNYLVFNDVDGLYTYTFEAERKENCPACSQLPQNIQFSPSAKLQEVLDYLTNSASLQMKSPAITATLEGKNRTLYLQSVTSIEERTRPNLSKTLKELGLVDGQELAVADVTTPQTVLFKLHFTS

2222222222222222222222222222222222222222222222222222222222222222222222222222222222222222222222222222222222222222222222222222222222222222222222222222222222222222222222222222222222222222222222222222222222222222222222222222222222222222222222222222222222222222222222222222222222222222222222222222222222222222222222222222222222222222222222222222222222222222222222222222222222222222222222222222222222222222222222222222222222112222222222222222222221112222212211222222222221222222222222222222222111122222222222222211122222222222222222222222222222222222222222222222222222222222222222222222222222222222222222222222222222222222222222222222222222222222222222222222222222222222222222222222222222222222222222222222222222222222222222222222222222222222222222222222222222222222222222222222222222222222222222222222222222222

>2IDXA

MPKIYTKTGDKGFSSTFTGERRPKDDQVFEAVGTTDELSSAIGFALELVTEKGHTFAEELQKIQCTLQDVGSALATPCSSAREAHLKYTTFKAGPILELEQWIDKYTSQLPPLTAFILPSGGKISSALHFCRAVCRRAERRVVPLVQMGETDANVAKFLNRLSDYLFTLARYAAMKEGNQEKIYMKNDPSAESEGL

2222222222222222222222222222222222222222222222222222222222222222222222222222222222222222222222222222222222222222222222222222222222222221221122222222222222222221222222222222222222222222222222222222

>1A82A

SKRYFVTGTDTEVGKTVASCALLQAAKAAGYRTAGYKPVASGSEKTPEGLRNSDALALQRNSSLQLDYATVNPYTFAEPTSPHIISAQEGRPIESLVMSAGLRALEQQADWVLVEGAGGWFTPLSDTFTFADWVTQEQLPVILVVGVKLGCINHAMLTAQVIQHAGLTLAGWVANDVTPPGKRHAEYMTTLTRMIPAPLLGEIPWLAENPENAATGKYINLALL

22222222221111111222222222222222222212222222222222222122222222222222222222222222222222222222222222222222222222222212212222222222222222222222222222222222222222222222222222222211222222222222222222222222221111222112222222222222

>1TYQB

MDSQGRKVVVCDNGTGFVKCGYAGSNFPEHIFPALVGRPIIRSTTKVGNIEIKDLMVGDEASELRSMLEVNYPMENGIVRNWDDMKHLWDYTFGPEKLNIDTRNCKILLTEPPMNPTKNREKIVEVMFETYQFSGVYVAIQAVLTLYAQGLLTGVVVDSGDGVTHICPVYEGFSLPHLTRRLDIAGRDITRYLIKLLLLRGYAFNHSADFETVRMIKEKLCYVGYNIEQEQKLALETTVLVESYTLPDGRIIKVGGERFEAPEALFQPHLINVEGVGVAELLFNTIQAADIDTRSEFYKHIVLSGGSTMYPGLPSRLERELKQLYLERVLKGDVEKLSKFKIRIEDPPRRKHMVFLGGAVLADIMKDKDNFWMTRQEYQEKGVRVLEKLGVTVR

2222222222222222222222222222222222222222222222222222222222222222222222222222222222222222222222222222222222222222222222222222222222222222222222222222222222222221111222222222222222222222212222222222222222222222222221221122222222222222222222222222222222222222222222222222222222222222222222222222222222222222111211222222222222222222222222222222222222222222222222222222222222222222222222222222222222

>2QRDG

AMDVQETQKGALKEIQAFIRSRTSYDVLPTSFRLIVFDVTLFVKTSLSLLTLNNIVSAPLWDSEANKFAGLLTMADFVNVIKYYYQSSSFPEAIAEIDKFRLLGLREVERKIGAIPPETIYVHPMHSLMDACLAMSKSRARRIPLIDVDGETGSEMIVSVLTQYRILKFISMNCKETAMLRVPLNQMTIGTWSNLATASMETKVYDVIKMLAEKNISAVPIVNSEGTLLNVYESVDVMHLIQDGDYSNLDLSVGEALLKRPANFDGVHTCRATDRLDGIFDAIKHSRVHRLFVVDENLKLEGILSLADILNYIIYDKTTTPGVPEQTDNFESAV

2222222222222222222222222222222222222222222222222222222222222222222222222222222222222222222222222222222222222222222222222222222222222222222212222222222222222222222222222222222222222222222222122111222222222222222222211121222222222222222222222222222222222222222222222222222222222222222222222122222222222212121122222222222222222222222222

>1X01A

MLGLKTSIIGRRVIYFQEITSTNEFAKTSYLEEGTVIVADKQTMGHGRLNRKWESPEGGLWLSIVLSPKVPQKDLPKIVFLGAVGVVETLKEFSIDGRIKWPNDVLVNYKKIAGVLVEGKGDKIVLGIGLNVNNKVPNGATSMKLELGSEVPLLSVFRSLITNLDRLYLNFLKNPMDILNLVRDNMILGVRVKILGDGSFEGIAEDIDDFGRLIIRLDSGEVKKVIYGDVSLRFL

2222222222222222222222222222222222222222222222111211112222222222222222222222222222222222222222222221221122222212222222222222222222122222122122222222222222222222222222222222222222222222222222222222222222222222222222222222222222222222222

>3H0RE

MNEKYEAVIGLEIHVQMDTKTKMFCGCKVEFGAEPNTNVCPVCLGMPGALPIVNKRAVEYAIRASLALNCEVHEESVFARKHYFYPDLPKGYQISQYEKPLATNGWVELNLPNGEKKKVRIRRLHIEEDAGKNIHEGDKTLVDLNRAGTPLMEIVTEPDIRTPEEARLFLEKLRNIMRYAGVSKADMEKGQLRCDINVSIRPKGSKEFGTRVEIKNVNSFRFVQKALEYEIERQINVVEEGGEVVQETRTFDPQTGKTYPMRTKEEAEDYRYFPDPDLVPLKVKKEWIEEIKKNMPELPDQRFERLIKEYGLSEYEAGILVNHKEVGDFFEEAVRHFKEPKGIVNWLINDLLGLLRDKGISIEESPVKPEHLAELVKLIKEKVISTKIGKEVIKEMVETGKTPSQIVEEKGLKQITDENQIKELVKKIFEKHPKEVERLKQGEEKLIGFFVGQVMRETRGKANPQVVNKVIRELVKEV

2222222121112222222222222222222222222222222222222222222222222222222222222222222222222222222222222222222222222222222222222222222222222222222222222222222222111122222222222222222222222222222222222222111222222221121222222222222222222222222222222222222222222222222222222222222222222222222222222222222222222222222222222222222222222222222222222222222222222222222222222222222222222222222222222222222222222222222222222222222222222222222222222222222222222222222222222222222222222222222222

>1J1ZA

MKIVLAYSGGLDTSIILKWLKETYRAEVIAFTADIGQGEEVEEAREKALRTGASKAIALDLKEEFVRDFVFPMMRAGAVYEGYYLLGTSIARPLIAKHLVRIAEEEGAEAIAHGATGKGNDQVRFELTAYALKPDIKVIAPWREWSFQGRKEMIAYAEAHGIPVPVTQEKPYSMDANLLHISYEGGVLEDPWAEPPKGMFRMTQDPEEAPDAPEYVEVEFFEGDPVAVNGERLSPAALLQRLNEIGGRHGVGRVDIVENRFVGMKSRGVYETPGGTILYHARRAVESLTLDREVLHQRDMLSPKYAELVYYGFWYAPEREALQAYFDHVARSVTGVARLKLYKGNVYVVGRKAPKSLYRQDLVSFDEAGGYDQKDAEGFIKIQALRLRVRALVEREGHGA

2222211122211222222222222222221112221222222222222222222222222222222222222222222222222222222122122222222222222222111222222222122222222222222222222222222222222222222222222222111222222222222222222222222222222222222222222222222222222222222222222222222222222222222222222222222222222222222222222222222222222222222222222222222222222222222222222222222222222222222222222222222222222222222222222222222222222222

>2IAJA

PISPIETVPVKLKPGMDGPKVKQWPLTEEKIKALVEICTEMEKEGKISKIGPENPYNTPVFAIKKKDSTKWRKLVDFRELNKRTQDFWEVQLGIPHPAGLKKNKSVTVLDVGDAYFSVPLDEDFRKYTAFTIPSINNETPGIRYQYNVLPQGWKGSPAIFQSSMTKILEPFKKQNPDIVICQYMDDLYVGSDLEIGQHRTKIEELRQHLLRWGLTTPDKKHQKEPPFLWMGYELHPDKWTVQPIVLPEKDSWTVNDIQKLVGKLNWASQIYPGIKVRQLSKLLRGTKALTEVIPLTEEAELELAENREILKEPVHGVYYDPSKDLIAEIQKQGQGQWTYQIYQEPFKNLKTGKYARMRGAHTNDVKQLTEAVQKITTESIVIWGKTPKFKLPIQKETWETWWTEYWQATWIPEWEFVNTPPLVKLWYQLEKEPIVGAETFYVDGAANRETKLGKAGYVTNKGRQKVVPLTNTTNQKTELQAIYLALQDSGLEVNIVTDSQYALGIIQAQPDKSESELVNQIIEQLIKKEKVYLAWVPAHKGIGGNEQVDKLVSAGIRKIL

22222222222222222222222222222222222222222222222222222222222222222222222222222222222222222222222222222222222221111112222222222222222222222222222222222212222222222222222222222222222222211122222222222222222222222222222222122222222222222222222222222222222222222222222222222222222222222222222222222222222222222222222222222222222222222222222222222222222222222222222222222222222222222222222222222222222222222222222222222222222222222222222222222222222222222222222222222222222222222222222222222222222222222222222222222222222222222222222222222222222222222222222222222222

>1TWAB

MSDLANSEKYYDEDPYGFEDESAPITAEDSWAVISAFFREKGLVSQQLDSFNQFVDYTLQDIICEDSTLILEQLAQHTTESDNISRKYEISFGKIYVTKPMVNESDGVTHALYPQEARLRNLTYSSGLFVDVKKRTYEAIDVPGRELKYELIAEESEDDSESGKVFIGRLPIMLRSKNCYLSEATESDLYKLKECPFDMGGYFIINGSEKVLIAQERSAGNIVQVFKKAAPSPISHVAEIRSALEKGSRFISTLQVKLYGREGSSARTIKATLPYIKQDIPIVIIFRALGIIPDGEILEHICYDVNDWQMLEMLKPCVEDGFVIQDRETALDFIGRRGTALGIKKEKRIQYAKDILQKEFLPHITQLEGFESRKAFFLGYMINRLLLCALDRKDQDDRDHFGKKRLDLAGPLLAQLFKTLFKKLTKDIFRYMQRTVEEAHDFNMKLAINAKTITSGLKYALATGNWGEQKKAMSSRAGVSQVLNRYTYSSTLSHLRRTNTPIGRDGKLAKPRQLHNTHWGLVCPAETPEGQACGLVKNLSLMSCISVGTDPMPIITFLSEWGMEPLEDYVPHQSPDATRVFVNGVWHGVHRNPARLMETLRTLRRKGDINPEVSMIRDIREKELKIFTDAGRVYRPLFIVEDDESLGHKELKVRKGHIAKLMATEYQDIEGGFEDVEEYTWSSLLNEGLVEYIDAEEEESILIAMQPEDLEPAEANEENDLDVDPAKRIRVSHHATTFTHCEIHPSMILGVAASIIPFPDHNQSPRNTYQSAMGKQAMGVFLTNYNVRMDTMANILYYPQKPLGTTRAMEYLKFRELPAGQNAIVAIACYSGYNQEDSMIMNQSSIDRGLFRSLFFRSYMDQEKKYGMSITETFEKPQRTNTLRMKHGTYDKLDDDGLIAPGVRVSGEDVIIGKTTPISPDEEELGQRTAYHSKRDASTPLRSTENGIVDQVLVTTNQDGLKFVKVRVRTTKIPQIGDKFASRHGQKGTIGITYRREDMPFTAEGIVPDLIINPHAIPSRMTVAHLIECLLSKVAALSGNEGDASPFTDITVEGISKLLREHGYQSRGFEVMYNGHTGKKLMAQIFFGPTYYQRLRHMVDDKIHARARGPMQVLTRQPVEGRSRDGGLRFGEMERDCMIAHGAASFLKERLMEASDAFRVHICGICGLMTVIAKLNHNQFECKGCDNKIDIYQIHIPYAAKLLFQELMAMNITPRLYTDRSRDF

222222222222222222222222222222222222222222222222222222222222222222222222222222222222222222222222222222222222222222222222222222222222222222222222222222222222222222222222222222222222222222222222222222222222222222222222222222222222222222222222222222222222222222222222222222222222222222222222222222222222222222222222222222222222222222222222222222222222222222222222222222222222222222222222222222222222222222222222222222222222222222222222222222222222222222222222222222222222222222222222222222222222222222222222222222222222222222222222222222222222222222222222222222222222222222222222222222222222222222222222222222222222222222222222222222222222222222222222222222222222222222222222222222222222222222222222222222222222222222222222222222222222222222222222222222222222222222222122222222222222222222222222222222222222222222222222222222222222222222221222222222222222222222222222222222222222222222222222222222222222222222222222222222222222222222222222222222222222222222222222222222222222222222222222211222222222222222222222222222222221222222222222222222222222222222222222222222222222222222222222222222222222222222222222222222222222222222222222222222222222222222222222222222222222222222222222222222222222222222222222222222222222222222222222

>1GN8A

MQKRAIYPGTFDPITNGHIDIVTRATQMFDHVILAIAASPSKKPMFTLEERVALAQQATAHLGNVEVVGFSDLMANFARNQHATVLIRGLRAVADFEYEMQLAHMNRHLMPELESVFLMPSKEWSFISSSLVKEVARHQGDVTHFLPENVHQALMAKLA

222222121112222211221222222222222222222222222222222222222222222222222222222222222222222112122222222222222222222222222221222122111112222222222222222222222222222

>2IJMA

GASTRDYEIQRERIELGRCIGEGQFGDVHQGIYMSPENPALAVAIKTCKNCTSDSVREKFLQEALTMRQFDHPHIVKLIGVITENPVWIIMELCTLGELRSFLQVRKYSLDLASLILYAYQLSTALAYLESKRFVHRDIAARNVLVSSNDCVKLGDFGLSRYMEDSTYYKASKGKLPIKWMAPESINFRRFTSASDVWMFGVCMWEILMHGVKPFQGVKNNDVIGRIENGERLPMPPNCPPTLYSLMTKCWAYDPSRRPRFTELKAQLSTILEEEKAQQEE

22222222222222222221111122212222222222222221212222222222222222122222222222222222222222222211112221222222222222222222222222222222222222222222221212222222222112122222222222222222222222222222222222222222222222222222222222222222222222222222222222222222222222222222222222222222222222222

>1YFRA

MSLSAHEIRELFLSFFEKKGHTRVKSAPLVPENDPTLLFVNAGMVPFKNVFLGLEKRPYKRATSCQKCLRVSGKHNDLEQVGYTSRHHTFFEMLGNFSFGDYFKKEAIEYAWEFVTEVLKLPKEKLYVSVYKDDEEAYRIWNEHIGIPSERIWRLGEEDNFWQMGDVGPCGPSSEIYVDRGEEYEGDERYLEIWNLVFMQYNRDENGVLTPLPHPNIDTGMGLERIASVLQGKNSNFEIDIIFPLIQFGEEVSGKKYGEKFETDVALRVIADHLRAITFAISDGVIPSNEGRGYVIRRILRRAMRFGYKLGIENPFLYKGVDLVVDIMKEPYPELELSREFVKGIVKGEEKRFIKTLKAGMEYIQEVIQKALEEGRKTLSGKEVFTAYDTYGFPVDLIDEIAREKGLGIDLEGFQCELEEQRERARKHFKVEAKKVKPVYSHLKELGKTSAFVGAAALEHHHHHH

222222222222222222222222222222222222222222222222222222222222222222222222222222222222211122221222222222222222222222222222222222222222222222222222222222222222222222222222222222122222222222222221221222222222222222222222222121221222222222212222222222222222222222222222222222222222222222222222222222222222222222222222222222222222222222222222222222222222222222222222222222222222222222222222222222222222222222222222222222222222222222222222222222222222222222222222222222222

>2RD5C

MQISSDYIPDSKFYKVEAIVRPWRIQQVSSALLKIGIRGVTVSDVRGFGAQGGSTERHGGSEFSEDKFVAKVKMEIVVKKDQVESVINTIIEGARTGEIGDGKIFVLPVSDVIRVRTGERGEKAEKMTGDMLSPS

222222222222222222122222222222222222222222222211121222222222222222222212222222222222222222222222221121121222222222222222222222222222222

>3R1RA

MNQNLLVTKRDGSTERINLDKIHRVLDWAAEGLHNVSISQVELRSHIQFYDGIKTSDIHETIIKAAADLISRDAPDYQYLAARLAIFHLRKKAYGQFEPPALYDHVVKMVEMGKYDNHLLEDYTEEEFKQMDTFIDHDRDMTFSYAAVKQLEGKYLVQNRVTGEIYESAQFLYILVAACLFSNYPRETRLQYVKRFYDAVSTFKISLPTPIMSGVRTPTRQFSSCVLIECGDSLDSINATSSAIVKYVSQRAGIGINAGRIRALGSPIRGGEAFHTGCIPFYKHFQTAVKSCSQGGVRGGAATLFYPMWHLEVESLLVLKNNRGVEGNRVRHMDYGVQINKLMYTRLLKGEDITLFSPSDVPGLYDAFFADQEEFERLYTKYEKDDSIRKQRVKAVELFSLMMQERASTGRIYIQNVDHCNTHSPFDPAIAPVRQSNLCLEIALPTKPLNDVNDENGEIALCTLSAFNLGAINNLDELEELAILAVRALDALLDYQDYPIPAAKRGAMGRRTLGIGVINFAYYLAKHGKRYSDGSANNLTHKTFEAIQYYLLKASNELAKEQGACPWFNETTYAKGILPIDTYKKDLDTIANEPLHYDWEALRESIKTHGLRNSTLSALMPSETSSQISNATNGIEPPRGYVSIKASKDGILRQVVPDYEHLHDAYELLWEMPGNDGYLQLVGIMQKFIDQSISANTNYDPSRFPSGKVPMQQLLKDLLTAYKFGVKTLYYQNTRDGAEDAQDDLVPSIQDDGCESGACKI

22222222122222111122112212222222222222222222222222222212221222222222222222222222222222222212222222222222222222222222222222222222222222222222222222222222222222222222222222222222222222222222222222222222222222222222222222222222222222222222222222222222222222222222222222222222222222222222222222222222222222222222222222222222222222222222222222222222222222222222222222222222222222222222222222222222222222222222222222222222222222222222222222222222222222222222222222222222222222222222222222222222222222222222222222222222222222222222222222222222222222222222222222222222222222222222222222222222222222222222222222222222222222222222222222222222222222222222222222222222222222222222222222222222222222222222222222222222222222222222222222222222222222222222222222222222222222222

>2DDOA

MSSLLLFNDKSRALQADIVAVQSQVVYGSVGNSIAVPAIKQNGLNVFAVPTVLLSNTPHYDTFYGGAIPDEWFSGYLRALQERDALRQLRAVTTGYMGTASQIKILAEWLTALRKDHPDLLIMVDPVIGDIDSGIYVKPDLPEAYRQYLLPLAQGITPNIFELEILTGKNCRDLDSAIAAAKSLLSDTLKWVVVTSASGNEENQEMQVVVVTADSVNVISHSRVKTDLKGTGDLFCAQLISGLLKGKALTDAVHRAGLRVLEVMRYTQQHESDELILPPLAEA

2222222222222222222222222222222222222222222222222222222222222222222222222222222222222222222222222222222222222222222222222222122222222222222222222222222222221212212222222222222222222222222222222212222222222122222222222222111122222211221222222222222222222222122122212222222222222222222

>2P09A

GSMDYKDDDDKKTNWLKRIYRVRPCVKCKVAPRDWKVKNKHLRIYNMCKTCFNNSIDIGDDTYHGHVDWLMYADSKEISNT

222222222222222222222222222222222121212222121111222122222222221111222222222222222

>1XMIA

STTEVVMENVTAFWEEGFGELFEKAKQNNNNRKTSNGDDSLSFSNFSLLGTPVLKDINFKIERGQLLAVAGSTGAGKTSLLMMIMGELEPSEGKIKHSGRISFCSQFSWIMPGTIKENIIAGVSYDEYRYRSVIKACQLEEDISKFAEKDNIVLGEGGITLSGGQRARISLARAVYKDADLYLLDSPFGYLDVLTEKEIFESCVCKLMANKTRILVTSKMEHLKKADKILILHEGSSYFYGTFSELQNLQPDFSSKLMGCDSFDQFSAERRNSILTETLRRFSLEGDAPVS

222222222222212222222222222222222222222222222222222212222222222222222222111111122222222222222222222222222122222222222222222222222222222222222222222222222222222222222222222222222222222222222222222222222222222222222222222222222222222222222222222222222222222222222222222222222222222222222222222

>2VHQA

MIHLYDAKSFAKLRAAQYAAFHTDAPGSWFDHTSGVLESVEDGTPVLAIGVESGDAIVFDKNAQRIVAYKEKSVKAEDGSVSVVQVENGFMKQGHRGWLVDLTGELVGCSPVVAEFGGHRYASGMVIVTGKGNSGKTPLVHALGEALGGKDKYATVRFGEPLSGYNTDFNVFVDDIARAMLQHRVIVIDSLKNVIGAAGGNTTSGGISRGAFDLLSDIGAMAASRGCVVIASLNPTSNDDKIVELVKEASRANSTSLVISTDVDGEWQVLTRTGEGLQRLTHTLQTSYGEHSVLTIHTSKQSGGKQASGKAIQTVIKNDELESVLRRLTSN

2222222222222222222222222222222222222222222222222222222222222222222222222222222222222222222222222222222222222222222222222222222222222211112222222222222222222222222222222222222222222222222222222222222222222222222222222222222222222222212222222222222222222222222222222222222122211112222222212221222222222222222222222222212222222222222

>2CBZA

MNSITVRNATFTWARSDPPTLNGITFSIPEGALVAVVGQVGCGKSSLLSALLAEMDKVEGHVAIKGSVAYVPQQAWIQNDSLRENILFGCQLEEPYYRSVIQACALLPDLEILPSGDRTEIGEKGVNLSGGQKQRVSLARAVYSNADIYLFDDPLSAVDAHVGKHIFENVIGPKGMLKNKTRILVTHSMSYLPQVDVIIVMSGGKISEMGSYQELLARDGAFAEFLRTYASHHHHHH

222222222222122222212222222222222222222111111122222222222222222222222222122222222222222222222222222222222222222222222222222222222222222222222222222222222222222222222222222222222222222222222222222222222222222222222222222222222222222222222

>2JK8A

MPKAKAKTKNTEIISPHHYVYPNTTTLKNKYGIKNLNAFLEKCSHDTAKAMINLREESLPEYFDTAYLCHIHQQLFKNTFEWAGYLRHIPFTFADGTTAAMPEMKRTGWKNAFAIGDEIQEGLQRLDQTLAEKNNLQGLTREEFNSEAIELFNSLNQLHPFREGNGRTQRLFFENLAKAAGHQLNFSLITKERMMVASVAVAENGDLEPMQHLFEDISNPEKIRLLKEFMHTMKNTGRNVNDRPVMVAKEGETYTGTYRGAGLEGFALNVKGAYIIGNIDHLPPEQLKILKPGDKITFTAPK

22222222222222222222222222222222222222222222222222222222222222222222222222222222222222222222222222222222222222222222222222222222222222222222222222222222222222222211111222222222222222222222222222222222222222222222222222222222222222222222222222222222222222222222222222222222222222222222222222222222222222

>3BJUA

MSVDPNQYYKIRSQAIHQLKVNGEDPYPHKFHVDISLTDFIQKYSHLQPGDHLTDITLKVAGRIHAKRASGGKLIFYDLRGEGVKLQVMANSRNYKSEEEFIHINNKLRRGDIIGVQGNPGKTKKGELSIIPYEITLLSPCLHMLPHLHFGLKDKETRYRQRYLDLILNDFVRQKFIIRSKIITYIRSFLDELGFLEIETPMMNIIPGGAVAKPFITYHNELDMNLYMRIAPELYHKMLVVGGIDRVYEIGRQFRNEGIDLTHNPEFTTCEFYMAYADYHDLMEITEKMVSGMVKHITGSYKVTYHPDGPEGQAYDVDFTPPFRRINMVEELEKALGMKLPETNLFETEETRKILDDICVAKAVECPPPRTTARLLDKLVGEFLEVTCINPTFICDHPQIMSPLAKWHRSKEGLTERFELFVMKKEICNAYTELNDPMRQRQLFEEQAKAKAAGDDEAMFIDENFCTALEYGLPPTAGWGMGIDRVAMFLTDSNNIKEVLLFPAMKPEDKKENLEHHHHHH

22222222222222222222222222222222222222222222222222222222222222222222222222222222222222222222222222222222222222222222222222222222222222222222222222222222222222222222222222222222222222222222222222222222222222222222222222222222222222222222222222222222222222121222211122121222222222222222222222222222222222222222222222222222222222222222222222222222222222222222222222222222222222222222222222222222222222222222222222222222222222222112122222222222222222222222222222222222222222222222222221221222222222212222222222222222222222222

>1Y8QB

MALSRGLPRELAEAVAGGRVLVVGAGGIGCELLKNLVLTGFSHIDLIDLDTIDVSNLNRQFLFQKKHVGRSKAQVAKESVLQFYPKANIVAYHDSIMNPDYNVEFFRQFILVMNALDNRAARNHVNRMCLAADVPLIESGTAGYLGQVTTIKKGVTECYECHPKPTQRTFPGATIRNTPSEPIHCIVWAKYLFNQLFGEEDADQEVSPDRADPEAAWEPTEAEARARASNEDGDIKRISTKEWAKSTGYDPVKLFTKLFKDDIRYLLTMDKLWRKRKPPVPLDWAEVQSQGEETNASDQQNEPQLGLKDQQVLDVKSYARLFSKSIETLRVHLAEKGDGAELIWDKDDPSAMDFVTSAANLRMHIFSMNMKSRFDIKSMAGNIIPAIATTNAVIAGLIVLEGLKILSGKIDQCRTIFLNKQPNPRKKLLVPCALDPPNPNCYVCASKPEVTVRLNVHKVTVLTLQDKIVKEKFAMVAPDVQIEDGKGTILISSEEGETEANNHKKLSEFGIRNGSRLQADDFLQDYTLLINILHSEDLGKDVEFEVVGDAPEKVGPKQAEDAAKSITNGSDDGAQPSTSTAQEQDDVLIVDSDEEDSSNNADVSEEERSRKRKLDEKENLSAKRSRIEQKEELDDVIALD

2222222222222222222222222112222222222222222222211122222122112222222222212222222222222222222221111222222222222222221111221222222222222222222222222222222222222222222222222222222222222222222222222222222222222222222222222222222222222222222222222222222222222222222222222222222222222222222222222222222222222222222222222222222222222222222222222222222221222222222222222222222222222222222222222222222222222222222222222222222222222222222222222222222222222222222222222222222222222222222222222222222222222222222222222222222222222222222222222222222222222222222222222222222222222222222222222222222222222222222222222222222222222222222222222222222222222222

>2ZHZA

MGNRLSKIATRTGDDGTTGLGDGSRVRKDDARIAAIGDVDELNSQIGVLLAEPLPDDVRAALSAIQHDLFDLGGELCIPGHAAITDAHLARLDGWLAHYNGQLPPLEEFILPGGARGAALAHVCRTVCRRAERSIVALGASEPLNAAPRRYVNRLSDLLFVLARVLNRAAGGADVLWDRTRAH

222222222222222222222222222222222222222222222222222222222222222222222222222222222222222222222222222222222222222222222222222222221221122222222222222222221222122222222222222222222222222

>2IVPA

MLALGIEGTAHTLGIGIVSEDKVLANVFDTLTTEKGGIHPKEAAEHHARLMKPLLRKALSEAGVSLDDIDVIAFSQGPGLGPALRVVATAARALAVKYRKPIVGVNHCIAHVEITKMFGVKDPVGLYVSGGNTQVLALEGGRYRVFGETLDIGIGNAIDVFARELGLGFPGGPKVEKLAEKGEKYIELPYAVKGMDLSFSGLLTEAIRKYRSGKYRVEDLAYSFQETAFAALVEVTERAVAHTEKDEVVLVGGVAANNRLREMLRIMTEDRGIKFFVPPYDLCRDNGAMIAYTGLRMYKAGISFRLEETIVKQKFRTDEVEIVWHHHHHH

222222221122222222222222222222222222222222222222222222222222222222222222222222222222222222222222222222222212222222222222222222121111222222222222222222222212221222222222222112212222222222222222222222222222222222222222222222222222222222222222222222222222112112222222222222222222222122211222222222222222222222222222222222222222222222

>3DNTA

UPKLVTWUNNQRVGELTKLANGAHTFKYAPEWLASRYARPLSLSLPLQRGNITSDAVFNFFDNLLPDSPIVRDRIVKRYHAKSRQPFDLLSEIGRDSVGAVTLIPEDETVTHPIUAWEKLTEARLEEVLTAYKADIPLGUIREENDFRISVAGAQEKTALLRIGNDWCIPKGITPTTHIIKLPIGEIRQPNATLDLSQSVDNEYYCLLLAKELGLNVPDAEIIKAGNVRALAVERFDRRWNAERTVLLRLPQEDUCQTFGLPSSVKYESDGGPGIARIUAFLUGSSEALKDRYDFUKFQVFQWLIGATQGHAKNFSVFIQAGGSYRLTPFYDIISAFPVLGGTGIHISDLKLAUGLNASKGKKTAIDKIYPRHFLATAKVLRFPEVQUHEILSDFARUIPAALDNVKTSLPTDFPENVVTAVESNVLRLHGRLSREYGSK

22222222222222222222222222222222222222222222222222222222222222222212222222222222222222222222222221222222222222222222222222222222222222222222222222222221111212222222222222222222221212222222222222222222222222222222222221222222222222221111122222222222222122222222222222222222222222222222222222222222222222222222221211222222222222222211222222222222222222222222222222222222222222222222222222222222222222222222222222222222222222222222222222222222

>1XNGA

MQKDYQKLIVYLCDFLEKEVQKRGFKKVVYGLSGGLDSAVVGVLCQKVFKENAHALLMPSSVSMPENKTDALNLCEKFSIPYTEYSIAPYDAIFSSHFKDASLTRKGNFCARLRMAFLYDYSLKSDSLVIGTSNKSERMLGYGTLFGDLACAINPIGELFKTEVYELARRLNIPKKILNKPPSADLFVGQSDEKDLGYPYSVIDPLLKDIEALFQTKPIDTETLAQLGYDEILVKNITSRIQKNAFKLELPAIAKRFNPELEHHHHHH

2222222222222222222222222222221112111122222222222222222111122222222222222222222222222222222222222222222222222222212222222222222222212222122222222221222222222222122222222222222222222111222222222222222222222222222222222222222222222222222222222222222222222222222222222222

>1XEXA

MPYIEKLELKGFKSYGNKKVVIPFSKGFTAIVGANGSGKSNIGDAILFVLGGLSAKAMRASRISDLIFAGSKNEPPAKYAEVAIYFNNEDRGFPIDEDEVVIRRRVYPDGRSSYWLNGRRATRSEILDILTAAMISPDGYNIVLQGDITKFIKMSPLERRLLIDDISGIAEYDSKKEKALEE

22222222222211222222222222222222211111111222222222222222221222221211122222222222222222222222222222222222222222222222222222222222222222222222222212222222222222222222222222222222222222

>1G5TA

MSDERYQQRQQKVKDRVDARVAQAQEERGIIIVFTGNGKGKTTAAFGTAARAVGHGKNVGVVQFIKGTWPNGERNLLEPHGVEFQVMATGFTWETQNREADTAACMAVWQHGKRMLADPLLDMVVLDELTYMVAYDYLPLEEVISALNARPGHQTVIITGRGCHRDILDLADTVSELRPVKHAFDAGVKAQMGIDY

2222222222222222222222222222222222221111111222222222222222222222222222222222222222222222222222222222222222222222222222222222222122122222222222222222222222222222222222222222222222222122222222222222

>1ESQA

MRGSHHHHHHGSMDAQSAAKCLTAVRRHSPLVHSITNNVVTNFTANGLLALGASPVMAYAKEEVADMAKIAGALVLNIGTLSKESVEAMIIAGKSANEHGVPVILDPVGAGATPFRTESARDIIREVRLAAIRGNAAEIAHTVGVTDWLIKGVDAGEGGGDIIRLAQQAAQKLNTVIAITGEVDVIADTSHVYTLHNGHKLLTKVTGAGSLLTSVVGAFCAVEENPLFAAIAAISSYGVAAQLAAQQTADKGPGSFQIELLNKLSTVTEQDVQEWATIERVTVS

22222222222222222222222222222222222222222222222222222222222222222222222222222222222222222222222222222222222222222222222222222222222212122222222222222222222222222222222222222222222111212222222222221111211222221221222222222222222222222222122222222222222222222222222222222222222222222222

>2QB8A

MSPLRKTVPEFLAHLKSLPISKIASNDVLTICVGNESADMDSIASAITYSYCQYIYNEGTYSEEKKKGSFIVPIIDIPREDLSLRRDVMYVLEKLKIKEEELFFIEDLKSLKQNVSQGTELNSYLVDNNDTPKNLKNYIDNVVGIIDHHFDLQKHLDAEPRIVKVSGSCSSLVFNYWYEKLQGDREVVMNIAPLLMGAILIDTSNMRRKVEESDKLAIERCQAVLSGAVNEVSAQGLEDSSEFYKEIKSRKNDIKGFSVSDILKKDYKQFNFQGKGHKGLEIGLSSIVKRMSWLFNEHGGEADFVNQCRRFQAERGLDVLVLLTSWRKAGDSHRELVILGDSNVVRELIERVSDKLQLQLFGGNLDGGVAMFKQLNVEATRKQVVPYLEEAYSNLEE

2222222222222222222222222222222222222222222222222222222222222222222222222222222222222222222222222222222222222222222222222222222222222222222222222222122222222222222222222222222222222222222222222222222222222222222222222222222222222222222222222222222222222222222222222221222222222222222221222222222222222222222222222222222222222222222222222222222222222222222222222222222222222222222112222222222222222

>1EE1A

SMQEKIMRELHVKPSIDPKQEIEDRVNFLKQYVKKTGAKGFVLGISGGQDSTLAGRLAQLAVESIREEGGDAQFIAVRLPHGTQQDEDDAQLALKFIKPDKSWKFDIKSTVSAFSDQYQQETGDQLTDFNKGNVKARTRMIAQYAIGGQEGLLVLGTDHAAEAVTGFFTKYGDGGADLLPLTGLTKRQGRTLLKELGAPERLYLKEPTADLLDEKPQQSDETELGISYDEIDDYLEGKEVSAKVSEALEKRYSMTEHKRQVPASMFDDWWK

2222222222222222222222222222222222222222221111211112222222222222222222222222111222212222222222222222222222222222222222222222222222222222221222222222222222221222212222222222122222222222212222222222222222222211222222222222222222222222222222222222222222222222222222222222222

>1TQPA

UNIAELYGKUGKHSWRIUDAIFKNLWDYEYVPLQLISSHARIGEEKARNILKYLSDLRVVQNRQKDYEGSTFTFIGLSLYSLHRLVRSGKVDAIGKLUGEGKESAVFNCYSEKFGECVVKFHKVGHTSFKKVKEKRDYGDLHFSVLAIRSARNEFRALQKLQGLAVPKVYAWEGNAVLUELIDAKELYRVRVENPDEVLDUILEEVAKFYHRGIVHGDLSQYNVLVSEEGIWIIDFPQSVEVGEEGWREILERDVRNIITYFSRTYRTEKDINSAIDRILQE

222222222222222222222222222222222222222222222222222222222222222222222222222222222222222222222222222222112122222222222121222222222222222222222222222222222222222222222212222222222221112221222222222222222222222222222222222221122222222221122222222222222222222222222222222222222222222222

>3FVQA

MTAALHIGHLSKSFQNTPVLNDISLSLDPGEILFIIGASGCGKTTLLRCLAGFEQPDSGEISLSGKTIFSKNTNLPVRERRLGYLVQEGVLFPHLTVYRNIAYGLGNGKGRTAQERQRIEAMLELTGISELAGRYPHELSGGQQQRAALARALAPDPELILLDEPFSALDEQLRRQIREDMIAALRANGKSAVFVSHDREEALQYADRIAVMKQGRILQTASPHELYRQPADLDAALFIGEGIVFPAALNADGTADCRLGRLPVQSGAPAGTRGTLLIRPEQYSLHPHSAPAASIHAVVLKTTPKARHTEISLRAGQTVLTLNLPSAPTLSDGISAVLHLDGPALFFPGNTLEHHHHHH

22222222222221221212222222222222222222111111122222222222222222222222222222222222222222122222222222222222222222222222222222222222222221222121111222222222222222222222222222222222222222222222222222222222222222222222222222222222222222222222222222222222222222222222222222222222222222222222222222222222222222222222222222222222222222222222222222222222222222222222222

>2A5YB

MLCEIECRALSTAHTRLIHDFEPRDALTYLEGKNIFTEDHSELISKMSTRLERIANFLRIYRRQASELGPLIDFFNYNNQSHLADFLEDYIDFAINEPDLLRPVVIAPQFSRQMLDRKLLLGNVPKQMTCYIREYHVDRVIKKLDEMCDLDSFFLFLHGRAGSGKSVIASQALSKSDQLIGINYDSIVWLKDSGTAPKSTFDLFTDILLMLKSEDDLLNFPSVEHVTSVVLKRMICNALIDRPNTLFVFDDVVQEETIRWAQELRLRCLVTTRDVEISNAASQTCEFIEVTSLEIDECYDFLEAYGMPMPVGEKEEDVLNKTIELSSGNPATLMMFFKSCEPKTFEKMAQLNNKLESRGLVGVECITPYSYKSLAMALQRCVEVLSDEDRSALAFAVVMPPGVDIPVKLWSCVIPVDICSNEEEQLDDEVADRLKRLSKRGALLSGKRMPVLTFKIDHIIHMFLKHVVDAQTIANGISILEQRLLEIGNNNVSVPERHIPSHFQKFRRSSASEMYPKTTEETVIRPEDFPKFMQLHQKFYDSLKNFACC

222222222222222222222222222222222222222222222222222222222222222222222222222222222222222222222222222222222222222222222222222222212212222222222222222222222222222111111112222222222222222222222222222222222222222222222222222222222222222222222222222222222222222222222222222222221222222222222222222222222222122212222222222222222222222221122122222222222222222222222222222222211222222222222222222222222222222222222222222222222222222222222222222222222222222222222222222222222222222222222222222222222222222222222222222222222222222222222222222222222222222222222

>1YIDB

MPFVDLEVPTMTTPTPAATPARPRVLTGDRPTGALHLGHLAGSLQNRVRLQDEAELFVLLADVQALTDHFDRPEQVRENVLAVALDYLAAGLDPQKTTCVVQSAVPELAELTVYFLNLVTVSHLRQNPTVKAEIAQKGYGERVPAGFFVYPVSQAADIAAFGATLVPVGDDQLPMLEQTREIVRRFNALYAPVLAEPQAQLSRVPRLPGLDGQAKMSKSLGNAIALGDSADEVARKVMGMYTDPGHLRASDPGRVEGNPVFTFLDAFDPDPARVQALKDQYRAGGLGDVKVKKHLIDVLNGVLAPIRTRRAEYERDPDAVLRFVTEGTARGREVAAQTLGQVRRAMRLFGH

222222222222222222222222222111222221211222222222222222222222222222222222222222222222222222222222222222222222222222222222222222222222222222222222222222222222222222222222222222222222222222222222222222222222211222222221121222222222222222222222222222222222222222222222222222222222222222222222222222222222222222222222222222222222222222222222222222222222222

>1N5IA

MLIAIEGVDGAGKRTLVEKLSGAFRAAGRSVATLAFPRYGQSVAADIAAEALHGEHGDLASSVYAMATLFALDRAGAVHTIQGLCRGYDVVILDRYVASNAAYSAARLHENAAGKAAAWVQRIEFARLGLPKPDWQVLLAVSAELAGERSRGRAQRDPGRARDNYERDAELQQRTGAVYAELAAQGWGGRWLVVGADVDPGRLAATLAPPDVPS

2222222222222222222222222222222211222222122222222222222222222222222222222222222222212222222222222222222222222222222222222222222222222222222222222222222222222222222222222222222222222222222222222222222222222222222222

>1SVMA

GLKEHDFNPEEAEETKQVSWKLVTEYAMETKCDDVLLLLGMYLEFQYSFEMCLKCIKKEQPSHYKYHEKHYANAAIFADSKNQKTICQQAVDTVLAKKRVDSLQLTREQMLTNRFNDLLDRMDIMFGSTGSADIEEWMAGVAWLHCLLPKMDSVVYDFLKCMVYNIPKKRYWLFKGPIDSGKTTLAAALLELCGGKALNVNLPLDRLNFELGVAIDQFLVVFEDVKGTGGESRDLPSGQGINNLDNLRDYLDGSVKVNLEKKHLNKRTQIFPPGIVTMNEYSVPKTLQARFVKQIDFRPKDYLKHCLERSEFLLEKRIIQSGIALLLMLIWYRPVAEFAQSIQSRIVEWKERLDKEFSLSVYQKMKFNVAMGIGVLD

22222222222222222222222222222222222222222222222222222222222222222222222222222222222222222222222222222222222222222222222222222222222222222222222222122222222222222222222122222222211111112222222222222222222222222222222222222221222222222222222222222222222222222222222222222222222222122222222221222222211122112212222221222222222222222222222222222222222222222222222222222222222222222

>2YWWA

MIPMEELKVKKITNGTVIDHIDAGKALMVFKVLNVPKETSVMIAINVPSKKKGKKDILKIEGIELKKEDVDKISLISPDVTINIIRNGKVVEKLKPQIPDEIEGTLKCTNPNCITNKEKVRGKFKIESKNPLKIRCYYCEKFLNEVIFE

22222222111122221211222222222222222222222122221111122212121222222222222222222222221212212122122222222222222222222222222222222222222222222222222222222

>1Y56A

MLMRPLDLTEKRGKKVTIYFEGKELEAYEGEKLPVALLANEIYWLTTSNEGRKRGAFTFGPVPMTVNGVKGLEARRIKVKDGMKIERQGYYDFHEEPVVEPGEIERVVVDVAIIGGGPAGIGAALELQQYLTVALIEERGWLGGDMWLKGIKQEGFNKDSRKVVEELVGKLNENTKIYLETSALGVFDKGEYFLVPVVRGDKLIEILAKRVVLATGAIDSTMLFENNDMPGVFRRDFALEVMNVWEVAPGRKVAVTGSKADEVIQELERWGIDYVHIPNVKRVEGNEKVERVIDMNNHEYKVDALIFADGRRPDINPITQAGGKLRFRRGYYSPVLDEYHRIKDGIYVAGSAVSIKPHYANYLEGKLVGAYILKEFGYDAQPCIYEEKLREYEPESLSIPRIPLDKFNLEDVQICGCDVSLKKVDEVIRKGITDLQIIKRLTHLAMGFCQGRYCLFNGAVVVSQRTGKKLSEIDLPVARSPIKNVKMGILARR

2222222222222222222222222222222222222222222222222222222222222222222222222222222222222222222222222222222222222222221211122222222222222222111222211222222222222222222222222222222222221212222222222222222222222222222221112222222222222222222222222222222222222222222222222222222222222222222222222222222222222222222222122222222222222222222222222222222222222112222221221222222222222222222222222222222222222222222222222222222222222222222222222222222222222222222222222222222222222222222222222112222222222

>1Z0SA

MGSSHHHHHHDYDIPTTENLYFQGGGGGGMRAAVVYKTDGHVKRIEEALKRLEVEVELFNQPSEELENFDFIVSVGGDGTILRILQKLKRCPPIFGINTGRVGLLTHASPENFEVELKKAVEKFEVERFPRVSCSAMPDVLALNEIAVLSRKPAKMIDVALRVDGVEVDRIRCDGFIVATQIGSTGYAFSAGGPVVEPYLECFILIPIAPFRFGWKPYVVSMERKIEVIAEKAIVVADGQKSVDFDGEITIEKSEFPAVFFKNEKRFRNLFGKVRSIG

22222222222222222222222222222222222222222222222222222222222222222222222222222212211222222222222222222222222222222222222222222222222222222222222112222222211122222222222222212122222222222112212222222222222222221212222222222222222222222222212122222222222222222222222222222222222222

>1HI1A

PRRAPAFPLSDIKAQMLFANNIKAQQASKRSFKEGAIETYEGLLSVDPRFLSFKNELSRYLTDHFPANVDEYGRVYGNGVRTNFFGMRHMNGFPMIPATWPLASNLKKRADADLADGPVSERDNLLFRAAVRLMFSDLEPVPLKIRKGSSTCIPYFSNDMGTKIEIAERALEKAEEAGNLMLQGKFDDAYQLHQMGGAYYVVYRAQSTDAITLDPKTGKFVSKDRMVADFEYAVTGGEQGSLFAASKDASRLKEQYGIDVPDGFFCERRRTAMGGPFALNAPIMAVAQPVRNKIYSKYAYTFHHTTRLNKEEKVKEWSLCVATDVSDHDTFWPGWLRDLICDELLNMGYAPWWVKLFETSLKLPVYVGAPAPEQGHTLLGDPSNPDLEVGLSSGQGATDLMGTLLMSITYLVMQLDHTAPHLNSRIKDMPSACRFLDSYWQGHEEIRQISKSDDAMLGWTKGRALVGGHRLFEMLKEGKVNPSPYMKISYEHGGAFLGDILLYDSRREPGSAIFVGNINSMLNNQFSPEYGVQSGVRDRSKRKRPFPGLAWASMKDTYGACPIYSDVLEAIERCWWNAFGESYRAYREDMLKRDTLELSRYVASMARQAGLAELTPIDLEVLADPNKLQYKWTEADVSANIHEVLMHGVSVEKTERFLRSVMPR

2222222222222222222222222222222222222222222222222222222222222222222222222222222222222222222222222222222222222222222222222222222222222222222222222222222222222222222222222222222222222222222222222222222222222122222222222222222222222222222222222222222222222222222222222221212222222222222222222222222222222222222222222222222222222212222222222222222222222222222222222222222222222222222222222222222222222222222222222222222222222222222222222222222222222222222222222222222222222222222222222222222222222222222222222222222222222222222222222222222222222222222222222222222222222222222222222222222222222222222222222222222222222222222222222222222222222222222222222222222222222222

>2O0HA

MGSSHHHHHHSSGLVPRGSHMLEDPMEQPINVLNDFHPLNEAGKILIKHPSLAERKDEDGIHWIKSQWDGKWYPEKFSDYLRLHKIVKIPNNSDKPELFQTYKDKNNKRSRYMGLPNLKRANIKTQWTREMVEEWKKCRDDIVYFAETYCAITHIDYGVIKVQLRDYQRDMLKIMSSKRMTVCNLSRQLGKTTVVAIFLAHFVCFNKDKAVGILAHKGSMSAEVLDRTKQAIELLPDFLQPGIVEWNKGSIELDNGSSIGAYASSPDAVRGNSFAMIYIEDCAFIPNFHDSWLAIQPVISSGRRSKIIITTTPNGLNHFYDIWTAAVEGKSGFEPYTAIWNSVKERLYNDEDIFDDGWQWSIQTINGSSLAQFRQEHTAAFEGTS

2222222222222222222222222222222222222222222222222222222222222222222222222222222222222222222222222222222222222222222222222222222222222222222222222222222112222222211212212222222222222222222121111222222222222222222222222222222222122222222222222222222222222222222222222222222222222222222222222222222222222222222222222222222222222222222222222222222222222222222222222222222222222222222222222

>1B0UA

MMSENKLHVIDLHKRYGGHEVLKGVSLQARAGDVISIIGSSGSGKSTFLRCINFLEKPSEGAIIVNGQNINLVRDKDGQLKVADKNQLRLLRTRLTMVFQHFNLWSHMTVLENVMEAPIQVLGLSKHDARERALKYLAKVGIDERAQGKYPVHLSGGQQQRVSIARALAMEPDVLLFDEPTSALDPELVGEVLRIMQQLAEEGKTMVVVTHEMGFARHVSSHVIFLHQGKIEEEGDPEQVFGNPQSPRLQQFLKGSLKKLEH

2222222222222221221212222222222222222222111111122222222222222222222222222222222222222222222222222222222222222222222222222222222222222222222222222222222222222222222222222222222222222222222222222222222222222222222222222222222222222222222222222222222222222222222222

>2CG9A

MASETFEFQAEITQLMSLIINTVYSNKEIFLRELISNASDALDKIRYKSLSDPKQLETEPDLFIRITPKPEQKVLEIRDSGIGMTKAELINNLGTIAKSGTKAFMEALSAGADVSMIGQFGVGFYSLFLVADRVQVISKSNDDEQYIWESNAGGSFTVTLDEVNERIGRGTILRLFLKDDQLEYLEEKRIKEVIKRHSEFVAYPIQLVVTKEVEKEVPIPEEEKKDEEKKDEEKKDEDDKKPKLEEVDEEEEKKPKTKKVKEEVQEIEELNKTKPLWTRNPSDITQEEYNAFYKSISNDWEDPLYVKHFSVEGQLEFRAILFIPKRAPFDLFESKKKKNNIKLYVRRVFITDEAEDLIPEWLSFVKGVVDSEDLPLNLSREMLQQNKIMKVIRKNIVKKLIEAFNEIAEDSEQFEKFYSAFSKNIKLGVHEDTQNRAALAKLLRYNSTKSVDELTSLTDYVTRMPEHQKNIYYITGESLKAVEKSPFLDALKAKNFEVLFLTDPIDEYAFTQLKEFEGKTLVDITKDFELEETDEEKAEREKEIKEYEPLTKALKEILGDQVEKVVVSYKLLDAPAAIRTGQFGWSANMERIMKAQALRDSSMSSYMSSKKTFEISPKSPIIKELKKRVDEGGAQDKTVKDLTKLLYETALLTSGFSLDEPTSFASRINRLISLGLN

22222222222222222222222222222222122211221222222222222222222222222222222222222212222122222221222222112222222222222222211111112222222222222222222222222222222222222222222222122222222222222222222222222222222222222222222222222222222222222222222222222222222222222222222222222222222222222222222222222222222222222222222222222222222222222222222222222222222222222222222222222222222222222221222222222222222222222222222222222222222222222222222222222222222222222222222222222222222222222222222222222222222222222222222222222222222222222222222222222222222222222222222222222222222222222222222222222222222222222222222222222222222222222222222222222222222222222222222222222222222222222222222222222

>1OBDA

USITKTELDGILPLVARGKVRDIYEVDAGTLLFVATDRISAYDVIMENSIPEKGILLTKLSEFWFKFLSNDVRNHLVDIAPGKTIFDYLPAKLSEPKYKTQLEDRSLLVHKHKLIPLEVIVRGYITGSAWKEYVKTGTVHGLKQPQGLKESQEFPEPIFTPSTKAEQGEHDENISPAQAAELVGEDLSRRVAELAVKLYSKCKDYAKEKGIIIADTKFEFGIDEKTNEIILVDEVLTPDSSRFWNGASYKVGESQDSYDKQFLRDWLTANKLNGVNGVKMPQDIVDRTRAKYIEAYETLTGSKWSH

222222222222222221121212222222121222222222222222222222222222222222222222221222222222222222222222222222222222211121222222222222222222222222222222222222222222222222222222222222222222222222222222222222222222222222222222221222222222222112222222222222222222222222222222222222222222222222222222222222222222222222

>2HS0A

MKLRYLNILKEKLGREPTFVELQAFSVMWSEHCGYSHTKKYIRRLPKTGFEGNAGVVNLDDYYSVAFKIESHNHPSAIEPYNGAATGVGGIIRDVLAMGARPTAIFDSLHMSRIIDGIIEGIADYGNSIGVPTVGGELRISSLYAHNPLVNVLAAGVVRNDMLVDSKASRPGQVIVIFGGATGRDGIHGASFASEDLTGDKATKLSIQVGDPFAEKMLIEAFLEMVEEGLVEGAQDLGAGGVLSATSELVAKGNLGAIVHLDRVPLREPDMEPWEILISESQERMAVVTSPQKASRILEIARKHLLFGDVVAEVIEEPVYRVMYRNDLVMEVPVQLLANAPEEDIVEYTPGKIPEFKRVEFEEVNAREVFEQYDHMVGTDTVVPPGFGAAVMRIKRDGGYSLVTHSRADLALQDTYWGTLIAVLESVRKTLSVGAEPLAITNCVNYGDPDVDPVGLSAMMTALKNACEFSGVPVASGNASLYNTYQGKPIPPTLVVGMLGKVNPQKVAKPKPSKVFAVGWNDFELEREKELWRAIRKLSEEGAFILSSSQLLTRTHVETFREYGLKIEVKLPEVRPAHQMVLVFSERTPVVDVPVKEIGTLSR

222222222222222222222222222222222212222221221222222222222222222222212122222222222222222222222122222222222212222222222222222222222222221111122222222222222222222222222222222222222222222222222222222222222222222222222222222222222222222222212222222222222222222222222222222222222222222222222222222222222222222222222222222222222222222222222222222222222222222222222222222221222122222222222222211122222222222222221222222222222222222222221222222222222121222222222222222222222222222221211112222222222222222222222222222222222222222222222222222222222222222222211122121122222222222222222222222222222222222222222222222

>1JJVA

MTYIVGLTGGIGSGKTTIANLFTDLGVPLVDADVVAREVVAKDSPLLSKIVEHFGAQILTEQGELNRAALRERVFNHDEDKLWLNNLLHPAIRERMKQKLAEQTAPYTLFVVPLLIENKLTALCDRILVVDVSPQTQLARSAQRDNNNFEQIQRIMNSQVSQQERLKWADDVINNDAELAQNLPHLQQKVLELHQFYLQQAENKNA

22222222221111111222222222222222222222222222222222222222222222222222222222222222222222222222222222222222222222222222222222222222222222222221222222222222222222222222222222222212111222222222222222222222222222

>2FSGA

VFGSRNDRTLRRURKVVNIINAUEPEUEKLSDEELKGKTAEFRARLEKGEVLENLIPEAFAVVREASKRVFGURHFDVQLLGGUVLNERCIAEURTGEGKTLTATLPAYLNALTGKGVHVVTVNDYLAQRDAENNRPLFEFLGLTVGINLPGUPAPAKREAYAADITYGTNNEYGFDYLRDNUAFSPEERVQRKLHYALVDEVDSILIDEARTPLIISGPAEDSSEUYKRVNKIIPHLIRQEKEDSETFQGEGHFSVDEKSRQVNLTERGLVLIEELLVKEGIUDEGESLYSPANIULUHHVTAALRAHALFTRDVDYIVKDGEVIIVDEHTGRTUQGRRWSDGLHQAVEAKEGVQIQNENQTLASITFQNYFRLYEKLAGUTGTADTEAFEFSSIYKLDTVVVPTNRPUIRKDLPDLVYUTEAEKIQAIIEDIKERTAKGQPVLVGTISIEKSELVSNELTKAGIKHNVLNAKFHANEAAIVAQAGYPAAVTIATNUAGRGTDIVLGGSWQAEVAALENPTAEQIEKIKADWQVRHDAVLEAGGLHIIGTERHESRRIDNQLRGRSGRQGDAGSSRFYLSUEDALURIFASDRVSGUURKLGUKPGEAIEHPWVTKAIANAQRKVESRNFDIRKQLLEYDDVANDQRRAIYSQRNELLDVSDVSETINSIREDVFKATIDAYIPPQSLEEUWDIPGLQERLKNDFDLDLPIAEWLDKEPELHEETLRERILAQSIEVYQRKEEVVGAEUURHFEKGVULQTLDSLWKEHLAAUDYLRQGIHLRGYAQKDPKQEYKRESFSUFAAULESLKYEVISTLSKVQVRUPEEVEELEQQRRUEAERLAQUQQLSHQD

2222222222222222222222222222222222222222222222222222222222222222222222222121221222222222222222221111112222222222222222222222222222222222222222222222222222222222222222222222222222222222222222222222222222222222222222222222222222222222222222222222222222222222222222222222222222222222222222222222222222222222222222222222222222222222222222222222222222222222222222222222222222222222222222222222222222222222222222222222222222222222222222222222222222222222222222222222222222222222222222222222222222222222222222222222222222222222222222222222222222222222222222222222222222222222222222222222222222222222222222222222222222222222222222222222222222222222222222222222222222222222222222222222222222222222222222222222222222222222222222222222222222222222222222222222222222222222222222222222222222222222222222222222222222222222222222222222222222222222222222222222222222222

>2V92E

MESVAAESAPAPENEHSQETPESNSSVYTTFMKSHRCYDLIPTSSKLVVFDTSLQVKKAFFALVTNGVRAAPLWDSKKQSFVGMLTITDFINILHRYYKSALVQIYELEEHKIETWREVYLQDSFKPLVCISPNASLFDAVSSLIRNKIHRLPVIDPESGNTLYILTHKRILKFLKLFITEFPKPEFMSKSLEELQIGTYANIAMVRTTTPVYVALGIFVQHRVSALPVVDEKGRVVDIYSKFDVINLAAEKTYNNLDVSVTKALQHRSHYFEGVLKCYLHETLEAIINRLVEAEVHRLVVVDEHDVVKGIVSLSDILQALVLTGGEKKP

222222222222222222222222222222222222222222222222222222222222222222221222222222222221211112222222222222222222222222222222222222111222222222222222222211121222222222222222122222222222222222222222222222222222222222222222222222221222222222222212111122222222222222222222222122212111222222222222222222211122222222222222222222222222222222

>1KO5A

MSTTNHDHHIYVLMGVSGSGKSAVASEVAHQLHAAFLDGDFLHPRRNIEKMASGEPLNDDDRKPWLQALNDAAFAMQRTNKVSLIVCSALKKHYRDLLREGNPNLSFIYLKGDFDVIESRLKARKGHFFKTQMLVTQFETLQEPGADETDVLVVDIDQPLEGVVASTIEVIKKGK

2222222222222221111111122222222222222222222222222222222222222222222222222222222222222222222222222222222222222222222222212221222222222222222222222222222222222111222222222222222

>1XSCA

GPLGSMALRACGLIIFRRCLIPKVDNNAIEFLLLQASDGIHHWTPPKGHVEPGEDDLETALRATQEEAGIEAGQLTIIEGFKRELNYVARNKPKTVIYWLAEVKDYDVEIRLSHEHQAYRWLGLEEACQLAQFKEMKAALQEGHQFLCSIEAL

222222222222222222222222222222222222222221222222222222222222222222222222222222222222221212222222222222222222222222122222222222222222121222222222222222222

>3E7EA

GSPQMSSLGTVDAPNFIVGNPWDDKLIFKLLSGLSKPVSSYPNTFEWQCKLPAIKPKTEFQLGSKLVYVHHLLGEGAFAQVYEATQGDLNDAKNKQKFVLKVQKPANPWEFYIGTQLMERLKPSMQHMFMKFYSAHLFQNGSVLVGELYSYGTLLNAINLYKNTPEKVMPQGLVISFAMRMLYMIEQVHDCEIIHGDIKPDNFILGNGFLEQDDEDDLSAGLALIDLGQSIDMKLFPKGTIFTAKCETSGFQCVEMLSNKPWNYQIDYFGVAATVYCMLFGTYMKVKNEGGECKPEGLFRRLPHLDMWNEFFHVMLNIPDCHHLPSLDLLRQKLKKVFQQHYTNKIRALRNRLIVLLLECKRSRK

22222222222222222222222222222222222222222222222222222222222222222222222221111112122222222222222222221222222222222222222222222222212222222222222222121222222222222222222222222222222222222222222222221222112122222222222222222222112122222222222222222222222222222222222222222222222222222222222222222222222222222222222222222222222222222222222222222222222222222222222222222

>3EFSA

MFKNLIWLKEVDSTQERLKEWNVSYGTALVADRQTKGRGRLGRKWLSQEGGLYFSFLLNPKEFENLLQLPLVLGLSVSEALEEITEIPFSLKWPNDVYFQEKKVSGVLRELSKDKLIVGIGINVNQREIPEEIKDRATTLYEITGKDWDRKEVLLKVLKRISENLKKFKEKSFKEFKGKIESKMLYLGEEVKLLGEGKITGKLVGLSEKGGALILTEEGIKEILSGEFSLRRS

22222222222222222222222222222222222222212211112222222222222222222222222222222222222222222221222121222212222222222222222222122222222212221222222222222222222222222222222222222222222222222222222122222222222222222222222222222222222211121

>3H39A

MQIFRDVSKLLVERVDPKILNLFRLLGKFGDEVNMPVYVVGGFVRDLLLGIKNLDIDIVVEGNALEFAEYAKRFLPGKLVKHDKFMTASLFLKGGLRIDIATARLEYYESPAKLPDVEMSTIKKDLYRRDFTINAMAIKLNPKDFGLLIDFFGGYRDLKEGVIRVLHTLSFVDDPTRILRAIRFEQRFDFRIEETTERLLKQAVEEGYLERTTGPRLRQELEKILEEKNPLKSIRRMAQFDVIKHLFPKTYYTPSMDEKMENLFRNIPWVEENFGEVDRFYAVLHVFLEFYDDESWKEVRDRYSLRRNLINEIRHVEKSAPALLEMLSERVPASFVYPLVKGVSNETICHFLAYLSGEKEGLFKSYLLKIKNTKLEKINGEYLIRKGITSGKIIGEVLEKILMKKLDGDTRDEEEILEEVLASLETEGKLAAALEHHHHHH

222222222222222222222222222222222222222211221222222222221222222222222222222222222222222222222222222222222222222222222222222222221122212222222222222222222222222222222222222221211221221122122222222222222222222222222222222222122222222222222222222222222222222222222222222222222222222222222222222222222222222222222222222222222222222222222222222222222222222222222222222222222222222222222222222222222222222222222222222222222222222222222222222222222

>2Q7GA

MGSSHHHHHHSSGLVPRGSHMASAPALTKSQTDRLEVLLNPKDEISLNSGKPFRELESELLSRRKKDLQQIYAEERENYLGKLEREITRFFVDRGFLEIKSPILIPLEYIERMGIDNDTELSKQIFRVDKNFCLRPMLAPNLYNYLRKLDRALPDPIKIFEIGPCYRKESDGKEHLEEFTMLNFCQMGSGCTRENLESIITDFLNHLGIDFKIVGDSCMVYGDTLDVMHGDLELSSAVVGPIPLDREWGIDKPWIGAGFGLERLLKVKHDFKNIKRAARSESYYNGISTNL

222222222222222222222222222222222222222222222222222222222222222222222222222222222222222222222222222222222222222222222222222222222222222222222222222222222222222222222212122222112212122222222222222222222222222222222222222222222222222211112222222222222222222221212212222222222222222222222222222

>2Z02A

MEIKLEEILKKQPLYSGKAKSIYEIDDDKVLIEFRDDITAGNGAKHDVKQGKGYLNALISSKLFEALEENGVKTHYIKYIEPRYMIAKKVEIIPIEVIVRNIAAGSLCRRYPFEEGKELPFPIVQFDYKNDEYGDPMLNEDIAVALGLATREELNKIKEIALKVNEVLKKLFDEKGIILVDFKIEIGKDREGNLLVADEISPDTMRLWDKETRDVLDKDVFRKDLGDVIAKYRIVAERLGLL

22222222222222121111212222222222222222222222222222222222222222222222222222122222222222212121222222222222222222222222222222222222122222222222222222222222222222222222222222222222222222221222222222222122222222222222222222222222222222222222222222

>3F5MA

MAVESRSRVTSKLVKAHRAMLNSVTQEDLKVDRLPGADYPNPSKKYSSRTEFRDKTDYIMYNPRPRDEPSSENPVSVSPLLCELAAARSRIHFNPTETTIGIVTCGGICPGLNDVIRSITLTGINVYNVKRVIGFRFGYWGLSKKGSQTAIELHRGRVTNIHHYGGTILGSSRGPQDPKEMVDTLERLGVNILFTVGGDGTQRGALVISQEAKRRGVDISVFGVPKTIDNDLSFSHRTFGFQTAVEKAVQAIRAAYAEAVSANYGVGVVKLMGRDSGFIAAQAAVASAQANICLVPENPISEQEVMSLLERRFCHSRSCVIIVAEGFGQDWGRGSGGYDASGNKKLIDIGVILTEKVKAFLKANKSRYPDSTVKYIDPSYMIRACPPSANDALFCATLATLAVHEAMAGATGCIIAMRHNNYILVPIKVATSVRRVLDLRGQLWRQVREITVDLGSDVRLARKLEIRRELEAINRNRDRLHEELAKL

2222222222222222222222222222222222222222222222222222222222222222222222222222222222222222222222222222222211122222222222222222222222222222221222222222222222222222222222222222111222222222222222222222111112112222222222222222222221222222222222222222222222222222222222222222222222222222222222222222222222222222222222222222222222222222222222222222121222222222222222222222222222222222222222222222222222222222222222222222222222222222222222222222222222222222222222222222222222222222222222222222222

>1II0A

MQFLQNIPPYLFFTGKGGVGKTSISCATAIRLAEQGKRVLLVSTDPASNVGQVFSQTIGNTIQAIASVPGLSALEIDPQAAAQQYRARIVDPIKGVLPDDVVSSINEQLSGACTTEIAAFDEFTGLLTDASLLTRFDHIIFDTAPTGHTIRLLQLPGAWSSFIDSNPEGASCLGPMAGLEKQREQYAYAVEALSDPKRTRLVLVARLQKSTLQEVARTHLELAAIGLKNQYLVINGVLPKTEAANDTLAAAIWEREQEALANLPADLAGLPTDTLFLQPVNMVGVSALSRLLSTQPVASPSSDEYLQQRPDIPSLSALVDDIARNEHGLIMLMGKGGVGKTTMAAAIAVRLADMGFDVHLTTSDPAAHLSMTLNGSLNNLQVSRIDPHEETERYRQHVLETKGKELDEAGKRLLEEDLRSPCTEEIAVFQAFSRVIREAGKRFVVMDTAPTGHTLLLLDATGAYHREIAKKMGEKGHFTTPMMLLQDPERTKVLLVTLPETTPVLEAANLQADLERAGIHPWGWIINNSLSIADTRSPLLRMRAQQELPQIESVKRQHASRVALVPVLASEPTGIDKLKQLAGHHHHHH

2222222222222222122222222222222222222222222222222222222222222222222222222222222222222222222222222222222222222222222222222222222222222222222222222222222222222222222222222222222222222222222222222222222222222221212222222222222222222222222222222222222222222222222222222222222222222222222222222222222222222222222222222222222222222222222222211111112222222222222222222222222222222222222222222222222222222222222222222222222222222222222222222222222222222222222222222222222222222222222222222222222222222222222222222222222222222222222222112222222222222222222222222222222222222121222122222222122222222

>1KP2A

TTILKHLPVGQRIGIAFSGGLDTSAALLWMRQKGAVPYAYTANLGQPDEEDYDAIPRRAMEYGAENARLIDCRKQLVAEGIAAIQCGAFHNTTGGLTYFNTTPLGRAVTGTMLVAAMKEDGVNIWGDGSTYKGNDIERFYRYGLLTNAELQIYKPWLDTDFIDELGGRHEMSEFMIACGFDYKMSVEKAYSTDSNMLGATHEAKDLEYLNSSVKIVNPIMGVKFWDESVKIPAEEVTVRFEQGHPVALNGKTFSDDVEMMLEANRIGGRHGLGMSDQIENRIIEAKSRGIYEAPGMALLHIAYERLLTGIHNEDTIEQYHAHGRQLGRLLYQGRWFDSQALMLRDSLQRWVASQITGEVTLELRRGNDYSILNTVSENLTYKPERLTMEKGDSVFSPDDRIGQLTMRNLDITDTREKLFGYAKTGLLSSSAASGVPQVENLENKGQSVEHHHHHH

22222222222222211222221222222222222222211122222222222222222222222222222222222222222222222222222222222222222222221222222222222211112222122212222222222222222222222222222222222222222222222222222212222222222222222222222222222222222222222222222222222222222222222222222222222222222222222222222222222222222222222222222222222222222222222222222222222222222222222222222222222222222222222222222222222222222222222222222222222222222222222222222222222222222222222222222

>1VJCA

SLSNKLTLDKLDVKGKRVVMRVDFNVPMKNNQITNNQRIKAAIPSIKFCLDNGAKSVVLMSHLGRPDGIPMPDKYSLEPVAVELKSLLGKDVLFLKDCVGPEVEKACADPAAGSVILLENLRFHVEEEGKGKDASGSKVKADPAKIEAFRASLSKLGDVYVNDAFGTAHRAHSSMVGVNLPKKAGGFLMKKELNYFAKALESPERPFLAILGGAKVADKIQLINNMLDKVNEMIIGGGMAFTFLKVLNNMEIGTSLFDEEGSKIVKDLMSKAEKNGVKITLPVDFVTADKFDENAKTGQATVASGIPAGWMGLDCGPESSKKYSEAVARAKQIVWNGPVGVFEWEAFAQGTKALMDEVVKATSRGCITIIGGGDTATCCAKWNTEDKVSHVSTGGGASLELLEGKVLPGVDALSNV

22222222222222222222222222222222222222222222222222222222222222222222222222222222222222222222222222222222222222222222222222222222222222222222222222222222222222222222222222222222222222222222222222222222222222222222112222122222222222222222112212222222222222212222222222222222222222222222222222222222222222222222222112222222222222222222222222211212222222222222222222222222222221222222222222222222222222222222222222222222

>4AT1B

MTHDNKLGVEAIKRGTVIDHIPAQIGFKLLSLFKLTETDQRITIGLNLPSGEMGRKDLIKIENTFLSEDQVDQLALYAPQATVNRIDNYEVVGKSRPSLPERIDNVLVCPNSNCISHAEPVSSSFAVRKRANDIALKCKYCEKEFSHNVVLAN

222222221111222212122222222222222222222222222222222222222121222222222222222222222221222212122122222222222222222222222222222222222222222222222222222222222

>1F9AA

LRGFIIGRFQPFHKGHLEVIKKIAEEVDEIIIGIGSAQKSHTLENPFTAGERILMITQSLKDYDLTYYPIPIKDIEFNSIWVSYVESLTPPFDIVYSGNPLVRVLFEERGYEVKRPEMFNRKEYSGTEIRRRMLNGEKWEHLVPKAVVDVIKEIKGVERLRKLAQTDK

222212111222121122122222222222222222222222222222222222222222222222222222222222222222222222222222222222222222222222211112122111122122222222222222222222222222222222222222

>1R8BA

MKVEEILEKALELVIPDEEEVRKGREAEEELRRRLDELGVEYVFVGSYARNTWLKGSLEIDVFLLFPEEFSKEELRERGLEIGKAVLDSYEIRYAEHPYVHGVVKGVEVDVVPCYKLKEPKNIKSAVDRTPFHHKWLEGRIKGKENEVRLLKGFLKANGIYGAEYKVRGFSGYLCELLIVFYGSFLETVKNARRWTRRTVIDVAKGEVRKGEEFFVVDPVDEKRNVAANLSLDNLARFVHLCREFMEAPSLGFFKPKHPLEIEPERLRKIVEERGTAVFAVKFRKPDIVDDNLYPQLERASRKIFEFLERENFMPLRSAFKASEEFCYLLFECQIKEISRVFRRMGPQFEDERNVKKFLSRNRAFRPFIENGRWWAFEMRKFTTPEEGVRSYASTHWHTLGKNVGESIREYFEIISGEKLFKEPVTAELCEMMGVKD

22222222222222222222222222222222222222222222211222222222222212122222222222222222222222222222221122222222222222222222222222222222212212222222222222222221222222221222222222212222222222222222222222222222222222222222222222222222222222222222222222222222222222222222222222222222222222222222222222222222222222122222212222222222222222222222222222222222222222222222222222222222222222222222222222222221222121122222222222222222222222222222222222222

>3CRCA

GHMNQIDRLLTIMQRLRDPENGCPWDKEQTFATIAPYTLEETYEVLDAIAREDFDDLRGELGDLLFQVVFYAQMAQEEGRFDFNDICAAISDKLERRHPHVFADSSAENSSEVLARWEQIKTEERAQKAQHSALDDIPRSLPALMRAQKIQKRCANVGFDWTTLGPVVDKVYEEIDEVMYEARQAVVDQAKLEEEMGDLLFATVNLARHLGTKAEIALQKANEKFERRFREVERIVAARGLEMTGVDLETMEEVWQQVARQEIDL

2222222222222222222222222222222222222222222222222222222222222222222222222222222222222222222222222222222222222222222222222222222222222222222222222222222222222222222222222222222222222222222222222222222222222222222222222222222112212222222222222222222222222212222222222

>2JJXA

MAHHHHHHMRPYKRVLIKLSGGALADQTGNSFNSKRLEHIANEILSIVDLGIEVSIVIGGGNIFRGHLAEEWGIDRVEADNIGTLGTIINSLMLRGVLTSKTNKEVRVMTSIPFNAVAEPYIRLRAVHHLDNGYIVIFGGGNGQPFVTTDYPSVQRAIEMNSDAILVAKQGVDGVFTSDPKHNKSAKMYRKLNYNDVVRQNIQVMDQAALLLARDYNLPAHVFNFDEPGVMRRICLGEHVGTLINDDASLLVHEK

222222222222222222222222222222222222222222222222222222222222222222222222222222222222222222222222222222222212222222112111212212212222212222222222222222222222222222222222222222222222222222222222222222222222222222222222222222222222222222222222222222222222222

>3G59A

GAMVMRLGDAAELCYNLTSSYLQIAAESDSIIAQTQRAINTTKSILINETFPKWSPLNGEISFSYNGGKDCQVLLLLYLSCLWEYYIVKLSQSQFDGKFHRFPLTKLPTVFIDHDDTFKTLENFIEETSLRYSLSLYESDRDKCETMAEAFETFLQVFPETKAIVIGIRHTDPFGEHLKPIQKTDANWPDFYRLQPLLHWNLANIWSFLLYSNEPICELYRYGFTSLGNVEETLPNPHLRKDKNSTPLKLNFEWEIENRYKHNEVTKAEPIPIADEDLVKIENLHEDYYPGWYLVDDKLERAGRIKKK

22222222222222222222222222222222222222222222222222222222222222211121111222222222222222222222222222222222222221112122222222222222222222222222222222222222222222222222211112212222222222222222222222222222222222222222222222212222211222222222222222222222222222222222222222222222222222222222222222222222222122222222

>1G3IA

MSEMTPREIVSELDQHIIGQADAKRAVAIALRNRWRRMQLQEPLRHEVTPKNILMIGPTGVGKTEIARRLAKLANAPFIKVEATKFTEVGYVGKEVDSIIRDLTDSAMKLVRQQEIAKNRARAEDVAEERILDALLPPAKNQWGEVENHDSHSSTRQAFRKKLREGQLDDKEIEIDVSAGVSMGVEIMAPPGMEEMTNQLQSLFQNLGSDKTKKRKMKIKDALKALIDDEAAKLINPEELKQKAIDAVEQNGIVFIDEIDKICKKGEYSGADVSREGVQRDLLPLVEGSTVSTKHGMVKTDHILFIASGAFQVARPSDLIPELQGRLPIRVELTALSAADFERILTEPHASLTEQYKALMATEGVNIAFTTDAVKKIAEAAFRVNEKTENIGARRLHTVMERLMDKISFSASDMNGQTVNIDAAYVADALGEVVENEDLSRFIL

222222222222222111222222222222222222222222222222222222222211111112222222222222222222222222222222222222222222222222222222222222222222222222222222222222222222222222222222222222222222222222222222222222222222222222222222222222222222222222222222222222222222222212222222222222222222222222222222222222222222222222222222222222222122222222222221222222212222222222222222222222222222222222222222222222221122222222222222222222222222222222222222222222222222

>3HRCA

GAMDGTAAEPRPGAGSLQHAQPPPQPRKKRPEDFKFGKILGEGSFSTVVLARELATSREYAIKILEKRHIIKENKVPYVTRERDVMSRLDHPFFVKLYFTFQDDEKLYFGLSYAKNGELLKYIRKIGSFDETCTRFYTAEIVSALEYLHGKGIIHRDLKPENILLNEDMHIQITDFGTAKVLSPESKQARANUFVGTAQYVSPELLTEKSACKSSDLWALGCIIYQLVAGLPPFRAGNEGLIFAKIIKLEYDFPEKFFPKARDLVEKLLVLDATKRLGCEEMEGYGPLKAHPFFESVTWENLHQQTPPKLT

22222222222222222222222222222222222222221111212122222222222212122222222222222222222222222222221222222222222222212122212222222222222222222222222222222222222222222221222222222222222222222222222222222222222222222222222222222222222222222222222222222222222222222222222222222222222222222222222222222222222222222222222

>3KMWA

MNKHSGIDFKQLNFLTKLNENHSGELWKGRWQGNDIVVKVLKVRDWSTRKSRDFNEECPRLRIFSHPNVLPVLGACQSPPAPHPTLITHWMPYGSLYNVLHEGTNFVVDQSQAVKFALDMARGMAFLHTLEPLIPRHALNSRSVMIDEDMTARISMADVKFSFQSPGRMYAPAWVAPEALQKKPEDTNRRSADMWSFAVLLWELVTREVPFADLSNMEIGMKVALEGLRPTIPPGISPHVSKLMKICMNEDPAKRPKFDMIVPILEKMQDK

2222222222222222221211122122222222221212222222222222222222222222222222222222222222222221111222222122222222222222222222222222222222222222222221221222222222222121222222222222222222222222222222222222222222222222222222222222222222222222222222222222222222222222222222222222222

>3FDXA

SNAILVPIDISDKEFTERIISHVESEARIDDAEVHFLTVIPSLPYYASLGUAYTAELPGUDELREGSETQLKEIAKKFSIPEDRUHFHVAEGSPKDKILALAKSLPADLVIIASHRPDITTYLLGSNAAAVVRHAECSVLVVR

22222211122222222222222222222222222211122222222222222222222222222222222222222222222222222222211221222222222222211211222222221111222222222222222

>3INNA

MAHHHHHHMGTLEAQTQGPGSMQIIHTIEELRQALAPARQQGKKIGFVPTMGYLHKGHLELVRRARVENDVTLVSIFVNPLQFGANEDLGRYPRDLERDAGLLHDAQVDYLFAPTVSDMYPRPMQTVVDVPPLGNQIEGEARPGHFAGVATVVSKLFNIVGPDAAYFGEKDFQQLVIIRRMVDDMAIPVRIVGVETVREDDGLACSSRNVYLTPEQRRAAIIVPQALDEADRLYRSGMDDPDALEAAIRTFIGRQPLAVPEVIAIRDPETLERLPALQGRPILVALFVRVGATRLLDNRVIGHAAPQITQERAA

22222222222222222222222222222222222222222222222211122212212212222222222222222222212222222222222222222222222222222222222222222222222222222222222222222221122222222222221121122122222222222222222222211222222112222222222222222222222222222222222222222222222222222222222222222222222222222222222222222222222222222222222222

>1MV5A

MLSARHVDFAYDDSEQILRDISFEAQPNSIIAFAGPSGGGKSTIFSLLERFYQPTAGEITIDGQPIDNISLENWRSQIGFVSQDSAIMAGTIRENLTYGLEGDYTDEDLWQVLDLAFARSFVENMPDQLNTEVGERGVKISGGQRQRLAIARAFLRNPKILMLDEATASLDSESESMVQKALDSLMKGRTTLVIAHRLSTIVDADKIYFIEKGQITGSGKHNELVATHPLYAKYVSEQLTVGQ

222222222211221212222222222222222221111111122222222222222222222222222222222222222222222222222222222222222222222222222222222222222222222222222222222222222222222222222222222222222222222222222222222222222222222222222222222222222222222222222222222

>2FAQA

MGARKASAGASRAATAGVRISHPQRLIDPSIQASKLELAEFHARYADLLLRDLRERPVSLVRGPDGIGGELFFQKHAARLKIPGIVQLDPALDPGHPPLLQIRSAEALVGAVQMGSIEFHTWNASLANLERPDRFVLDLDPDPALPWKRMLEATQLSLTLLDELGLRAFLKTSGGKGMHLLVPLERRHGWDEVKDFAQAISQHLARLMPERFSAVSGPRNRVGKIFVDYLRNSRGASTVAAYSVRAREGLPVSVPVFREELDSLQGANQWNLRSLPQRLDELAGDDPWADYAGTRQRISAAMRRQLGRG

222222222222222222222222222222222222222222222222222222222222222222222222122222222222222222222222222222222222222222222221222222222222222221212222222222222222222222222222222212111212222222222222222222222222222222222222222222222222221222221122222212122222222222222222222222222222222222222222222222222222222222222

>1OL6A

ESKKRQWALEDFEIGRPLGKGKFGNVYLAREKQSKFILALKVLFKAQLEKAGVEHQLRREVEIQSHLRHPNILRLYGYFHDATRVYLILEYAPLGTVYRELQKLSKFDEQRTATYITELANALSYCHSKRVIHRDIKPENLLLGSAGELKIANFGWSVHAPSSRRTTLCGTLDYLPPEMIEGRMHDEKVDLWSLGVLCYEFLVGKPPFEANTYQETYKRISRVEFTFPDFVTEGARDLISRLLKHNPSQRPMLREVLEHPWITANSSKPSNCQNKESASKQS

222222222222222221112121212222222222222212222222222222222222222222222222122222222222222221212221222222222222222222222222222222222222222222222122222222221222222222222222222222222222222222222222222222222222222222222222222222222222222222222222222222222222222222222222222222222222222222

>1TF7A

MTSAEMTSPNNNSEHQAIAKMRTMIEGFDDISHGGLPIGRSTLVSGTSGTGKTLFSIQFLYNGIIEFDEPGVFVTFEETPQDIIKNARSFGWDLAKLVDEGKLFILDASPDPEGQEVVGGFDLSALIERINYAIQKYRARRVSIDSVTSVFQQYDASSVVRRELFRLVARLKQIGATTVMTTERIEEYGPIARYGVEEFVSDNVVILRNVLEGERRRRTLEILKLRGTSHMKGEYPFTITDHGINIFPLGAMRLTQRSSNVRVSSGVVRLDEMCGGGFFKDSIILATGATGTGKTLLVSRFVENACANKERAILFAYEESRAQLLRNAYSWGMDFEEMERQNLLKIVCAYPESAGLEDHLQIIKSEINDFKPARIAIDSLSALARGVSNNAFRQFVIGVTGYAKQEEITGLFTNTSDQFMGAHSITDSHISTITDTIILLQYVEIRGEMSRAINVFKMRGSWHDKAIREFMISDKGPDIKDSFRNFERIISGSPTRITVDEKSELSRIVRGVQEKGPESHHHHHH

222222222222222222222222222222222222222222222221111111222222222222222222222222222222222211222222222222222222222222222222222222222222222222222222222222222222222222222222222222222222222222222222222222222222222222222222212222212121112122222211122222222222222222222222222222222222222222222222211111112222222222222222222222222222222222122222222222222222222222222222222222222222222222222222222222222222222222222222222222222222222222222222222222222222222222122222111111121222222122222222222222222222222222222222222222222222222222222

>1H8HF

AAQASPSPKAGATTGRIVAVIGAVVDVQFDEGLPPILNALEVQGRETRLVLEVAQHLGESTVRTIAMDGTEGLVRGQKVLDSGAPIRIPVGPETLGRIMNVIGEPIDERGPIKTKQFAAIHAEAPEFVEMSVEQEILVTGIKVVDLLAPYAKGGKIGLFGGAGVGKTVLIMELINNVAKAHGGYSVFAGVGERTREGNDLYHEMIESGVINLKDATSKVALVYGQMNEPPGARARVALTGLTVAEYFRDQEGQDVLLFIDNIFRFTQAGSEVSALLGRIPSAVGYQPTLATDMGTMQERITTTKKGSITSVQAIYVPADDLTDPAPATTFAHLDATTVLSRAIAELGIYPAVDPLDSTSRIMDPNIVGSEHYDVARGVQKILQDYKSLQDIIAILGMDELSEEDKLTVSRARKIQRFLSQPFQVAEVFTGHLGKLVPLKETIKGFQQILAGEYDHLPEQAFYMVGPIEEAVAKADKLAEEHS

22222222222222222222222222222222222222222222222222222222222222222222222222222222222222222222222222222222222222222222222222222222222222222222222222222222222222221111111122222222222222222222222212222222222222222222222222222222222222222222222222222222222222222222222222222222222222222222222222222222222222222222222222222222222222222222222222222222222212222222222222222222222122222222222222222222222222222222222222222222222221221221122222222222222222222222222222222222222222222222222222

>2Q0DA

MGSSHHHHHHSSGLVPRGSHMPPSPAVVGRSLVNSFKQFVSKDLHTRHVDATYRLVLDCVAAVDPLMRLYTFGSTVVYGVHEKGSDVDFVVLNKTDVEDGKGGDAATQVAKGLQADILAKLARVIRQKHLSWNVEEVRRTRVPVVRVKGGGAVDFDITAYRRNGVRNSALLRAYFEQNPPCRWLSMSIKRWSKQTGLNASVIGGSITSYGFNLMVVYYLLQRNHLQFVPPSTIDVSRVEPLPPHLPLEEPADEGLELGTQVLDFLHFFLHEFDSDKQVISLNRPGITTKEELDWTKSAEDFARMNGEKVHYQWCIEDPYELNLNVGRNVTPLKRDFLRRHLEKARDTALLTIV

22222222222222222222222222222222222222222222222222222222222222222222222111222222222211212222222222222222222222222222222222222222222222222222122222222222222222222221221122222222222222222222122212222222222222111222222222222222222222222222222222222222222222222222222222222222222222222222222222222222222222222222222222222222221212122222222222222222222222222

>1XDPA

GQEKLYIEKELSWLSFNERVLQEAADKSNPLIERMRFLGIYSNNLDEFYKVRFAELKRRIIISEEQGSNSHSRHLLGKIQSRVLKADQEFDGLYNELLLEMARNQIFLINERQLSVNQQNWLRHYFKQYLRQHITPILINPDTDLVQFLKDDYTYLAVEIIRGDTIRYALLEIPSDKVPRFVNLPPEAPRRRKPMILLDNILRYCLDDIFKGFFDYDALNAYSMKMTRDAEYDLVHEMEASLMELMSSSLKQRLTAEPVRFVYQRDMPNALVEVLREKLTISRYDSIVPGGRYHNFKDFINFPNVGKANLVNKPLPRLRHIWFDKAQFRNGFDAIRERDVLLYYPYHTFEHVLELLRQASFDPSVLAIKINIYRVAKDSRIIDSMIHAAHNGKKVTVVVELQARFDEEANIHWAKRLTEAGVHVIFSAPGLKIHAKLFLISRKENGEVVRYAHIGTGNFNEKTARLYTDYSLLTADARITNEVRRVFNFIENPYRPVTFDYLMVSPQNSRRLLYEMVDREIANAQQGLPSGITLKLNNLVDKGLVDRLYAASSSGVPVNLLVRGMCSLIPNLEGISDNIRAISIVDRYLEHDRVYIFENGGDKKVYLSSADWMTRNIDYRIEVATPLLDPRLKQRVLDIIDILFSDTVKARYIDKELSNRYVPRGNRRKVRAQLAIYDYIKSLEQPE

222222222222222122222222222222222222222122212222222222222222222222222222222222222222222222222222222222222222222222222222222222222222222222222222222222222222222222222222222222222222222222222222222222222222222222222222222222222222222222222222222222222222222222222222222222222222222222222222222222222222222222222222222222222222222222222222222222222222222222222222222222222222112222222222222222222222222222212222222222222222222222222222212222222222222222222222222222222212222222222222222222222222222222222222222222222222222222222222222222222222222222222222222222222212222222222222222222222222121212222222222222222222222222222222222222222222222222222222222222222222222222222222222222222222222

>1V1BA

MLEVVTAGEPLVALVPQEPGHLRGKRLLEVYVGGAEVNVAVALARLGVKVGFVGRVGEDELGAMVEERLRAEGVDLTHFRRAPGFTGLYLREYLPLGQGRVFYYRKGSAGSALAPGAFDPDYLEGVRFLHLSGITPALSPEARAFSLWAMEEAKRRGVRVSLDVNYRQTLWSPEEARGFLERALPGVDLLFLSEEEAELLFGRVEEALRALSAPEVVLKRGAKGAWAFVDGRRVEGSAFAVEAVDPVGAGDAFAAGYLAGAVWGLPVEERLRLANLLGASVAASRGDHEGAPYREDLEVLLKATQTFMR

222222222222222222222222222222222222222222222222222222222222222222222222222222222222222222222222222222222222222222222222222222222222222222222222222222222222222222221222222222222222222222222222122222222222222222222222221212212222222222222112122221211112122222222222222222222212211221222222222222222222222222222

>2ILYA

GEIQWMRPSKEVGYPIINAPSKTKLEPSAFHYVFEGVKEPAVLTKNDPRLKTDFEEAIFSKYVGNKITEVDEYMKEAVDHYAGQLMSLDINTEQMULEDAMYGTDGLEALDLSTSAGYPYVAMGKKKRDILNKQTRDTKEMQKLLDTYGINLPLVTYVKDELRSKTKVEQGKSRLIEASSLNDSVAMRMAFGNLYAAFHKNPGVITGSAVGUDPDLFWSKIPVLMEEKLFAFDYTGYDASLSPAWFEALKMVLEKIGFGDRVDYIDYLNHSHHLYKNKTYUVKGGMPSGUSGTSIFNSMINNLIIRTLLLKTYKGIDLDHLKMIAYGDDVIASYPHEVDASLLAQSGKDYGLTMTPADKSATFETVTWENVTFLKRFFRADEKYPFLIHPVMPMKEIHESIRWTKDPRNTQDHVRSLCLLAWHNGEEEYNKFLAKIRSVPIGRALDLPEYSTLYDRWLDSF

22222222222222222222222222222222222222222222222222222222222222222222222222222222222222222222222222222222222222222222222222222222222222222222222222222222222222122212221222222111222222222222222222222222222222222222222222222222222222222111112222222222222222222222222222222222222222222222222122222222222222222222222222222222222222212222222222222222222222222222221222222222222222222222222222222222222222222222222222222222222222222222222222222222222222222222222222222

>3GNIB

MAHHHHHHMENLYFQGMSSFLPEGGCYELLTVIGKGFEDLMTVNLARYKPTGEYVTVRRINLEACSNEMVTFLQGELHVSKLFNHPNIVPYRATFIADNELWVVTSFMAYGSAKDLICTHFMDGMNELAIAYILQGVLKALDYIHHMGYVHRSVKASHILISVDGKVYLSGLRSNLSMISHGQRQRVVHDFPKYSVKVLPWLSPEVLQQNLQGYDAKSDIYSVGITACELANGHVPFKDMPATQMLLEKLNGTVPCLLDTSTIPAEELTMSPSRSVANSGLSDSLTTSTPRPSNGDSPSHPYHRTFSPHFHHFVEQCLQRNPDARPSASTLLNHSFFKQIKRRASEALPELLRPVTPITNFEGSQSQDHSGIFGLVTNLEELEVDDWEF

22222222222222222222222222222222111112221212222222222221212222222222222222222222222222222222222222222222112122212212222222222222222222222222222222222222222211212222222222221222222222222222222222222222222222222222222222222222222222222222222222222222222222222222222222222222222222222222222222222222222222222222222222222222222222222222222222222222222222222222222222222222222222222222222222222

>1S9IA

EAFLTQKAKVGELKDDDFERISELGAGNGGVVTKVQHRPSGLIMARKLIHLEIKPAIRNQIIRELQVLHECNSPYIVGFYGAFYSDGEISICMEHMDGGSLDQVLKEAKRIPEEILGKVSIAVLRGLAYLREKHQIMHRDVKPSNILVNSRGEIKLCDFGVSGQLIDSMANSFVGTRSYMAPERLQGTHYSVQSDIWSMGLSLVELAVGRYPIPPPDAKELEAIFGRPVVDGEEGEPHSISPRPRPPGRPVSGHGMDSRPAMAIFELLDYIVNEPPPKLPNGVFTPDFQEFVNKCLIKNPAERADLKMLTNHTFIKRSEVEEVDFAGWLCKTLRLNQPGTPTRTAVLEHHHHHH

222222222222222222222221111121212222222222221212222222222222222222222222222222222222222222221111222122122222222222222222222222222222222222212121221222222222212222222222222222222222222222222222222222222222222222222222222222222222222222222222222222222222222222222222222222222222222222222222222222222222222222222222222222222222222222222222222222222222222222

>1HP1A

YEQDKTYKITVLHTNDHHGHFWRNEYGEYGLAAQKTLVDGIRKEVAAEGGSVLLLSGGDINTGVPESDLQDAEPDFRGMNLVGYDAMAIGNHEFDNPLTVLRQQEKWAKFPLLSANIYQKSTGERLFKPWALFKRQDLKIAVIGLTTDDTAKIGNPEYFTDIEFRKPADEAKLVIQELQQTEKPDIIIAATHMGHYDNGEHGSNAPGDVEMARALPAGSLAMIVGGHSQDPVCMAAENKKQVDYVPGTPCKPDQQNGIWIVQAHEWGKYVGRADFEFRNGEMKMVNYQLIPVNLKKKRVLYTPEIAENQQMISLLSPFQNKGKAQLEVKIGETNGRLEGDRDKVRFVQTNMGRLILAAQMDRTGADFAVMSGGGIRDSIEAGDISYKNVLKVQPFGNVVVYADMTGKEVIDYLTAVAQMKPDSGAYPQFANVSFVAKDGKLNDLKIKGEPVDPAKTYRMATLNFNATGGDGYPRLDNKPGYVNTGFIDAEVLKAYIQKSSPLDVSVYEPKGEVSWQ

222222222222222222222222222222222222222222222222222222222222222222222222222222222222222222222222222222222222222222222222222222222222222222222222222222222222222222222222222222222222222222222222222222222222222222222222222222222222222222222222222222222222222222222222222222222222222222222222222222222222222222222222222222222222222222222222222212221222222222222222222222222212112122222222222222222212122222222222222222222222222122222222222222222222222222222222222222212222212222222222222222222222222222222222222222222222

>2FGHA

MVVEHPEFLKAGKEPGLQIWRVEKFDLVPVPPNLYGDFFTGDAYVILKTVQLRNGILQYDLHYWLGNECSQDESGAAAIFTVQLDDYLNGRAVQHREVQGFESATFLGYFKSGLKYKKGGVASGFKHVVPNEVVVQRLLQVKGRRVVRATEVPVSWESFNNGDCFILDLGNNIYQWCGSKSNRFERLKATQVSKGIRDNERSGRAQVSVFEEGAEPEAMLQVLGPKPTLPEATEDTVKEDAANRKLAKLYKVSNGAGPMVVSLVADENPFAQGALRSEDCFILDHGKDGKIFVWKGKQANMEERKAALKTASDFISKMDYPKQTQVSVLPEGGETPLFRQFFKNWRDPDQTEGLGLAYLSSHIAHVERVPFDAATLHTSTAMAAQHGMDDDGTGQKQIWRVEGSNKVPVDPATYGQFYGGDSYIILYNYRHGSRQGQIIYNWQGAQSTQDEVAASAILTAQLDEELGGTPVQSRVVQGKEPAHLMSLFGGKPMIVYKGGTSREGGQTAPASTRLFQVRASSSGATRAVEIIPKAGALNSNDAFVLKTPSAAYLWVGAGASEAEKTGAQELLRVLRAQPVQVAEGSEPDSFWEALGGKATYRTSPRLKDKKMDAHPPRLFACSNKIGRFVIEEVPGEFMQEDLATDDVMLLDTWDQVFVWVGKDSQDEEKTEALTSAKRYIDTDPAHRDRRTPITVVKQGFEPPSFVGWFLGWDDSYWSVDPLDRALAELAA

22222222222222222222222222222222222222222222222222222222222222222222222222222222222222222222222222222222222222222222222222222222222222222222222222222222222222222222222222222222222222222222222222222222222222222222222222222222222222222222222222222222222222222222222222222222222222222222222222222222222222222222222222222222222222222222222222122221111222222222222222222222222222222222222222222222222222222222222222222222222222222222222222222222222222222222222222222222222222222222221222222222222222222222222222222222222221222222222222222222222222222222222222222222222222222222222222222222222222222222222222221122222211222222222222222222222222222222222222222222222222222222222222222222222222222222222222222222222222222222222222222222222

>1ATPE

GNAAAAKKGSEQESVKEFLAKAKEDFLKKWETPSQNTAQLDQFDRIKTLGTGSFGRVMLVKHKESGNHYAMKILDKQKVVKLKQIEHTLNEKRILQAVNFPFLVKLEFSFKDNSNLYMVMEYVAGGEMFSHLRRIGRFSEPHARFYAAQIVLTFEYLHSLDLIYRDLKPENLLIDQQGYIQVTDFGFAKRVKGRTWULCGTPEYLAPEIILSKGYNKAVDWWALGVLIYEMAAGYPPFFADQPIQIYEKIVSGKVRFPSHFSSDLKDLLRNLLQVDLTKRFGNLKNGVNDIKNHKWFATTDWIAIYQRKVEAPFIPKFKGPGDTSNFDDYEEEEIRVUINEKCGKEFTEF

22222222222222222222222222222222222222222222222211111112122222222222212122222222222222222222222222222221222222222222222112122212222222222222222222222222222222222222212121121222222222112222222222222222222222222222222222222222222222222222222222222222222222222222222222222222222222222222222222222222222222222222222222222222222222122222222222222222222222

>3CQDA

MVRIYTLTLAPSLDSATITPQIYPEGKLRCTAPVFEPGGGGINVARAIAHLGGSATAIFPAGGATGEHLVSLLADENVPVATVEAKDWTRQNLHVHVEASGEQYRFVMPGAALNEDEFRQLEEQVLEIESGAILVISGSLPPGVKLEKLTQLISAAQKQGIRCIVDSSGEALSAALAIGNIELVKPNQKELSALVNRELTQPDDVRKAAQEIVNSGKAKRVVVSLGPQGALGVDSENCIQVVPPPVKSQSTVGAGDSMVGAMTLKLAENASLEEMVRFGVAAGSAATLNQGTRLCSHDDTQKIYAYLSR

222222222222222222222222222222222222222222222222222222222222222222222222222222222222222222222222222222222222222222222222222222222222222222222222222222222222222222222222222222222222222212121222222222222222222222222222222222211112122222222222222222212212211221222222222222222222222122112212222222222222222222222

>1G21E

MAMRQCAIYGKGGIGKSTTTQNLVAALAEMGKKVMIVGCDPKADSTRLILHSKAQNTIMEMAAEAGTVEDLELEDVLKAGYGGVKCVESGGPEPGVGCAGRGVITAINFLEEEGAYEDDLDFVFYDVGDVVCGGFAMPIRENKAQEIYIVCSGEMMAMYAANNISKGIVKYANSGSVRLGGLICNSRNTDREDELIIALANKLGTQMIHFVPRDNVVQRAEIRRMTVIEYDPKAKQADEYRALARKVVDNKLLVIPNPITMDELEELLMEFGIMEVEDESIVGKTAEEV

2222222222211111112222222222222222222221212222222222222222222222222222222222222222222222222222222222222222222222222222222222222222222222222222222222222222212222222222222222222222222222122222222222222222222222221111221222122222222222222122222222222222222222222222222222222222222222222222222

>3B2QA

MKHHHHHHPMVKEYKTITQIAGPLIFVEKTEPVGYNEIVNIKMGDGTVRRGQVLDSSADIVVVQVFEGTGGLDKDCGVIFTGETLKLPASVDLLGRILSGSGEPRDGGPRIVPDQLLDINGAAMNPYARLPPKDFIQTGISTIDGTNTLVRGQKLPIFSASGLPHNEIALQIARQASVPGSESAFAVVFAAMGITNEEAQYFMSDFEKTGALERAVVFLNLADDPAVERIVTPRMALTAAEYLAYEHGMHVLVILTDITNYAEALRQMGAARNEVPGRRGYPGYMYTDLATLYERAGIVKGAKGSVTQIPILSMPGDDITHPIPDLSGYITEGQIVVARELHRKGIYPPINVLPSLSRLMNSGIGAGKTREDHKAVSDQMYAGYAEGRDLRGLVAIVGKEALSERDTKFLEFADLFEDKFVRQGWNENRTIEDTLEIGWQILTHLPENQLGRIDNKYIQKYHPAHRKAK

2222222222222222222222222222222222222222222222222222222222222222222222222222222222222222222222222222222222222222222222222222222222222222222222222222222222222121221222222222222222222222222222222222222222222222222222222222222222222222222222222222222222222222222222222222222222222222222222222222222222222222222222222222222222222112212221211112222222222222212222222222222222222222222222222222222222222222222222222222222222222222222222222222222222222222222222222222222222222

>1ZP9A

UKDLKKIESYLDKLRIKEKDGEERKIYAEVLDGRTLKTLYKLSAKGYITAUGGVISTGKEANVFYADGVFDGKPVAUAVKIYRIETSEFDKUDEYLYGDERFDURRISPKEKVFIWTEKEFRNLERAKEAGVSVPQPYTYUKNVLLUEFIGEDELPAPTLVELGRELKELDVEGIFNDVVENVKRLYQEAELVHADLSEYNIUYIDKVYFIDUGQAVTLRHPUAESYLERDVRNIIRFFSKYGVKADFEEULKEVKGE

222222222222222222222222222222222222222222222222222222112221121222222222222221212122222222222222222222222222222222222222222222222222221222222222222111222222221222222222222222222222222222222222222222221222222222112222222222222222222222222222222222222222222222

>3FKQA

UGSDKIHHHHHHENLYFQGUKIKVALLDKDKEYLDRLTGVFNTKYADKLEVYSFTDEKNAIESVKEYRIDVLIAEEDFNIDKSEFKRNCGLAYFTGTPGIELIKDEIAICKYQRVDVIFKQILGVYSDUAANVATISGENDKSSVVIFTSPCGGVGTSTVAAACAIAHANUGKKVFYLNIEQCGTTDVFFQAEGNATUSDVIYSLKSRKANLLLKLESCIKQSQEGVSYFSSTKVALDILEISYADIDTLIGNIQGUDNYDEIIVDLPFSLEIEKLKLLSKAWRIIVVNDGSQLSNYKFURAYESVVLLEQNDDINIIRNUNUIYNKFSNKNSEULSNISIKTIGGAPRYEHATVRQIIEALTKUEFFEEILQ

2222222222222222222222222222222222222222222222222222222222222222222222222222222222222222222222222222222222222222222222222222222222222222222222222222222211111112222222222222222222221222222222222222222222222222222222222222222222222222222222222222222222222222222222222222222222222222222222222222222222222222222222222222222222222112222222222222222222211122222221222222222222222

>3HGMA

MFNRIMVPVDGSKGAVKALEKGVGLQQLTGAELYILCVFKHHSLLEASLSMARPEQLDIPDDALKDYATEIAVQAKTRATELGVPADKVRAFVKGGRPSRTIVRFARKRECDLVVIGAQGTNGDKSLLLGSVAQRVAGSAHCPVLVV

222222211121211222222222222222222221212222222222222222222222222222222222222222222222222222222222212221222222222222211211111222222111121222222222222

>3H8VA

MGSSHHHHHHSSGLVPRGSMALKRMGIVSDYEKIRTFAVAIVGVGGVGSVTAEMLTRCGIGKLLLFDYDKVELANMNRLFFQPHQAGLSKVQAAEHTLRNINPDVLFEVHNYNITTVENFQHFMDRISNGGLEEGKPVDLVLSCVDNFEARMTINTACNELGQTWMESGVSENAVSGHIQLIIPGESACFACAPPLVVAANIDEKTLKREGVCAASLPTTMGVVAGILVQNVLKFLLNFGTVSFYLGYNAMQDFFPTMSMKPNPQCDDRNCRKQQEEYKKKVAALPKQEVIQ

2222222222222222222222222222222222222222221211222222222222222222221112222222222222222222212222222222222222222222111222222222222222222222222222211112212222222222222222222222222222222222222222222222222222222222222222222222222222222222222222222222222222222222222222222222222222222222222222222222

>2J9LA

MEAKEEFAHKTLAMDVMKPRRNDPLLTVLTQDSMTVEDVETIISETTYSGFPVVVSRESQRLVGFVLRRDLIISIENARKKQDGVVSTSIIYFTEHSPPLPPYTPPTLKLRNILDLSPFTVTDLTPMEIVVDIFRKLGLRQCLVTHNGRLLGIITKKDVLKHIAQMANQDPDSILFNEFLEVLFQ

22222222222222222122222221122222222222222222221111112222222222222222222222222222222222222222222222222222222222222222222222222222222222222222222222222222121211222222222222222222222222222

>1E4GT

UIDLSKTVFYTSIDIGSRYIKGLVLGKRDQEWEALAFSSVKSRGLDEGEIKDAIAFKESVNTLLKELEEQLQKSLRSDFVISFSSVSFEREDTVIERDFGEEKRSITLDILSEUQSEALEKLKENGKTPLHIFSKRYLLDDERIVFNPLDUKASKIAIEYTSIVVPLKVYEUFYNFLQDTVKSPFQLKSSLVSTAEGVLTTPEKDRGVVVVNLGYNFTGLIAYKNGVPIKISYVPVGUKHVIKDVSAVLDTSFEESERLIITHGNAVYNDLKEEEIQYRGLDGNTIKTTTAKKLSVIIHARLREIUSKSKKFFREVEAKIVEEGEIGIPGGVVLTGGGAKIPRINELATEVFKSPVRTGCYANSDRPSIINADEVANDPSFAAAFGNVFAVSENPYEETPVKSENPLKKIFRLFKELUE

22222222222222211112122222222222222222222222221222222222222222222222222222222222222122222222222222222222222222222222222222222222222222222222222222222222222222222222222222222222222222222222222222222222222222222222111222222222222222222222222221222222222222221222222222222222222222222222222222222222222222222222222222222222222222222222222212211222222222222222222222222222222222222221222222222222222222222222222222222222222

>1F2UB

KYKALAREAALSKIGELASEIFAEFTEGKYSEVVVRAEENKVRLFVVWEGKERPLTFLSGGERIALGLAFRLAMSLYLAGEISLLILDEPTPYLDEERRRKLITIMERYLKKIPQVILVSHDEELKDAADHVIRISLENGSSKVEVVS

2222222222222222222222222222112222222222222222222222222212111122222222222222222222222222122222222222222222222222222222222222222222222222222222222222

>1F2UA

MKLERVTVKNFRSHSDTVVEFKEGINLIIGQNGSGKSSLLDAILVGLYWPLRIKDIKKDEFTKVGARDTYIDLIFEKDGTKYRITRRFLKGYSSGEIHAMKRLVGNEWKHVTEPSSKAISAFMEKLIPYNIFLNAIYIRQGQIDAILES

22222222222112222222222222222211111111222222222222222222222121112222222222222222222222222222222222222222222222222222222222222222222222222221222222222

>1FMWA

MNPIHDRTSDYHKYLKVKQGDSDLFKLTVSDKRYIWYNPDPKERDSYECGEIVSETSDSFTFKTVDGQDRQVKKDDANQRNPIKFDGVEDMSELSYLNEPAVFHNLRVRYNQDLIYTYSGLFLVAVNPFKRIPIYTQEMVDIFKGRRRNEVAPHIFAISDVAYRSMLDDRQNQSLLITGESGAGKTENTKKVIQYLASVAGRNQANGSGVLEQQILQANPILEAFGNAKTTRNNNSSRFGKFIEIQFNNAGFISGASIQSYLLEKSRVVFQSETERNYHIFYQLLAGATAEEKKALHLAGPESFNYLNQSGCVDIKGVSDSEEFKITRQAMDIVGFSQEEQMSIFKIIAGILHLGNIKFEKGAGEGAVLKDKTALNAASTVFGVNPSVLEKALMEPRILAGRDLVAQHLNVEKSSSSRDALVKALYGRLFLWLVKKINNVLCQERKAYFIGVLDISGFEIFKVNSFEQLCINYTNEKLQQFFNHHMFKVEQEEYLKEKINWTFIDFGLDSQATIDLIDGRQPPGILALLDEQSVFPNATDNTLITKLHSHFSKKNAKYEEPRFSKTEFGVTHYAGQVMYEIQDWLEKNKDPLQQDLELCFKDSSDNVVTKLFNDPNIASRAKKGANFITVAAQYKEQLASLMATLETTNPHFVRCIIPNNKQLPAKLEDKVVLDQLRCNGVLEGIRITRKGFPNRIIYADFVKRYYLLAPNVPRDAEDSQKATDAVLKHLNIDPEQYRFGITKIFFRAGQLARIEEAREPN

22222222222222222222222222222222222222222222222222222222222222222222222222222222222222222222222222222222222222222212222222222211112222122222222222222222222222222222222222222222222111111112222222222222222222222222222222222222222222221221122222222222222222222222222222222222222222222222222222222222222222222222222222222222222222222222222222222222222222222222222222222222222222222222222222222222222222222222222222222222222222222222222222222222222222222222222222222222222222222222222222222222222222222222222222222222222222222222222222222222222222222222222222222222222222222222222222222222222222222222222222222222222222222222222222222222222222222222222222222222222222222222222222222222222222222222222222222222222222222222222222222222222222222222222222222222222222222

>2J3MA

MKQSKMLIPTLREVPNDAEVLSHQILLRAGYIRQVAAGIYSYLPLANRVLEKLKTIMREEFEKIDAVEMLMPALLPAELWKESGRYETYGPNLYRLKDRNDRDYILGPTHEETFTELIRDEINSYKRLPLNLYQIQTKYRDEKRSRSGLLRGREFIMKDGYSFHADEASLDQSYRDYEKAYSRIFERCGLEFRAIIGDGGAMGGKDSKEFMAISEIGEDTICYSTESDYAANLEMATSLYTPKKSHETQLDLEKIATPEVGTIAEVANFFEVEPQRIIKSVLFIADEEPVMVLVRGDHDVNDVKLKNFLGADFLDEATEEDARRVLGAGFGSIGPVNVSEDVKIYADLAVQDLANAIVGANEDGYHLTNVNPDRDFQPISYEDLRFVQEGDPSPDGNGVLAFTKGIEIGHIFKLGTRYSDAMGATVLDENGREKSVIMGCYGIGVSRLLSAIVEQNADERGINWPTGIAPFDLHVVQMNVKDEYQTKLSQEVEAMMTEAGYEVLVDDRNERAGVKFADADLIGCPIRITVGKKAVDGVVEVKIKRTGEMLEVRKEELESTLSILMNTTSEVE

22222222222222222222222222222222222222222222222222222222222222222222222222222222222222222222222222222222222222222222222222222222222222222221212222221111221212222222222222222222222222222222222222222222222222222222222222222222222222222222222222222222222222222222222222222222222222222222222222222222222222222222222222222222222222222222222222222222222222222222222222222222222222222222222222222222222222222222221111212222222222222222222222222222212122122222222222222222222222222222222222222222222222222222222222222222222222222222222222222222222222222222222222222222222222222222

>1E8XA

AASEETLAFQRQLNALIGYDVTDVSNVHDDELEFTRRRLVTPRMAEVAGRDPKLYAMHPWVTSKPLPEYLLKKITNNCVFIVIHRSTTSQTIKVSADDTPGTILQSFFTKMAKKKSLMDIPESQNERDFVLRVCGRDEYLVGETPIKNFQWVRQCLKNGEEIHLVLDTPPDPALDEVRKEEWPLVDDCTGVTGYHEQLTIHGKDHESVFTVSLWDCDRKFRVKIRGIDIPVLPRTADLTVFVEANIQYGQQVLCQRRTSPKPFTEEVLWNVWLEFSIKIKDLPKGALLNLQIYCGKAPALSGKTSAEMPSPESKGKAQLLYYVNLLLIDHRFLLRHGEYVLHMWQLSGKGEDQGSFNADKLTSATNPDKENSMSISILLDNYCHPIALPKHRPTPDPEGDRVRAEMPNQLRKQLEAIIATDPLNPLTAEDKELLWHFRYESLKDPKAYPKLFSSVKWGQQEIVAKTYQLLAKREVWDQSALDVGLTMQLLDCNFSDENVRAIAVQKLESLEDDDVLHYLLQLVQAVKFEPYHDSALARFLLKRGLRNKRIGHFLFWFLRSEIAQSRHYQQRFAVILEAYLRGCGTAMLHDFTQQVQVIDMLQKVTIDIKSLSAEKYDVSSQVISQLKQKLENLQNLNLPQSFRVPYDPGLKAGALVIEKCKVMASKKKPLWLEFKCADPTALSNETIGIIFKHGDDLRQDMLILQILRIMESIWETESLDLCLLPYGCISTGDKIGMIEIVKDATTIAKIQQSTVGNTGAFKDEVLSHWLKEKCPIEEKFQAAVERFVYSCAGYCVATFVLGIGDRHNDNIMISETGNLFHIDFGHILGNYKSFLGINKERVPFVLTPDFLFVMGTSGKKTSLHFQKFQDVCVKAYLALRHHTNLLIILFSMMLMTGMPQLTSKEDIEYIRDALTVGKSEEDAKKYFLDQIEVCRDKGWTVQFNWFLHLVLGIKQGEKHSA

2222222222222222222222222222222222222222222222222222222222222222222222222222222222222222222222222222222222222222222222222222222222222222222222222222222222222222222222222222222222222222222222222222222222222222222222222222222222222222222222222222222222222222222222222222222222222222222222222222222222222222222222222222222222222222222222222222222222222222222222222222222222222222222222222222222222222222222222222222222222222222222222222222222222222222222222222222222222222222222222222222222222222222222222222222222222222222222222222222222222222222222222222222222222222222222222222222222222222222222222222222222222222222222222222222222222222222222222222222222222222212111212222222222222222222212122122222222222222222222222222222212222222222211112222222122222222222222222222222222222222222222222222222222222222222212122222222211222222222222222222222222222222222222222222222222222222222222222222222222222222222222222222222222222222222222222222222222222222222222222222

>3C4WA

MDFGSLEUVVANSAFIAARGSFDASSGPASRDRKYLARLKLPPLSKCEALRESLDLGFEGMCLEQPIGKRLFQQFLRTHEQHGPALQLWKDIEDYDTADDALRPQKAQALRAAYLEPQAQLFCSFLDAETVARARAGAGDGLFQPLLRAVLAHLGQAPFQEFLDSLYFLRFLQWKWLEAQPMGEDWFLDFRVLGRGGFGEVFACQMKATGKLYACKKLNKKRLKKRKGYQGAMVEKKILAKVHSRFIVSLAYAFETKTDLCLVMTIMNGGDIRYHIYNVDEDNPGFQEPRAIFYTAQIVSGLEHLHQRNIIYRDLKPENVLLDDDGNVRISDLGLAVELKAGQTKTKGYAGTPGFMAPELLLGEEYDFSVDYFALGVTLYEMIAARGPFRARGEKVENKELKQRVLEQAVTYPDKFSPASKDFCEALLQKDPEKRLGFRDGSCDGLRTHPLFRDISWRQLEAGMLTPPFVPDSRTVYAKNIQDVGAFSTVKGVAFEKADTEFFQEFASGTCPIPWQEEMIETGVFGDLNVWRPDGVDHHHHHH

222222222222222222222222222222222222222222222222222222222222222222222222222222222222222222222222222222222222222222222222222222222222222222222222222222222222222222222222222222222222222222222222111112221222222222222121222222222222222222222222222222222222222222222221121222222222222222222222222222222222222222222222222222221222222222212222222222222222222222222222222222222222222222222222222222222222222222222222222222222222222222222222222222222222222222222222222222222222222222222222222222222222222222222222222222222222222222222222222222222222222

>1Q97A

DYRPGGYHPAFKGEPYKDARYILVRKLGWGHFSTVWLAKDMVNNTHVAMKIVRGDKVYTEAAEDEIKLLQRVNDADNTKEDSMGANHILKLLDHFNHKGPNGVHVVMVFEVLGENLLALIKKYEHRGIPLIYVKQISKQLLLGLDYMHRRCGIIHTDIKPENVLMEIVDSPENLIQIKIADLGNACWYDEHYTNSIQTREYRSPEVLLGAPWGCGADIWSTACLIFELITGDFLFEPDEGHSYTKDDDHIAQIIELLGELPSYLLRNGKYTRTFFNSRGLLRNISKLKFWPLEDVLTEKYKFSKDEAKEISDFLSPMLQLDPRKRADAGGLVNHPWLKDTLGMEEIRVPDRELYGSGSDIPGWFEEVRDHKRH

2222222222222222222222222221211112122222222222212122222222222222222222222222222222222222122222222222222222222111121222222222222222222222222222222222222222222222112122222222222222221222222222222222222222222222222222222222222222222222222222222222222222222222222222222222222222222222222222222222222222222222222222222222222222222222222222222222222222222222222222222222222222222

>3D2EA

USTPFGLDLGNNNSVLAVARNRGIDIVVNEVSNRSTPSVVGFGPKNRYLGETGKNKQTSNIKNTVANLKRIIGLDYHHPDFEQESKHFTSKLVELDDKKTGAEVRFAGEKHVFSATQLAAUFIDKVKDTVKQDTKANITDVCIAVPPWYTEEQRYNIADAARIAGLNPVRIVNDVTAAGVSYGIFKTDLPEGEEKPRIVAFVDIGHSSYTCSIUAFKKGQLKVLGTACDKHFGGRDFDLAITEHFADEFKTKYKIDIRENPKAYNRILTAAEKLKKVLSANTNAPFSVESVUNDVDVSSQLSREELEELVKPLLERVTEPVTKALAQAKLSAEEVDFVEIIGGTTRIPTLKQSISEAFGKPLSTTLNQDEAIAKGAAFICAIHSPTLRVRPFKFEDIHPYSVSYSWDKQVEDEDHUEVFPAGSSFPSTKLITLNRTGDFSUAASYTDITQLPPNTPEQIANWEITGVQLPEGQDSVPVKLKLRCDPSGLHTIEEAYTIEDIEAGSDTKTVKKDDLTIVAHTFGLDAKKLNELIEKENEULAQDKLVAETEDRKNTLEEYIYTLRGKLEEEYAPFASDAEKTKLQGULNKAEEWLYDEGFDSIKAKYIAKYEELASLGNIIRGRYLAKEEEKKQAIRSKQEASQUAAUAEKLAAQRKAEAEKKEEKKDTEGDVDUD

222222222111122222222222222222222222222222222222222222222222222222221222222222222222222222222222222222222222222222222222222222222222222222222222222222222222222222222222222222222222222222222222222222222222111122222222222222222222222221222222222222222222222222222222222222212211221222222222222222222222222222222222222222222222222222222222222221112112222222222222222222222222222222222222222222222222222222222222222222222222222222222222222222222222222222222222222222222222222222222222222222222222222222222222222222222222222222222222222222222222222222222222222222222222222222222222222222222222222222222222222222222222222222222222222222222222222222222222222222222222222222222222222

>3G6VA

MELADVGAAASSQGVHDQVLPTPNASSRVIVHVDLDCFYAQVEMISNPELKDKPLGVQQKYLVVTCNYEARKLGVKKLMNVRDAKEKCPQLVLVNGEDLTRYREMSYKVTELLEEFSPVVERLGFDENFVDLTEMVEKRLQQLQSDELSAVTVSGHVYNNQSINLLDVLHIRLLVGSQIAAEMREAMYNQLGLTGCAGVASNKLLAKLVSGVFKPNQQTVLLPESCQHLIHSLNHIKEIPGIGYKTAKCLEALGINSVRDLQTFSPKILEKELGISVAQRIQKLSFGEDNSPVILSGPPQSFSEEDSFKKCSSEVEAKNKIEELLASLLNRVCQDGRKPHTVRLIIRRYSSEKHYGRESRQCPIPSHVIQKLGTGNYDVMTPMVDILMKLFRNMVNVKMPFHLTLLSVCFCNLKALNTAK

222222222222222222222222222222222111111222222222222222222212222112212212222211222222222222222222222222222222222222222222222221222222222222222222222222222222222222222222222222222222222222222222222222222222222222222122222222222222222222222222222222222222222222222222222222222222222222222222222222222222222222222222222222222222222222222222222222222222222222222222222222222222222222222222222222222222222222222222222222222222

>1B76A

AASSLDELVALCKRRGFIFQSSEIYGGLQGVYDYGPLGVELKNNLKQAWWRRNVYERDDMEGLDASVLTHRLVLHYSGHEATFADPMVDNAKARYWTPPRYFNMMFQDLRGPRGGRGLLAYLRPETAQGIFVNFKNVLDATSRKLGFGIAQIGKAFRNEITPRNFIFRVREFEQMEIEYFVRPGEDEYWHRYWVEERLKWWQEMGLSRENLVPYQQPPESSAHYAKATVDILYRFPHGSLELEGIAQRTDFDLGSHTKDQEALGITARVLRNEHSTQRLAYRDPETGKWFVPYVIEPSAGVDRGVLALLAEAFTREELPNGEERIVLKLKPQLAPIKVAVIPLVKNRPEITEYAKRLKARLLALGLGRVLYEDTGNIGKAYRRHDEVGTPFAVTVDYDTIGQSKDGTTRLKDTVTVRDRDTMEQIRLHVDELEGFLRERLRW

2222222222222222222222222222222222222222222222222222222222222222222222222222222222222222222222222222222222222222222222222222222222222222222222222222222222221212222222111221212222222222222222222222222222222222222222222222222222222222222222221112222222222222222222222222222222222222222222222222222221212212222222222222222222222222222222222222222222222222222222222222222222222222222222222222222222222222222222222222222222222222222222222222222222

>1J7KA

MSEFLTPERTVYDSGVQFLRPKSLDEFIGQENVKKKLSLALEAAKMRGEVLDHVLLAGPPGLGKTTLAHIIASELQTNIHVTSGPVLVKQGDMAAILTSLERGDVLFIDEIHRLNKAVEELLYSAIEDFQIDIMIGKGPSAKSIRIDIQPFTLVGATTRSGLLSSPLRSRFGIILELDFYTVKELKEIIKRAASLMDVEIEDAAAEMIAKRSRGTGRIAIRLTKRVRDMLTVVKADRINTDIVLKTMEVLNIDDEGLDEFDRKILKTIIEIYRGGPVGLNALAASLGVEADTLSEVYEPYLLQAGFLARTPRGRIVTEKAYKHLKYEVPENRLF

2222222222222222221122222111222222222222222222222222222222211111112222222222222222222222222222222222222222222222222222222222222222222222222222222222222222222222222222222222222222212222222122222222222222222222222222211221222222222222222222222222222222222222222222222222222222222222222222222222222222222222222222222222222222222222222222

>2QKMB

MSFTNATFSQVLDDLSARFILNLPAEEQSSVERLCFQIEQAHWFYEDFIRAQNDQLPSLGLRVFSAKLFAHCPLLWKWSKVHEEAFDDFLRYKTRIPVRGAIMLDMSMQQCVLVKGWKASSGWGFPKGKIDKDESDVDCAIREVYEETGFDCSSRINPNEFIDMTIRGQNVRLYIIPGISLDTRFESRTRKEISKIEWHNLMDLPTFKKNKPQTMKNKFYMVIPFLAPLKKWIKKRNIANNTTKEKNISVDVDADASSQLLSLLKS

22222222222222222222222222222222222222222222222222222222222222222222222222222222222222222222222222222222222222222222222111222222222222222222222222222222222222222222221222222222222222222222222222222222222222222222222222212222222222222222222222222222222222222222222222

>2R7LA

MISKDEILEIFDKYNKDEITIATLGSHTSLHILKGAKLEGFSTVCITMKGRDVPYKRFKVADKFIYVDNFSDIKNEEIQEKLRELNSIVVPHGSFIAYCGLDNVENSFLVPMFGNRRILRWESERSLEGKLLREAGLRVPKKYESPEDIDGTVIVKFPGARGGRGYFIASSTEEFYKKAEDLKKRGILTDEDIANAHIEEYVVGTNFCIHYFYSPLKDEVELLGMDKRYESNIDGLVRIPAKDQLEMNINPSYVITGNIPVVIRESLLPQVFEMGDKLVAKAKELVPPGMIGPFCLQSLCNENLELVVFEMSARVDGGTNSFMNGGPYSFLYNGEPLSMGQRIAREIKMALQLDMIDKIIS

2222222222222222222222222222222222222222222222222222222222222222222222222222222222222222222222222222222222222222222222222222222222222222222122222222222221212222222221222222222222222222222222222222221111222222222222222222222222212122222222222222222222221212222222222222222222222222222222222222222212122222222211222222222222222222222222222222222222222222222222222

>3DKCA

GSLNTVHIDLSALNPELVQAVQHVVIGPSSLIVHFNEVIGRGHFGCVYHGTLLDNDGKKIHCAVKSLNRITDIGEVSQFLTEGIIMKDFSHPNVLSLLGICLRSEGSPLVVLPYMKHGDLRNFIRNETHNPTVKDLIGFGLQVAKGMKFLASKKFVHRDLAARNCMLDEKFTVKVADFGLARDMYDKEFDSVHNKTGAKLPVKWMALESLQTQKFTTKSDVWSFGVLLWELMTRGAPPYPDVNTFDITVYLLQGRRLLQPEYCPDPLYEVMLKCWHPKAEMRPSFSELVSRISAIFSTFIGEHYVHVNATYVNVKEG

22222222222222222222222222222222222222112111121222222222222222121222222222222222222222222222222222222222222222211112222222222222222222222222222222222222222222122211212222222222122222222222222222222222222222222222222222222222222222222222222222222222222222222222222222222222222222222222222222222222222222222222222222222

>1BCPF

GLPTHLYKNFTVQELALKLKGKNQEFCLTAFMSGRSLVRACLSDAGHEHDTWFDTMLGFAISAYALKSRIALTVEDSPYPGTPGDLLELQICPLNGYCE

222222222222222222222222222222222222222222222222222222122112212222222222222222222222222222222222222

>2NYJA

SVSAGEKPPRLYDRRSIFDAVAQSNCQELESLLPFLQRSKKRLTDSEFKDPETGKTCLLKAULNLHNGQNDTIALLLDVARKTDSLKQFVNASYTDSYYKGQTALHIAIERRNUTLVTLLVENGADVQAAANGDFFKKTKGRPGFYFGELPLSLAACTNQLAIVKFLLQNSWQPADISARDSVGNTVLHALVEVADNTVDNTKFVTSUYNEILILGAKLHPTLKLEEITNRKGLTPLALAASSGKIGVLAYILQREIHEPECRHAAAHHHHHH

222222222222222222222222222222222222222222222222222222122221221122222222222222222222222222222122221221222222211222222222222222222222222222222222222222222222222222222222222222222222222222222222222222222222222222222222222222222222222222222222222222222222222222222222222222222

>1YUNA

MGSSHHHHHHSSGLVPRGSHMGKRIGLFGGTFDPVHIGHMRSAVEMAEQFALDELRLLPNARPPHRETPQVSAAQRLAMVERAVAGVERLTVDPRELQRDKPSYTIDTLESVRAELAADDQLFMLIGWDAFCGLPTWHRWEALLDHCHIVVLQRPDADSEPPESLRDLLAARSVADPQALKGPGGQITFVWQTPLAVSATQIRALLGAGRSVRFLVPDAVLNYIEAHHLYRAPHLEHHHHHH

22222222222222222222222222211111222121122122222222222222222222222222222222222222222222222222222222222222222222222222222222222222222222222222222222222222222222222222222222222222222222222222222221121111221222222222222222222222222222222222222222

>2ZT7A

MDGAGAEEVLAPLRLAVRQQGDLVRKLKEDKAPQVDVDKAVAELKARKRVLEAKELALQPKDDIVDRAKMEDTLKRRFFYDQAFAIYGGVSGLYDFGPVGCALKNNIIQTWRQHFIQEEQILEIDCTMLTPEPVLKTSGHVDKFADFMVKDVKNGECFRADHLLKAHLQKLMSDKKCSVEKKSEMESVLAQLDNYGQQELADLFVNYNVKSPITGNDLSPPVSFNLMFKTFIGPGGNMPGYLRPETAQGIFLNFKRLLEFNQGKLPFAAAQIGNSFRNEISPRSGLIRVREFTMAEIEHFVDPSEKDHPKFQNVADLHLYLYSAKAQVSGQSARKMRLGDAVEQGVINNTVLGYFIGRIYLYLTKVGISPDKLRFRQHMENEMAHYACDCWDAESKTSYGWIEIVGCADRSCYDLSCHARATKVPLVAEKPLKEPKTVNVVQFEPSKGAIGKAYKKDAKLVMEYLAICDECYITEMEMLLNEKGEFTIETEGKTFQLTKDMINVKRFQKTLYVEEVVPNVIEPSFGLGRIMYTVFEHTFHVREGDEQRTFFSFPAVVAPFKCSVLPLSQNQEFMPFVKELSEALTRHGVSHKVDDSSGSIGRRYARTDEIGVAFGVTIDFDTVNKTPHTATLRDRDSMRQIRAEISELPSIVQDLANGNITWADVEARYPLFEGQETGKKETIEELEHHHHHH

222222222222222222222222222222222222222222222222222222222222222222222222222222222222222222222222222222222222222222222222222222222222222222222222222222222222221222222222222222222222222222222222222222222222222222222222222222222222222222222222222222222222222222222222222222222222121222222211122122222222222222222222222222222222222222222222222222222222222222222222222222222222222222222222212222222222222222111122222222222222222222222222222222222222222222222222222222222222222222222222222222222222222222222222222222222222222222212122122222222222222222222222222222222222222222222222222222222222222222222222222222222222222222222222222222222222222222222222222222222222222222222222222222222222222222222

>1A49A

SKSHSEAGSAFIQTQQLHAAMADTFLEHMCRLDIDSAPITARNTGIICTIGPASRSVETLKEMIKSGMNVARMNFSHGTHEYHAETIKNVRTATESFASDPILYRPVAVALDTKGPEIRTGLIKGSGTAEVELKKGATLKITLDNAYMEKCDENILWLDYKNICKVVDVGSKVYVDDGLISLQVKQKGPDFLVTEVENGGFLGSKKGVNLPGAAVDLPAVSEKDIQDLKFGVEQDVDMVFASFIRKAADVHEVRKILGEKGKNIKIISKIENHEGVRRFDEILEASDGIMVARGDLGIEIPAEKVFLAQKMIIGRCNRAGKPVICATQMLESMIKKPRPTRAEGSDVANAVLDGADCIMLSGETAKGDYPLEAVRMQHLIAREAEAAMFHRKLFEELARSSSHSTDLMEAMAMGSVEASYKCLAAALIVLTESGRSAHQVARYRPRAPIIAVTRNHQTARQAHLYRGIFPVVCKDPVQEAWAEDVDLRVNLAMNVGKARGFFKKGDVVIVLTGWRPGSGFTNTMRVVPVP

22222222222222222222222222222222222222222222222212212222222222222222222121221222211222222222222222222222222222222222221222222222222222222222222222222222222222222222222222222222222222222222222222222222222221222222222222222222222222222222222222222222222222222222222222221222222222222222222222222212222222222222222222222222222222222222222222222222222222222222222212221122222222222222222222222222222222222222222222222222222222222222222222222222222222222222222222222222222222222222222222222222222222222222222222222222222222222222222222

>1BCPE

DVPYVLVKTNMVVTSVAMKPYEVTPTRMLVCGIAAKLGAAASSPDAHVPFCFGKDLKRPGSSPMEVMLRAVFMQQRPLRMFLGPKQLTFEGKPALELIRMVECSGKQDCP

22222222222222222122222222222222222222222222222222222222222112221222122122222222222222222222222222222222222222

>2OGXA

UTDTTNSIKHVISPLARQTLQDRDLTRPVAGKRPIRLLPWLQVVKIGGRVUDRGADAILPLVEELRKLLPEHRLLILTGAGVRARHVFSVGLDLGLPVGSLAPLAASEAGQNGHILAAULASEGVSYVEHPTVADQLAIHLSATRAVVGSAFPPYHHHEFPGSRIPPHRADTGAFLLADAFGAAGLTIVENVDGIYTADPNGPDRGQARFLPETSATDLAKSEGPLPVDRALLDVUATARHIERVQVVNGLVPGRLTAALRGEHVGTLIRTGVRPA

222222222222222222222222222222222222222222221211122222222222222222222222222222111222122222222222222222222222222222222222222222222222222222222222222222222222222222222222222222222222222222222111211121111222222222222222222222221112222222222222222222222222222222222222222222222222

>3LKIB

USLKKTILCFGEALIDULAQPLVKKGUPRAFLQCAGGAPANVAVAVARLGGAVQFVGULGSDUFGDFLFDSFAEAGVVTDGIVRTSTAKTALAFVALDAHGERSFSFYRPPAADLLFRVEHFQDASFSDALIFHACSNSUTDADIAEVTFEGURRAQAAGAIVSFDLNFRPULWPNGENPASRLWKGLSLADVVKLSSEELDYLANTLAADANAVIQQLWQGRAQLLLVTDAAGPVHWYTRTAGGEVPTFRVQVQDSNAAGDAFVGGULYTFAQQFDDAAALIDFCHDPESIVSTLRFAAAVGALAVTRQGAFTAUPULSEVLSLIQEQSEGHHHHHH

22222222222222222222222222222222222222222222222222222222222222222222222222222222222222222222222222222222222222222222222222222222222222222222222222222222222222222222222221222222222222222222222222122222222222222222222222222222222221112221222222222222112121222221112122222222222222222222222222222222222122122222222222222222222222222222222222

>1UF9C

UGHEAKHPIIIGITGNIGSGKSTVAALLRSWGYPVLDLDALAARARENKEEELKRLFPEAVVGGRLDRRALARLVFSDPERLKALEAVVHPEVRRLLUEELSRLEAPLVFLEIPLLFEKGWEGRLHGTLLVAAPLEERVRRVUARSGLSREEVLARERAQUPEEEKRKRATWVLENTGSLEDLERALKAVLAELTGGATEGRG

22222222222222211111111222222222222222222222222222222222222222222222222222222222222222222222222222222222222222222222222222222222222222222222122212222222222222222222222222222221122222222222222222222222222

>1WKLB

MERTFVMIKPDGVRRGLVGEILARFERKGFRIAALKLMQISQELAERHYAEHREKPFFPGLVRFITSGPVVAMVLEGPGVVAEVRKMMGATHPKDALPGTIRGDFATTIDENVIHGSATLEDAQREIALFFRPEELL

22222222122222222222222222222222222222222222222222212222122212222222222222222222222212222212222222222122222211212211222222222222222222222

>2F02A

USLIVTVTUNPSIDISYLLDHLKLDTVNRTSQVTKTPGGKGLNVTRVIHDLGGDVIATGVLGGFHGAFIANELKKANIPQAFTSIKEETRDSIAILHEGNQTEILEAGPTVSPEEISNFLENFDQLIKQAEIVTISGSLAKGLPSDFYQELVQKAHAQEVKVLLDTSGDSLRQVLQGPWKPYLIKPNLEELEGLLGQDFSENPLAAVQTALTKPUFAGIEWIVISLGKDGAIAKHHDQFYRVKIPTIQAKNPVGSGDATIAGLAYGLAKDAPAAELLKWGUAAGUANAQERUTGHVDVENVKKHLUNIQVVEIAKEGHHHHHH

22222222222222222222222222222222222222222222222222222222222222222222222222222222222222222222222222222222222222222222222222222222222222222222222222222222222222222222222222222222222222221212222222222222222222222222222222222222111121222222222222212212122221111212222222222222222222222222222222222222222222222222222222222222222

>3ETHA

MKQVCVLGNGQLGRMLRQAGEPLGIAVWPVGLDAEPAAVPFQQSVITAEIERWPETALTRQLARHPAFVNRDVFPIIADRLTQKQLFDKLHLPTAPWQLLAERSEWPAVFDRLGELAIVKRRTGGYDGRGQWRLRANETEQLPAECYGECIVEQGINFSGEVSLVGARGFDGSTVFYPLTHNLHQDGILRTSVAFPQANAQQQARAEEMLSAIMQELGYVGVMAMECFVTPQGLLINELAPRVHNSGHWTQNGASISQFELHLRAITDLPLPQPVVNNPSVMINLIGSDVNYDWLKLPLVHLHWYDKEVRPGRKVGHLNLTDSDTSRLTATLEALIPLLPPEYASGVIWAQSKFG

2222222222222222222222222222222222222222222222222222222222222222222222222222222122222222222222222222222222222222222221212222111122122222222222222222222211112122122222222222222222222221222222222222222222222222222222222222222221212222222211222222222222222222222222222222222222222222222222222222222222222222222222222222222222222222222222222222222222222222222

>2FGJA

HHHHHHDITFRNIRFRYKPDSPVILDNINLSIKQGEVIGIVGRSGSGKSTLTKLIQRFYIPENGQVLIDGHDLALADPNWLRRQVGVVLQDNVLLNRSIIDNISLANPGMSVEKVIYAAKLAGAHDFISELREGYNTIVGEQGAGLSGGQRQRIAIARALVNNPKILIFDEATSALDYESEHVIMRNMHKICKGRTVIIIAARLSTVKNADRIIVMEKGKIVEQGKHKELLSEPESLYSYLYQLQSD

2222222222222222122222212222222222222222221111111122122222222222222222222222222222222222222222222222222222222222222222222222222222222222222222221111112222222222222222222222222222222222222222222222222222222222222222222222222222222222222222222222222

>1Y8PA

GGSHHHHHHGMARLENLYFQGKQPVPKQIERYSRFSPSPLSIKQFLDFGRDNACEKTSYMFLRKELPVRLANTMREVNLLPDNLLNRPSVGLVQSWYMQSFLELLEYENKSPEDPQVLDNFLQVLIKVRNRHNDVVPTMAQGVIEYKEKFGFDPFISTNIQYFLDRFYTNRISFRMLINQHTLLFGGDTNPVHPKHIGSIDPTCNVADVVKDAYETAKMLCEQYYLVAPELEVEEFNAKAPDKPIQVVYVPSHLFHMLFELFKNSMRATVELYEDRKEGYPAVKTLVTLGKEDLSIKISDLGGGVPLRKIDRLFNYMYSTAPRPSLEPTRAAPLAGFGYGLPISRLYARYFQGDLKLYSMEGVGTDAVIYLKALSSESFERLPVFNKSAWRHYKTTPEADDWSNPSSEPRDASKYKAKQ

22222222222222222222222222222222222222222222222222222222222222222222222222222222222222222222222222222222222222222222222222222222222222222222222222222222222222222222222222222222222222222222222222222222222222222222222222222222222222222222222222222222222222222221221122112222222222222222222222222222222122221222222212222111222222222222211111111122222222222222222222221222222222222222222222222222222222222222222222222222222

>2ZSFA

MSRLSEPSPYVEFDRRQWRALRMSTPLALTEEELVGLRGLGEQIDLLEVEEVYLPLARLIHLQVAARQRLFAATAEFLGEPQQNPDRPVPFIIGVAGSVAVGKSTTARVLQALLARWDHHPRVDLVTTDGFLYPNAELQRRNLMHRKGFPESYNRRALMRFVTSVKSGSDYACAPVYSHLHYDIIPGAEQVVRHPDILILEGLNVLQTGPTLMVSDLFDFSLYVDARIEDIEQWYVSRFLAMRTTAFADPESHFHHYAAFSDSQAVVAAREIWRTINRPNLVENILPTRPRATLVLRKDADHSINRLRLRKL

222222222222222222222222222222222222221221222222222222222222222222222222222222222222222222222222222111111221222222222222222222222222222222222222222222222222222222222222222222222212222222222222222222222222222222222222222222222222222222222122212221222222222222222222222222222222222222222222222222222222222222222222

>3FPBA

MEAAHSKSTEECLAYFGVSETTGLTPDQVKRHLEKYGHNELPAEEGKSLWELVIEQFEDLLVRILLLAACISFVLAWFEEGEETITAFVEPFVILLILIANAIVGVWQERNAENAIEALKEYEPEMGKVYRADRKSVQRIKARDIVPGDIVEVAVGDKVPADIRILSIKSTTLRVDQSILTGESVSVIKHTEPVPDPRAVNQDKKNMLFSGTNIAAGKALGIVATTGVSTEIGKIRDQMAATEQDKTPLQQKLDEFGEQLSKVISLICVAVWLINIGHFNDPVHGGSWIRGAIYYFKIAVALAVAAIPEGLPAVITTCLALGTRRMAKKNAIVRSLPSVETLGCTSVICSDKTGTLTTNQMSVCKMFIIDKVDGDFCSLNEFSITGSTYAPEGEVLKNDKPIRSGQFDGLVELATICALCNDSSLDFNETKGVYEKVGEATETALTTLVEKMNVFNTEVRNLSKVERANACNSVIRQLMKKEFTLEFSRDRKSMSVYCSPAKSSRAAVGNKMFVKGAPEGVIDRCNYVRVGTTRVPMTGPVKEKILSVIKEWGTGRDTLRCLALATRDTPPKREEMVLDDSSRFMEYETDLTFVGVVGMLDPPRKEVMGSIQLCRDAGIRVIMITGDNKGTAIAICRRIGIFGENEEVADRAYTGREFDDLPLAEQREACRRACCFARVEPSHKSKIVEYLQSYDEITAMTGDGVNDAPALKKAEIGIAMGSGTAVAKTASEMVLADDNFSTIVAAVEEGRAIYNNMKQFIRYLISSNVGEVVCIFLTAALGLPEALIPVQLLWVNLVTDGLPATALGFNPPDLDIMDRPPRSPKEPLISGWLFFRYMAIGGYVGAATVGAAAWWFMYAEDGPGVTYHQLTHFMQCTEDHPHFEGLDCEIFEAPEPMTMALSVLVTIEMCNALNSLSENQSLMRMPPWVNIWLLGSICLSMSLHFLILYVDPLPMIFKLKALDLTQWLMVLKISLPVIGLDEILKFIARNYLEG

2222222222222222222222222222222222222222222222222222222222222222222222222222222222222222222222222222222222222222222222222222222222222222222222222222222222222222222222222222222222222222222222222222222222221222222222222222222222222222222222222222222222222222222222222222222222222222222222222222222222222222222222222222222222222222222222222222222222222222222222222222222222222222222222222222222222222222222222222222222222222222222222222222222222222222222222222222222222222222222222222222221222212122222222222222222222112222222222222222222222222222222222222222222122222222222222222222222222222222222222222222222222222222222222222221222222222222222222222222222222222222222222222222212222222222222222222222222222222222222222222222222222222222222222222222222222222222222222222222222222222222222222222222222222222222222222222222222222222222222222222222222222222222222222222222222222222222222222222222222222222222222222222222222222222222222222222222222222222222222222222222222222222222222222222222222222

>1UA2A

MALDVKSRAKRYEKLDFLGEGQFATVYKARDKNTNQIVAIKKIKLGHRSEAKDGINRTALREIKLLQELSHPNIIGLLDAFGHKSNISLVFDFMETDLEVIIKDNSLVLTPSHIKAYMLMTLQGLEYLHQHWILHRDLKPNNLLLDENGVLKLADFGLAKSFGSPNRAYUHQVVTRWYRAPELLFGARMYGVGVDMWAVGCILAELLLRVPFLPGDSDLDQLTRIFETLGTPTEEQWPDMCSLPDYVTFKSFPGIPLHHIFSAAGDDLLDLIQGLFLFNPCARITATQALKMKYFSNRPGPTPGCQLPRPNCPVETLKEQSNPALAIKRKRTEALEQGGLPKKLIF

2222222222222222212111112122222222222212122222222222222222222222222222222222222222222222222111222222222222222222222222222222222222222222221221222222222222222222122222222222222222222222222222222222222222222222222222222222222222222222222222222222222222222222222222222222222222222222222222222222222222222222222222222222222222222222222222222222222222

>3IQ0A

USLSKVFTIGEILVEIUASKIGQPFDQPGIWNGPYPSGAPAIFIDQVTRLGVPCGIISCVGNDGFGDINIHRLAADGVDIRGISVLPLEATGSAFVTYHNSGDRDFIFNIKNAACGKLSAQHVDENILKDCTHFHIUGSSLFSFHUVDAVKKAVTIVKANGGVISFDPNIRKEULDIPEURDALHFVLELTDIYUPSEGEVLLLSPHSTPERAIAGFLEEGVKEVIVKRGNQGASYYSANEQFHVESYPVEEVDPTGAGDCFGGAWIACRQLGFDAHRALQYANACGALAVTRRGPUEGTSRLUEIETFIQRHDUSIREAAQEGHHHHHH

222222222222222222222222222222222222222222222222222222222222222222222222222222222222222222222222222222222222222222222222222222222222222222222222222222222222222222222222122222222222222222222222222212222222222222222222222222222221111212222222222222212121221121122122222222222222222222212211222222222222222222222222222222222222222222

>1CSNA

MSGQNNVVGVHYKVGRRIGEGSFGVIFEGTNLLNNQQVAIKFEPRRSDAPQLRDEYRTYKLLAGCTGIPNVYYFGQEGLHNVLVIDLLGPSLEDLLDLCGRKFSVKTVAMAAKQMLARVQSIHEKSLVYRDIKPDNFLIGRPNSKNANMIYVVDFGMVKFYRDPVTKQHIPYREKKNLSGTARYMSINTHLGREQSRRDDLEALGHVFMYFLRGSLPWQGLKAATNKQKYERIGEKKQSTPLRELCAGFPEEFYKYMHYARNLAFDATPDYDYLQGLFSKVLERLNTTEDENFDWNLL

2222222222222222211111212122222222222212122222222222222222222222222222222222222222221121222222222222222222222222222222222222222222221212212222222222222221222222222222222222222222222222222222222222222222222222222222222222222222222222222222222222222222222222222222222222222222222222222222222222222222

>3I7VA

MKKEFSAGGVLFKDGEVLLIKTPSNVWSFPKGNIEPGEKPEETAVREVWEETGVKGEILDYIGEIHYWYTLKGERIFKTVKYYLMKYKEGEPRPSWEVKDAKFFPIKEAKKLLKYKGDKEIFEKALKLKEKFKL

22222122222222222222222222222211122222222222222222222222222222221212122222222122222222222222222222222222222222222212222222222222222222

>2ZANA

MASTNTNLQKAIDLASKAAQEDKAGNYEEALQLYQHAVQYFLHVVKYEAQGDKAKQSIRAKCTEYLDRAEKLKEYLKKKEKKPQKPVKEEQSGPVDEKGNDSDGEAESDDPEKKKLQNQLQGAIVIERPNVKWSDVAGLEGAKEALKEAVILPIKFPHLFTGKRTPWRGILLFGPPGTGKSYLAKAVATEANNSTFFSISSSDLVSKWLGESEKLVKNLFQLARENKPSIIFIDEIDSLCGSRSENESEAARRIKTEFLVQMQGVGVDNDGILVLGATNIPWVLDSAIRRRFEKRIYIPLPEAHARAAMFRLHLGSTQNSLTEADFQELGRKTDGYSGADISIIVRDALMQPVRKVQSATHFKKVRGPSRADPNCIVNDLLTPCSPGDPGAIEMTWMDVPGDKLLEPVVSMWDMLRSLSSTKPTVNEQDLLKLKKFTEDFGQEG

222222222222222222222222222222222222222222222222222222222222222222222222222222222222222222222222222222222222222222222222222222222222221112122222222222222222222222222222222222111111112222222222222222222222222222222222222222222222222222222222222222222222222222222222222222222222221222222222222222222222222222221222122222222222222222222222211221222222222222222222222222222222222222222222222222222222222222222222222222222222222222222222222222222222

>1NYRA

MEQINIQFPDGNKKAFDKGTTTEDIAQSISPGLRKKAVAGKFNGQLVDLTKPLETDGSIEIVTPGSEEALEVLRHSTAHLMAHAIKRLYGNVKFGVGPVIEGGFYYDFDIDQNISSDDFEQIEKTMKQIVNENMKIERKVVSRDEAKELFSNDEYKLELIDAIPEDENVTLYSQGDFTDLCRGVHVPSTAKIKEFKLLSTAGAYWRGDSNNKMLQRIYGTAFFDKKELKAHLQMLEERKERDHRKIGKELELFTNSQLVGAGLPLWLPNGATIRREIERYIVDKEVSMGYDHVYTPVLANVDLYKTSGHWDHYQEDMFPPMQLDETESMVLRPMNCPHHMMIYANKPHSYRELPIRIAELGTMHRYEASGAVSGLQRVRGMTLNDSHIFVRPDQIKEEFKRVVNMIIDVYKDFGFEDYSFRLSYRDPEDKEKYFDDDDMWNKAENMLKEAADELGLSYEEAIGEAAFYGPKLDVQVKTAMGKEETLSTAQLDFLLPERFDLTYIGQDGEHHRPVVIHRGVVSTMERFVAFLTEETKGAFPTWLAPKQVQIIPVNVDLHYDYARQLQDELKSQGVRVSIDDRNEKMGYKIREAQMQKIPYQIVVGDKEVENNQVNVRQYGSQDQETVEKDEFIWNLVDEIRLKKHR

222222222222222222222222222222222222222222222222222222222222222222222222222222222222222222222222222222222222222222222222222222222222222222222222222222222222222222222222222222222222222222222222222222222222222222222222222222222222222222222222222222222222222222222222222222222222222222222222222222222222222222222222222222222222222222222222222222222222222222222222222212122222222211221212222222222222222222222222222222222222222222222222222222222222222222222222222222222222221222222222222111212122222222222222222222222222222221122122222222222222222222222222222222222222222222222222222222222222222222222222222222222222222222222222222222222222222222222

>3EHGA

GIRLKDELINIKQILEAADIMFIYEEEKWPENISLLNENILSMCLKEAVTNVVKHSQAKTCRVDIQQLWKEVVITVSDDGTFKGEENSFSKGHGLLGMRERLEFANGSLHIDTENGTKLTMAIPNNSK

22222222222222222222222222222222222222222222221222112111222222222222222222222122111122212221111222222222222222222222122222222222

>1JI0A

UVSDIVLEVQSLHVYYGAIHAIKGIDLKVPRGQIVTLIGANGAGKTTTLSAIAGLVRAQKGKIIFNGQDITNKPAHVINRUGIALVPEGRRIFPELTVYENLUUGAYNRKDKEGIKRDLEWIFSLFPRLKERLKQLGGTLSGGEQQULAIGRALUSRPKLLUUDEPSLGLAPILVSEVFEVIQKINQEGTTILLVEQNALGALKVAHYGYVLETGQIVLEGKASELLDNEUVRKAYLGVA

222222222222222122122222222222222222222111111112222222222222222222222222222222222222222122222222222222222222222222222222222222222222222222222222222222222222222222211222222222222222222222222222222222222222222222222222222222222222222222222222

>1JKNA

GPLGSMDSPPEGYRRNVGICLMNNDKKIFAASRLDIPDAWQMPQGGIDEGEDPRNAAIRELREETGVTSAEVIAEVPYWLTYDFPPKVREKLNIQWGSDWKGQAQKWFLFKFTGQDQEINLLGDGSEKPEFGEWSWVTPEQLIDLTVEFKKPVYKEVLSVFAPHL

222222222222222122222222222222221222222212212222222222222222222222222222222222222121222122122221222222122222222222222222222222222222222222222222221211222222222222222

>1L2TA

MIKLKNVTKTYKMGEEIIYALKNVNLNIKEGEFVSIMGPSGSGKSTMLNIIGCLDKPTEGEVYIDNIKTNDLDDDELTKIRRDKIGFVFQQFNLIPLLTALENVELPLIFKYRGAMSGEERRKRALECLKMAELEERFANHKPNQLSGGQQQRVAIARALANNPPIILADQPTGALDSKTGEKIMQLLKKLNEEDGKTVVVVTHDINVARFGERIIYLKDGEVEREEKLRGFDDR

2222222222121222222122222222222222222221111111222222222222222222222222222222222222222222222222222222222222222222222222222222222222222222212212211211112222222222222222222222221222222222222222222222222222212222222222222222222222222222222

>3IE7A

USLIYTITLNPAIDRLLFIRGELEKRKTNRVIKTEFDCGGKGLHVSGVLSKFGIKNEALGIAGSDNLDKLYAILKEKHINHDFLVEAGTSTRECFVVLSDDTNGSTUIPEAGFTVSQTNKDNLLKQIAKKVKKEDUVVIAGSPPPHYTLSDFKELLRTVKATGAFLGCDNSGEYLNLAVEUGVDFIKPNEDEVIAILDEKTNSLEENIRTLAEKIPYLVVSLGAKGSICAHNGKLYQVIPPKVQERNDTGAGDVFVGAFIAGLAUNUPITETLKVATGCSASKVUQQDSSSFDLEAAGKLKNQVSIIQLEEREGHHHHHH

22222222222222222222222222222222222222222222222222222222222222222222222222222222222222222222222222222222222222222222222222222222222222222222222222222222222222222222222222222222222222222212122222222222222222222222222222221111212222222222222122121221211112222222222222222222222222212221222222222222222222222222222222222222

>1PHKA

TRDAALPGSHSTHGFYENYEPKEILGRGVSSVVRRCIHKPTCKEYAVKIIDVTGGGSFSAEEVQELREATLKEVDILRKVSGHPNIIQLKDTYETNTFFFLVFDLMKKGELFDYLTEKVTLSEKETRKIMRALLEVICALHKLNIVHRDLKPENILLDDDMNIKLTDFGFSCQLDPGEKLREVCGTPSYLAPEIIECSMNDNHPGYGKEVDMWSTGVIMYTLLAGSPPFWHRKQMLMLRMIMSGNYQFGSPEWDDYSDTVKDLVSRFLVVQPQKRYTAEEALAHPFFQQYVVEEVRHF

2222222222222222222222221121121212222222222221212222222222222222222222222222222222222212222222222222221111222122222222222222222222222222222222222222221211212222222222122222222222222222222222222222222222222222222222222222222222222222222222222222222222222222222222222222222222222222222222222222222222

>2W02A

MNNRNHDVLSRMISEKAALHGLLNCLIKEFAIPEGYLRYEWPDEMKGIPPGAYFDGADWKGIPMMIGLPDQLQLFVMVDRRDTFGSQHYLSDVYLRQAQGDWQCPDFEPLVARLLAACEHIAGRKNPELYEQILQSQRLVSAIVSHNGRQRADAPLQHYLQSEQGLWFGHPSHPAPKARLWPAHLGQEQWAPEFQARAALHQFEVPVDGLHIGANGLTPQQVLDGFADQQPASPGHAIICMHPVQAQLFMQDARVQQLLRDNVIRDLGQSGRVASPTASIRTWFIDDHDYFIKGSLNVRITNCVRKNAWYELESTVLIDRLFRQLLDQHADTLGGLVAAAEPGVVSWSPAAAGELDSHWFREQTGGILRENFCRRTGAERSIMAGTLFARGVDLQPMIQTFLRTHYGEALDDNALLYWFDDYQTRLLRPVLSLFFNHGVVMEPHLQNSVLVHQQGRPQQVLLRDFEGVKLTDDLGIRYIDDDIHPRVRQSLLYSREQGWNRIMYCLFINHLSETILALSQGRPQLAPLMWRRVQQQLRAIQGELKQPSPELDALIAGHPVACKTNLKVRLAAEADRQASYVRLPSPWGHAVQHGSEVQHDERRHGDVRHEEARHGEVQHG

22222222222222222222222222222222222222222222222222222222222222222222222222222222222222222222222222222222222222222222222222222222222222222222222222222222222222222222212221122222222222222222222222222222222222222222222222222222222222222222222222222222222222222222222222222222222222121122222222221222222212221222222222222222222222222222222222222222222222222222222222222222122222222222222222222222222222222222222222222222222222222222222222222222222111122222222222222221212222222222222222222222222222222222222222221222222222222222222222222222222222222222222222222222222222222222222222222222222222222222222222222222222222222222

>2W5GA

KETAAAKFERQHMDSSTSAASSSNYCNQMMKSRNLTKDRCKPVNTFVHESLADVQAVCSQKNVACKNGQTNCYQSYSTMSITDCRETGSSKYPNCAYKTTQANKHIIVACEGNPYVPVHFDASV

2222221222112222222222222222222222222222122222222222222222222222121212122222222222222222222222222222222222221212222221112222

>1KJ8A

TLLGTALRPAATRVMLLGSGELGKEVAIECQRLGVEVIAVDRYADAPAMHVAHRSHVINMLDGDALRRVVELEKPHYIVPEIEAIATDMLIQLEEEGLNVVPCARATKLTMNREGIRRLAAEELQLPTSTYRFADSESLFREAVADIGYPCIVKPVMSSSGKGQTFIRSAEQLAQAWKYAQQGGRAGAGRVIVEGVVKFDFEITLLTVSAVDGVHFCAPVGHRQEDGDYRESWQPQQMSPLALERAQEIARKVVLALGGYGLFGVELFVCGDEVIFSEVSPRPHDTGMVTLISQDLSEFALHVRAFLGLPVGGIRQYGPAASAVILPQLTSQNVTFDNVQNAVGADLQIRLFGKPEIDGSRRLGVALATAESVVDAIERAKHAAGQVKVQG

2222222222222222222222222222222222222222222222222222222222222222222222222222222222222222222222222222222222222222122222222222222212222222222222222222222121222111122122222222222222222222222222222111121221222222222222222222222122122222222222222222222222222222222222222121222222222122222222222222222222222222222222222222222222222222222222222222222222222222222222222222222222222222222222222222222

>1LHRA

MEEECRVLSIQSHVVRGYVGNRAATFPLQVLGFEVDAVNSVQFSNHTGYSHWKGQVLNSDELQELYDGLKLNHVNQYDYVLTGYTRDKSFLAMVVDIVQELKQQNPRLVYVCDPVMGDQRNGEGAMYVPDDLLPVYREKVVPVADIITPNQFEAELLTGRKIHSQEEALEVMDMLHSMGPDTVVITSSNLLSPRGSDYLMALGSQRTRAPDGSVVTQRIRMEMHKVDAVFVGTGDLFAAMLLAWTHKHPNNLKVACEKTVSAMHHVLQRTIKCAKAKSGEGVKPSPAQLELRMVQSKKDIESPEIVVQATVL

222222222222222222222222222222222222222222222222222222222222222222222222222222222222222222222222222222222222222222222212222222222222222222222222222221222222222222222222222222222222222221122222222222222222222222222222222222111121222211221222222222222222222222222222221222222222222222222222222222222222222222222222

>2PBZA

USLIVSTIASHSSLQILLGAKKEGFKTRLYVSPKRRPFYSSLPIVDDLVVAEEUTSILNDDGIVVPHGSFVAYLGIEAIEKAKARFFGNRRFLKWETTFELQDKALEGAGIPRVEVVEPEDAKPDELYFVRIEGPRGGSGHFIVEGSELEERLSTLEEPYRVERFIPGVYLYVHFFYSPILERLELLGVDERVLIADGNARWPVKPLPYTIVGNRAIALRESLLPQLYDYGLAFVRTURELEPPGVIGPFALHFAYDGSFKAIGIASRIDGGSNADHWYSELYWGERLSUGRRIARELRLAEEEDRLEEVVTEGHHHHHH

22222222222222222222222222222222222222222222222222222222222222222222222222222222222222222222222222222222222222222222222222222222122212222222222212222222222222222211112221212222222222222222222221222222122222221222222222222222222222222222222222222222222222222222221222222222222222222222222222222222222222222222222222222222

>1ZFNA

GSMNDRDFMRYSRQILLDDIALDGQQKLLDSQVLIIGLGGLGTPAALYLAGAGVGTLVLADDDDVHLSNLQRQILFTTEDIDRPKSQVSQQRLTQLNPDIQLTALQQRLTGEALKDAVARADVVLDCTDNMATRQEINAACVALNTPLITASAVGFGGQLMVLTPPWEQGCYRCLWPDNQEPERNCRTAGVVGPVVGVMGTLQALEAIKLLSGIETPAGELRLFDGKSSQWRSLALRRASGCPVCGGSNADPV

2222222222221222222222222222222222211111222222222222222222211212222212211222222222221222222222222222222222111222222222222222221112221222222222222222222222222222222222222222222222222222222222222222222222222222222222222222222222222222222222222222222222222

>1QHGA

MNFLSEQLLAHLNKEQQEAVRTTEGPLLIMAGAGSGKTRVLTHRIAYLMAEKHVAPWNILAITFTNKAAREMRERVQSLLGGAAEDVWISTFHSMCVRILRRDIDRIGINRNFSILDPTDQLSVMKTILKEKNIDPKKFEPRTILGTISAAKNELLPPEQFAKRASTYYEKVVSDVYQEYQQRLLRNHSLDFDDLIMTTIQLFDRVPDVLHYYQYKFQYIHIDEYQDTNRAQYTLVKKLAERFQNICAVGDADQSIYRWRGADIQNILSFERDYPNAKVILLEQNYRSTKRILQAANEVIEHNVNRKPKRIWTENPEGKPILYYEAMNEADEAQFVAGRIREAVERGERRYRDFAVLYRTNAQSRVMEEMLLKANIPYQIVGGLKFYDRKEIKDILAYLRVIANPDDDLSLLRIINVPKRGIGASTIDKLVRYAADHELSLFEALGELEMIGLGAKAAGALAAFRSQLEQWTQLQEYVSVTELVEEVLDKSGYREMLKAERTIEAQSRLENLDEFLSVTKHFENVSDDKSLIAFLTDLALISDLDELDGTEQAAEGDAVMLMTLHAAKGLEFPVVFLIGMEEGIFPHNRSLEDDDEMEEERRLAYVGITRAEEELVLTSAQMRTLFGNIQMDPPSRFLNEIPAHLLETASRRQAGASRPAVSRPQASGAVGSWKVGDRANHRKWGIGTVVSVRGGGDDQELDIAFPSPIGIKRLLAKFAPIEKV

2222222222122221222222222222222221111212222222222222222222222222222222222222222222222222222222222222222222222222222222222222222222222222222222222222222222222222222222222222222222222222222222222222222222222222222222222222222222222222222222222222222222222222222222222222222222222222222221122222222222222222222222222222222222222222222222222222222222222222222222222222222222222222222222222222222222222222222222222222222222222222222222222222222222222222222222222222222222222222222222222222222222222222222222222222222222222222222222222222222222222222222222222222222222222222122222222222222222222222222222222222222222222222222222222222222222222222222222222222222222222222222222222222222222222222222222222222222222222222222222222222

>2Z1UA

GAMSGETLLLDYGSGGRASHRLISDLFLRHFDNPILGTLNDAARLDLTGPLAMSTDSYTVDPIFFPGGDIGTLAVHGTVNDVSMLGARPRYLSCGFILEEGLDMDILERVVASMGKAAREAGVFIVTGDTKVVPRGACDKMFINTTGIGEILVDPAPSGDRARPGDAILISGSMGDHGLTILSQRQGLNFAADVCSDSASLNRVVEKLVLEVGDIHVLRDPTRGGLATTLNEIAGQSQAVCHVLETAVPVRESVRNGCSFLGLDPLYLANEGKLICILPEERAEAALAVLREGPHGEHAARIGSVKSVGELGAARAGQVVMETALGGHRLLSMLEGEQLPRIC

2222222222222222222222222222222222222222222222222222222122222222222222222222222212222222222222222222222222222222222222222222222222222222222222222222222222222222222222222222222222222222222222222222222222222222222222222221211222222222222222222222222222222222222222222222222222222222222222222222222222222222222222222222222222222222222222222222222

>3GBUA

USLIASIGELLIDLISVEEGDLKDVRLFEKHPGGAPANVAVGVSRLGVKSSLISKVGNDPFGEYLIEELSKENVDTRGIVKDEKKHTGIVFVQLKGASPSFLLYDDVAYFNUTLNDINWDIVEEAKIVNFGSVILARNPSRETVUKVIKKIKGSSLIAFDVNLRLDLWRGQEEEUIKVLEESIKLADIVKASEEEVLYLENQGVEVKGSULTAITLGPKGFRLIKNETVVDVPSYNVNPLDTTGAGDAFUAALLVGILKLKGLDLLKLGKFANLVAALSTQKRGAWSTPRKDELLKYKEAREVLAEGHHHHHH

2222222222222222222222222222222222222222222222222222222222222222222222222222222222222222222222222222222222222222222222222222222222222222222222222222222222222222212122222222222222222222222221222222222222222222222222121121122222222222211212122122112222222222222222222222222212212222222222222222222222222222222222222

>1QHXA

MTTRMIILNGGSSAGKSGIVRCLQSVLPEPWLAFGVDSLIEAMPLKMQSAEGGIEFDADGGVSIGPEFRALEGAWAEGVVAMARAGARIIIDDVFLGGAAAQERWRSFVGDLDVLWVGVRCDGAVAEGRETARGDRVAGMAAKQAYVVHEGVEYDVEVDTTHKESIECAWAIAAHVVP

2222222222111111112222222222222222221222222222222222222222222222222222222222222222222222222222222222222222222222222222222222222212221221222222222222222222222221221112222222222222

>3BG5A

MGSSHHHHHHSSGLVPRGSHMASMKQIKKLLVANRGEIAIRIFRAAAELDISTVAIYSNEDKSSLHRYKADESYLVGSDLGPAESYLNIERIIDVAKQANVDAIHPGYGFLSENEQFARRCAEEGIKFIGPHLEHLDMFGDKVKARTTAIKADLPVIPGTDGPIKSYELAKEFAEEAGFPLMIKATSGGGGKGMRIVREESELEDAFHRAKSEAEKSFGNSEVYIERYIDNPKHIEVQVIGDEHGNIVHLFERDCSVQRRHQKVVEVAPSVGLSPTLRQRICDAAIQLMENIKYVNAGTVEFLVSGDEFFFIEVNPRVQVEHTITEMVTGIDIVKTQILVAAGADLFGEEINMPQQKDITTLGYAIQCRITTEDPLNDFMPDTGTIIAYRSSGGFGVRLDAGDGFQGAEISPYYDSLLVKLSTHAISFKQAEEKMVRSLREMRIRGVKTNIPFLINVMKNKKFTSGDYTTKFIEETPELFDIQPSLDRGTKTLEYIGNVTINGFPNVEKRPKPDYELASIPTVSSSKIASFSGTKQLLDEVGPKGVAEWVKKQDDVLLTDTTFRDAHQSLLATRVRTKDMINIASKTADVFKDGFSLEMWGGATFDVAYNFLKENPWERLERLRKAIPNVLFQMLLRASNAVGYKNYPDNVIHKFVQESAKAGIDVFRIFDSLNWVDQMKVANEAVQEAGKISEGTICYTGDILNPERSNIYTLEYYVKLAKELEREGFHILAIKDMAGLLKPKAAYELIGELKSAVDLPIHLHTHDTSGNGLLTYKQAIDAGVDIIDTAVASMSGLTSQPSANSLYYALNGFPRHLRTDIEGMESLSHYWSTVRTYYSDFESDIKSPNTEIYQHEMPGGQYSNLSQQAKSLGLGERFDEVKDMYRRVNFLFGDIVKVTPSSKVVGDMALYMVQNDLDEQSVITDGYKLDFPESVVSFFKGEIGQPVNGFNKDLQAVILKGQEALTARPGEYLEPVDFEKVRELLEEEQQGPVTEQDIISYVLYPKVYEQYIQTRNQYGNLSLLDTPTFFFGMRNGETVEIEIDKGKRLIIKLETISEPDENGNRTIYYAMNGQARRIYIKDENVHTNANVKPKADKSNPSHIGAQMPGSVTEVKVSVGETVKANQPLLITEAMKMETTIQAPFDGVIKQVTVNNGDTIATGDLLIEIEKATD

222222222222222222222222222222222222222222222222222222222222222222222222222222222222222222222222222222222222222222222222222222222222222222222122222222222222222222222222222222222222212122222222222222222222222222222222222222222111122221222222222222222222222221221212222222222222222222222222222222222222121222222222121222222222222222222222222222222222222222222222222222222222222222222222222222222222222222222222222222222222222222222222222222222222222222222222222222222222222222222222222222222222222222222222222222222222222222222222222222222222222222222222222222222222222222222222222222222222222222222222222222222222222222222222222222222222222222222222222222222222222222222222222222222222222222222222222222222222222222222222222222222222222222222222222222222222222222222222222222222222222222222222222222222222222222222222222222222222222222222222222222222222222222222222222222222222222222222222222222222222222222222222222222222222222222222222222222222222222222222222222222222222222222222222222222222222222222222222222222222222222222222222222222222222222222222222222222222222222222222222222222222222222222222222222222222222222222222222222222222222222222222222222222222222222222222

>3K5HA

MGSSHHHHHHSSENLYFQGHMWNSRKVGVLGGGQLGRMLVESANRLNIQVNVLDADNSPAKQISAHDGHVTGSFKEREAVRQLAKTCDVVTAEIEHVDTYALEEVASEVKIEPSWQAIRTIQNKFNQKEHLRKYGIPMAEHRELVENTPAELAKVGEQLGYPLMLKSKTMAYDGRGNFRVNSQDDIPEALEALKDRPLYAEKWAYFKMELAVIVVKTKDEVLSYPTVETVQEDSICKLVYAPARNVSDAINQKAQELARKAVAAFDGKGVFGVEMFLLEDDSIMLCEIASRIHNSGHYTIEGCALSQFDAHLRAILDLPIPAQSLEIRQPSIMLNIIGGAAPDTHLQAAECALSIPNASIHLYSKGAAKPGRKMGHITVTAPTMHEAETHIQPLIDVVDRIRA

2222222222222222222222222222222222222222222222222222222222222222222222222222222222222222222222222222222222222222222222222221222222222222222222222222222222222222222121222211112212222222222222222222222211112122122222222222222222222212212222222222222222222222222222222222222221212222222221122222222222222222222222222222222222222222222222222222222222222222222222222222222222222222222222222222222222222222222

>2CJAA

UGSSHHHHHHSSGLVPRGSHUKLQFNLKAYFKTSADPTPAKDAIAALFEEANSTLLTRGAPEGQGAKVTEWKLGEDRIELTLQSGRYVRVHDAIFRLRKQLAEALGKKYKIGIRGIEVESFIIKVPADHELRULKVPYIKSUENIEGGIQLELEVGEAEUKNRVPDRILTLLEEKIEAAQYGAKAEHWNLLWQREPUEHPFKEDPTQAUUKEGWLKRGSSRGQWIHGPQSARIFRTFEKIVLEELLEPLGYREUIFPKLVTWEVWUKSGHAKGVYPEIYYVCPPQTRDPDYWEEVADYYKVTHEVPTKLIKEKIAEPIGGUCYAQCPPFWUYVAGETLPNEEIPVKVFDRSGTSHRYESGGIHGIERVDEFHRIEIVWIGTKEEVLKCAEELHDRYUHIFNDILDIEWRKARVTPWFUAQEGLLGLAEENTVGTTDYEACLPYRGPDGEWLEFQNVSINGDKYPKGFNVKLQSGDELWSGCSGVGLERWAAVFLAQKGLDPANWPEEFRNRVGEUPKGIRFL

222222222222222222222222222222222222222222222222222222222222222222222222222222222222222222222222222222222222222222222222222222222222222222222222222222222222222222222222222222222222222222222222222222222222222222222222222222222222222222222222222222222222222222222222222221222222222222222222222222222222222222222222222222222211222222222222222222222222222222212122222222212212122222222222222222222222222222222222222222222222222222222222222222222222222222212212222222222222222222222222222212212222222222222222222222222222222222

>1KVKA

MLSEVLLVSAPGKVILHGEHAVVHGKVALAVALNLRTFLVLRPQSNGKVSLNLPNVGIKQVWDVATLQLLDTGFLEQGDVPAPTLEQLEKLKKVAGLPRDCVGNEGLSLLAFLYLYLAICRKQRTLPSLDIMVWSELPPGAGLGSSAAYSVCVAAALLTACEEVTNPLKDRGSIGSWPEEDLKSINKWAYEGERVIHGNPSGVDNSVSTWGGALRYQQGKMSSLKRLPALQILLTNTKVPRSTKALVAGVRSRLIKFPEIMAPLLTSIDAISLECERVLGEMAAAPVPEQYLVLEELMDMNQHHLNALGVGHASLDQLCQVTAAHGLHSKLTGAGGGGCGITLLKPGLERAKVEAAKQALTGCGFDCWETSIGAPGVSMHSATSIEDPVRQALGL

22222222222212222222222222222222222222222222222222221212222222222222222222222222222222222222222222222221122122222222222222222222222222122221211111222222222222222222222222222222222222222222222222211222222122222222222222222222222222222222222222222222222222222222222222222222222222222222222222222222222222222222222222222222222222222222222222222222222222222222222222222222222222222222222222222222222

>1A0IA

VNIKTNPFKAVSFVESAIKKALDNAGYLIAEIKYDGVRGNICVDNTANSYWLSRVSKTIPALEHLNGFDVRWKRLLNDDRCFYKDGFMLDGELMVKGVDFNTGSGLLRTKWTDTKNQEFHEELFVEPIRKKDKVPFKLHTGHLHIKLYAILPLHIVESGEDCDVMTLLMQEHVKNMLPLLQEYFPEIEWQAAESYEVYDMVELQQLYEQKRAEGHEGLIVKDPMCIYKRGKKSGWWKMKPENEADGIIQGLVWGTKGLANEGKVIGFEVLLESGRLVNATNISRALMDEFTETVKEATLSQWGFFSPYGIGDNDACTINPYDGWACQISYMEETPDGSLRHPSFVMFR

222222222222222222222222222222111122212222222222222221222222222222222222222222222222222222212222222222222222222222222222222222222222222222222222222122222222222222222222222222222222222222222222222222222222222222222222221212222222221222121222122222222222222222222222222222222222222222222222222222222222222222222222222222222222222222222222222222222222

>3C5EA

MGHHHHHHSSGVDLGTENLYFQSMSLQWGHQEVPAKFNFASDVLDHWADMEKAGKRPPSPALWWVNGKGKELMWNFRELSENSQQAANVLSGACGLQRGDRVAVVLPRVPEWWLVILGCIRAGLIFMPGTIQMKSTDILYRLQMSKAKAIVAGDEVIQEVDTVASECPSLRIKLLVSEKSCDGWLNFKKLLNEASTTHHCVETGSQEASAIYFTSGTSGLPKMAEHSYSSLGLKAKMDAGWTGLQASDIMWTISDTGWILNILCSLMEPWALGACTFVHLLPKFDPLVILKTLSSYPIKSMMGAPIVYRMLLQQDLSSYKFPHLQNCVTVGESLLPETLENWRAQTGLDIRESYGQTETGLTCMVSKTMKIKPGYMGTAASCYDVQIIDDKGNVLPPGTEGDIGIRVKPIRPIGIFSGYVDNPDKTAANIRGDFWLLGDRGIKDEDGYFQFMGRADDIINSSGYRIGPSEVENALMEHPAVVETAVISSPDPVRGEVVKAFVVLASQFLSHDPEQLTKELQQHVKSVTAPYKYPRKIEFVLNLPKTVTGKIQRAKLRDKEWKMSGKARAQ

222222222222222222222222222222222222222222222222222222222222222222222222222222222222222222222222222222222222222222222222222222222222222222222222222222222222222222222222222222222222222222222222222222222222222222222111112221222222222222222222222222222222222222222222222222222222222222222222222222222222222222222222222222222222222222111222222222222222222111111222222222222222222222222222222222222222222222222222222222222222222222222222222222122222222222122122222222222222222222222222222222222222222222222222222222222222222222222222222222222222222222222122222222222222222222

>1MO8A

QNPMTVAHMWFDNQIHEADTTENQSGVSFDKTSATWFALSRIAGLCNRAVFQANQENLPILKRAVAGDASESALLKCIEVCCGSVMEMREKYTKIVEIPFNSTNKYQLSIHKNPNASEPKHLLVMKGAPERILDRCSSILLHGKEQPLDEELKDAFQNAYLELGGLGERVLGFCHLLLPDEQFPEGFQFDTDEVNFPVDNLCFVGLISMIDPP

222222222222222222222222222222222222222222222221222222222222222222222122222222222222222222222222222122221222222222222222222221112222222222222222222222222222222222222221222222222222222222222222222222222222222222222

>2E5YA

MKTIHVSVVTPDGPVYEDDVEMVSVKAKSGELGILPGHIPLVAPLEISAARLKKGGKTQYIAVSGGFLEVRPDKVTILAQAAERAEDIDVLRAKAAKERAERRLQSQQDDIDFKRAELALKRAMNRLSVAEMK

2222222222212222222222222222222222222222222222222222222222222222222222222222222212122211122112222212222222222222221222222122212222222

>1D9ZA

VEGRFQLVAPYEPQGDQPQAIAKLVDGLRRGVKHQTLLGATGTGKTFTISNVIAQVNKPTLVIAHNKTLAGQLYSELKEFFPHNAVEYFVSYYDYYQPEAYVPQTDTYIEKDAKINDEIDKLRHSATSALFERRDVIIVASVSCIYGLGSPEEYRELVVSLRVGMEIERNALLRRLVDIQYDRNDIDFRGTFRVRGDVVEIFPASRDEHCIRVEFFGDEIERIREVDALTGKVLGEREHVAIFPASHFVTREEKMRLAIQNIEQELEERLAELRAQGKLLEAQRLEQRTRYDLEMMREMGFCSGIENYSRHLALRPPGSTPYTLLDYFPDDFLIIVDESHVTLPQLRGMYNGDRARKQVLVDHGFRLPSALDNRPLTFEEFEQKINQIIYVSATPGPYELEHSPGVVEQIIRPTGLLDPTIDVRPTKGQIDDLIGEIRERVERNERTLVTTLTKKMAEDLTDYLKEAGIKVAYLHSEIKTLERIEIIRDLRLGKYDVLVGINLLREGLDIPEVSLVAILDADKEGFLRSERSLIQTIGRAARNANGHVIMYADTITKSMEIAIQETKRRRAIQEEYNRKHGIVPRTVKKEIRDVIRATYAAEETEMYEAKPAAAMTKQEREELIRTLEAEMKEAAKALDFERAAQLRDIIFELKAEG

222222222211212212222222222222222222222111211122222222222222222222222222222222222222222222222222222222222222222222222222222222222222222222222222222222222222222222222222222222222222222222222222222222222222222222222222222222222222222222222222222222222222222222222222222222222222222222222222222222222222222222222222222222222222222222222222222222222222222222222222222222222222222222222222222222222222222222222222222222222222222222222222222222222222222222222222222222222222222222222222222222222222222222222222222212222222222222222222222222222222222222222222222222222222222222222222222222222222222222222222222222222222222222222222222222222222222222222222222222222

>1XDNA

GHMDQSDFSPYIEIDLPSESRIQSLHKSGLAAQEWVACEKVHGTNFGIYLINQGDHEVVRFAKRSGIUDPNENFFGYHILIDEFTAQIRILNDLLKQKYGLSRVGRLVLNGELFGAKYKHPLVPKSEKWCTLPNGKKFPIAGVQIQREPFPQYSPELHFFAFDIKYSVSGAEEDFVLLGYDEFVEFSSKVPNLLYARALVRGTLDECLAFDVENFUTPLPALLGLGNYPLEGNLAEGVVIRHVRRGDPAVEKHNVSTIIKLRCSSFUELKHPGKQKE

2222222222111122222222222222222222222211122212222222222222222221222222222222222222222222222222222222222222222221222222222222222222222222222222222222222222222221212222222222222222222222222222222222222222222222222222222222222222222222222222122222222222222222222121222222222222222

>3HAVA

MVNLDAEIYEHLNKQIKINELRYLSSGDDSDTFLCNEQYVVKVPKRDSVRISQKREFELYRFLENCKLSYQIPAVVYQSDRFNIMKYIKGERITYEQYHKLSEKEKDALAYDEATFLKELHSIEIDCSVSLFSDALVNKKDKFLQDKKLLISILEKEQLLTDEMLEHIETIYENILNNAVLFKYTPCLVHNDFSANNMIFRNNRLFGVIDFGDFNVGDPDNDFLCLLDCSTDDFGKEFGRKVLKYYQHKAPEVAERKAELNDVYWSIDQIIYGYERKDREMLIKGVSELLQTQAEMFIF

22222222222222222222222112222121222222212122222222222222222222222222222222222222222211112222222222222222222222222222222222222222222222222222222222222222222222222222222222222222222222222222222222212222222222221122222222222222222222222222222222222222222222222222222222222222222222222222222222222222222

>1Z7EA

MKTVVFAYHDMGCLGIEALLAAGYEISAIFTHTDNPGEKAFYGSVARLAAERGIPVYAPDNVNHPLWVERIAQLSPDVIFSFYYRHLIYDEILQLAPAGAFNLHGSLLPKYRGRAPLNWVLVNGETETGVTLHRMVKRADAGAIVAQLRIAIAPDDIAITLHHKLCHAARQLLEQTLPAIKHGNILEIAQRENEATCFGRRTPDDSFLEWHKPASVLHNMVRAVADPWPGAFSYVGNQKFTVWSSRVHPHASKAQPGSVISVAPLLIACGDGALEIVTGQAGDGITMQGSQLAQTLGLVQGSRLNSQPACTARRRTRVLILGVNGFIGNHLTERLLREDHYEVYGLDIGSDAISRFLNHPHFHFVEGDISIHSEWIEYHVKKCDVVLPLVAIATPIEYTRNPLRVFELDFEENLRIIRYCVKYRKRIIFPSTSEVYGMCSDKYFDEDHSNLIVGPVNKPRWIYSVSKQLLDRVIWAYGEKEGLQFTLFRPFNWMGPRLDNLNAARIGSSRAITQLILNLVEGSPIKLIDGGKQKRCFTDIRDGIEALYRIIENAGNRCDGEIINIGNPENEASIEELGEMLLASFEKHPLRHHFPPFAGFRVVESSSYYGKGYQDVEHRKPSIRNAHRCLDWEPKIDMQETIDETLDFFLRTVDLTDKPS

222222222222222222222222222222222222222222222222222222222222222222222222222222222222222222222222222222222222222222222222222222222222222222222222222222222222222222222222222222222222222222222222222222222222222222222222222222222222222222222222222222222222222222222222222222222222222222222222222222222222222222222222222222222121111222222222222222222211222222222222222222111222222222222222222211221222222222222222222222222222222222222222222222222222222222222222222222222222222222222222222222222222222222222222222221222222222222222222222222222222222222222222222222222222222222222222222222222222222222222222222222222222222222222222222222222222222222222222222222222222

>2OH5A

UADVAGTSNRDFRGREQRLFNSEQYNYNNSLNGEVSVWVYAYYSDGSVLVINKNSQYKVGISETFKALKEYRKGQHNDSYDEYEVNQSIYYPNGGDARKFHSNAKPRAIQIIFSPSVNVRTIKMAKGNAVSVPDEYLQRSHPWEATGIKYRKIKRDGEIVGYSHYFELPHEYNSISLAVSGVHKNPSSYNVGSAHNVMDVFQSCDLALRFCNRYWAELELVNHYISPNAYPYLDINNHSYGVALSNRQ

22222222222222222222222212222222222222222222222222222222222222222222222222222222222222222222222222222222222222222222222222222222222222222222222222222221212212122122222222222222222222212222222222222222222222222222222222222222222222222222222222222222

>1J09A

MVVTRIAPSPTGDPHVGTAYIALFNYAWARRNGGRFIVRIEDTDRARYVPGAEERILAALKWLGLSYDEGPDVGGPHGPYRQSERLPLYQKYAEELLKRGWAYRAFETPEELEQIRKEKGGYDGRARNIPPEEAEERARRGEPHVIRLKVPRPGTTEVKDELRGVVVYDNQEIPDVVLLKSDGYPTYHLANVVDDHLMGVTDVIRAEEWLVSTPIHVLLYRAFGWEAPRFYHMPLLRNPDKTKISKRKSHTSLDWYKAEGFLPEALRNYLCLMGFSMPDGREIFTLEEFIQAFTWERVSLGGPVFDLEKLRWMNGKYIREVLSLEEVAERVKPFLREAGLSWESEAYLRRAVELMRPRFDTLKEFPEKARYLFTEDYPVSEKAQRKLEEGLPLLKELYPRLRAQEEWTEAALEALLRGFAAEKGVKLGQVAQPLRAALTGSLETPGLFEILALLGKERALRRLERALA

222222222222221212222222222222222222222222222222222222222222222222222222222222222222222222222222222222222222222222222222222222222222222222222222222222222222222222222222222222222222222222222222222222222222212112222222222222222222222222112222221111122222222222222222222222222222222222222222222222222222222222222222222222222222222222222222222222222222222222222222222222222222222222222222222222222222222222222222222222222222222222222222222222222222222222222222222222222222

>2W00A

UTHQTHTIAESNNFIVLDKYIKAEPTGDSYQSESDLERELIQDLRNQGYEFISVKSQSAULANVREQLQNLNGVVFNDSEWRRFTEQYLDNPSDGILDKTRKIHIDYICDFIFDDERLENIYLIDKKNLURNKVQIIQQFEQAGSHANRYDVTILVNGLPLVQIELKKRGVAIREAFNQIHRYSKESFNSENSLFKYLQLFVISNGTDTRYFANTTKRDKNSFDFTUNWAKSDNTLIKDLKDFTATCFQKHTLLNVLVNYSVFDSSQTLLVURPYQIAATERILWKIKSSFTAKNWSKPESGGYIWHTTGSGKTLTSFKAARLATELDFIDKVFFVVDRKDLDYQTUKEYQRFSPDSVNGSENTAGLKRNLDKDDNKIIVTTIQKLNNLUKAESDLPVYNQQVVFIFDECHRSQFGEAQKNLKKKFKRYYQFGFTGTPIFPENALGSETTASVFGRELHSYVITDAIRDEKVLKFKVDYNDVRPQFKSLETETDEKKLSAAENQQAFLHPURIQEITQYILNNFRQKTHRTFPGSKGFNAULAVSSVDAAKAYYATFKRLQEEAANKSATYKPLRIATIFSFAANEEQNAIGEISDETFDTSAUDSSAKEFLDAAIREYNSHFKTNFSTDSNGFQNYYRDLAQRVKNQDIDLLIVVGUFLTGFDAPTLNTLFVDKNLRYHGLUQAFSRTNRIYDATKTFGNIVTFRDLERSTIDAITLFGDKNTKNVVLEKSYTEYUEGFTDAATGEAKRGFUTVVSELEQRFPDPTSIESEKEKKDFVKLFGEYLRAENILQNYDEFATLKALQQIDLSDPVAVEKFKAEHYVDDEKFAELQTIRLPADRKIQDYRSAYNDIRDWQRREKEAEKKEKSTTDWDDVVFEVDLLKSQEINLDYILGLIFEHNRQNKGKGEUIEEVKRLIRSSLGNRAKEGLVVDFIQQTNLDDLPDKASIIDAFFTFAQREQQREAEALIKEENLNEDAAKRYIRTSLKREYATENGTELNETLPKLSPLNPQYKTKKQAVFQKIVSFIEKFKGVGGKI

222222222222222222222222222222222222222222222222222222222222222222222222222222222222222222222222222222222222222222222222222222222222222222222222222222222222222222222222222222222222222222222222222222222222222222222222222122222222222222222222222222222222222222222222222221121221222222222222222222222222222222221111111222222222222222222222222222222222222222222222222222222222222222222222222222222222222222222222222222222222222222222222222222222222222222222222222222222222222222222222222222222222222222222222222222222222222222222222222222222222222222222222222222222222222222222222222222222222222222222222222222222222222222222222222222222222222222222222222222222222112122222222222222222222222122122222222222222222222222222222222222222222222222222222222222222222222222222222222222222222222222222222222222222222222222222222222222222222222222222222222222222222222222222222222222222222222222222222222222222222222222222222222222222222222222222222222222222222222222222222222222222222222222222222222222222222222222222222222222222222222222222222222222

>3HMNA

HHHHHHSSGLVPRGSGMKETAAAKFERQHMDSPDLGTDDDDKASSSANECISVKGRIYSILKQIGSGGSSKVFQVLNEKKQIYAIKYVNLEEADNQTLDSYRNEIAYLNKLQQHSDKIIRLYDYEITDQYIYMVMECGNIDLNSWLKKKKSIDPWERKSYWKNMLEAVHTIHQHGIVHSDLKPANFLIVDGMLKLIDFGIANQMQPDTTSVVKDSQVGTVNYMPPEAIKDMSSSRENGKSKSKISPKSDVWSLGCILYYMTYGKTPFQQIINQISKLHAIIDPNHEIEFPDIPEKDLQDVLKCCLKRDPKQRISIPELLAHPYVQIQTHPVNQMAKGTTEEM

222222222222222222222222222222222222222222222222222222222222222111122121222222222221222222222222222222222222222222222222222222222222221121221222222222222222222222222222222222222222222222122222222222222221222222222222222222222222222222222222222222222222222222222222222222222222222222222222222222222222222222222222222222222222222222222222222222

>2E89A

MNPESRVIRKVLALQNDEKIFSGERRVLIAFSGGVDSVVLTDVLLKLKNYFSLKEVALAHFNHMLRESAERDEEFCKEFAKERNMKIFVGKEDVRAFAKENRMSLEEAGRFLRYKFLKEILESEGFDCIATAHHLNDLLETSLLFFTRGTGLDGLIGFLPKEEVIRRPLYYVKRSEIEEYAKFKGLRWVEDETNYEVSIPRNRIRHRVIPELKRINENLEDTFLKMVKVLRAEREFLEEEAQKLYKEVKKGNCLDVKKLKEKPLALQRRVIRKFIGEKDYEKVELVRSLLEKGGEVNLGKGKVLKRKERWLCFSPEV

22222222222222222222222222222111222222222222222222222222221112222222222222222222222222222222222222222222222221221222222222222222221112222222222222222222222222222222222222222222222222222222222222222222122222222222222222222222222222222222222222222222222222222222222222222222222222222222222222222222222222222222222222222

>2NPIA

UGSSHHHHHHSQDPNSASLPGIDEHTTSEELITGDNEWHKLVIPKGSDWQIDLKAEGKLIVKVNSGIVEIFGTELAVDDEYTFQNWKFPIYAVEETELLWKCPDLTTNTITVKPNHTUKYIYNLHFULEKIRUSNFEGPRVVIVGGSQTGKTSLSRTLCSYALKFNAYQPLYINLDPQQPIFTVPGCISATPISDILDAQLPTWGQSLTSGATLLHNKQPUVKNFGLERINENKDLYLECISQLGQVVGQRLHLDPQVRRSGCIVDTPSISQLDENLAELHHIIEKLNVNIULVLCSETDPLWEKVKKTFGPELGNNNIFFIPKLDGVSAVDDVYKRSLQRTSIREYFYGSLDTALSPYAIGVDYEDLTIWKPSNVFDNEVGRVELFPVTITPSNLQHAIIAITFAERRADQATVIKSPILGFALITEVNEKRRKLRVLLPVPGRLPSKAUILTSYRYLE

2222222222222222222222222222222222222222222222212122222222222222222222122222222222222212122222222222222222222222221222222222222222222222222222222211111112222222222222222222222222222222222222222222222222222222222222222222222222222222222222222222222222222222222222222222222222222222222222222222222222222222222222222222222222222111121222122222222222222222222222222222222222222222222222222222222222222222222222222222222222222222222222222222222222222222222222222222

>3IKHA

USLRVYVTGNITVDETWSIPDIPKKGASIHGVKVSQDIGGKGANQAIILSRCGIETRLIAATGNDSNGAWIRQQIKNEPLULLPDGHFNQHSDTSIILNSADGDNAIITTTAAADTFSLDEUIPHUADAVAGDILLQQGNFSLDKTRALFQYARSRGUTTVFNPSPVNPDFCHLWPLIDIAVVNESEAELLQPYGVKTLVITQGAAGAWLVQEGQRQFCPAVPAEALDTTGAGDTFLAVULASALLRGVAPDALALAHASRAAAITVSRRGTLSAFPGSRELAALLTTDGAEGHHHHHH

22222222222222222222222222222222222222222222222222222222222222222222222222222222222222222222222222222222222222222222222222222222222222222222222222222222222222222222222222222222222222212222222222222222211112122222222222221122212222211221222222222222222222222221221222122222222222222222222222222222222

>1SU2A

MEHDERTHVPVELRAAGVVLLNERGDILLVQEKGIPGHPEKAGLWHIPSGAVEDGENPQDAAVREACEETGLRVRPVKFLGAYLGRFPDGVLILRHVWLAEPEPGQTLAPAFTDEIAEASFVSREDFAQLYAAGQIRMYQTKLFYADALREKGFPALPV

112121222222121222222222222222222222222222222222221222222222222222222222222222222222221212121212222222222222222222222222222222222222222222222222222222222222222

>3GQNA

HHHHHHFADLVIQVIDELKQFGVSVKTYGAKGDGVTDDIRAFEKAIESGFPVYVPYGTFMVSRGIKLPSNTVLTGAGKRNAVIRFMDSVGRGESLMYNENVTTGNENIFLSSFTLDGNNKRLGQGISGIGGSRESNLSIRACHNVYIRDIEAVDCTLHGIDITCGGLDYPYLGDGTTAPNPSENIWIENCEATGFGDDGITTHHSQYINILNCYSHDPRLTANCNGFEIDDGSRHVVLSNNRSKGCYGGIEIKAHGDAPAAYNISINGHMSVEDVRSYNFRHIGHHAATAPQSVSAKNIVASNLVSIRPNNKRGFQDNATPRVLAVSAYYGVVINGLTGYTDDPNLLTETVVSVQFRARNCSLNGVVLTGFSNSENGIYVIGGSRGGDAVNISNVTLNNSGRYGVSIGSGIENVSITNISGIGDGINSPVALVSTINSNPEISGLSSIGYPTVARVAGTDYNDGLTLFNGAFRASTTSSGKIHSEGFIMGSTSGCEASVSKSGVLTSSSSKTSSERSLIAGSSTSEAKGTYNTILGSLGAVADEQFAALISASQSRASGNHNLILSSYGINTTGSYKVNGGFEKINWELDSLNGRIKARDTVTGGNTWSDFAEYFESLGGQVIETGYLVTLEKGKIRKAEKGEKIIGVISETAGFVLGESSFEWQGAVLKNEFGGIIYEEVTTEDGVKFKRPLPNPDFDPNKNYIPRSQRREWHVVGLLGQIAVRIDETVKQGHSIDAVGGVATDGDNFIVQEITTPYTKEKGYGVAIVLVK

2222222222222222222222222222222222222222222222222222222222222222222222222222222222222222222222222222222222222222222222222222222222222222222222222222222222222222222222222222222222222222222222222222222222222222222222222222222222222222222222222222222222222222222222222222222222222222222222222222222222222222222222222222222222222222222222222222222222222222222222222222222222222222222222222222222222222222222222222222222222222222222222222222222222222222222222222222222222222222222222222222222222222222222222222222222222222222222222222222222222222222222222222222222222222222222222222222222222222222222222222222222222222222222222222222222222222222222222222222222222222221122222222222222222222222222222221112211112122122122222222222222222222222222222222222222222222222222222222222

>1MB9A

MGAPVLPAAFGFLASARTGGGRAPGPVFATRGSHTDIDTPQGERSLAATLVHAPSVAPDRAVARSLTGAPTTAVLAGEIYNRDELLSVLPAGPAPEGDAELVLRLLERYDLHAFRLVNGRFATVVRTGDRVLLATDHAGSVPLYTCVAPGEVRASTEAKALAAHRDPKGFPLADARRVAGLTGVYQVPAGAVMDIDLGSGTAVTHRTWTPGLSRRILPEGEAVAAVRAALEKAVAQRVTPGDTPLVVLSGGIDSSGVAACAHRAAGELDTVSMGTDTSNEFREARAVVDHLRTRHREITIPTTELLAQLPYAVWASESVDPDIIEYLLPLTALYRALDGPERRILTGYGADIPLGGMHREDRLPALDTVLAHDMATFDGLNEMSPVLSTLAGHWTTHPYWDREVLDLLVSLEAGLKRRHGRDKWVLRAAMADALPAETVNRPKLGVHEGSGTTSSFSRLLLDHGVAEDRVHEAKRQVVRELFDLTVGGGRHPSEVDTDDVVRSVADRTARGAA

222222222222222222222222222222222222222222222222222222222222222222222222222222222222222222222222222222222222222222222222222222222222222222222222222222222222222222222222222222222222222222222222222222222222222222222222222222222222222222222222222222111211112222222222222222111222222222222222222222222222222222222222222222222222222221221222222222222211221222222222222222222222222222222222222222222222222222222222222222222222221222222222222222222212222222222222222222222222222222222222222222222222222222222222222222222

>2I4OA

MGSSHHHHHHSSGLVPRGSHMRLSRFFLPILKENPKEAEIVSHRLMLRAGMLRQEAAGIYAWLPLGHRVLKKIEQIVREEQNRAGAIELLMPTLQLADLWRESGRYDAYGPEMLRIADRHKRELLYGPTNEEMITEIFRAYIKSYKSLPLNLYHIQWKFRDEQRPRFGVMRGREFLMKDAYSFDVDEAGARKSYNKMFVAYLRTFARMGLKAIPMRAETGPIGGDLSHEFIVLAETGESGVYIDRDVLNLPVPDENVDYDGDLTPIIKQWTSVYAATEDVHEPARYESEVPEANRLNTRGIEVGQIFYFGTKYSDSMKANVTGPDGTDAPIHGGSYGVGVSRLLGAIIEACHDDNGIIWPEAVAPFRVTILNLKQGDAATDAACDQLYRELSAKGVDVLYDDTDQRAGAKFATADLIGIPWQIHVGPRGLAEGKVELKRRSDGARENLALADVVARLT

22222222222222222222222222222222222222222222222222222222222222222222222222222222222222222222222222222222222222222222222222222222222222222222222222222222222222212122222211112212122222222222222222222222222222222222222222222222222222222222222222222222222222222222222222222222222222222222222222222222222221111222222222222222222222222222222222122122222222222222222222222222222222222222222222222222222222222222222222222222222222222222222222222222222222222222222222

>1GOLA

HHHHHHMAAAAAAGPEMVRGQVFDVGPRYTNLSYIGEGAYGMVCSAYDNLNKVRVAIRKISPFEHQTYCQRTLREIKILLRFRHENIIGINDIIRAPTIEQMKDVYIVQDLMETDLYKLLKTQHLSNDHICYFLYQILRGLKYIHSANVLHRDLKPSNLLLNTTCDLKICDFGLARVADPDHDHTGFLTEYVATRWYRAPEIMLNSKGYTKSIDIWSVGCILAEMLSNRPIFPGKHYLDQLNHILGILGSPSQEDLNCIINLKARNYLLSLPHKNKVPWNRLFPNADSKALDLLDKMLTFNPHKRIEVEQALAHPYLEQYYDPSDEPIAEAPFKFDMELDDLPKEKLKELIFEETARFQPGYRS

2222222222222222222222222222222222222222221222222222222122222222222222222222222222222222222222222222222222222111221221222222222222222222222222222222222222122221222222222222222222222222222222222222222222222222222222222222222222222222222222222222222222222222222222222222222222222222222222222222222222222222222222222222222222222222222222222222222222222222222222222222

>2QUIA

MPNSEPASLLELFNSIATQGELVRSLKAGNASKDEIDSAVKMLVSLKMSYKAAAGEDYKADCPPGNPAPTSNHGPDATEAEEDFVDPWTVQTSSAKGIDYDKLIVRFGSSKIDKELINRIERATGQRPHHFLRRGIFFSHRDMNQVLDAYENKKPFYLYTGRGPSSEAMHVGHLIPFIFTKWLQDVFNVPLVIQMTDDEKYLWKDLTLDQAYSYAVENAKDIIACGFDINKTFIFSDLDYMGMSSGFYKNVVKIQKHVTFNQVKGIFGFTDSDCIGKISFPAIQAAPSFSNSFPQIFRDRTDIQCLIPCAIDQDPYFRMTRDVAPRIGYPKPALLHSTFFPALQGAQTKMSASDPNSSIFLTDTAKQIKTKVNKHAFSGGRDTIEEHRQFGGNCDVDVSFMYLTFFLEDDDKLEQIRKDYTSGAMLTGELKKALIEVLQPLIAEHQARRKEVTDEIVKEFMTPRKLSFDFQHHHHHH

222222222222222222222222222222222222222222222222222222222222222222222222222222222222222222222222222222222222222222222222222222222222222222222222222222222222222221121121212112222222222222222222222222222222222222222222222222222222222222222222222222222222222222222222222222222222222222222222222222222222222222222221122222222222222222222222221122222222111112222222222222222222222222222222222222222222222222222222222222222222222222222222222222222222222222222222222222222222222222222

>3C16A

MHHHHHHAMEMKADINAKQEDMMFHKIYIQKHDNVSILFADIEGFTSLASQCTAQELVMTLNELFARFDKLAAENHCLRIKILGDCYYCVSGLPEARADHAHCCVEMGMDMIEAISLVREMTGVNVNMRVGIHSGRVHCGVLGLRKWQFDVWSNDVTLANHMEAGGKAGRIHITKATLSYLNGDYEVEPGCGGERNAYLKEHSIETFLILRCTQKRKEEKAMIAK

222222222222222222222222222222222222222211121122222222222222222222222222222222222211122222222222222222222222222222222222222222221222222222222222222222222222222222222222222222222222222222222222222222222222222222222222222222222

>3C16B

RSLKNEELYHQSYDCVCVMFASIPDFKEFYTESDVNKEGLECLRLLNEIIADFDDLLSKPKFSGVEKIKTIGSTYMAATGLSAIPSQEHAQEPERQYMHIGTMVEFAYALVGKLDAINKHSFNDFKLRVGINHGPVIAGVIGAQKPQYDIWGNTVNVASRMDSTGVLDKIQVTEETSLILQTLGYTCTCRGIINVKGKGDLKTYFVNTEMSR

22222222222222222222222222222222222222222222222222222222222222222222222222222222222222222222222222222222222222222222222222222222222222222222222222221122222122222222222222222222222222222222222222212222222222222222

>1E2QA

GSHMAARRGALIVLEGVDRAGKSTQSRKLVEALCAAGHRAELLRFPERSTEIGKLLSSYLQKKSDVEDHSVHLLFSANRWEQVPLIKEKLSQGVTLVVDRYAFSGVAFTGAKENFSLDWCKQPDVGLPKPDLVLFLQLQLADAAKRGAFGHERYENGAFQERALRCFHQLMKDTTLNWKMVDASKSIEAVHEDIRVLSEDAIATATEKPLGELWK

22222222222222222111111122222222222222222222222222222222222222222222222222222222222222222222222222212222222222222222222222222222222222222222222221222222222222222222222222222222222222221112222222222222222222222222222

>3C9RA

MGSHHHHHHDITSLYKKAGSAAAVLEENLYFQGSFTMRLKELGEFGLIDLIKKTLESKVIGDDTAPVEYCSKKLLLTTDVLNEGVHFLRSYIPEAVGWKAISVNVSDVIANGGLPKWALISLNLPEDLEVSYVERFYIGVKRACEFYKCEVVGGNISKSEKIGISVFLVGETERFVGRDGARLGDSVFVSGTLGDSRAGLELLLMEKEEYEPFELALIQRHLRPTARIDYVKHIQKYANASMDISDGLVADANHLAQRSGVKIEILSEKLPLSNELKMYCEKYGKNPIEYALFGGEDYQLLFTHPKERWNPFLDMTEIGRVEEGEGVFVDGKKVEPKGWKHF

222222222222222222222222222222222222222222221221221222222221111222222222222222122222222222222222222222222212222222222221222222222222222212222222222222221111222222222222222222222122222222222222222222222222222222222222222222222222222222222222221211222222222222222222222222222222222222222222222222222222222222222222222222222222222222222222222222

>1U5RA

MSYYHHHHHHDYDIPTTENLYFQGAMDPMPAGGRAGSLKDPDVAELFFKDDPEKLFSDLREIGHGSFGAVYFARDVRNSEVVAIKKMSYSGKQSNEKWQDIIKEVRFLQKLRHPNTIQYRGCYLREHTAWLVMEYCLGSASDLLEVHKKPLQEVEIAAVTHGALQGLAYLHSHNMIHRDVKAGNILLSEPGLVKLGDFGSASIMAPANUFVGTPYWMAPEVILAMDEGQYDGKVDVWSLGITCIELAERKPPLFNMNAMSALYHIAQNESPALQSGHWSEYFRNFVDSCLQKIPQDRPTSEVLLKHRFVLRERPPTVIMDLIQRTKDAVRELDNLQYRKMKKILFQEA

222222222222222222222222222222222222222222222222222222222222212111222122222222222212122222222222222222222222222222221222222222222222112122222222222222222222222222222222222222222222222121222222222212222222222222222222222222222222222222222222222222222222222222222222222222222222222222222222222222222222222222222222222222222222222222222222222221222222

## #ADP of NUC5

>1T6XA

MVVSIGVFDGVHIGHQKVLRTMKEIAFFRKDDSLIYTISYPPEYFLPDFPGLLMTVESRVEMLSRYARTVVLDFFRIKDLTPEGFVERYLSGVSAVVVGRDFRFGKNASGNASFLRKKGVEVYEIEDVVVQGKRVSSSLIRNLVQEGRVEEIPAYLGRYFEIEGIVHKDREFGRKLGFPTANIDRGNEKLVDLKRGVYLVRVHLPDGKKKFGVMNVGFRPTVGDARNVKYEVYILDFEGDLYGQRLKLEVLKFMRDEKKFDSIEELKAAIDQDVKSARNMIDDIINSKFEKEG

22222222222222222222222222222222222222222222222222222222222222222222222222222222222222222222222222222222222222222222222222222222222222222222222222222222222222222222222222222222221111222222222222222222222222222222222222222222222222212122121111222222222222222222222222222222222222222222222222222

>2JLSA

GSAMGEPDYEVDEDIFRKKRLTIMDLHPGAGKTKRILPSIVREALKRRLRTLILAPTRVVAAEMEEALRGLPIRYQTPAVKSDHTGREIVDLMCHATFTTRLLSSTRVPNYNLIVMDEAHFTDPCSVAARGYISTRVEMGEAAAIFMTATPPGSTDPFPQSNSPIEDIEREIPERSWNTGFDWITDYQGKTVWFVPSIKAGNDIANCLRKSGKRVIQLSRKTFDTEYPKTKLTDWDFVVTTDISEMGANFRAGRVIDPRRCLKPVILTDGPERVILAGPIPVTPASAAQRRGRIGRNPAQEDDQYVFSGDPLKNDEDHAHWTEAKMLLDNIYTPEGIIPTLFGPEREKTQAIDGEFRLRGEQRKTFVELMRRGDLPVWLSYKVASAGISYKDREWCFTGERNNQILEENMEVEIWTREGEKKKLRPKWLDARVYADPMALKDFKEFASGRK

2222222222222222222222222221111111222222222222222222222222222222222222222222222222222222222222222222222222222222222222222222222222222222222222222222222222222222212222222222222222222222222222222222222222222222222222222222222222222222222222222222222222122222222222222222222222222222222222222222222122222222222222222222222222222222222222222222222222222222222222222222222222222222222222222222222222222222222222222222222222222222222222222222222222222222222

>1IOVA

MTDKIAVLLGGTSAEREVSLNSGAAVLAGLREGGIDAYPVDPKEVDVTQLKSMGFQKVFIALHGRGGEDGTLQGMLELMGLPYTGSGVMASALSMDKLRSKLLWQGAGLPVAPWVALTRAEFEKGLSDKQLAEISALGLPVIVKPSREGSSVGMSKVVAENALQDALRLAFQHDEEVLIEKWLSGPEFTVAILGEEILPSIRIQPSGTFYDYEAKYLSDETQYFCPAGLEASQEANLQALVLKAWTTLGCKGWGRIDVMLDSDGQFYLLEANTSPGMTSHSLVPMAARQAGMSFSQLVVRILELAD

222222222222222222222222222222222222222222222222222222222222222222222222222222222222222222222222122222222222222222222222222222222222222222222121222111122122222222222222222222222221111222122222222222222222222211222212222222222222222222222222222222222222222212122222222211222222222222222222222222222222222222

>2IOPA

MKGQETRGFQSEVKQLLHLMIHSLYSNKEIFLRELISNASDAADKLRFRALSNPDLYEGDGELRVRVSFDKDKRTLTISDNGVGMTRDEVIDHLGTIAKSGTKSFLESLGSDQAKDSQLIGQFGVGFYSAFIVADKVTVRTRAAGEKPENGVFWESAGEGEYTVADITKEDRGTEITLHLREGEDEFLDDWRVRSIISKYSDHIALPVEIEKREEKDGETVISWEKINKAQALWTRNKSEITDEEYKEFYKHIAHDFNDPLTWSHNRVEGKQEYTSLLYIPSQAPWDMWNRDHKHGLKLYVQRVFIMDDAEQFMPNYLRFVRGLIDSSDLPLNVSREILQDSTVTRNLRNALTKRVLQMLEKLAKDDAEKYQTFWQQFGLVLKEGPAEDFANQEAIAKLLRFASTHTDSSAQTVSLEDYVSRMKEGQEKIYYITADSYAAAKSSPHLELLRKKGIEVLLLSDRIDEWMMNYLTEFDGKPFQSVSKVDESLEKLADEVDESAKEAEKALTPFIDRVKALLGERVKDVRLTHRLTDTPAIVSTDADEMSTQMAKLFAAAGQKVPEVKYIFELNPDHVLVKRAADTEDEAKFSEWVELLLDQALLAERGTLEDPNLFIRRMNQLLVS

222222222222222222222222222222222222212222222222222222222222222222222222222222212222122222221221112222222222222222222222222221122222222222222222222222222222222222222222222221222222222222222222222222222222222222222222222222222222222222222222222222222222222222222222222222222222222222222222222222222222222222222222222222222222222222222222222222222222222222222222222222222222222222222222222222222222222222222222222222222222222222222222222222222222222222222222222222222222222222222222222222222222222222222222222222222222222222222222222222222222222222222222222222222222222222222222222222222222222222222222222222222222222222222222

>1A9XA

MPKRTDIKSILILGAGPIVIGQACEFDYSGAQACKALREEGYRVINVNSNPATIMTDPEMADATYIEPIHWEVVRKIIEKERPDAVLPTMGGQTALNCALELERQGVLEEFGVTMIGATADAIDKAEDRRRFDVAMKKIGLETARSGIAHTMEEALAVAADVGFPCIIRPSFTMGGSGGGIAYNREEFEEICARGLDLSPTKELLIDESLIGWKEYEMEVVRDKNDNCIIVCSIENFDAMGIHTGDSITVAPAQTLTDKEYQIMRNASMAVLREIGVETGGSNVQFAVNPKNGRLIVIEMNPRVSRSSALASKATGFPIAKVAAKLAVGYTLDELMNDITGGRTPASFEPSIDYVVTKIPRFNFEKFAGANDRLTTQMKSVGEVMAIGRTQQESLQKALRGLEVGATGFDPKVSLDDPEALTKIRRELKDAGADRIWYIADAFRAGLSVDGVFNLTNIDRWFLVQIEELVRLEEKVAEVGITGLNADFLRQLKRKGFADARLAKLAGVREAEIRKLRDQYDLHPVYKRVDTCAAEFATDTAYMYSTYEEECEANPSTDREKIMVLGGGPNRIGQGIEFDYCCVHASLALREDGYETIMVNCNPETVSTDYDTSDRLYFEPVTLEDVLEIVRIEKPKGVIVQYGGQTPLKLARALEAAGVPVIGTSPDAIDRAEDRERFQHAVERLKLKQPANATVTAIEMAVEKAKEIGYPLVVRASYVLGGRAMEIVYDEADLRRYFQTAVSVSNDAPVLLDHFLDDAVEVDVDAICDGEMVLIGGIMEHIEQAGVHSGDSACSLPAYTLSQEIQDVMRQQVQKLAFELQVRGLMNVQFAVKNNEVYLIEVNPRAARTVPFVSKATGVPLAKVAARVMAGKSLAEQGVTKEVIPPYYSVKEVVLPFNKFPGVDPLLGPEMRSTGEVMGVGRTFAEAFAKAQLGSNSTMKKHGRALLSVREGDKERVVDLAAKLLKQGFELDATHGTAIVLGEAGINPRLVNKVHEGRPHIQDRIKNGEYTYIINTTSGRRAIEDSRVIRRSALQYKVHYDTTLNGGFATAMALNADATEKVISVQEMHAQIK

22222222222222222222222222222222222222222222222222222222222222222222222222222222222222222222222222222222222222222222222222222222122222222222222122222222222222222222221212221111222222222222222222222222222222112112221222222222222222222222222111112222222222222222222222222222222222222222122222222222211222222222222222222222222222222222222222222222222222222222222222222222222222212222222222222222222222222222222222222222222222222222222222222222222222222222222222222222222222222222222222222222222222222222222222222222222222222222222222222222222222222222222222222222222222222222222222222222222222222222222222222222222222222222222222222222222222222222222222222222222222222222222222222222222222222122222222222222222222221212222222221222222222222222222222222222211122221222222222222222222222221111122222222222222222222222222222222222222212222222222212222222222222222222222222222222222222222222222222222222222222222222122222222222222222222222222222222222222222222222222222222222222222222222222222222222222222222222222222222222222222222222222222222222222222222222222222222222222222222

>2YX6B

MRVAIPAEDDRGIKSNVSKHFGRSRYFVFVDIEGEDVKNVEVVEVPFEEHGPGDLPNFIKDHGAKIVLTYGIGRRAIEYFNSLGISVVTGVYGRISDVIKAFIGGKLKIDYDWKEKIEKEH

2222222222222222222222222222222222222222222222222222222222222222222222222122122222222212122222222222222222222222212222222

>1TF2A

GPHMLGILNKMFDPTKRTLNRYEKIANDIDAIRGDYENLSDDALKHKTIEFKERLEKGATTDDLLVEAFAVVREASRRVTGMFPFKVQLMGGVALHDGNIAEMKTGEGKTLTSTLPVYLNALTGKGVHVVTVNEYLASRDAEQMGKIFEFLGLTVGLNLNSMSKDEKREAYAADITYSTNNELGFDYLRDNMVLYKEQMVQRPLHFAVIDEVDSILIDEARTPLIISGQAAKSTKLYVQANAFVRTLKAEKDYTYDIKTKAVQLTEEGMTKAEKAFGIDNLFDVKHVALNHHINQALKAHVAMQKDVDYVVEDGQVVIVDSFTGRLMKGRRYSEGLHQAIEAKEGLEIQNESMTLATITFQNYFRMYEKLAGMTGTAKTEEEEFRNIYNMQVVTIPTNRPVVRDDRPDLIYRTMEGKFKAVAEDVAQRYMTGQPVLVGTVAVETSELISKLLKNKGIPHQVLNAKNHEREAQIIEEAGQKGAVTIATNMAGRGTDIKLGEGVKELGGLAVVGTERHESRRIDNQLRGRSGRQGDPGITQFYLSMEDELMRRFGAERTMAMLDRFGMDDSTPIQSKMVSRAVESSQKRVEGNNFDSRKQLLQYDDVLRQQREVIYKQRFEVIDSENLREIVENMIKSSLERAIAAYTPREELPEEWKLDGLVDLINTTYLDEGALEKSDIFGKEPDEMLELIMDRIITKYNEKEEQFGKEQMREFEKVIVLRAVDSKWMDHIDAMDQLRQGIHLRAYAQTNPLREYQMEGFAMFEHMIESIEDEVAKFVMKAEIENNLEREEVVQGQTTAHQPQEGDDNKKAKKAPVRKVVDIGRNAPCHCGSGKKYKNCCGRTE

2222222222222222222222222222222222222222222222222222222222222222222222222222222221111221222222222222222121111112222222222222222222222222222222222222222222222222222222222222222222222222222222222222222222222222222222222222222222222222222222222222222222222222222222222222222222222222222222222222222222222222222222222222222222222222222222222222222222222222222222222222222222222222222222222222222222222222222222222222222222222222222222222222222222222222222222222222222222222222222222222222222222222212122222222222222222222222222222222212222222222222222222222222222222222222222222222222222222222222222222222222222222222222222222222222222222222222222222222222222222222222222222222222222222222222222222222222222222222222222222222222222222222222222222222222222222222222222222222222222222222222222222222222222222222222222222222222222222222222222222222222

>1GSAA

MIKLGIVMDPIANINIKKDSSFAMLLEAQRRGYELHYMEMGDLYLINGEARAHTRTLNVKQNYEEWFSFVGEQDLPLADLDVILMRKDPPFDTEFIYATYILERAEEKGTLIVNKPQSLRDCNEKLFTAWFSDLTPETLVTRNKAQLKAFWEKHSDIILKPLDGMGGASIFRVKEGDPNLGVIAETLTEHGTRYCMAQNYLPAIKDGDKRVLVVDGEPVPYCLARIPQGGETRGNLAAGGRGEPRPLTESDWKIARQIGPTLKEKGLIFVGLDIIGDRLTEINVTSPTCIREIEAEFPVSITGMLMDAIEARLQQQ

2222222222222222222222222222222222222222222222222222222222222222222222222222222222222222222222222222222222222222222222222222122222222222222222222222222222222121222111112122222222222222222222222222211112212221222222222222222222222221111222222222222222222222222222222222222212122222122222222222222222222222222222222222

>2CN5A

GPLGSHMSVYPKALRDEYIMSKTLGSGACGEVKLAFERKTCKKVAIKIISKRKFAIGSAREADPALNVETEIEILKKLNHPCIIKIKNFFDAEDYYIVLELMEGGELFDKVVGNKRLKEATCKLYFYQMLLAVQYLHENGIIHRDLKPENVLLSSQEEDCLIKITDFGHSKILGETSLMRTLCGTPTYLAPEVLVSVGTAGYNRAVDCWSLGVILFICLSGYPPFSEHRTQVSLKDQITSGKYNFIPEVWAEVSEKALDLVKKLLVVDPKARFTTEEALRHPWLQDEDMKRKFQDLLSEENESTALPQVLAQPSTSRKRPREGEAEGAE

22222222222222222222222211111121222222222222121222222222222222222222222222222222222122222222222222211122212222222222222222222222222222222222222222221121222222222222212222222222222222222222222222222222222222222222222222222222222222222222222222222222222222222222222222222222222222222222222222222222222222222222222222222222222222222

>2V1XA

MCLEDSDAGASNEYDSSPAAWNKEDFPWSGKVKDILQNVFKLEKFRPLQLETINVTMAGKEVFLVMPTGGGKSLCYQLPALCSDGFTLVICPLISLMEDQLMVLKQLGISATMLNASSSKEHVKWVHAEMVNKNSELKLIYVTPEKIAKSKMFMSRLEKAYEARRFTRIAVDEVHCCSQWGHDFRPDYKALGILKRQFPNASLIGLTATATNHVLTDAQKILCIEKCFTFTASFNRPNLYYEVRQKPSNTEDFIEDIVKLINGRYKGQSGIIYCFSQKDSEQVTVSLQNLGIHAGAYHANLEPEDKTTVHRKWSANEIQVVVATVAFGMGIDKPDVRFVIHHSMSKSMENYYQESGRAGRDDMKADCILYYGFGDIFRISSMVVMENVGQQKLYEMVSYCQNISKCRRVLMAQHFDEVWNSEACNKMCDNCCKDSAFERKNITEYCRDLIKILKQAEELNEKLTPLKLIDSWMGKGAAKLRVAGVVAPTLPREDLEKIIAHFLIQQYLKEDYSFTAYATISYLKIGPKANLLNNEAHAITMQVTKSTQNSFRAESSQTCHSEQGDKKMEAENLYFQSHHHHHHDYKDDDDK

222222222222222222222222222222222222222221211122122222222222222222111111112222222222222222222222222222222222222222222222222222222222222222222222222222222222222222222222222222222222222222222222222222222222222222222222222222222222222222222222222222222222222222222222222222222222222222222222222222222222222222222222222222222222222222212222222222222222222222222222222222222222222222222222222222222222222222222222222222222222222222222222222222222222222222222222222222222222222222222222222222222222222222222222222222222222222222222222222222222222222222222222222222222222222222222222222222222222222

>1AO0A

CGVFGIWGHEEAPQITYYGLHSLQHRGQEGAGIVATDGEKLTAHKGQGLITEVFQNGELSKVKGKGAIGHVRYATAGGGGYENVQPLLFRSQNNGSLALAHNGNLVNATQLKQQLENQGSIFQTSSDTEVLAHLIKRSGHFTLKDQIKNSLSMLKGAYAFLIMTETEMIVALDPNGLRPLSIGMMGDAYVVASETCAFDVVGATYLREVEPGEMLIINDEGMKSERFSMNINRSICSMEYIYFSRPDSNIDGINVHSARKNLGKMLAQESAVEADVVTGVPDSSISAAIGYAEATGIPYELGLIKNRYVGRTFIQPSQALREQGVRMKLSAVRGVVEGKRVVMVDDSIVRGTTSRRIVTMLREAGATEVHVKISSPPIAHPCFYGIDTSTHEELIASSHSVEEIRQEIGADTLSFLSVEGLLKGIGRKYDDSNCGQCLACFTGKYPTEIYQDTVLPHVK

222222222222222222222222122222222222222222222222222222222222222222222222222222222222222222222222222222222222222222222222222222222222222222222222222222222222222222222222222222222222222222222222222222222222222222222222222222222222222222222222212111222222222222122222222222222222222211122222222222222222222111122222222222222222222122222222222222222222222222222222222222222222222222222222222222222222222222222222222222222222222222222222222222222222222222222222222

>1I58B

GSHMVPISFVFNRFPRMVRDLAKKMNKEVNFIMRGEDTELDRTFVEEIGEPLLHLLRNAIDHGIEPKEERIAKGKPPIGTLILSARHEGNNVVIEVEDDGRGIDKEKIIRKAIEKGLIDESKAATLSDQEILNFLFVPGFSTKEKVSEVSGRGVGMDVVKNVVESLNGSISIESEKDKGTKVTIRLPLT

222222222222222222222222222222222222222222222222222221222112211222222222222222222222222222222222212221122212222222222222222222222222222222221222222222222211222222222222222222222221222222222

>1T5CA

AEEGAVAVCVRVRPLNSREESLGETAQVYWKTDNNVIYQVDGSKSFNFDRVFHGNETTKNVYEEIAAPIIDSAIQGYNGTIFAYGQTASGKTYTMMGSEDHLGVIPRAIHDIFQKIKKFPDREFLLRVSYMEIYNETITDLLCGTQKMKPLIIREDVNRNVYVADLTEEVVYTSEMALKWITKGEKSRHYGETKMNQRSSRSHTIFRMILESREKGEPSNCEGSVKVSHLNLVDLAGSERAAQTGAAGVRLKEGCNINRSLFILGQVIKKLSDGQVGGFINYRDSKLTRILQNSLGGNAKTRIICTITPVSFDETLTALQFASTAKYMKNTPYVNEVSTDELEHHHHHH

2222222222121122222222222222222222222222222222222222222222222222222222222222222222222111111112222222222222222222222222222222222222222222222222222222222222222222222222222222222222222222222222222222222222222222222222222222222222222222222222222222222222222222222222222222222222222222222222222222222222222222222222222222222222222222222222222222222222222

>1PKGA

QKPMYEVQWKVVEEINGNNUVUIDPTQLPYDHKWEFPRNRLSFGKTLGAGAFGKVVEATAYGLIKSDAAMTVAVKMLKPSAHLTEREALMSELKVLSYLGNHMNIVNLLGACTIGGPTLVITEYCCYGDLLNFLRRKRDSFICSKTSPAIMEDDELALDLEDLLSFSYQVAKGMAFLASKNCIHRDLAARNILLTHGRITKICDFGLARDIKNDSNYVVKGNARLPVKWMAPESIFNCVYTFESDVWSYGIFLWELFSLGSSPYPGMPVDSKFYKMIKEGFRMLSPEHAPAEMYDIMKTCWDADPLKRPTFKQIVQLIEKQISESTNHI

22222222222222222222222222222222222222222222221121111212222222222222222212122222222222222222222222222222212222222222222221121222122222222222222222222222222222222222222222222222222222222222211212222222222122222222222222222222222222222222222222222222222222222222222222222222222222222222222222222222222222222222222222222222222222222

>1FP6A

AMRQCAIYGKGGIGKSTTTQNLVAALAEMGKKVMIVGCDPKADSTRLILHSKAQNTIMEMAAEAGTVEDLELEDVLKAGYGGVKCVESGGPEPGVGCAGRGVITAINFLEEEGAYEDDLDFVFYDVLGDVVCGGFAMPIRENKAQEIYIVCSGEMMAMYAANNISKGIVKYANSGSVRLGGLICNSRNTDREDELIIALANKLGTQMIHFVPRDNVVQRAEIRRMTVIEYDPKAKQADEYRALARKVVDNKLLVIPNPITMDELEELLMEFGIMEVEDESIVGKTAEEV

2222222221211111122222222222222222222222222222222222222222222222222222222222222222222222222222222222222222222222222222222222222222222222222222222222222222222222222222222222222222222222122222222222222222222222221111221122122222222222222122212222222222222222222222222222222222222222222222222

>2J9DB

GSMKKVEAIIRPEKLEIVKKALSDAGYVGMTVSEVKGRGVQGGIVERYRGREYIVDLIPKVKIELVVKEEDVDNVIDIICENARTGNPGDGKIFVIPVERVVRVRTKEEGKEALLEHHH

22222222222222222222222222221112222222222222222222222222222222211122222222222222222222222222222222222212122222222222222

>3B5ZA

MHNDKDLSTWQTFRRLWPTIAPFKAGLIVAGIALILNAASDTFMLSLLKPLLDDGFGKTDRSVLLWMPLVVIGLMILRGITSYISSYCISWVSGKVVMTMRRRLFGHMMGMPVAFFDKQSTGTLLSRITYDSEQVASSSSGALITVVREGASIIGLFIMMFYYSWQLSIILVVLAPIVSIAIRVVSKRFRSISKNMQNTMGQVTTSAEQMLKGHKEVLIFGGQEVETKRFDKVSNKMRLQGMKMVSASSISDPIIQLIASLALAFVLYAASFPSVMDSLTAGTITVVFSSMIALMRPLKSLTNVNAQFQRGMAACQTLFAILDSEQEKDEGKRVIDRATGDLEFRNVTFTYPGREVPALRNINLKIPAGKTVALVGRSGSGKSTIASLITRFYDIDEGHILMDGHDLREYTLASLRNQVALVSQNVHLFNDTVANNIAYARTEEYSREQIEEAARMAYAMDFINKMDNGLDTIIGENGVLLSGGQRQRIAIARALLRDSPILILDEATSALDTESERAIQAALDELQKNRTSLVIAHRLSTIEQADEIVVVEDGIIVERGTHSELLAQHGVYAQLHKMQFGQ

222222222222222222222222222222222222222222222222222222222222222222222222222222222222222222222222222222222222222222222222222222222222222222222222222222222222222222222222222222222222222222222222222222222222222222222222222222222222222222222222222222222222222222222222222222222222222222222222222222222222222222222222222222222222222222222222222222222222222222222222222222222222222221121222222222222222222222222222222222222222222222222222222222222222222222222222222222222222222222222221212222222222222222222222222222222222222222222222222222222222222222222222222222222222222222222222222222

>3C9UA

MGSHHHHHHDITSLYKKAGSAAAVLEENLYFQGSFTMRLKELGEFGLIDLIKKTLESKVIGDDTAPVEYCSKKLLLTTDVLNEGVHFLRSYIPEAVGWKAISVNVSDVIANGGLPKWALISLNLPEDLEVSYVERFYIGVKRACEFYKCEVVGGNISKSEKIGISVFLVGETERFVGRDGARLGDSVFVSGTLGDSRAGLELLLMEKEEYEPFELALIQRHLRPTARIDYVKHIQKYANASMDISDGLVADANHLAQRSGVKIEILSEKLPLSNELKMYCEKYGKNPIEYALFGGEDYQLLFTHPKERWNPFLDMTEIGRVEEGEGVFVDGKKVEPKGWKHF

222222222222222222222222222222222222222222221221221222222221111222222222222222122222222222222222222222222212222222222221212222222222222212222222222222221111222222222222222222222122222222222222222222222222222222222222222222222222222222222222222222222222222222222222222222222222222222222222222222222222222222222222222222222222222222222222222222

>1EQMA

TVAYIAIGSNLASPLEQVNAALKALGDIPESHILTVSSFYRTPPLGPQDQPDYLNAAVALETSLAPEELLNHTQRIELQQGRVRKAERWGPRTLDLDIMLFGNEVINTERLTVPHYDMKNRGFMLWPLFEIAPELVFPDGEMLRQILHTRAFDKLNKW

22222222222222222222222222222222222222222222222222222222222222222222212221222222222222222221221211222222222221112222222222222222222222222222222222222222222222

>3FMPB

MATDSWALAVDEQEAAAESLSNLHLKEEKIKPDTNGAVVKTNANAEKTDEEEKEDRAAQSLLNKLIRSNLVDNTNQVEVLQRDPNSPLYSVKSFEELRLKPQLLQGVYAMGFNRPSKIQENALPLMLAEPPQNLIAQSQSGTGKTAAFVLAMLSQVEPANKYPQCLCLSPTYELALQTGKVIEQMGKFYPELKLAYAVRGNKLERGQKISEQIVIGTPGTVLDWCSKLKFIDPKKIKVFVLDEADVMIATQGHQDQSIRIQRMLPRNCQMLLFSATFEDSVWKFAQKVVPDPNVIKLKREEETLDTIKQYYVLCSSRDEKFQALCNLYGAITIAQAMIFCHTRKTASWLAAELSKEGHQVALLSGEMMVEQRAAVIERFREGKEKVLVTTNVCARGIDVEQVSVVINFDLPVDKDGNPDNETYLHRIGRTGRFGKRGLAVNMVDSKHSMNILNRIQEHFNKKIERLDTDDLDEIEKIAN

22222222222222222222222222222222222222222222222222222222222222222222212222222222222222222222212222222222222222212121221222222222222222222212111111222222222222222222222222222222222222222222222222222222222222222222222222222222222222222222222222222222222222222222222222222222222222222222222222222222222222222222222222222222222222222222222222222222222222222222222222222222222222222222222222222222222222222222222222222222222222222222222222222222222222222222222222222222222222222222222

>2F1JA

MGDNLTDLPGVGPSTAEKLVEAGYIDFMKIATATVGELTDIEGISEKAAAKMIMGARDLCDLGFKSGIDLLKQRSTVWKLSTSSSELDSVLGGGLESQSVTEFAGVFGSGKTQIMHQSCVNLQNPEFLFYDEEAVSKGEVAQPKAVYIDTDGTFRPERIMQMAEHAGIDGQTVLDNTFVARAYNSDMQMLFAEKIEDLIQEGNNIKLVVIDSLTSTFRNEYTGRGKLAERQQKLGRHMATLNKLADLFNCVVLVTNQVSAKPDAFFGMAEQAIGGHIVGHAATFRFFVRKGKGDKRVAKLYDSPHLPDAEAIFRITEKGIQD

2222222222222222222222222222222222222222222222222222222222222222222222222222222222222222222222222222222221111111122222222222222222222222222222222222222222222122122222222222222222222222222222222222222222222222222222222222222222222222222222222222222222222222222222222222222222222222222222222222222122222222222222222211122222

>2YWVA

GHMPTKQQLLYEGKAKKIYATDEPDVLWVEYKDSATAFNGEKKATIAGKGRLNNEISSLLFLKLREAGIANHFIEKLSPTEQLVRRVTIIPLEVVVRNVVAGSLAKRIGLEEGTPLEAPLVEFYYKNDDLGDPLLLEDHIFILKLASREEVAALKQAALAVNDVLRLHFAERNVRLIDFKLEFGRTADGAILLADEISPDTCRLWDAKTNEKLDKDVFRRDLGSLTDAYEVILQRLGGESACTK

2222222222121111212222222212222222222222222222222222222222222222222222212222222222211212122222222222222222222222222222222222212222222222222222222222222222222222222222222222222222222122222222222112222222222222222222222222222222222222222222222222

>3LV8A

MHHHHHHSSGVDLGTENLYFQSNAMNAKFIVIEGLEGAGKSTAIQVVVETLQQNGIDHITRTREPGGTLLAEKLRALVKEEHPGEELQDITELLLVYAARVQLVENVIKPALARGEWVVGDRHDMSSQAYQGGGRQIAPSTMQSLKQTALGDFKPDLTLYLDIDPKLGLERARGRGELDRIEKMDISFFERARERYLELANSDDSVVMIDAAQSIEQVTADIRRALQDWLSQVNRV

22222222222222222222222222222222221211111122222222222222222222222222222222222222222222222222222222222222222222222222222222222222222222222222222222222222222222222222222222122222222222222222222222222222222222222212111221222222222222222222

>3KB1A

UQKRVTDEDIKERLDKIGFRIAVUSGKGGVGKSTVTALLAVHYAKQGKKVGILDADFLGPSIPHLFGLEKGKVAVSDEGLEPVLTQRLGIKVUSIQFLLPKRETPVIWRGPLIAGUIREFLGRVAWGELDYLLIDLPPGTGDAPLTVUQDAKPNGAVIVSTPQELTAAVVEKAITUAEQTKTAVLGIVENUAYFECPNCGERTYLFGEGKASELARKYKIEFITEIPIDSDLLKLSDLGRVEEYEPDWFEFFPYLEHHHHHH

2222222222222222222222222211111111222222222222222222222222222222222222222222222222222222222222222222222222222222222222222222222222222222222222222222222222222222221222222222222222222222222221222222222222222222222222222222222222111222122122222222222222222222222222

>2FV7A

MGSSHHHHHHSSGLVPRGSWQEEVAAVVVVGSCMTDLVSLTSRLPKTGETIHGHKFFIGFGGKGANQCVQAARLGAMTSMVCKVGKDSFGNDYIENLKQNDISTEFTYQTKDAATGTASIIVNNEGQNIIVIVAGANLLLNTEDLRAAANVISRAKVMVCQLEITPATSLEALTMARRSGVKTLFNPAPAIADLDPQFYTLSDVFCCNESEAEILTGLTVGSAADAGEAALVLLKRGCQVVIITLGAEGCVVLSQTEPEPKHIPTEKVKAVDTTGAGDSFVGALAFYLAYYPNLSLEDMLNRSNFIAAVSVQAAGTQSSYPYKKDLPLTLF

2222222222222222222222222222222222222222222222222222222222222222222222222222222222222222222222222222222222222222222222222222222222222222222222222222222222222222222222222222222222222222222222222222222222222221222222222222222222222222222222222221111212222222222222221121212222211222222222222222222222222221221122122222222222222222222

>2BFRA

UEVLFEAKVGDITLKLAQGDITQYPAKAIVNAANKRLEHGGGVAYAIAKACAGDAGLYTEISKKAMREQFGRDYIDHGEVVVTPAMNLEERGIKYVFHTVGPICSGMWSEELKEKLYKAFLGPLEKAEEMGVESIAFPAVSAGIYGCDLEKVVETFLEAVKNFKGSAVKEVALVIYDRKSAEVALKVFERSL

222222222222222222111222222222212222222221122122222222222222222222222222222222222222222222222222222222222222222222222222222222222222222222121111122222222222222222222222222222212222222222222222

>1XTJA

GSPGHMSSGFRDFLLKPELLRAIVDCGFEHPSEVQHECIPQAILGMDVLCQAKSGMGKTAVFVLATLQQLEPVTGQVSVLVMCHTRELAFQISKEYERFSKYMPNVKVAVFFGGLSIKKDEEVLKKNCPHIVVGTPGRILALARNKSLNLKHIKHFILDECDKMLEQLDMRRDVQEIFRMTPHEKQVMMFSATLSKEIRPVCRKFMQDPMEIFVDDETKLTLHGLQQYYVKLKDNEKNRKLFDLLDVLEFNQVVIFVKSVQRCIALAQLLVEQNFPAIAIHRGMPQEERLSRYQQFKDFQRRILVATNLFGRGMDIERVNIAFNYDMPEDSDTYLHRVARAGRFGTKGLAITFVSDENDAKILNDVQDRFEVNISELPDEIDISSY

22222222212222222222222222212111221222222222222222222111111122222222222222222222222222222222222222222222222222222222222222222222222222222222222222222222222222222222222222222222222222222222222222222222222222222222222222222222222222222222222222222222222222222222222222222222222222222222212222222222222222222222222222222222222222222222222222222222222222222222222222222222222222222222222222

>2VOSA

UNSTNSGPPDSGSATGVVPTPDEIASLLQVEHLLDQRWPETRIDPSLTRISALUDLLGSPQRSYPSIHIAGTNGKTSVARUVDALVTALHRRTGRTTSPHLQSPVERISIDGKPISPAQYVATYREIEPLVALIDQQSQASAGKGGPAUSKFEVLTAUAFAAFADAPVDVAVVEVGUGGRWDATNVINAPVAVITPISIDHVDYLGADIAGIAGEKAGIITRAPDGSPDTVAVIGRQVPKVUEVLLAESVRADASVAREDSEFAVLRRQIAVGGQVLQLQGLGGVYSDIYLPLHGEHQAHNAVLALASVEAFFGAGAQRQLDGDAVRAGFAAVTSPGRLERURSAPTVFIDAAHNPAGASALAQTLAHEFDFRFLVGVLSVLGDKDVDGILAALEPVFDSVVVTHNGSPRALDVEALALAAGERFGPDRVRTAENLRDAIDVATSLVDDAAADPDVAGDAFSRTGIVITGSVVTAGAARTLFGRDPQ

2222222222222222222222222222222222222222222222222222222222222222222222211111122222222222222222222222222222222222222222222222222222222222222222222222222222222222222222222222212222222222222222222222222222222222222222222222222222222222222222222222222222222222222222222222222222222222222222222222222212221222222222222222222222222222222222222122222222222211122211221222222222222222222222222222222222222222222222222222222222222222222222222222222222222222222222222222222222222222222222222222222

>1UKYA

TAATTSQPAFSPDQVSVIFVLGGPGAGKGTQCEKLVKDYSFVHLSAGDLLRAEQGRAGSQYGELIKNCIKEGQIVPQEITLALLRNAISDNVKANKHKFLIDGFPRKMDQAISFERDIVESKFILFFDCPEDIMLERLLERGKTSGRSDDNIESIKKRFNTFKETSMPVIEYFETKSKVVRVRCDRSVEDVYKDVQDAIRDSL

22222222222222222222221111111122222222222222211221122222222222222221122211122221222222222222222222222211212221222222222222222222222222221222122222121222222221222222222222222222222222212111222222222222222

>2HMVA

MGRIKNKQFAVIGLGRFGGSIVKELHRMGHEVLAVDINEEKVNAYASYATHAVIANATEENELLSLGIRNFEYVIVAIGANIQASTLTTLLLKELDIPNIWVKAQNYYHHKVLEKIGADRIIHPEKDMGVKIAQSLSDENVLNY

222222222222111122222222222222222221112212222222222222111122222222222222222211122221222222222222222222222222222222222222222222222222222222222222

>1AONA

AAKDVKFGNDARVKMLRGVNVLADAVKVTLGPKGRNVVLDKSFGAPTITKDGVSVAREIELEDKFENMGAQMVKEVASKANDAAGDGTTTATVLAQAIITEGLKAVAAGMNPMDLKRGIDKAVTAAVEELKALSVPCSDSKAIAQVGTISANSDETVGKLIAEAMDKVGKEGVITVEDGTGLQDELDVVEGMQFDRGYLSPYFINKPETGAVELESPFILLADKKISNIREMLPVLEAVAKAGKPLLIIAEDVEGEALATLVVNTMRGIVKVAAVKAPGFGDRRKAMLQDIATLTGGTVISEEIGMELEKATLEDLGQAKRVVINKDTTTIIDGVGEEAAIQGRVAQIRQQIEEATSDYDREKLQERVAKLAGGVAVIKVGAATEVEMKEKKARVEDALHATRAAVEEGVVAGGGVALIRVASKLADLRGQNEDQNVGIKVALRAMEAPLRQIVLNCGEEPSVVANTVKGGDGNYGYNAATEEYGNMIDMGILDPTKVTRSALQYAASVAGLMITTECMVTDLPKNDAADLGAAGGMGGMGGMGGMM

2222222222222222222222222222111122222222222222222122222222222222222222222222222222222111112222222222222222222222222222222222222222222222222222222222122222222222222222222222222222222222222222222222222222222222222222222222222222222222222222222222222222222222222222222222222222222222222222222222222222222222222222222222222222222222222222222222222222222222222222222222222222222222222222222222222222222222222222222222112222222222222222222222222222222222222212222222222222222222222221112222222222212122222222222222222222222222222222222222222222222222222

>2CDUA

MKVIVVGCTHAGTFAVKQTIADHPDADVTAYEMNDNISFLSUGIALYLGKEIKNNDPRGLFYSSPEELSNLGANVQMRHQVTNVDPETKTIKVKDLITNEEKTEAYDKLIMTTGSKPTVPPIPGIDSSRVYLCKNYNDAKKLFEEAPKAKTITIIGSGYIGAELAEAYSNQNYNVNLIDGHERVLYKYFDKEFTDILAKDYEAHGVNLVLGSKVAAFEEVDDEIITKTLDGKEIKSDIAILCIGFRPNTELLKGKVAMLDNGAIITDEYMHSSNRDIFAAGDSAAVHYNPTNSNAYIPLATNAVRQGRLVGLNLTEDKVKDMGTQSSSGLKLYGRTYVSTGINTALAKANNLKVSEVIIADNYRPEFMLSTDEVLMSLVYDPKTRVILGGALSSMHDVSQSANVLSVCIQNKNTIDDLAMVDMLFQPQFDRPFNYLNILGQAAQAQADKAHK

22222222222222222222222222222222222222222222222222222222222222222222222222222222222222222222222222222222222222222222222221222222222222222222222222222222221121112222222222222222221112222211222222222222222222222222112222222222222222222222222221112222222222222222222222222222222222222222222222222222222222222222222222222222222222222222222222222222222222222222222222222222222222222222222222222222222222222222222222222222222222222222222222222222222222222222

>2E2PA

MMIIVGVDAGGTKTKAVAYDCEGNFIGEGSSGPGNYHNVGLTRAIENIKEAVKIAAKGEADVVGMGVAGLDSKFDWENFTPLASLIAPKVIIQHDGVIALFAETLGEPGVVVIAGTGSVVEGYNGKEFLRVGGRGWLLSDDGSAYWVGRKALRKVLKMMDGLENKTILYNKVLKTINVKDLDELVMWSYTSSCQIDLVASIAKAVDEAANEGDTVAMDILKQGAELLASQAVYLARKIGTNKVYLKGGMFRSNIYHKFFTLYLEKEGIISDLGKRSPEIGAVILAYKEVGCDIKKLISD

22222222222222222222222222222222222222222222222222222222222222222222222222222222222222222222222222222222222222222211222222222222222222222222222122222222222222222222222222222222222222222222222222222211211221222222222222222222222222222222222222222211121122222222222222222222222222222222222222222222222

>3HZ6A

USLAFYIATFDIGTTEVKAALADRDGGLHFQRSIALETYGDGNGPVEQDAGDWYDAVQRIASSWWQSGVDARRVSAIVLSGQUQNFLPLDQDHEPLHRAVLYSDKRPLKEAEEINARHGADNLWSALENPUTAASILPKLVFWRASFPQAFGRLRHVVLGAKDYVVLRLTGRHATDRTNASTTGLYRPKDDAWHVELLADYGFSLDLUPRLLEPGEQVGGVSALAARQTGFVSGTPVLCGLGDAGAATLGVGVLDDEDAYLHLGTTGWLARLTQTDPVGDUPVGTIFRLAGIIAGKTLQVAPVLNAGNILQWALTLVGHRPGEDCAEYFHUAAAEVQGVTVPDGLLFVPYLHAERCPVELPAPRGALLGVTGATTRAQILLAVLEGAALSLRWCAELLGUEKVGLLKVVGGGARSEAWLRUIADNLNVSLLVKPDAHLHPLRGLAALAAVELEWSHSIQDFLREADLREPASNILHPQPCDEGRRRRKFERFKQCVETLGRLDEGHHHHHH

2222222222222222222222222222222222222222222222222222222222222222222222222222222222222222222222222222222222222222222222222222222222222222222222222222222222222222222222222222222222222222222222222222222222222222222222222222222222222222222222222222222222222222222222211222222222222222222222222222222222222222221122122222222222222222122222222222222222222222222222222222222222222222222222222222222222222222222222222111211222222222222222222222222222222222222222222222222222222222222222222222222222222222222222222222222

>3GR4A

MGSSHHHHHHSSGLVPRGSMSKPHSEAGTAFIQTQQLHAAMADTFLEHMCRLDIDSPPITARNTGIICTIGPASRSVETLKEMIKSGMNVARLNFSHGTHEYHAETIKNVRTATESFASDPILYRPVAVALDTKGPEIRTGLIKGSGTAEVELKKGATLKITLDNAYMEKCDENILWLDYKNICKVVEVGSKIYVDDGLISLQVKQKGADFLVTEVENGGSLGSKKGVNLPGAAVDLPAVSEKDIQDLKFGVEQDVDMVFASFIRKASDVHEVRKVLGEKGKNIKIISKIENHEGVRRFDEILEASDGIMVARGDLGIEIPAEKVFLAQKMMIGRCNRAGKPVICATQMLESMIKKPRPTRAEGSDVANAVLDGADCIMLSGETAKGDYPLEAVRMQHLIAREAEAAIYHLQLFEELRRLAPITSDPTEATAVGAVEASFKCCSGAIIVLTKSGRSAHQVARYRPRAPIIAVTRNPQTARQAHLYRGIFPVLCKDPVQEAWAEDVDLRVNFAMNVGKARGFFKKGDVVIVLTGWRPGSGFTNTMRVVPVP

2222222222222222222222222222222222222222222222222222222222222222222212112222222222222222222121221222222222222222222222222222222222222222221222222222222222222222222222222222222222222222222222222222222222222222222222222222222222222222222222222222222222222222222222222222222222222222222222222222222222222222222222222222222222222222222222222222222222222222222222222222222222222222222212221222222222222222222222222222222222222222222222222222222222222222222222222222222222222222222222222222222222222222222222222222222222222222222222222222222222222222222222

>3F61A

GSHMTTPSHLSDRYELGEILGFGGMSEVHLARDLRDHRDVAVKVLRADLARDPSFYLRFRREAQNAAALNHPAIVAVYDTGEAETPAGPLPYIVMEYVDGVTLRDIVHTEGPMTPKRAIEVIADACQALNFSHQNGIIHRDVKPANIMISATNAVKVMDFGIARAIADSGNSVTQTAAVIGTAQYLSPEQARGDSVDARSDVYSLGCVLYEVLTGEPPFTGDSPDSVAYQHVREDPIPPSARHEGLSADLDAVVLKALAKNPENRYQTAAEMRADLVRVHNGEPPEAPKVLTDAERTSLLSSAAGNLSGPR

22222222222222222222111221212222222222221212222222222222222222222222222222122222222222222222221121222122222222222222222222222222222222222222221211212222222221122222222222222222222222222222222222222222222222222222222222222222222222222222222222222222222222222222222222222222222222222222222222222222222222222222222

>3EGIA

GPLGSRLFSRFDDGIKLDREGWFSVTPEKIAEHIAGRVSQSFKCDVVVDAFCGVGGNTIQFALTGURVIAIDIDPVKIALARNNAEVYGIADKIEFICGDFLLLASFLKADVVFLSPPWGGPDYATAETFDIRTUUSPDGFEIFRLSKKITNNIVYFLPRNADIDQVASLAGPGGQVEIEQNFLNNKLKTITAYFGDLIRRPASET

22222222222222222222222222222222222222222222222221121222222222222222222111221222222222222222222222111222222222222222212222222222222222222222222222222222222222222222222222222222222222222222222222222222222222

>1O92B

MNGPVDGLCDHSLSEEGAFMFTSESVGEGHPDKICDQISDAVLDAHLKQDPNAKVACETVCKTGMVLLCGEITSMAMIDYQRVVRDTIKHIGYDDSAKGFDFKTCNVLVALEQQSPDIAQCVHLDRNEEDVGAGDQGLMFGYATDETEECMPLTIVLAHKLNTRMADLRRSGVLPWLRPDSKTQVTVQYVQDNGAVIPVRVHTIVISVQHNEDITLEAMREALKEQVIKAVVPAKYLDEDTIYHLQPSGRFVIGGPQGDAGVTGRKIIVDTYGGWGAHGGGAFSGKDYTKVDRSAAYAARWVAKSLVKAGLCRRVLVQVSYAIGVAEPLSISIFTYGTSKKTERELLEVVNKNFDLRPGVIVRDLDLKKPIYQKTACYGHFGRSEFPWEVPKKLVF

222222222222222222222222222222222222222222222222222222212222222222222212222222222222222222222222222222222222222222222222222222222222221222222222222222222222222222222222222222222222222222222222222222222222222222222222222222222222222222222222222222222222222222222222222222222222222222222122212122222222222222222222222222222222222222222222222222222222222222222222222222222222222222222222222222222222

>2W6ED

MLGLVGRVVAASASGALRGLSPSAPLPQAQLLLRAAPAALQPARDYAAQASPSPKAGATTGRIVAVIGAVVDVQFDEGLPPILNALEVQGRETRLVLEVAQHLGESTVRTIAMDGTEGLVRGQKVLDSGAPIRIPVGPETLGRIMNVIGEPIDERGPIKTKQFAAIHAEAPEFVEMSVEQEILVTGIKVVDLLAPYAKGGKIGLFGGAGVGKTVLIMELINNVAKAHGGYSVFAGVGERTREGNDLYHEMIESGVINLKDATSKVALVYGQMNEPPGARARVALTGLTVAEYFRDQEGQDVLLFIDNIFRFTQAGSEVSALLGRIPSAVGYQPTLATDMGTMQERITTTKKGSITSVQAIYVPADDLTDPAPATTFAHLDATTVLSRAIAELGIYPAVDPLDSTSRIMDPNIVGSEHYDVARGVQKILQDYKSLQDIIAILGMDELSEEDKLTVSRARKIQRFLSQPFQVAEVFTGHLGKLVPLKETIKGFQQILAGEYDHLPEQAFYMVGPIEEAVAKADKLAEEHS

222222222222222222222222222222222222222222222222222222222222222222222222222222222222222222222222222222222222222222222222222222222222222222222222222222222222222222222222222222222222222222222222222222222222222111111122222222222222222222222222222222222222222222222222222222222222222222222222222222222222222222222222222222222222222222222222222222222222222222222222222222222222222222222222222222222212222222222222222222222222222222222222222222222222222222222222222222222221221221122222222222222222222222222222222222222222222222222222

>1W44A

MIHLYDAKSFAKLRAAQYAAFHTDAPGSWFDHTSGVLESVEDGTPVLAIGVESGDAIVFDKNAQRIVAYKEKSVKAEDGSVSVVQVENGFMKQGHRGWLVDLTGELVGCSPVVAEFGGHRYASGMVIVTGKGNSGKTPLVHALGEALGGKDKYATVRFGEPLSGYNTDFNVFVDDIARAMLQHRVIVIDSLKNVIGAAGGNTTSGGISRGAFDLLSDIGAMAASRGCVVIASLNPTSNDDKIVELVKEASRSNSTSLVISTDVDGEWQVLTRTGEGLQRLTHTLQTSYGEHSVLTIHTSKQSGGKQASGKAIQTVIKNDELESVLRRLTSN

2222222222222222222222222222222222222222222222222222222222222222222222222222222222222222222222222222222222222222222222222222222222222211112222222222222222222222222222222222222222222222222222222222222222222222222222222222222222222222212222222222222222222222222222222222222122211122222222212221222222222222222221222222222222222222222

>2W5AA

MPSRAEDYEVLYTIGTGSYGRCQKIRRKSDGKILVWKELDYGSMTEAEKQMLVSEVNLLRELKHPNIVRYYDRIIDRTNTTLYIVMEYCEGGDLASVITKGTKERQYLDEEFVLRVMTQLTLALKECHRRSDGGHTVLHRDLKPANVFLDGKQNVKLGDFGLARILNHDTSFAKTFVGTPYYMSPEQMNRMSYNEKSDIWSLGCLLYELCALMPPFTAFSQKELAGKIREGKFRRIPYRYSDELNEIITRMLNLKDYHRPSVEEILENPLILEHHHHHH

222222222222212211222122222222222222122222222222222222222222222222212222222222222222221112221222222222222222222222222222222222222222222222222222222122222222222222212222222222222222222222222222222222222222222222222222222222222222222222222222222222222222222222222222222222222222222

>2HMFA

TTVMKFGGTSVGSGERIRHVAKIVTKRKKEDDDVVVVVSAMSEVTNALVEISQQALDVRDIAKVGDFIKFIREKHYKAIEEAIKSEEIKEEVKKIIDSRIEELEKVLIGVAYLGELTPKSRDYILSFGERLSSPILSGAIRDLGEKSIALEGGEAGIITDNNFGSARVKRLEVKERLLPLLKEGIIPVVTGFIGTTEEGYITTLGRGGSDYSAALIGYGLDADIIEIWTDVSGVYTTDPRLVPTARRIPKLSYIEAMELAYFGAKVLHPRTIEPAMEKGIPILVKNTFEPESEGTLITNDMEMSDSIVKAISTIKNVALINIFGAGMVGVSGTAARIFKALGEEEVNVILISQGSSETNISLVVSEEDVDKALKALKREFGDFGKKSFLNNNLIRDVSVDKDVCVISVVGAGMRGAKGIAGKIFTAVSESGANIKMIAQGSSEVNISFVIDEKDLLNCVRKLHEKFIEK

2222121122222222222222222222222222222222222222222222222222222222222222222222222222222222222222222222222222222222222222222222222222222222222222222222222222222222222222222222222222222222222222222222222222222222222222222222222222221112111211112222222222222222222222211122222222222222222222222222222222222222222222222222222222222222222222222222222222222222222222222222222222222222222222222222222222222222222222222222222222222222222222222222222222222222222222222222222222222

>2IF8A

MDTVNNYRVLEHKAAGHDGTLTDGDGLLIFKPAFPQELEFYKAIQVRDVSRRKSSADGDAPLCSWMPTYLGVLNEGAKIEQSGDAALLKIDERLSDSTDNLDSIPVKSEKSKQYLVLENLLYGFSKPNILDIKLGKTLYDSKASLEKRERMKRVSETTTSGSLGFRICGMKIQKNPSVLNQLSLEYYEEEADSDYIFINKLYGRSRTDQNVSDAIELYFNNPHLSDARKHQLKKTFLKRLQLFYNTMLEEEVRMISSSLLFIYEGDPERWELLNDVDKLMRDDFIDDDDDDDDNDDDDDDDAEGSSEGPKDKKTTGSLSSMSLIDFAHSEITPGKGYDENVIEGVETLLDIFMKFLEHHHHHH

222222222222222222222222222222122222222222222222222222222222222222122222222222222222222222222222222222222222222222222111222222222212222222222222222222222222222222222222222222222222222222222222222222222222222222222222222222222222222222222222222222222222222222222222222222222222222222222222222222222222222222222222222222222221122222222222222222222222222222222222222

>2VEDA

MRGSHHHHHHGSVIFDKRIKDEEDVEKELGLPVLGSIQKFNMTNTRRSTSSLIVHEQPKSPISEKFRGIRSNIMFANPDSAVQSIVITSEAPGAGMSTIAANLAVAYAQAGYKTLIVDGDMRKPTQHYIFNLPNNEGLSSLLLNWSTYQDSIISTEIEDLDVLTSGPIPPNPSELITSRAFANLYDTLLMNYNFVIIDTPPVNTVTDAQLFSKFTGNVVYVVNSENNNKDEVKKGKELIEATGAKLLGVVLNRMPKDKSASYYAYYGTDES

2222222222222222222222222222222222221111222222222212222222222222222222222222222222222222222111111122222222222222222222222222222222222222222222222222222222222222222222222222222222222222222222222222222222222222222222222222222222222222222222222222222222211222222222222222222

>1W5SA

MKVLRHGLFKDRRVFDENYIPPELRVRRGEAEALARIYLNRLLSGAGLSDVNMIYGSIGRVGIGKTTLAKFTVKRVSEAAAKEGLTVKQAYVNAFNAPNLYTILSLIVRQTGYPIQVRGAPALDILKALVDNLYVENHYLLVILDEFQSMLSSPRIAAEDLYTLLRVHEEIPSRDGVNRIGFLLVASDVRALSYMREKIPQVESQIGFKLHLPAYKSRELYTILEQRAELGLRDTVWEPRHLELISDVYGEDKGGDGSARRAIVALKMACEMAEAMGRDSLSEDLVRKAVSENEAASIQTHELEALSIHELIILRLIAEATLGGMEWINAGLLRQRYEDASLTMYNVKPRGYTQYHIYLKHLTSLGLVDAKPSGRGMRGRTTLFRLAPHLPADRLIEVVDNIIQAKMASGYE

2222222222222222221212212212222222222222222222222222222222221111111222222222222222222222222222222222222222222222222222222222222222222222222222222222222222222222222222222222222222222222222222222222222222222222222222122222221222122222222222222222222222222222221122222222222222222222222222222222222222222222222222222222222222222222222222222222222222222222222222222222222222222222222222222222222222222222222222222222

>1GKZA

STSATDTHHVELARERSKTVTSFYNQSAIDVVAEKPSVRLTPTMMLYSGRSQDGSHLLKSGRYLQQELPVRIAHRIKGFRSLPFIIGCNPTILHVHELYIRAFQKLTDFPPIKDQADEAQYCQLVRQLLDDHKDVVTLLAEGLRESRKHIEDEKLVRYFLDKTLTSRLGIRMLATHHLALHEDKPDFVGIICTRLSPKKIIEKWVDFARRLCEHKYGNAPRVRINGHVAARFPFIPMPLDYILPELLKNAMRATMESHLDTPYNVPDVVITIANNDVDLIIRISDRGGGIAHKDLDRVMDYHFTTAEASTQDPRISPLFGHLDMHSGGQSGPMHGFGFGLPTSRAYAEYLGGSLQLQSLQGIGTDVYLRLRHIDGREESFRIHHHHHH

2222222222222222222222222222222222222222222222222222222222222222222222222222222222222222222222222222222222222222222222222222222222222222222222222222222222222222222222222222222222222222222222222222222222222222222222222222222222222222222222222222222212211222222222222222222222222222222212221122222221222221122222222222222222222222222221111211122222222222222222222221222222222222222222222222

>2BVCA

MAHHHHHHGTEKTPDDVFKLAKDEKVEYVDVRFCDLPGIMQHFTIPASAFDKSVFDDGLAFDGSSIRGFQSIHESDMLLLPDPETARIDPFRAAKTLNINFFVHDPFTLEPYSRDPRNIARKAENYLISTGIADTAYFGAEAEFYIFDSVSFDSRANGSFYEVDAISGWWNTGAATEADGSPNRGYKVRHKGGYFPVAPNDQYVDLRDKMLTNLINSGFILEKGHHEVGSGGQAEINYQFNSLLHAADDMQLYKYIIKNTAWQNGKTVTFMPKPLFGDNGSGMHCHQSLWKDGAPLMYDETGYAGLSDTARHYIGGLLHHAPSLLAFTNPTVNSYKRLVPGYEAPINLVYSQRNRSACVRIPITGSNPKAKRLEFRSPDSSGNPYLAFSAMLMAGLDGIKNKIEPQAPVDKDLYELPPEEAASIPQTPTQLSDVIDRLEADHEYLTEGGVFTNDLIETWISFKRENEIEPVNIRPHPYEFALYYDV

222222222222222222222222222222222222222222222222222222222222222222222222222222222222222222222222222222222222222222222222222222222222222212121222222222222222222222222222222222222222222222222222222222222222222222222222222221122222222222121111222222222222222222222222222222222222222222222121222222222222222222222222222222222222222222222222222222222222222222122221222222221221212222222222222222222222222222222222222222222222222222222222222222222222222222222222222222222222222222222222222222

>2WQNA

MDEQSQGMQGPPVPQFQPQKALRPDMGYNTLANFRIEKKIGRGQFSEVYRAACLLDGVPVALKKVQIFDLMDAKARADCIKEIDLLKQLNHPNVIKYYASFIEDNELNIVLELADAGDLSRMIKHFKKQKRLIPERTVWKYFVQLCSALEHMHSRRVMHRDIKPANVFITATGVVKLGDLGLGRFFSSKTTAAHSLVGTPYYMSPERIHENGYNFKSDIWSLGCLLYEMAALQSPFYGDKMNLYSLCKKIEQCDYPPLPSDHYSEELRQLVNMCINPDPEKRPDVTYVYDVAKRMHACTASSLEHHHHHH

2222222222222222222222222222222222222221221111212222222222221212222222222222222222222222222222222222222222222211212222222222222222222222222222222222222222222222222222222222222222122222222222222222222222222222222222222222222222222222222222222222222222222222222222222222222222222222222222222222222222222222222222

>1U0JA

GMELVGWLVDKGITSEKQWIQEDQASYISFNAASNSRSQIKAALDNAGKIMSLTKTAPDYLVGQQPVEDISSNRIYKILELNGYDPQYAASVFLGWATKKFGKRNTIWLFGPATTGKTNIAEAIAHTVPFYGCVNWTNENFPFNDCVDKMVIWWEEGKMTAKVVESAKAILGGSKVRVDQKCKSSAQIDPTPVIVTSNTNMCAVIDGNSTTFEHQQPLQDRMFKFELTRRLDHDFGKVTKQEVKDFFRWAKDHVVEVEHEFYVKKGG

222222222222222222222222222222222222222222222222222222222222222222222222222222222222222222222222222222222222222211111112222222222222222222222222222222222222222222222222222222222222222222222222222222222222222222222222222222222222221112111222222222222222222222222222222

>2C31A

MSNDDNVELTDGFHVLIDALKMNDIDTMYGVVGIPITNLARMWQDDGQRFYSFRHEQHAGYAASIAGYIEGKPGVCLTVSAPGFLNGVTSLAHATTNCFPMILLSGSSEREIVDLQQGDYEEMDQMNVARPHCKASFRINSIKDIPIGIARAVRTAVSGRPGGVYVDLPAKLFGQTISVEEANKLLFKPIDPAPAQIPAEDAIARAADLIKNAKRPVIMLGKGAAYAQCDDEIRALVEETGIPFLPMGMAKGLLPDNHPQSAAATRAFALAQCDVCVLIGARLNWLMQHGKGKTWGDELKKYVQIDIQANEMDSNQPIAAPVVGDIKSAVSLLRKALKGAPKADAEWTGALKAKVDGNKAKLAGKMTAETPSGMMNYSNSLGVVRDFMLANPDISLVNEGANALDNTRMIVDMLKPRKRLDSGTWGVMGIGMGYCVAAAAVTGKPVIAVEGDSAFGFSGMELETICRYNLPVTVIIMNNGGIYKGNEADPQPGVISCTRLTRGRYDMMMEAFGGKGYVANTPAELKAALEEAVASGKPCLINAMIDPDAGVESGRIKSLNVVSKVGKK

2222222222222222222222222222222222222222222222222222222222222222222222222222222222222222222222222122222222222222222222222222222222222222222222222222222222222221222222222222222222222222222222222222222222222222222222222222111222222222222222222222221222222222222222222222222222222221112121222222222222222222211222222222222222211122222222222222222222222222222222222222222222222222222222222222222222222222222222222222222222222222222222222222222222222222222222222222222222222222222222222222222222222222222222222222222222222222222222222222222222222222222222222222222222222222

>1NQTA

DPNFFKMVEGFFDRGASIVEDKLVEDLRTRESEEQKRNRVRGILRIIKPCNHVLSLSFPIRRDDGSWEVIEGYRAQHSHQRTPCKGGIRYSTDVSVDEVKALASLMTYKCAVVDVPFGGAKAGVKINPKNYTDNELEKITRRFTMELAKKGFIGPGIDVPAPDMSTGEREMSWIADTYASTIGHYDINAHACVTGKPISQGGIHGRISATGRGVFHGIENFINEASYMSILGMTPGFGDKTFVVQGFGNVGLHSMRYLHRFGAKCIAVGESDGSIWNPDGIDPKELEDFKLQHGSILGFPKAKPYEGSILEADCDILIPAASEKQLTKSNAPRVKAKIIAEGANGPTTPEADKIFLERNIMVIPDLYLNAGGVTVSYFEWLKNLNHVSYGRLTFKYERDSNYHLLMSVQESLERKFGKHGGTIPIVPTAEFQDRISGASEKDIVHSGLAYTMERSARQIMRTAMKYNLGLDLRTAAYVNAIEKVFKVYNEAGVTFT

2222222222222222222222222222222222222222222222222222222222222222222222222222222112222222222222222222222222222212211212222222222222222222222222222222222222222222222222222222222222222222222222222222212222212222222222222222222222222222222222222222222222222222222222222222222222222222222222222222222222222222222222222222222222222222222222222222222222222222222222222222222222222222222221222221221222222222222222222222222222222222222222222222222222222222222221222222222222222222222222222211221222222222

>2V2ZA

GSHMIKVLSPAKINLGLWVLGRLPSGYHEILTLYQEIPFYDEIYIREGVLRVETNIGIPQEENLVYKGLREFERITGIEINYSIFIQKNIPPGAGLGGGSSNLAVVLKKVNELLGSPLSEEELRELVGSISADAPFFLLGKSAIGRGKGEVLEPVETEISGKITLVIPQVSSSTGRVYSSLREEHFVTPEYAEEKIQRIISGEVEEIENVLGDIARELYPEINEVYRFVEYLGFKPFVSGSGSTVYFFGGASEELKKAAKMRGWKVVELEL

2222222222222222222222222222222222222222222222222222222221222111222222222222222222222221221111111111212222222222222222222222222222222222222222222222222222222222222222222222212222222222222222222222222222222222222222222222222222222222222222222222222222222222222222222222222

>1OSNA

MSTDKTDVKMGVLRIYLDGAYGIGKTTAAEEFLHHFAITPNRILLIGEPLSYWRNLAGEDAICGIYGTQTRRLNGDVSPEDAQRLTAHFQSLFCSPHAIMHAKISALMDTSTSDLVQVNKEPYKIMLSDRHPIASTICFPLSRYLVGDMSPAALPGLLFTLPAEPPGTNLVVCTVSLPSHLSRVSKRARPGETVNLPFVMVLRNVYIMLINTIIFLKTNNWHAGWNTLSFCNDVFKQKLQKSECIKLREVPGIEDTLFAVLKLPELCGEFGNILPLWAWGMETLSNCLRSMSPFVLSLEQTPQHAAQELKTLLPQMTPANMSSGAWNILKELVNAVQDNTS

22222222222222222222111111122222222222222222222222222222222222222222222222222222222222222222222222222222222222222222222222222222222222222222222222222222222222222222222222222222222222222222222222222222222222222222222222222222222222222222222222222222222222222222222222222222222222222222222222222222222222222222222222222222222222222222222222222

>3BRBA

MGSSHHHHHHSSGLVPRGSEELQNKLEDVVIDRNLLILGKILGEGEFGSVMEGNLKQEDGTSLKVAVKTMKLDNSSQREIEEFLSEAACMKDFSHPNVIRLLGVCIEMSSQGIPKPMVILPFMKYGDLHTYLLYSRLETGPKHIPLQTLLKFMVDIALGMEYLSNRNFLHRDLAARNCMLRDDMTVCVADFGLSKKIYSGDYYRQGRIAKMPVKWIAIESLADRVYTSKSDVWAFGVTMWEIATRGMTPYPGVQNHEMYDYLLHGHRLKQPEDCLDELYEIMYSCWRTDPLDRPTFSVLRLQLEKLLESLPDV

2222222222222222222222222222222222222222211222222122222222222222212122222222222222222222222222222212222222222222222222221112221222222222222222222222222222222222222222222222222112122222222221222222222222222222222222222222222222222222222222222222222222222222222222222222222222222222222222222222222222222222222222222

>2EWVA

MFEKQEVEQKKELKILEIIKEAIELGASDIHLTAGAPPAVRIDGYIKFLKDFPRLTPEDTQKLAYSVMSEKHRQKLEENGQVDFSFGVRGVGRFRANVFYQRGSVAAALRSLPAEIPEFKKLGLPDKVLELCHRKMGLILVTGPTGSGKSTTIASMIDYINQTKSYHIITIEDPIEYVFKHKKSIVNQREVGEDTKSFADALRAALREDPDVIFVGEMRDLETVETALRAAETGHLVFGTLHTNTAIDTIHRIVDIFPLNQQEQVRIVLSFILQGIISQRLLPKIGGGRVLAYELLIPNTAIRNLIRENKLQQVYSLMQSGQAETGMQTMNQTLYKLYKQGLITLEDAMEASPDPKELERMIRGGRHHHHHH

222222222222222222222222222222222222222222222222222222222222222222222222222222222222222222222222222222222222222222222222212222222222222222222222211111122222222222222222222222222222222222222222222222222222222222222222222222222222222222222222222222222222222222222222222222222222222212222222121222222222222222222222222222222222222222222222222222222222222222222222222222222222

>1HTWA

MESLTQYIPDEFSMLRFGKKFAEILLKLHTEKAIMVYLNGDLGAGKTTLTRGMLQGIGHQGNVKSPTYTLVEEYNIAGKMIYHFDLYRLADPEELEFMGIRDYFNTDSICLIEWSEKGQGILPEADILVNIDYYDDARNIELIAQTNLGKNIISAFSN

22222222211221222222222222222222222222221111111122222222222222222222222222222222222222222222222222222222222222221222222222222222222222212122222222222222222222

>3EPQA

SNAULGGIEAGGTUFVCAVGREDGTIIDRIEFPTUUPDETIEUVIQYFSQFSLQAIGIGSFGPVDNDUTSQTYGTITATPUAGWRHYPFLQTVUNEUUIPVGFSTDVNAAALGEFLFGEAUGLDSCLYITIGTGIGAGAIVEGRLLQGLSHPEUGHIYIRRHPDDVYQGUCPYHGDCFEGLASGPAIEARWGUKAADLSDIAQVWELEGYYIAQALAQYILILAPUUIILGGGVUQQUQVFSYIYQYVPKIUNSYLDFSELSDDISDYIVPPRLGSNAGIIGTLVLAHQALQAEAASGEVRS

22222222222222222222222222222222222222222222222222222222222222222222222222222222222222222222222222222222222222222222222222222222222112222222222222222222222222222222222222222222222222211222222222112222222222222222222222222222222222211122222222222222222222222222222222222222222222222222222222222222222222

>2VF7A

MTPSRPSPDFPDGGFVQVRGARQHNLKDISVKVPRDALVVFTGVSGSGKSSLAFGTLYAEAQRRYLESVSPYARRLFNQAGVPDVDAIDGLPPAVALQQARGTPTARSSVGSVTTLSNLLRMLYSRAGDYPPGQGIVYAEGFSPNTPEGACPECHGLGRVYTVTEDSMVPDPSLTIRERAVAAWPQAWGGQNQRDILVTLGIDVDVPWRELPEETRHWILFTDEQPVVPVYPGLTPAETQRALKKKMEPSYMGTFSSARRHVLHTFANTESASMKKRVQGYMISEECPLCHGKRLRQEALNVTFAGLDITELSRLPLARVSELLRPYAEEREPGHAERVKNRPEQAIALQRMAADLVKRLDVLLHLGLGYLGLDRSTPTLSPGELQRLRLATQLYSNLFGVVYVLDEPSAGLHPADTEALLSALENLKRGGNSLFVVEHDLDVIRRADWLVDVGPEAGEKGGEILYSGPPEGLKHVPESQTGQYLFADRHTEPHTPREPAGWLELNGVTRNNLDNLDVRFPLGVMTSVTGVSGSGKSTLVSQALVDALAAHFGQPVNPDPEDDEDPADHTAGSARLGGDLAQITRLVRVDQKPIGRTPRSNMATYTGLFDQVRKLFAATPLAKKRGYNAGRFSFNVKGGRCEHCQGEGWVMVELLFLPSVYAPCPVCHGTRYNAETLEVEYRGKNIADVLALTVDEAHDFFADESAIFRALDTLREVGLGYLRLGQPATELSGGEAQRIKLATELRRSGRGGTVYVLDEPTTGLHPADVERLQRQLVKLVDAGNTVIAVEHKMQVVAASDWVLDIGPGAGEDGGRLVAQGTPAEVAQAAGSVTAPYLRAALR

22222222222222222222222112222222222222222221111111122222222222222222222222222222222222222222222222221222222222222222222222222222222222222222222222222222222222222222222222222222222222222222222222222222222222222222222222222222222222222222222222222222222222222222222222222222222222222222222222222222222222222222222222222222222222222222222222222222222222222222222222222222212222122222222222222222222222222222222222222222222222222222222222222222222222222222222222222222222222222222222222222222222222222222222222222211222222222222222222111111112221222222222222222222222222222222222222222222222222222222222222222222222222222222222222222222222222222222222222222222222222222222222222222222222222222222222222222222222222222222222222222122212222222222222222222222222222222222222222222222222222222222222222222222222222222122222222222222222222222222222222

>2DCNA

MAKLITLGEILIEFNALSPGPLRHVSYFEKHVAGSEANYCVAFIKQGNECGIIAKVGDDEFGYNAIEWLRGQGVDVSHMKIDPSAPTGIFFIQRHYPVPLKSESIYYRKGSAGSKLSPEDVDEEYVKSADLVHSSGITLAISSTAKEAVYKAFEIASNRSFDTNIRLKLWSAEEAKREILKLLSKFHLKFLITDTDDSKIILGESDPDKAAKAFSDYAEIIVMKLGPKGAIVYYDGKKYYSSGYQVPVEDVTGAGDALGGTFLSLYYKGFEMEKALDYAIVASTLNVMIRGDQENLPTTKDIETFLREMKK

22222222222222222222222222222222222222222222222222222222222222222222222222222222222222222222222222222222222222222222222222222222222222222222222222222222222222222222222222222222222222222222222221222222222222222222222222222221211212222222222222212221221221122122222222222222222222212212221222222222222222222222222

>3H4SA

RKRYYNTIEDMKGKIRVYCRIRPLNEKESSEREKQMLTTVDEFTVEHPWKDDKRKQHIYDRVFDMRASQDDIFEDTKYLVQSAVDGYNVCIFAYGQTGSGKTFTIYGHESNPGLTPRATKELFNILKRDSKRFSFSLKAYMVELYQDTLVDLLLPKSARRLKLEIKKDSKGMVFVENVTTIPISTLEELRMILERGSERRHVSGTNMNEESSRSHLILSVVIESIDLQTQSAARGKLSFVDLAGSERVKKSGSAGNQLKEAQSINKSLSALGDVIGALSSGNQHIPYRNHKLTMLMSDSLGGNAKTLMFVNVSPAESNLDETYNSLLYASRVRTIVNDPSKHISSKEMVRLKKLVAYWKEQAGKKGEEEDLVDIEEDRTRKDEADS

22222222222222222221211222222222222222222222222222222222222222222222222222222222222222222222222111111112222222222222222222222222222222222222222222222222222222222222222222222222222222222222222222222222222222222222222222222222222222222222222222222222222222222222222222222222222222222222222222222222222222222222222222222222222222222222222222222222222222222222222222222222222222222222222222

>1GKIA

LNSVGQGEFGGAPFKRFLRGTRIVSGGKLKRMTREKAKQVTVAGVPMPRDAEPRHLLVNGATGTGKSVLLRELAYTGLLRGDRMVIVDPNGDMLSKFGRDKDIILNPYDQRTKGWSFFNEIRNDYDWQRYALSVVPRGKTDEAEEWASYGRLLLRETAKKLALIGTPSMRELFHWTTIATFDDLRGFLEGTLAESLFAGSNEASKALTSARFVLSDKLPEHVTMPDGDFSIRSWLEDPNGGNLFITWREDMGPALRPLISAWVDVVCTSILSLPEEPKRRLWLFIDELASLEKLASLADALTKGRKAGLRVVAGLQSTSQLDDVYGVKEAQTLRASFRSLVVLGGSRTDPKTNEDMSLSLGEHEVERDRYSKNTGKHHSTGRALERVRERVVMPAEIANLPDLTAYVGFAGNRPIAKVPLEIKQFANRQPAFVEGTI

22222222222222222222222222222222222222222222222222222122222212111111222222222222222222222222222222222222222222222222222222222222222222222222222222222222222222222222222222222222222222222222222222222222222222222222222222222222222222222222222222222222222222222222222222222222222222222222222222222222222222221222222222222222222222222222222222222222222222222222222222222222222222222222222222222222222222222222222222222222222221222222222222222

>1DJNA

ARDPKHDILFEPIQIGPKTLRNRFYQVPHCIGAGSDKPGFQSAHRSVKAEGGWAALNTEYCSINPESDDTHRLSARIWDEGDVRNLKAMTDEVHKYGALAGVELWYGGAHAPNMESRATPRGPSQYASEFETLSYCKEMDLSDIAQVQQFYVDAAKRSRDAGFDIVYVYGAHSYLPLQFLNPYYNKRTDKYGGSLENRARFWLETLEKVKHAVGSDCAIATRFGVDTVYGPGQIEAEVDGQKFVEMADSLVDMWDITIGDIAEWGEDAGPSRFYQQGHTIPWVKLVKQVSKKPVLGVGRYTDPEKMIEIVTKGYADIIGCARPSIADPFLPQKVEQGRYDDIRVCIGCNVCISRWEIGGPPMICTQNATAGEEYRRGWHPEKFRQTKNKDSVLIVGAGPSGSEAARVLMESGYTVHLTDTAEKIGGHLNQVAALPGLGEWSYHRDYRETQITKLLKKNKESQLALGQKPMTADDVLQYGADKVIIATGARWNTDGTNCLTHDPIPGADASLPDQLTPEQVMDGKKKIGKRVVILNADTYFMAPSLAEKLATAGHEVTIVSGVHLANYMHFTLEYPNMMRRLHELHVEELGDHFCSRIEPGRMEIYNIWGDGSKRTYRGPGVSPRDANTSHRWIEFDSLVLVTGRHSECTLWNELKARESEWAENDIKGIYLIGDAEAPRLIADATFTGHRVAREIEEANPQIAIPYKRETIAWGTPHMPGGNFKIEYKV

222222222222222222222222222222222222222222222222222222222222222222222222222222222222222222222222222222222222222222222222222222222222222222222222222222222222222222222222222222222222222222222222222222222222222222222222222222222222222222222222222222222222222222222222222222222222222222222222222222222222222222222222222222222222222222222222222222222222222222222222222222222222222222222222222222222211211122222222222222222211222221122222222222222222222222222222222222222222112222222222222221111222222222222222222222222222222222222222222222222222222222222222222222222222222222222222222222222222222222222222222222222222222222222222222222222222222222222222222222222222222222222222112222222222222222222222222222222222222222222222222222222

>1HW8B

GAMASSVLVTQEPEIELPREPRPNEECLQILGNAEKGAKFLSDAEIIQLVNAKHIPAYKLETLIETHERGVSIRRQLLSKKLSEPSSLQYLPYRDYNYSLVMGACCENVIGYMPIPVGVAGPLCLDEKEFQVPMATTEGCLVASTNRGCRAIGLGGGASSRVLADGMTRGPVVRLPRACDSAEVKAWLETSEGFAVIKEAFDSTSRFARLQKLHTSIAGRNLYIRFQSRSGDAMGMNMISKGTEKALSKLHEYFPEMQILAVSGNYCTDKKPAAINWIEGRGKSVVCEAVIPAKVVREVLKTTTEAMIEVNINKNLVGSAMAGSIGGYNAHAANIVTAIYIACGQDAAQNVGSSNCITLMEASGPTNEDLYISCTMPSIEIGTVGGGTNLLPQQACLQMLGVQGACKDNPGENARQLARIVCGTVMAGELSLMAALAAGHLVKSHMIHNRSKINLQDLQGACTKKTA

22222222222222222222222222222222222222222222222222222222222222222222222222222222222222222222222222222222222222222222222222222222222222222222221221122222222222222222222222222222222222222222222222222222222222222222222222222222222222222222222222222222222222222222222222222222222222222222222222222222222212222222222222222222222222222222222222222222222222222222222222222222222222222222222222222222222222222222222222222222222222222222222222222222222222222222222222222222222

>1WBPA

PEQEEEILGSDDDEQEDPNDYCKGGYHLVKIGDLFNGRYHVIRKLGWGHFSTVWLSWDIQGKKFVAMKVVKSAEHYTETALDEIRLLKSVRNSDPNDPNREMVVQLLDDFKISGVNGTHICMVFEVLGHHLLKWIIKSNYQGLPLPCVKKIIQQVLQGLDYLHTKCRIIHTDIKPENILLSVNEQYIRRLAAEATEWQRSGAPPPSGSAVSTAPATAGNFLVNPLEPKNAEKLKVKIADLGNACWVHKHFTEDIQTRQYRSLEVLIGSGYNTPADIWSTACMAFELATGDYLFEPHSGEEYTRDEDHIALIIELLGKVPRKLIVAGKYSKEFFTKKGDLKHITKLKPWGLFEVLVEKYEWSQEEAAGFTDFLLPMLELIPEKRATAAECLRHPWLNS

2222222222222222222222222222222222222222222211112112122222222222212122222222222222222222222222222222222222222222222222222222121222222222222222222222222222222222222222222222222222222222222222222222222222222222222222222222222222222222222222222222222222222222222222222222222222222222222222222222222222222222222222222222222222222222222222222222222222222222222222222222222222222222222222222222222222222

>2CVXA

MYVYKRDGRKEPVQFDKITARISRLCYGLDPKHIDAVKVTQRIISGVYEGVTTIELDNLAAETCAYMTTVHPDYATLAARIAISNLHKQTTKQFSKVVEDLYRYVNAATGKPAPMISDDVYNIVMENKDKLNSAIVYDRDFQYSYFGFKTLERSYLLRINGQVAERPQHLIMRVALGIHGRDIEAALETYNLMSLKYFTHASPTLFNAGTPKPQMSSCFLVAMKEDSIEGIYDTLKECALISKTAGGIGLHIHNIRSTGSYIAGTNGTSNGLIPMIRVFNNTARYVDQGGNKRPGAFALYLEPWHADIFDFIDIRKNHGKEEIRARDLFPALWIPDLFMKRVEENGTWTLFSPTSAPGLSDCYGDEFEALYTRYEKEGRGKTIKAQKLWYSILEAQTETGTPFVVYKDACNRKSNQKNLGVIKSSNLCCEIVEYSAPDETAVCNLASVALPAFIETSEDGKTSTYNFKKLHEIAKVVTRNLNRVIDRNYYPVEEARKSNMRHRPIALGVQGLADTFMLLRLPFDSEEARLLNIQIFETIYHASMEASCELAQKDGPYETFQGSPASQGILQFDMWDQKPYGMWDWDTLRKDIMKHGVRNSLTMAPMPTASTSQILGYNECFEPVTSNMYSRRVLSGEFQVVNPYLLRDLVDLGIWDEGMKQYLITQNGSIQGLPNVPQELKDLYKTVWEISQKTIINMAADRSVYIDQSHSLNLFLRAPTMGKLTSMHFYGWKKGLKTGMYYLRTQAASAAIQFTIDQKIADQATENVADISNLKRPSYMPSSASYAASDFVPAAVTANATIPSLDSSSEASREASPAPTGSHSLTKGMAELNVQESKVEVPEVPAPTKNEEKAAPIVDDEETEFDIYNSKVIACAIDNPEACEMCSG

222222222222222222222222222222222222222222222222222222222222222222222222222222222222222222222222222222222222222222222222222222222222222222222222222222222222222222222222222222222222222222222222222222221122222222222222112222222222222222222222222221122222222222222222222222222222222222222221222212212222222222222222222222222222222222222222222222222222222222222222222222222222222222222222222222222222222222222222222222222222222221112122222222222222122222222222222222222222222222222222222222222222222222222222222222222222222222222222222222222222222222222222222222222222222222222222222222222222222222222222222221111112222222222222222222222222222222222222222222222222222222222222222222222222222222222222222222222222222222222222222222222222222222222222222222222222222222222222222222222222222222222222222222222222222222222222222222222222222222222222222222222222222222222222222222222222222222222222

>1IQPA

MSEEIREVKVLEKPWVEKYRPQRLDDIVGQEHIVKRLKHYVKTGSMPHLLFAGPPGVGKTTAALALARELFGENWRHNFLELNASDERGINVIREKVKEFARTKPIGGASFKIIFLDEADALTQDAQQALRRTMEMFSSNVRFILSCNYSSKIIEPIQSRCAIFRFRPLRDEDIAKRLRYIAENEGLELTEEGLQAILYIAEGDMRRAINILQAAAALDKKITDENVFMVASRARPEDIREMMLLALKGNFLKAREKLREILLKQGLSGEDVLVQMHKEVFNLPIEEPKKVLLADKIGEYNFRLVEGANEIIQLEALLAQFTLIGKK

222222222222222222211222221122222222222222222222222222111111122222222222222222222222222222222222222222222222222222222222222222222222222222222222222222222222222222222222222222222222222222222222222222222222122222222222222222222222222222222222222222222222222222222222222222222222222222222222222222222222222222222222222222222222222

>1UM8A

EEEFLLSYIPAPKELKAVLDNYVIGQEQAKKVFSVAVYNHYKRLSFKEKLKKQDNQDSNVELEHLEEVELSKSNILLIGPTGSGKTLMAQTLAKHLDIPIAISDATSLTEAGYVGEDVENILTRLLQASDWNVQKAQKGIVFIDEIDKISRLSENRSITRDVSGEGVQQALLKIVEGSLVNIPPKGGRKHPEGNFIQIDTSDILFICAGAFDGLAEIIKKRTTQNVLGFTQEKMSKKEQEAILHLVQTHDLVTYGLIPELIGRLPVLSTLDSISLEAMVDILQKPKNALIKQYQQLFKMDEVDLIFEEEAIKEIAQLALERKTGARGLRAIIEDFCLDIMFDLPKLKGSEVRITKDCVLKQAEPLIIAKTHSKILP

2222222222222222222221112222222222222222222222222222222222222222222222222222222211111112222222222222222222222222222222222222222222222222222222222222222222222222222222222222222222222222222222222222222222222222222222222222222222222222222222222222222222222222222222222222222222222222122222222222222222222222222222222222222222221122222222222222222222222222222222222222222222222222

>2V1UA

LESKIFRKRWVLLPDYVPDVLPHREAELRRLAEVLAPALRGEKPSNALLYGLTGTGKTAVARLVLRRLEARASSLGVLVKPIYVNARHRETPYRVASAIAEAVGVRVPFTGLSVGEVYERLVKRLSRLRGIYIIVLDEIDFLPKRPGGQDLLYRITRINQELGDRVWVSLVGITNSLGFVENLEPRVKSSLGEVELVFPPYTAPQLRDILETRAEEAFNPGVLDPDVVPLCAALAAREHGDARRALDLLRVAGEIAERRREERVRREHVYSARAEIERDRVSEVVRTLPLHAKLVLLSIMMLEDGGRPASTGEIYERYKELTSTLGLEHVTLRRVSGIISELDMLGIVKSRVVSRGRYGKTREVSLDADRLAVENALSEDPFVARLL

222222222222222122221121222222222222222222222222222121111112222222222222222222222222222222222222222222222222222222222222222222222222222222222222222222222222222222222222222222222222222222222222222222221222222212221222222222222222222222222222211221222222222222222222222222222222222222222222222222222222222222222222222222222222222222222222222222222222222222222222222222222222222222222222222

>1X3MA

MRGSHHHHHHGMASNEFPVVLVINCGSSSIKFSVLDVATCDVLMAGIADGMNTENAFLSINGDKPINLAHSNYEDALKAIAFELEKRDLTDSVALIGHRIAHGGELFTQSVIITDEIIDNIRRVSPLAPLHNYANLSGIDAARHLFPAVRQVAVFDTSFHQTLAPEAYLYGLPWEYFSSLGVRRYGFHGTSHRYVSRRAYELLDLDEKDSGLIVAHLGNGASICAVRNGQSVDTSMGMTPLEGLMMGTRSGDVDFGAMAWIAKETGQTLSDLERVVNKESGLLGISGLSSDLRVLEKAWHEGHERARLAIKTFVHRIARHIAGHAASLHRLDGIIFTGGIGENSVLIRQLVIEHLGVLGLTLDVEMNKQPNSHGERIISANPSQVICAVIPTNEEKMIALDAIHLGNVKAPVEFA

2222222222222222222222222222222222222222222222222222222222222222222222222222222222222222222222222222222222222222222222222222222222222222222222222222222222222222222222222222222222222222222222222222222222222222222222212112222222222222222222222222222222222222222222222222222222222222222222222111122122222222222222222222222222222222222222222111221122222222222222222222222222222222222222222222222122222222222222222222222

>1Q8YA

DYRPGGYHPAFKGEPYKDARYILVRKLGWGHFSTVWLAKDMVNNTHVAMKIVRGDKVYTEAAEDEIKLLQRVNDADNTKEDSMGANHILKLLDHFNHKGPNGVHVVMVFEVLGENLLALIKKYEHRGIPLIYVKQISKQLLLGLDYMHRRCGIIHTDIKPENVLMEIVDSPENLIQIKIADLGNACWYDEHYTNSIQTREYRSPEVLLGAPWGCGADIWSTACLIFELITGDFLFEPDEGHSYTKDDDHIAQIIELLGELPSYLLRNGKYTRTFFNSRGLLRNISKLKFWPLEDVLTEKYKFSKDEAKEISDFLSPMLQLDPRKRADAGGLVNHPWLKDTLGMEEIRVPDRELYGSGSDIPGWFEEVRDHKRH

2222222222222222222222222221111112122222222222212122222222222222222222222222222222222222122222222222222222222111121222222222222222222222222222222222222222222222112122222222222222221222222222222222222222222222222222222222222222222222222222222222222222222222222222222222222222222222222222222222222222222222222222222222222222222222222222222222222222222222222222222222222222222

>1L8QA

KDFLNPKYTLENFIVGEGNRLAYEVVKEALENLGSLYNPIFIYGSVGTGKTHLLQAAGNEAKKRGYRVIYSSADDFAQAUVEHLKKGTINEFRNUYKSVDLLLLDDVQFLSGKERTQIEFFHIFNTLYLLEKQIILASDRHPQKLDGVSDRLVSRFEGGILVEIELDNKTRFKIIKEKLKEFNLELRKEVIDYLLENTKNVREIEGKIKLIKLKGFEGLERKERKERDKLUQIVEFVANYYAVKVEDILSDKRNKRTSEARKIAUYLCRKVCSASLIEIARAFKRKDHTTVIHAIRSVEEEKKKDRKFKHLVGFLEKQAFDKIC

222222222221112222122222222222222222222222221111111122222222222222222222222222222222222222222222222222222222222222222222222222222222222222222222222222222222222222222122222221222222222222222222222222221222222222222222222222222222222222222222222222222222222222222222222222222222222222222222222222222222222222222222222222222222

>3BK7A

MASWSHPQFEKGAHMVRKMRIAVIDYDKCNPDKCGHFLCERVCPVNRMGGEAIIIDEENYKPIIQEASCTGCGICVHKCPFNAISIVNLPEQLDEDCVHRYGVNAFVLYRLPIVKDGMVVGIVGPNGTGKTTAVKILAGQLIPNLCEDNDSWDNVIRAFRGNELQNYFERLKNGEIRPVVKPQYVDLLPKAVKGKVRELLKKVDEVGKFEEVVKELELENVLDRELHQLSGGELQRVAIAAALLRKAHFYFFDEPSSYLDIRQRLKVARVIRRLANEGKAVLVVEHDLAVLDYLSDVIHVVYGEPGVYGIFSKPKGTRNGINEFLQGYLKDENVRFRPYEIRFTKLSERVDVERETLVEYPRLVKDYGSFKLEVEPGEIRKGEVIGIVGPNGIGKTTFVKMLAGVEEPTEGKVEWDLTVAYKPQYIKAEYEGTVYELLSKIDSSKLNSNFYKTELLKPLGIIDLYDRNVEDLSGGELQRVAIAATLLRDADIYLLDEPSAYLDVEQRLAVSRAIRHLMEKNEKTALVVEHDVLMIDYVSDRLIVFEGEPGRHGRALPPMGMREGMNRFLASVGITFRRDPDSGRPRANKEGSVKDREQKARGEYYYA

2222222222222222222222222222222222222222222222222222222222222222222222222222222222222222222222222222122221222222222222222222111111112222222222222222222222222222222222222222222222222222222222222222222222222222222222222222222222222222222222222222222222222222222222222222222222222222222222222222222222222222212222222222222222222222222222222222222222222222222222222222221211222222222222222222211111111222222222222222222222222222222222222222222222222222222222222222222222222222222222222222222222222222222222222222222222222222222222222222222222222222222221222222222222222222222222222222222222222222222222222222222

>2GR0A

MSQEALKAPVVVLGAGLASVSFVAELRQAGYQGLITVVGDEAERPYDRPPLSKDFMAHGDAEKIRLDCKRAPEVEWLLGVTAQSFDPQAHTVALSDGRTLPYGTLVLATGAAPRALPTLQGATMPVHTLRTLEDARRIQAGLRPQSRLLIVGGGVIGLELAATARTAGVHVSLVETQPRLMSRAAPATLADFVARYHAAQGVDLRFERSVTGSVDGVVLLDDGTRIAADMVVVGIGVLANDALARAAGLACDDGIFVDAYGRTTCPDVYALGDVTRQRNPLSGRFERIETWSNAQNQGIAVARHLVDPTAPGYAELPWYWSDQGALRIQVAGLASGDEEIVRGEVSLDAPKFTLIELQKGRIVGATCVNNARDFAPLRRLLAVGAKPDRAALADPATDLRKLAAAVAA

222222222222222222222222222222222222222222222222222222222222222222222222222222222222222222222222222222222222222221212222222222222222222222222222222222111111222222222222222221111222211222222222222222222222222222222222222222222222222221112222222222222222222222222222222222222222222222222222222222222222222222222222222222222222222222222222222222222222222222222222222222222222222222222222222222222222222222222222

>2O0JA

MGSSHHHHHHSSGLVPRGSHMLEDPMEQPINVLNDFHPLNEAGKILIKHPSLAERKDEDGIHWIKSQWDGKWYPEKFSDYLRLHKIVKIPNNSDKPELFQTYKDKNNKRSRYMGLPNLKRANIKTQWTREMVEEWKKCRDDIVYFAETYCAITHIDYGVIKVQLRDYQRDMLKIMSSKRMTVCNLSRQLGKTTVVAIFLAHFVCFNKDKAVGILAHKGSMSAEVLDRTKQAIELLPDFLQPGIVEWNKGSIELDNGSSIGAYASSPDAVRGNSFAMIYIEDCAFIPNFHDSWLAIQPVISSGRRSKIIITTTPNGLNHFYDIWTAAVEGKSGFEPYTAIWNSVKERLYNDEDIFDDGWQWSIQTINGSSLAQFRQEHTAAFEGTS

2222222222222222222222222222222222222222222222222222222222222222222222222222222222222222222222222222222222222222222222222222222222222222222222222222222222222222211112212222222222222222222111111222222222222222222222222222222222122222222222222222222222222222222222222222222222222222222222222222222222222222222222222222222222222222222222222222222222222222222222222222222222222222222222222

>1RFUA

MEEECRVLSIQSHVVRGYVGNRAATFPLQVLGFEVDAVNSVQFSNHTGYSHWKGQVLNSDELQELYDGLKLNHVNQYDYVLTGYTRDKSFLAMVVDIVQELKQQNPRLVYVCDPVMGDQRNGEGAMYVPDDLLPVYREKVVPVADIITPNQFEAELLTGRKIHSQEEALEVMDMLHSMGPDTVVITSSNLLSPRGSDYLMALGSQRTRAPDGSVVTQRIRMEMHKVDAVFVGTGDLFAAMLLAWTHKHPNNLKVACEKTVSAMHHVLQRTIKCAKAKSGEGVKPSPAQLELRMVQSKKDIESPEIVVQATVL

222222222222222222222222222222222222222222222222222222222222222222222222222222222222222222222222222222222222222222222222222222222222222222222222222221222222222222222222222222222222222221122222222222122222222222222222222222111121212211221222222222222222222222212212221222222222222222222222222222222222222222222222

>2OJWA

MHHHHHHSSGVDLGTENLYFQSMASSHLNKGIKQVYMSLPQGEKVQAMYIWIDGTGEGLRCKTRTLDSEPKCVEELPEWNFDGSSTLQSEGSNSDMYLVPAAMFRDPFRKDPNKLVLCEVFKYNRRPAETNLRHTCKRIMDMVSNQHPWFGMEQEYTLMGTDGHPFGWPSNGFPGPQGPYYCGVGADRAYGRDIVEAHYRACLYAGVKIAGTNAEVMPAQWEFQIGPCEGISMGDHLWVARFILHRVCEDFGVIATFDPKPIPGNWNGAGCHTNFSTKAMREENGLKYIEEAIEKLSKRHQYHIRAYDPKGGLDNARRLTGFHETSNINDFSAGVANRSASIRIPRTVGQEKKGYFEDRRPSANCDPFSVTEALIRTCLLNETG

222222222222222222222222222222222222222222222222222222222222222222222222222222222222222222222222222222222222222222222222222222222222222222222222222212121222222222222222222222222222222222222222222222222222222221222222222221211112222222222222222222222222222222222222222222222121222212222222222222222222222222222222222222222222222222222222212222122222222222121222222222222222222222222222

>2D2FA

MSQLEIRDLWASIDGETILKGVNLVVPKGEVHALMGPNGAGKSTLGKILAGDPEYTVERGEILLDGENILELSPDERARKGLFLAFQYPVEVPGVTIANFLRLALQAKLGREVGVAEFWTKVKKALELLDWDESYLSRYLNEGFSGGEKKRNEILQLLVLEPTYAVLDETDSGLDIDALKVVARGVNAMRGPNFGALVITHYQRILNYIQPDKVHVMMDGRVVATGGPELALELEAKGYEWLKEKVKEGA

2222222222221222212222222222222222222111111122222222222222222222222222222222222222222222222222222222222222222222222222222222222222222222222222222222222222222222222222222222222222222222222222222222222222222222222222222222222222222222222222222222222222

>2JA3A

MEAKEEFAHKTLAMDVMKPRRNDPLLTVLTQDSMTVEDVETIISETTYSGFPVVVSRESQRLVGFVLRRDLIISIENARKKQDGVVSTSIIYFTEHSPPLPPYTPPTLKLRNILDLSPFTVTDLTPMEIVVDIFRKLGLRQCLVTHNGRLLGIITKKDVLKHIAQMANQDPDSILFNEFLEVLFQ

22222222222222222122222221122222222222222222221111222222222222222222222222222222222222222222222222222222222222222222222222222222222222222222222222222222221211222222222222222222222222222

>3BXZA

MHHHHHHLTKVFGSRNDRTLRRMRKVVNIINAMEPEMEKLSDEELKGKTAEFRARLEKGEVLENLIPEAFAVVREASKRVFGMRHFDVQLLGGMVLNERCIAEMRTGEGKTLTATLPAYLNALTGKGVHVVTVNDYLAQRDAENNRPLFEFLGLTVGINLPGMPAPAKREAYAADITYGTNNEYGFDYLRDNMAFSPEERVQRKLHYALVDEVDSILIDEARTPLIISGANQTLASITFQNYFRLYEKLAGMTGTADTEAFEFSSIYKLDTVVVPTNRPMIRKDLPDLVYMTEAEKIQAIIEDIKERTAKGQPVLVGTISIEKSELVSNELTKAGIKHNVLNAKFHANEAAIVAQAGYPAAVTIATNMAGRGTDIVLGGSWQAEVAALENPTAEQIEKIKADWQVRHDAVLEAGGLHIIGTERHESRRIDNQLRGRSGRQGDAGSSRFYLSMEDALMRIFASDRVSGMMRK

222222222222222222222222222222222222222222222222222222222222222222222222222222222211112212222222222222221111111122222222222222222222222222222222222222222222222222222222222222222222222222222222222222222222222222222222222222222222222222222222222222222222222222222222222222222222222222222222222222222222222222222222222222222222222222222222222222222222222222222222222222222222212222222222222222222222222222222222222222222222222222222222222222222222222222222222222222222222222

>1G6HA

MRDTMEILRTENIVKYFGEFKALDGVSISVNKGDVTLIIGPNGSGKSTLINVITGFLKADEGRVYFENKDITNKEPAELYHYGIVRTFQTPQPLKEMTVLENLLIGEICPGESPLNSLFYKKWIPKEEEMVEKAFKILEFLKLSHLYDRKAGELSGGQMKLVEIGRALMTNPKMIVMDEPIAGVAPGLAHDIFNHVLELKAKGITFLIIEHRLDIVLNYIDHLYVMFNGQIIAEGRGEEEIKNVLSDPKVVEIYIGE

22222222222222221221222222222222222222221111111122222222222222222222222222222222222222222222222222222222222222222222222222222222222222222222222222222222222222222222222222222222222222222222222222222222222222222222222222222222222222222222222222222222222222222

>3I61A

GSLYNDGNRDQRNFGRNQRNNNSNRYRNSRFNSRPRTRSREDDDEVHFDKTTFSKLIHVPKEDNSKEVTLDSLLEEGVLDKEIHKAITRMEFPGLTPVQQKTIKPILSSEDHDVIARAKTGTGKTFAFLIPIFQHLINTKFDSQYMVKAVIVAPTRDLALQIEAEVKKIHDMNYGLKKYACVSLVGGTDFRAAMNKMNKLRPNIVIATPGRLIDVLEKYSNKFFRFVDYKVLDEADRLLEIGFRDDLETISGILNEKNSKSADNIKTLLFSATLDDKVQKLANNIMNKKECLFLDTVDKNEPEAHERIDQSVVISEKFANSIFAAVEHIKKQIKERDSNYKAIIFAPTVKFTSFLCSILKNEFKKDLPILEFHGKITQNKRTSLVKRFKKDESGILVCTDVGARGMDFPNVHEVLQIGVPSELANYIHRIGRTARSGKEGSSVLFICKDELPFVRELEDAKNIVIAKQEKYEPSEEIKSEVLEAVTEEPEDISDIVISLISSYRSCIKEYRFSERRILPEIASTYGVLLNDPQLKIPVSRRFLDKLGLSRSPIGKAMFEIRDY

22222222222222222222222222222222222222222222222222222222222222222222222222222222222222222221211122122222222222222222221111111122222222222222222222222222222222222222222222222222222222222222222222222222222222222222222222222222222222222222222222222222222222222222222222222222222222222222222222222222222222222222222222222222222222222222222222222222222222222222222222222222222222222222222222222222222222222222221222222222222222222222222222112222222222222222222222222222222222222222222222222222222222222222222222222222222222222222222222222222222222222222222222222222222

>3EZ2A

MSDSSQLHKVAQRANRMLNVLTEQVQLQKDELHANEFYQVYAKAALAKLPLLTRANVDYAVSEMEEKGYVFDKRPAGSSMKYAMSIQNIIDIYEHRGVPKYRDRYSEAYVIFISNLKGGVSKTVSTVSLAHAMRAHPHLLMEDLRILVIDLDPQSSATMFLSHKHSIGIVNATSAQAMLQNVSREELLEEFIVPSVVPGVDVMPASIDDAFIASDWRELCNEHLPGQNIHAVLKENVIDKLKSDYDFILVDSGPHLDAFLKNALASANILFTPLPPATVDFHSSLKYVARLPELVKLISDEGCECQLATNIGFMSKLSNKADHKYCHSLAKEVFGGDMLDVFLPRLDGFERCGESFDTVISANPATYVGSADALKNARIAAEDFAKAVFDRIEFIRSN

22222222222222222222222222222222222222222222222222222222222222222222222222222222222222222222222222222222222222222222211111112222222222222222222222222222222222122111221222222222222222222222222222222222222222222222222222222222222222222222222222222222222222222222222222222222222212112222222222222222222222222222222222212222222222222222222222222221112211221221222222222222222222222222222222222222222222

>2BUFB

TLSRDDAAQVAKVLSEALPYIRRFVGKTLVIKYGGNAMESEELKAGFARDVVLMKAVGINPVVVHGGGPQIGDLLKRLSIESHFIDGMRVTDAATMDVVEMVLGGQVNKDIVNLINRHGGSAIGLTGKDAELIRAKKLTVTRQTPEMTKPEIIDIGHVGEVTGVNVGLLNMLVKGDFIPVIAPIGVGSNGESYNINADLVAGKVAEALKAEKLMLLTNIAGLMDKQGQVLTGLSTEQVNELIADGTIYGGMLPKIRCALEAVQGGVTSAHIIDGRVPNAVLLEIFTDSGVGTLISNRKRH

222222222222222222222222222222212111222222222222222222222222222222222222222222222222222222222222222222222222222222222222222222222222222222222222222222222222222222222222222222222222222222222222222221222222222222222222111211122222222222222222222221112112212222222222222222222222222222222222222222222222

>2G5IB

MHFETVIGLEVHVELKTDSKMFSPSPAHFGAEPNSNTNVIDLAYPGVLPVVNKRAVDWAMRAAMALNMEIATESKFDRKNYFYPDNPKAYQISQFDQPIGENGYIDIEVDGETKRIGITRLHMEEDAGKSTHKGEYSLVDLNRQGTPLIEIVSEPDIRSPKEAYAYLEKLRSIIQYTGVSDVKMEEGSLRCDANISLRPYGQEKFGTKAELKNLNSFNYVRKGLEYEEKRQEEELLNGGEIGQETRRFDESTGKTILMRVKEGSDDYRYFPEPDIVPLYIDDAWKERVRQTIPELPDERKAKYVNELGLPAYDAHVLTLTKEMSDFFESTIEHGADVKLTSNWLMGGVNEYLNKNQVELLDTKLTPENLAGMIKLIEDGTMSSKIAKKVFPELAAKGGNAKQIMEDNGLVQISDEATLLKFVNEALDNNEQSVEDYKNGKGKAMGFLVGQIMKASKGQANPQLVNQLLKQELDKRLEHHHHHH

222221122122222222222222222222222222222222222222222222222222222222222222222222222222222222222222222222222222222222222222222222222222222222222222222222211212222222222222222222222222222222222222212122222222112122222222222222222222222222222222222222222222222222222222222222222222222222222222222222222222222222222222222222222222222222222222222222222222222222222222222222222222222222222222222222222222222222222222222222222222222222222222222222222222222222222222222222222222222222222222222

>1ZARA

MNIAELYGKMGKHSWRIMDAIFKNLWDYEYVPLQLISSHARIGEEKARNILKYLSDLRVVQNRQKDYEGSTFTFIGLSLYSLHRLVRSGKVDAIGKLMGEGKESAVFNCYSEKFGECVVKFHKVGHTSFKKVKEKRDYGDLHFSVLAIRSARNEFRALQKLQGLAVPKVYAWEGNAVLMELIDAKELYRVRVENPDEVLDMILEEVAKFYHRGIVHGDLSQYNVLVSEEGIWIIDFPQSVEVGEEGWREILERDVRNIITYFSRTYRTEKDINSAIDRILQE

222222222222222222222222222222222222222222222222222222222222222222222222222222222222222222222222212222112122222222222121222222222222222222222222222222222222222222222212222222222211112221222222222222222222222222222222222221122222222221122222222222222222222222222222222222222222222222

>2DWCA

MVVMIKLRDELGTATTDSAQKILLLGSGELGKEIAIEAQRLGVEVVAVDRYANAPAMQVAHRSYVGNMMDKDFLWSVVEREKPDAIIPEIEAINLDALFEFEKDGYFVVPNARATWIAMHRERLRETLVKEAKVPTSRYMYATTLDELYEACEKIGYPCHTKAIMSSSGKGSYFVKGPEDIPKAWEEAKTKARGSAEKIIVEEHIDFDVEVTELAVRHFDENGEIVTTFPKPVGHYQIDGDYHASWQPAEISEKAEREVYRIAKRITDVLGGLGIFGVEMFVKGDKVWANEVSPRPHDTGMVTLASHPPGFSEFALHLRAVLGLPIPGEWVDGYRLFPMLIPAATHVIKAKVSGYSPRFRGLVKALSVPNATVRLFGKPEAYVGRRLGIALAWDKDVEVAKRKAEMVAHMIELRTRSSDWHDQNYEKRKHLLR

2222222222222222222222222222222222222222222222222222222222222222222222222222222222222222222222222222222222222222222222221222222222222222222222222222222222222221212222222222222222222222222222222222222221111212212222222222222222222222222212222222222222222222222222222222222222222222122222222112222222222222222222222222222222222222222222222222222222222222222222222222222222222222222222222222222222222222222222222222222222222222222222222

>1OXUA

MVRIIVKNVSKVFKKGKVVALDNVNINIENGERFGILGPSGAGKTTFMRIIAGLDVPSTGELYFDDRLVASNGKLIVPPEDRKIGMVFQTWALYPNLTAFENIAFPLTNMKMSKEEIRKRVEEVAKILDIHHVLNHFPRELSGGQQQRVALARALVKDPSLLLLDEPFSNLDARMRDSARALVKEVQSRLGVTLLVVSHDPADIFAIADRVGVLVKGKLVQVGKPEDLYDNPVSIQVASLIGEINELEGKVTNEGVVIGSLRFPVSVSSDRAIIGIRPEDVKLSKDVIKDDSWILVGKGKVKVIGYQGGLFRITITPLDSEEEIFTYSDHPIHSGEEVLVYVRKDKIKVFEKN

22222222222212222221222222222222222222111111112222222222222222222222222222222222222222222222222222222222222222222222222222222222222222222222222222222222222222222222222222222222222222222222222222222222222222222222222222222222222222222222222222222222222222222222222222222222222222222222222222222222222222222222222222222222222222222222222222222222222222222

>3EX7C

MATTATMATSGSARKRLLKEEDMTKVEFETSEEVDVTPTFDTMGLREDLLRGIYAYGFEKPSAIQQRAIKQIIKGRDVIAQSQSGTGKTATFSISVLQCLDIQVRETQALILAPTRELAVQIQKGLLALGDYMNVQCHACIGGTNVGEDIRKLDYGQHVVAGTPGRVFDMIRRRSLRTRAIKMLVLDEADEMLNKGFKEQIYDVYRYLPPATQVVLISATLPHEILEMTNKFMTDPIRILVKRDELTLEGIKQFFVAVEREEWKFDTLCDLYDTLTITQAVIFCNTKRKVDWLTEKMREANFTVSSMHGDMPQKERESIMKEFRSGASRVLISTDVWARGLDVPQVSLIINYDLPNNRELYIHRIGRSGRYGRKGVAINFVKNDDIRILRDIEQYYSTQIDEMPMNVADLILE

22222222222222222222222222222222222222212222222222222221212122221222222222222222222211111122222222222222222222222222222222222222222222222222222222222222222222222222222222222222222222222222222222222222222222222222222222222222222222222222222222222222222222222222222222222222222222222222222222222222222222222222222222222222222222222222222222222122222222222222222222222222211222222222222222222222222222222222222222222

>1SVLA

GLKEHDFNPEEAEETKQVSWKLVTEYAMETKCDDVLLLLGMYLEFQYSFEMCLKCIKKEQPSHYKYHEKHYANAAIFADSKNQKTICQQAVDTVLAKKRVDSLQLTREQMLTNRFNDLLDRMDIMFGSTGSADIEEWMAGVAWLHCLLPKMDSVVYDFLKCMVYNIPKKRYWLFKGPIDSGKTTLAAALLELCGGKALNVNLPLDRLNFELGVAIDQFLVVFEDVKGTGGESRDLPSGQGINNLDNLRDYLDGSVKVNLEKKHLNKRTQIFPPGIVTMNEYSVPKTLQARFVKQIDFRPKDYLKHCLERSEFLLEKRIIQSGIALLLMLIWYRPVAEFAQSIQSRIVEWKERLDKEFSLSVYQKMKFNVAMGIGVLD

22222222222222222222222222222222222222222222222222222222222222222222222222222222222222222222222222222222222222222222222222222222222222222222221222122222222222222222222222222222111111112222222222222222222222222222222222222222222222222222222222222222222222222222222222222222222222222222222222222222211122122212222221222222222222222222222222222222222222222222222222222222222222222

>1XMVA

GSHMAIDENKQKALAAALGQIEKQFGKGSIMRLGEDRSMDVETISTGSLSLDIALGAGGLPMGRIVEIYGPESSGKTTLTLQVIAAAQREGKTCAFIDAEHALDPIYARKLGVDIDNLLCSQPDTGEQALEICDALARSGAVDVIVVDSVAALTPKAEIEGEIGDSHMGLAARMMSQAMRKLAGNLKQSNTLLIFINQIRMKIGVMFGNPETTTGGNALKFYASVRLDIRRIGAVKEGENVVGSETRVKVVKNKIAAPFKQAEFQILYGEGINFYGELVDLGVKEKLIEKAGAWYSYKGEKIGQGKANATAWLKDNPETAKEIEKKVRELLLSNPNSTPDFSVDDSEGVAETNEDF

22222222222222222222222222222222222222222222222222222222222222222222221111111122222222222222222222222221221222222222222222222222222222222222222222222222222222222222222222222222222222222222222222222122222222222222222222222222222222222222222222222222222222222222222221211222222222222222222222222222222222222222222222222222222222222222222222222222222222222222

>3L8KA

MSLKYDVVVIGAGGAGYHGAFRLAKAKYNVLMADPKGELGGNCLYSGCVPSKTVREVIQTAWRLTNIANVKIPLDFSTVQDRKDYVQELRFKQHKRNMSQYETLTFYKGYVKIKDPTHVIVKTDEGKEIEAETRYMIIASGAETAKLRLPGVEYCLTSDDIFGYKTSFRKLPQDMVIIGAGYIGLEIASIFRLMGVQTHIIEMLDRALITLEDQDIVNTLLSILKLNIKFNSPVTEVKKIKDDEYEVIYSTKDGSKKSIFTNSVVLAAGRRPVIPEGAREIGLSISKTGIVVDETMKTNIPNVFATGDANGLAPYYHAAVRMSIAAANNIMANGMPVDYVDVKSIPVTIYTIPSLSYVGILPSKARKMGIEIVEAEYNMEEDVSAQIYGQKEGVLKLIFERGSMRLIGAWMIGVHSQYLINELGLAVAYGLNAKQLASFAEQHPSTNEIISYTARKVIEGHHHHHH

2222222221121112222222222222222211112222111222222222222222222222222222222222222222222222222222222222222222221112222222222222222222222222221111222222222222222222222222222222222222222222222222222222222222222222222222222222222222222222222222222222222222222222222222222222222212122222222222222222222222222222221122222222221222222222222222222222222222222222222222222222222222222222222222222222222222222222222222222222222222222222222222222222222222222222222222222222222222

>2CGJA

MTFRNCVAVDLGASSGRVMLARYERECRSLTLREIHRFNNGLHSQNGYVTWDVDSLESAIRLGLNKVCAAGIAIDSIGIDTWGVDFVLLDQQGQRVGLPVAYRDSRTNGLMAQAQQQLGKRDIYQRSGIQFLPFNTLYQLRALTEQQPELIPHIAHALLMPDYFSYRLTGKMNWEYTNATTTQLVNINSDDWDESLLAWSGANKAWFGRPTHPGNVIGHWICPQGNEIPVVAVASHDTASAVIASPLNGSRAAYLSSGTWSLMGFESQTPFTNDTALAANITNEGGAEGRYRVLKNIMGLWLLQRVLQERQINDLPALIAATQALPACRFIINPNDDRFINPDEMCSEIQAACREMAQPIPESDAELARCIFDSLALLYADVLHELAQLRGEDFSQLHIVGGGCQNTLLNQLCADACGIRVIAGPVEASTLGNIGIQLMTLDELNNVDDFRQVVSTTANLTTFTPNPDSEIAHYVALIHSTRQTKELCA

222222222222212212222222222222222222222222222222222222222222222222222222222222222222222222222222222222222222222222222222222222222222222222222222222222222222222222222222222222222222222222222222222222222222222222222222222222222222222222222222222222222222222211122222222222222222222222222222222222222221222122222222221222122222222222222222222222222222222222222222222222222222222222222222222222222222222211121122222222222222222222222222222222222222222222222222222222222222222222222222222222222

>3KO3D

MRVVIQRVKGAILSVRKENIGENEKELEIISEIKNGLICFLGIHKNDTWEDALYIIRKCLNLRLWNNDNKTWDKNVKDLNYELLIVSQFTLFGNTKKGNKPDFHLAKEPNEALIFYNKIIDEFKKQYNDDKIKIGKFGNYMNIDVTNDGPVTIYIDTHDINLNK

22222222222222222222222222222222222222222222222222222222222222222222222222222222222222221222222222222222211212222222222222222222222222222222222222222222222222222222

>1XJKA

MKLSDLISRWIDVEPSKNAQIILRDRYFMKDLDGNYLETKWEDVARRVARVVATAELLNPSYKKNEKLDRIKEWEDIFFRVLKARLFIPNSPTLFNAGLGVKHDLLWKPIDQMTLEDYEEIYRSRNHLHMLSACFVVPVGDSIEEIFEAVKEYALITKVGGGVGSNFSELRPKGSFVAGTHGKASGPVSFMHVFNSAISVVKQGSRRRGALMGILNINHPDIEEFIDAKKENTGEAVLNFFNLSVGFPMDKKEILKLYEEDGELELSHPRSTIRKKVKIRELFRKIATNAWKSGDPGLAFLGEMNKYYPLYPHRKINSTNPCGEIGLSDYEACNLGSIDVAKFYNNGFVDLEALQELVQIAVRFLDNVIDVNVFPIDKITKAVKESRRLGLGIMGFADLLYKLEIPYNSQEARDFAANLMAFIALHAHRTSYELGKEKGNFPLLEISRYRTEDNFVPFAMGMSNYDDEIREVMKMTKEFRRNVALLTIAPTGSISNIADTSSGLEPNFLLAYTRFVTKEDGTKEPLLYVNQVLREKLNPEILKRIEKELIEKGSLKDIPDVPEKIKKVFVVALDIDPMDHLLMQDAFQRYVDNNISKTINMPQSATVDDVLNVYLEALRTNVRGITVYRDGSLQTQVLTKALKT

22222222222222222222222222222222222222222222222222222222222222222222222222222222222222222112222222222222222222222222222222222222222211222222222222222222222222221122222222222222222222222222222222222222212222222122222222222222222222222222222222222222222222222222222222222222222222222222222222222222222222222222222222222221112122222222222222222222222222222222222222222222222222222222222222222222222222222222222222222222222222222222222222222222222222222222222222222222222222222222222222222222111111222222222222222222222222222222222222222222222222222222222222222222222222222222222222222222222222222222222222222222222222222222222222222222222222222222

>3DZVA

GUKTSVKFETIFPLTTAPLIQCITNEITCESUANALLYIDAKPIUADDPREFPQUFQQTSALVLNLGHLSQEREQSLLAASDYARQVNKLTVVDLVGYGASDIRNEVGEKLVHNQPTVVKGNLSEURTFCQLVSHGRGVDGSPLDQSEEAIEELIQALRQQTQKFPQTVFLATGIQDVLVSQEQVIVLQNGVPELDCFTGTGDLVGALVAALLGEGNAPUTAAVAAVSYFNLCGEKAKTKSQGLADFRQNTLNQLSLLUKEKDWFEAVKGRVL

222222222222222222222222222222222222222222222222222222222222222222222222222222222222222222222222222222222222222222222222212222222222222222222222222222222222222222222222222211121222222222222111121122221122122222222222222222222212221222222222222222222222222222222222222222222

>2HGSA

MATNWGSLLQDKQQLEELARQAVDRALAEGVLLRTSQEPTSSEVVSYAPFTLFPSLVPSALLEQAYAVQMDFNLLVDAVSQNAAFLEQTLSSTIKQDDFTARLFDIHKQVLKEGIAQTVFLGLNRSDYMFQRSADGSPALKQIEINTISASFGGLASRTPAVHRHVLSVLSKTKEAGKILSNNPSKGLALGIAKAWELYGSPNALVLLIAQEKERNIFDQRAIENELLARNIHVIRRTFEDISEKGSLDQDRRLFVDGQEIAVVYFRDGYMPRQYSLQNWEARLLLERSHAAKCPDIATQLAGTKKVQQELSRPGMLEMLLPGQPEAVARLRATFAGLYSLDVGEEGDQAIAEALAAPSRFVLKPQREGGGNNLYGEEMVQALKQLKDSEERASYILMEKIEPEPFENCLLRPGSPARVVQCISELGIFGVYVRQEKTLVMNKHVGHLLRTKAIEHADGGVAAGVAVLDNPYPV

222222222222222222222222222222222222222222222222222222222222222222222222222222222222222222222222222222222222222222222222222222221222222222222211222222222222222222222222222222222222222222222222222222222222222222222222222222222222222222222222222222222222222222222222222222222222222222222222222222222222222212222222222222222222222222222222222222222222222222222222212122211122121222222222222222222222211112222222222222222222222212222222222222222222222222212222111222222222222222

>3FWYA

MHHHHHHHGMHHHHHHHGSPKDLTIPTGADGEGSVQVHLDEADKITGAKVFAVYGKGGIGKSTTSSNLSAAFSILGKRVLQIGCDPKHDSTFTLTGSLVPTVIDVLKDVDFHPEELRPEDFVFEGFNGVMCVEAGGPPAGTGCGGYVVGQTVKLLKQHHLLDDTDVVIFDVLGDVVCGGFAAPLQHADQAVVVTANDFDSIYAMNRIIAAVQAKSKNYKVRLAGCVANRSRATDEVDRFCKETNFRRLAHMPDLDAIRRSRLKKKTLFEMDEDQDVLAARAEYIRLAESLWRGLDPIDPHSLPDRDIFELLGFD

22222222222222222222222222222222222222222222222222222222211111122222222222222222222222122222222222222222222222222222222222222222222222222222222222222222222222222222222222222222222222222222222222222222222222222222222222222222222112222222222222222222221111221222122222222222222222222222222222222222222222222222222222

>1FNNA

MAIVVDDSVFSPSYVPKRLPHREQQLQQLDILLGNWLRNPGHHYPRATLLGRPGTGKTVTLRKLWELYKDKTTARFVYINGFIYRNFTAIIGEIARSLNIPFPRRGLSRDEFLALLVEHLRERDLYMFLVLDDAFNLAPDILSTFIRLGQEADKLGAFRIALVIVGHNDAVLNNLDPSTRGIMGKYVIRFSPYTKDQIFDILLDRAKAGLAEGSYSEDILQMIADITGAQTPLDTNRGDARLAIDILYRSAYAAQQNGRKHIAPEDVRKSSKEVLFGISEEVLIGLPLHEKLFLLAIVRSLKISHTPYITFGDAEESYKIVCEEYGERPRVHSQLWSYLNDLREKGIVETRQNKRGEGVRGRTTLISIGTEPLDTLEAVITKLIKEELR

22222222222221212211212222222222222222222222222222222111111222222222222222222222222222222222222222222222222222222222222222222222222222222222222222222222222222222222222222222222222222222222222212222222122212222222222222222222222222222222222112212222222222222222222222222222222222222222222222222222222222222222222222222222222222222222222222222222222222222222222222222222222222222222222222222

>1GC5A

MKESLKDRIRLWKRLYVNAFENALNAIPNVKGVLLAYNTNIDAIKYLDADDLEKRVTEKGKEKVFEIIENPPEKISSIEELLGGILRSIKLGKAMEWFVESEEVRRYLREWGWDELRIGGQAGIMANLLGGVYRIPTIVHVPQNPKLQAELFVDGPIYVPVFEGNKLKLVHPKDAIAEEEELIHYIYEFPRGFQVFDVQAPRENRFIANADDYNARVYMRREFREGFEEITRNVELAIISGLQVLKEYYPDGTTYKDVLDRVESHLNILNRYNVKSHFEFAYTANRRVREALVELLPKFTSVGLNEVELASIMEIIGDEELAKEVLEGHIFSVIDAMNVLMDETGIERIHFHTYGYYLALTQYRGEEVRDALLFASLAAAAKAMKGNLERIEQIRDALSVPTNERAIVLEEELEKEFTEFENGLIDMVDRQLAFVPTKIVASPKSTVGIGDTISSSAFVSEFGMRKR

22222222222222222222222222222222222222222222222222222222222222222222222222222222222222222222222222222222222222222222222222222222222222222222222222222222222222222222222222222222222222222222222222222222222212222222222222222222222222222222222222222222222222222222222222222222222222222222222222222222222222221222222222222222222222222222222222222222222222211222222222222222222222222222222222222222222222222222222222222222222222222222222222222111221222221122122222222222222

>1M15A

MVDQATLDKLEAGFKKLQEASDCKSLLKKHLTKDVFDSIKNKKTGMGATLLDVIQSGVENLDSGVGIYAPDAESYRTFGPLFDPIIDDYHGGFKLTDKHPPKQWGDINTLVGLDPAGQFIISTRVRCGRSLQGYPFNPCLTAEQYKEMEEKVSSTLSSMEDELKGTYYPLTGMSKATQQQLIDDHFLFKEGDRFLQTANACRYWPTGRGIFHNDAKTFLVWVNEEDHLRIISMQKGGDLKTVYKRLVTAVDNIESKLPFSHDDRFGFLTFCPTNLGTTMRASVHIQLPKLAKDRKVLEDIASKFNLQVRGTRGEHTESEGGVYDISNKRRLGLTEYQAVREMQDGILEMIKMEKAAA

222222222222222222222222222222222222222222222222222222222222222222222222222222222222222222222222222222222222222222222222212121222222222222222222222222222222222222222222222222222222222212222222222222222222222222222222222212222222122212222222222222222222222222222222222222222222222121112222222222222222222222221211112222222221222222222222222222222222222222222

>2B9FA

MPKRIVYNISSDFQLKSLLGEGAYGVVCSATHKPTGEIVAIKKIEPFDKPLFALRTLREIKILKHFKHENIITIFNIQRPDSFENFNEVYIIQELMQTDLHRVISTQMLSDDHIQYFIYQTLRAVKVLHGSNVIHRDLKPSNLLINSNCDLKVCDFGLARIIDESAADNSEPTGQQSGMTEYVATRWYRAPEVMLTSAKYSRAMDVWSCGCILAELFLRRPIFPGRDYRHQLLLIFGIIGTPHSDNDLRCIESPRAREYIKSLPMYPAAPLEKMFPRVNPKGIDLLQRMLVFDPAKRITAKEALEHPYLQTYHDPNDEPEGEPIPPSFFEFDHYKEALTTKDLKKLIWNEIFS

22222222222222222212222112122222222222212122222222222212222222222222222122222222222222222222112122122222222222222222222222222222222222222222112122222222211222222222222222222222222222222222222222222222222222222222222222222222222222222222222222222222222222222222222222222222222222222222222222222222222222222222222222222222222222222222222222222222222222222

>1NY5A

MNVLVIEDDKVFRGLLEEYLSMKGIKVESAERGKEAYKLLSEKHFNVVLLDLLLPDVNGLEILKWIKERSPETEVIVITGHGTIKTAVEAMKMGAYDFLTKPCMLEEIELTINKAIEHRKLRKENELLRREKDLKEEEYVFESPKMKEILEKIKKISCAECPVLITGESGVGKEVVARLIHKLSDRSKEPFVALNVASIPRDIFEAELFGYEKGAFTGAVSSKEGFFELADGGTLFLDEIGELSLEAQAKLLRVIESGKFYRLGGRKEIEVNVRILAATNRNIKELVKEGKFREDLYYRLGVIEIEIPPLRERKEDIIPLANHFLKKFSRKYAKEVEGFTKSAQELLLSYPWYGNVRELKNVIERAVLFSEGKFIDRGELSCLVNSK

222222222222222222222222222222222222222222222222222222222222222222222222222222222222222222222222222222222222222222222222222222222222222211111222222222222222222222222222211111122222222222222222222222222222222222222222222222222222222222222222222222222222222222222222222222222222222222222222222222222222222222222222222222212221222222222222222222222222222222211221222222222222222222222222222

>1J7LA

MAKMRISPELKKLIEKYRCVKDTEGMSPAKVYKLVGENENLYLKMTDSRYKGTTYDVEREKDMMLWLEGKLPVPKVLHFERHDGWSNLLMSEADGVLCSEEYEDEQSPEKIIELYAECIRLFHSIDISDCPYTNSLDSRLAELDYLLNNDLADVDCENWEEDTPFKDPRELYDFLKTEKPEEELVFSHGDLGDSNIFVKDGKVSGFIDLGRSGRADKWYDIAFCVRSIREDIGEEQYVELFFDLLGIKPDWEKIKYYILLDELF

222222222222222222222121221222122222222221212222222222222222222222222222222222222222222221111222122222222222222222222222222222222222222222222222222222222222222222222222222222222222222222222222211212222222221122222222222222222222222222222222222222222222222222222222

>1TZDA

SWVQLAGHTGSFKAAGTSGLILKRSSEPEHYCUVRLUADVLRGCVPAFHGVVERDGESYLQLQDLLDGFDGPCVLDCKUGVRTYLEEELTKARERPKLRKDUYKKULAVDPEAPTEEEHAQRAVTKPRYUQWREGISSSTTLGFRIEGIKKADGSCSTDFKTTRSREQVTRVFEEFUQGDAEVLKRYLNRLQQIRDTLEISDFFRRHEVIGSSLLFVHDHCHRAGVWLIDFGKTTPLPNGQILDHRRPWEEGNREDGYLLGLDNLIGILANLAER

22222222222222222222221222222222222222222222212222222222222222111222222222212222222222222222222222222222222222222222222222222222222222222222222222222222222222222222222222222222222222222222222222222222222222222222222222222222222211222222222222222222222222222222222222222222222

>2QENA

MLFDLRPKTRREDIFDREEESRKLEESLENYPLTLLLGIRRVGKSSLLRAFLNERPGILIDCRELYAERGHITREELIKELQSTISPFQKFQSKFKISLNLKFLTLEPRKLSLREVFRELNDLGEELGEFIVAFDEAQYLRFYGSRGGKELLALFAYAYDSLPNLKIILTGSEVGLLHDFLKITDYESPLYGRIAGEVLVKPFDKDTSVEFLKRGFREVNLDVPENEIEEAVELLDGIPGWLVVFGVEYLRNGDFGRAMKRTLEVAKGLIMGELEELRRRSPRYVDILRAIALGYNRWSLIRDYLAVKGTKIPEPRLYALLENLKKMNWIVEEDNTYKIADPVVATVLRI

21222221222221121222222222222222222222111111112222222222222222222222222222222222222222222222222222222222222222222222222222222222222222222222222222222222222222222222222222222222222222222222222222222222222222222212222222222222222222222222221122222222222222222222222222222222222222222222222222222222222222222222222222222222222222222222222222222222222222

>1OH9A

MMNPLIIKLGGVLLDSEEALERLFSALVNYRESHQRPLVIVHGGGCVVDELMKGLNLPVKKKNGLRVTPADQIDIITGALAGTANKTLLAWAKKHQIAAVGLFLGDGDSVKVTQLDEELGHVGLAQPGSPKLINSLLENGYLPVVSSIGVTDEGQLMNVNADQAATALAATLGADLILLSDVSGILDGKGQRIAEMTAAKAEQLIEQGIITDGMIVKVNAALDAARTLGRPVDIASWRHAEQLPALFNGMPMGTRILA

222222212112222222222222222222222222222222222222222222222222222222222222222222222222222222222222222222222222222222222222222222222222222222222222222222222222222221222222222222222221222111222222222222222222222211111122122222222222222222222222222222222222222222

>1CQIB

MNLHEYQAKQLFARYGLPAPVGYACTTPREAEEAASKIGAGPWVVKCQVHAGGRGKAGGVKVVNSKEDIRAFAENWLGKRLVTYQTDANGQPVNQILVEAATDIAKELYLGAVVDRSSRRVVFMASTEGGVEIEKVAEETPHLIHKVALDPLTGPMPYQGRELAFKLGLEGKLVQQFTKIFMGLATIFLERDLALIEINPLVITKQGDLICLDGKLGADGNALFRQPDLREMRDQSQEDPREAQAAQWELNYVALDGNIGCMVNGAGLAMGTMDIVKLHGGEPANFLDVGGGATKERVTEAFKIILSDDKVKAVLVNIFGGIVRCDLIADGIIGAVAEVGVNVPVVVRLEGNNAELGAKKLADSGLNIIAAKGLTDAAQQVVAAV

2222222222222222222222222222222222222222222121222222111222222222222222222222222222222222222222222211112122122222222222222222222222222222222222222222222222222222222222222222222222222222222222222222221122222222222112222222222222222222222222222222222222222222222222222222222222222222222222222222222222222222222222222222222222222222222222222222222222222222222222222222222222222222222222222

>1HUXA

MSIYTLGIDVGSTASKCIILKDGKEIVAKSLVAVGTGTSGPARSISEVLENAHMKKEDMAFTLATGYGRNSLEGIADKQMSELSCHAMGASFIWPNVHTVIDIGGQDVKVIHVENGTMTNFQMNDKCAAGTGRFLDVMANILEVKVSDLAELGAKSTKRVAISSTCTVFAESEVISQLSKGTDKIDIIAGIHRSVASRVIGLANRVGIVKDVVMTGGVAQNYGVRGALEEGLGVEIKTSPLAQYNGALGAALYAYKKAAKSAWSHPQFEK

222222222211112122222222222222222222222222222222222222222222222222222222222222222222222222222222222222211222222222222222222222222221222222222222211222222222222222222222222222222222222222222222222222222222222222222222112122222222222222222222221222222222222222222222222222

>2DHRA

RNGRAGPSDSAFSFTKSRARVLTEAPKVTFKDVAGAEEAKEELKEIVEFLKNPSRFHEMGARIPKGVLLVGPPGVGKTHLARAVAGEARVPFITASGSDFVEMFVGVGAARVRDLFETAKRHAPCIVFIDEIDAVGRKRGSGVGGGNDEREQTLNQLLVEMDGFEKDTAIVVMAATNRPDILDPALLRPGRFDRQIAIDAPDVKGREQILRIHARGKPLAEDVDLALLAKRTPGFVGADLENLLNEAALLAAREGRRKITMKDLEEAADRVMMLPAKKSLVLSPRDRRITAYHEAGHALAAHFLEHADGVHKVTIVPRGRALGFMMPRREDMLHWSRKRLLDQIAVALAGRAAEEIVFDDVTTGAENDFRQATELARRMITEWGMHPEFGPVAYAVREDTYLGGYDVRQYSEETAKRIDEAVRRLIEEQYQRVKALLLEKREVLERVAETLLERETLTAEEFQRVVEGLPLEAPEEAREEREPPRVVPKVKPGGALGGA

2222222222222222222222222222222222222222222222222222222222222222222222212111111221222222222222222222222222222222222222222222222222222222222222222222222222222222222222222222222222222222222222222222222222222222122212222222222222222222222221221222222222222222222222222222222222222222222222222222222222222222222222222222222222222222222222222222222222222222222222222222222222222222222222222222222222222222222222222222222222222222222222222222222222222222222222222222222222222222222222222222222222222222222

>2QR1G

AMDVQETQKGALKEIQAFIRSRTSYDVLPTSFRLIVFDVTLFVKTSLSLLTLNNIVSAPLWDSEANKFAGLLTMADFVNVIKYYYQSSSFPEAIAEIDKFRLLGLREVERKIGAIPPETIYVHPMHSLMDACLAMSKSRARRIPLIDVDGETGSEMIVSVLTQYRILKFISMNCKETAMLRVPLNQMTIGTWSNLATASMETKVYDVIKMLAEKNISAVPIVNSEGTLLNVYESVDVMHLIQDGDYSNLDLSVGEALLKRPANFDGVHTCRATDRLDGIFDAIKHSRVHRLFVVDENLKLEGILSLADILNYIIYDKTTTPGVPEQTDNFESAV

2222222222222222222222222222222211122222222222222222221212122222222222222222222222222222222222222222222222222222222222222222222222222222222211222222222222222222212112222222222222222222222222122111222222222222222222111121222222222222222222222222222222222222222222222222222222222222222222121122222222222212122122222222222222222222222222

>1KSFX

MLNQELELSLNMAFARAREHRHEFMTVEHLLLALLSNPSAREALEACSVDLVALRQELEAFIEQTTPVLPASEEERDTQPTLSFQRVLQRAVFHVQSSGRNEVTGANVLVAIFSEQESQAAYLLRKHEVSRLDVVNFISHGTRKDEPTQSSDPGSQPNSEEQAGGEERLENFTTNLNQLARVGGIDPLIGREKELERAIQVLCRRRKNNPLLVGESGVGKTAIAEGLAWRIVQGDVPEVMADCTIYSLDIGSLLAGTKYRGDFEKRFKALLKQLEQDTNSILFIDEIHTIIGAGAASGGQVDAANLIKPLLSSGKIRVIGSTTYQEFSNIFEKDRALARRFQKIDITEPSIEETVQIINGLKPKYEAHHDVRYTAKAVRAAVELAVKYINDRHLPDKAIDVIDEAGARARLMPVSKRKKTVNVADIESVVARIARIPEKSVSQSDRDTLKNLGDRLKMLVFGQDKAIEALTEAIKMARAGLGHEHKPVGSFLFAGPTGVGKTEVTVQLSKALGIELLRFDMSEYMERHTVSRLIGAPPGYVGFDQGGLLTDAVIKHPHAVLLLDEIEKAHPDVFNILLQVMDNGTLTDNNGRKADFRNVVLVMTTNAGVRETERKSIGLIHQDNSTDAMEEIKKIFTPEFRNRLDNIIWFDHLSTDVIHQVVDKFIVELQVQLDQKGVSLEVSQEARNWLAEKGYDRAMGARPMARVIQDNLKKPLANELLFGSLVDGGQVTVALDKEKNELTYGFQSAQKHKAEAAH

22222222222222222222222222222222222222222222222222222222222222222222222222222222222222222222222222222222222222222222222222222222222222222222222222222222222222222222222222222222222222222211121222222222222222222222221111111122222222222222222222222222222222222222222222222222222222222222222222222222222222222222222222222222222222222222222222222222222222222222122212222222222222222222222222222222221122122222222222222222222222222222222222222222222222222222222222111212222222222222222222222222222222221111111222222222222222222222222222222222222222222222222222222222222222222222222222222222222222222222222222222222222222222222222222222222222222222222222222221222222212221222222222222222222222222222222222221122222222222222222222222222222222222222222222222222222222

>1TY8A

MTGSLNRHSLLNGVKKMRIILCDTNEVVTNLWQESIPHAYIQNDKYLCIHHGHLQSLMDSMRKGDAIHHGHSYAIVSPGNSYGYLGGGFDKALYNYFGGKPFETWFRNQLGGRYHTVGSATVVDLQRCLEEKTIECRDGIRYIIHVPTVVAPSAPIFNPQNPLKTGFEPVFNAMWNALMHSPKDIDGLIIPGLCTGYAGVPPIISCKSMAFALRLYMAGDHISKELKNVLIMYYLQYPFEPFFPESCKIECQKLGIDIEMLKSFNVEKDAIELLIPRRILTLDL

22222222222222222222221112222222222222222222222222211122222222222222222222222122222222211221222222222222222222222222222222222222222222222222222222212222222222222222222222222222222222222222222121111122222222222222222222222222222222222222222222222222222222222222222222222222222222222222

>1YP4A

MAVSDSQNSQTCLDPDASRSVLGIILGGGAGTRLYPLTKKRAKPAVPLGANYRLIDIPVSNCLNSNISKIYVLTQFNSASLNRHLSRAYASNMGGYKNEGFVEVLAAQQSPENPDWFQGTADAVRQYLWLFEEHTVLEYLILAGDHLYRMDYEKFIQAHRETDADITVAALPMDEKRATAFGLMKIDEEGRIIEFAEKPQGEQLQAMKVDTTILGLDDKRAKEMPFIASMGIYVISKDVMLNLLRDKFPGANDFGSEVIPGATSLGMRVQAYLYDGYWEDIGTIEAFYNANLGITKKPVPDFSFYDRSAPIYTQPRYLPPSKMLDADVTDSVIGEGCVIKNCKIHHSVVGLRSCISEGAIIEDSLLMGADYYETDADRKLLAAKGSVPIGIGKNCHIKRAIIDKNARIGDNVKIINKDNVQEAARETDGYFIKSGIVTVIKDALIPSGIII

2222222222222222222222222111122222222222222222222222222222222222222222221222222222222222222222222222222222222222222221212212222222222222222222111222222222222222222222222222222222222222222222222222222222222222222222222222222222222222222222222222222222221211222222222222222222222222222222222222222222222222222222222222222222222222222222222222222222222222222222222222222222222222222222222222222222222222222222222222222222222222222222222222222222222222222

>2QSYA

UGSSHHHHHHSSGLVPRGSKTFIIGISGVTNSGKTTLAKNLQKHLPNCSVISQDDFFKPESEIETDKNGFLQYDVLEALNUEKUUSAISCWUESARHSVVSTDQESAEEIPILIIEGFLLFNYKPLDTIWNRSYFLTIPYEECKRRRSTRVYQPPDSPGYFDGHVWPUYLKYRQEUQDITWEVVYLDGTKSEEDLFLQVYEDLIQEL

222222222222222222222222222222111111222222222222222222222222222222222222222222222222222222222222222222222222222222222222222222222222222222222222212221222222222222222222222222222222222222222111221222222222222

>3CR3A

LLTIDTTIEWLGKFNEKIQENKAYLSELDGPIGDGDHGANUARGUSETUKALEVSNFGNVSEIFKKVAUTLUSKVGGASGPLYGSAFLAUSKTAIETLDTSELIYAGLEAIQKRGKAQVGEKTUVDIWSAFLNDLQTDSASKDNLEKVVKASAGLLATKGRASYLGERSIGHIDPGTQSSAYLFETLLEVVA

222222222222222222222222222212222121122122222222222222222222222222222222222211122122222222222222222222222222222222121222221222222222222222222222222222222222222112222222222221112222222222222222

>3I0OA

MLKQPIQAQQLIELLKVHYGIDIHTAQFIQGGADTNAFAYQADSESKSYFIKLKYGYHDEINLSIIRLLHDSGIKEIIFPIHTLEAKLFQQLKHFKIIAYPFIHAPNGFTQNLTGKQWKQLGKVLRQIHETSVPISIQQQLRKEIYSPKWREIVRSFYNQIEFDNSDDKLTAAFKSFFNQNSAAIHRLVDTSEKLSKKIQPDLDKYVLCHSDIHAGNVLVGNEESIYIIDWDEPMLAPKERDLMFIGGGVGNVWNKPHEIQYFYEGYGEINVDKTILSYYRHERIVEDIAVYGQDLLSRNQNNQSRLESFKYFKEMFDPNNVVEIAFATEQLEHHHHHH

222222222222222222222222222222221222222222222222212122222222222222222222222221222222222222222222222211122212222222222222222222222222222222222222222222222222222222222222222222222222222222222222222222222222222222222222122222222222112122222222222222222222222222222222222222222222222222222222222222222222222222222222222222222222222222222222222

>2OWMA

MPNSLDVHQRQTRSNVSTPTLRPRDDTASSFVSKDPGANVRVVVRVRAFLPRELERNAECIVEMDPATERTSLLVPQETDFADARGARSRRVLEEKSFTFDKSFWSHNTEDEHYATQEHVYDSLGEEFLDHNFEGYHTCIFAYGQTGSGKSYTMMGTPDQPGLIPRTCEDLFQRIASAQDETPNISYNVKVSYFEVYNEHVRDLLAPVVPNKPPYYLKVRESPTEGPYVKDLTEVPVRGLEEIIRWMRIGDGSRTVASTKMNDTSSRSHAVFTIMLKQIHHDLETDDTTERSSRIRLVDLAGSERAKSTEATGQRLREGSNINKSLTTLGRVIAALADPKSSASRPSSPVKSGRGRTPGPANSVVPYRDSVLTWLLKDSLGGNSKTAMIACISPTDYDETLSTLRYADQAKRIRTRAVVNQVDGVSAAERDAQIPSIVHRKCF

22222222222222222222222222222222222222222222221121222222222222222222222222222222222222222222222222222222212222222222222222222222222222222222222211111111222222122222222222222222222222222222222222222222222222222222222222222222222222222222222222222222222222222222222222222222222222222222222222222222222222222222222222222222222222222222222222222222222222222222222222222222222222222222222222222222222222222222222222222222222222222222222222222222222

>1Z6TA

MDAKARNCLLQHREALEKDIKTSYIMDHMISDGFLTISEEEKVRNEPTQQQRAAMLIKMILKKDNDSYVSFYNALLHEGYKDLAALLHDGIPVVSSSSGKDSVSGITSYVRTVLCEGGVPQRPVVFVTRKKLVNAIQQKLSKLKGEPGWVTIHGMAGCGKSVLAAEAVRDHSLLEGCFPGGVHWVSVGKQDKSGLLMKLQNLCTRLDQDESFSQRLPLNIEEAKDRLRILMLRKHPRSLLILDDVWDSWVLKAFDSQCQILLTTRDKSVTDSVMGPKYVVPVESSLGKEKGLEILSLFVNMKKADLPEQAHSIIKECKGSPLVVSLIGALLRDFPNRWEYYLKQLQNKQFKRIRKSSSYDYEALDEAMSISVEMLREDIKDYYTDLSILQKDVKVPTKVLCILWDMETEEVEDILQEFVNKSLLFCDRNGKSFRYYLHDLQVDFLTEKNCSQLQDLHKKIITQFQRYHQPHTLSPDQEDCMYWYNFLAYHMASAKMHKELCALMFSLDWIKAKTELVGPAHLIHEFVEYRHILDEKDCAVSENFQEFLSLNGHLLGRQPFPNIVQLGLCEPETSEVYQQAKLQAKQEVDNG

222222222222222222222222222222222222222222222222222222222222222222222222222222222222222222222222222222222222222222222222222211121222222222222222222222222212111111222222222222222222222222222222222222222222222222222222222222222222222222222222222222222222222222222222222222222222222222222222222222222222222222222222222222221122122222222222222222222222222222222222222222222222222222222222222222222222222222222222222222222222222212222222222221222222222222222222222222222222222222222222222222222222222222222222222222222222222222222222222222222222222222222222222222222222222222222222222222222222222

>3C4NA

UTGPEPVPAGPPPDPTPPRRAGSVWAHVGQHFTEEAFDIVVIGAGRUGAACAFYLRQLAPGRSLLLVEEGGLPNEEGATILAPGVWTAQDIPAGQEAQAEWTREQLLGALGSGKTLEVEDRPLLHLLPAGEGSGLTPTLDALADFPEALALLDPARLPVARVDPRALTYRPGSLALLAAQQAIGQGAGLLLNTRAELVPGGVRLHRLTVTNTHQIVVHETRQIRAGVIIVAAGAAGPALVEQGLGLHTRHGRAYRQFPRLDLLSGAQTPVLRASGLTLRPQNGGYTLVPAIHHRDPHGYHPAGGSLTGVPTGLRRELLEDLVGLUDAVPALAGEGLELGRSSADVPGAWLALPGGRPDAPPQAEELAPGLHLLLGGPLADTLGLAAAHELAQRVSASLEHHHHHH

222222222222222222222222222222222222222221111122222222222222222222111222212221122222222222222222222222222222222222222222222222222222222222222222222222222222222222222222222222222222222222222222111222222222222222222222222222222222221112222212222222222222222222222222222222222222222222222222222222222222222222222222222222222222222222222222222222222222222222222222222222222222222222222222222222222222222222222

>2P05A

GSMDYKDDDDKKTNWLKRIYRVRPCVKCKVAPRNWKVKNKHLRIYNMCKTCFNNSIDIGDDTYHGHDDWLMYADSKEISNT

222222222222222222222222222222222121212222121111222122222222221111222222222222222

>2BTDA

MSLSRTQIVNWLTRCGDIFSTESEYLTGLDREIGDADHGLNMNRGFSKVVEKLPAIADKDIGFILKNTGMTLLSSVGGASGPLFGTFFIRAAQATQARQSLTLEELYQMFRDGADGVISRGKAEPGDKTMCDVWVPVVESLRQSSEQNLSVPVALEAASSIAESAAQSTITMQARKGRASYLGERSIGHQDPGATSVMFMMQMLALAAKE

222222222222222222222222222221222212112212222222222222222222222222222222222221112212222222222222222222222222222222222222122222221122222222222222222222222222222222222222222222221122222222222211112222222222222222

>1SXJE

MSLWVDKYRPKSLNALSHNEELTNFLKSLSDQPRDLPHLLLYGPNGTGKKTRCMALLESIFGPGVYRLKIDVRQFVTASNRKLELNVVSSPYHLEITPSDMGNNDRIVIQELLKEVAQMEQVDFQDSKDGLAHRYKCVIINEANSLTKDAQAALRRTMEKYSKNIRLIMVCDSMSPIIAPIKSQCLLIRCPAPSDSEISTILSDVVTNERIQLETKDILKRIAQASNGNLRVSLLMLESMALNNELALKSSSPIIKPDWIIVIHKLTRKIVKERSVNSLIECRAVLYDLLAHCIPANIILKELTFSLLDVETLNTTNKSSIIEYSSVFDERLSLGNKAIFHLEGFIAKVMCCLD

222212221122222211222222222222222222222222211111111222222222222222222222222222222222222222222222222222222222222222222222222222222222222222222222222222222222222222222222222222222222222222222222222222221222222222222222222222222222211221222222222222222222222222222222222222222222222222222222222222222222222222222222222222222222222222222222222222222222222222

>3BF1A

MDPMYLLVDVGNTHSVFSITEDGKTFRRWRLSTGVFQTEDELFSHLHPLLGDAMREIKGIGVASVVPTQNTVIERFSQKYFHISPIWVKAKNGCVKWNVKNPSEVGADRVANVVAFVKEYGKNGIIIDMGTATTVDLVVNGSYEGGAILPGFFMMVHSLFRGTAKLPLVEVKPADFVVGKDTEENIRLGVVNGSVYALEGIIGRIKEVYGDLPVVLTGGQSKIVKDMIKHEIFDEDLTIKGVYHFCFGD

222222222211112222222222222221222211222222222222222222222222222222212222222222222222222222222222222222222222222222222222222222222112222222222222222222221222222222222222222222222222222222222222222222222222222222222222222122222222222222222222222222222

>1XRJA

MAGDSEQTLQNHQQPNGGEPFLIGVSGGTASGKSSVCAKIVQLLGQNEVDYRQKQVVILSQDSFYRVLTSEQKAKALKGQFNFDHPDAFDNELILKTLKEITEGKTVQIPVYDFVSHSRKEETVTVYPADVVLFEGILAFYSQEVRDLFQMKLFVDTDADTRLSRRVLRDISERGRDLEQILSQYITFVKPAFEEFCLPTKKYADVIIPRGADNLVAINLIVQHIQDILNGGPSKRQTNGCLNGYTPSRKRQASESSSRPH

222222222222222222222222222121111112222222222222222222222222222222222222222222222222222222222222222222222222222222222222222222222222222222222222222222222222222222221222122222222222222222222222222222222222222222221222222222222222222222222222222222222222222222222

>1XX6A

MYRPKDHGWVEVIVGPUYSGKSEELIRRIRRAKIAKQKIQVFKPEIDNRYSKEDVVSHUGEKEQAVAIKNSREILKYFEEDTEVIAIDEVQFFDDEIVEIVNKIAESGRRVICAGLDUDFRGKPFGPIPELUAIAEFVDKIQAICVVCGNPATRTQRLINGKPAFYDDPVVLIGAUESYEARCRKCHVVPQ

22222222222222212111111222222222222222222222222222222222112222222222222222222222222222222222222222222222222222222222222222222222222222222222221121222222222222222222222222222222222222222222222

>2NR8A

MGSSHHHHHHSSGLVPRGSGTRKKVHAFVRVKPTDDFAHEMIRYGDDKRSIDIHLKKDIRRGVVNNQQTDWSFKLDGVLHDASQDLVYETVAKDVVSQALDGYNGTIMCYGQTGAGKTYTMMGATENYKHRGILPRALQQVFRMIEERPTHAITVRVSYLEIYNESLFDLLSTLPYVGPSVTPMTIVENPQGVFIKGLSVHLTSQEEDAFSLLFEGETNRIIASHTMNKNSSRSHCIFTIYLEAHSRTLSEEKYITSKINLVDLAGSERLGKSGSEGQVLKEATYINKSLSFLEQAIIALGDQKRDHIPFRQCKLTHALKDSLGGNCNMVLVTNIYGEAAQLEETLSSLRFASRMKLV

2222222222222222222222222222212112222222222222222222222222222222222222222222222222222222222222222222222222222221111111122222222222222222222222222222222222222222222222222222222222222222222222222222222222222222222222222222222222222222222222222222222222222222222222222222222222222222222222222222222222222222222222222222222222222222222222222222222222222222222222

>2AD5A

MTTNYIFVTGGVVSSLGKGIAAASLAAILEARGLNVTIMKLDPYINVDPGTMSPIQHGEVFVTEDGAETDLDLGHYERFIRTKMSRRNNFTTGRIYSDVLRKERRGDYLGATVQVIPHITNAIKERVLEGGEGHDVVLVEIGGTVGDIESLPFLEAIRQMAVEIGREHTLFMHLTLVPYMAASGEVKTKPTQHSVKELLSIGIQPDILICRSDRAVPANERAKIALFCNVPEKAVISLKDVDSIYKIPGLLKSQGLDDYICKRFSLNCPEANLSEWEQVIFEEANPVSEVTIGMVGKYIELPDAYKSVIEALKHGGLKNRVSVNIKLIDSQDVETRGVEILKGLDAILVPGGFGYRGVEGMITTARFARENNIPYLGICLGMQVALIDYARHVANMENANSTEFVPDCKYPVVALITEWRDENGNVEVRSEKSDLGGTMRLGAQQCQLVDDSLVRQLYNAPTIVERHRHRYEVNNMLLKQIEDAGLRVAGRSGDDQLVEIIEVPNHPWFVACQFHPEFTSTPRDGHPLFAGFVKAASEFQKRQAK

22222222222222111111222222222222222222222222222222222222222222222222222122222222222222222222222222222222222222222222222222222222222222222221222222222222222222222222222222222222222221222222222222222222222222222212222222222222222222222222221112222222222222222222222222222222222222222222222222222222222222222222222222222222222222222222222222222222222222222222222222222222222222222222222222222222222222222222222222222222222222222222222222222222222222222222222222222222222222222222222222222222222222222222222222222222222222222222222222222222222222222

>2FNAA

GULFDTSPKDNRKDFFDREKEIEKLKGLRAPITLVLGLRRTGKSSIIKIGINELNLPYIYLDLRKFEERNYISYKDFLLELQKEINKLVKRLPSLLKALKNIQGIVIUGNEIKFNWNRKDRLSFANLLESFEQASKDNVIIVLDEAQELVKLRGVNLLPALAYAYDNLKRIKFIUSGSEUGLLYDYLRVEDPESPLFGRAFSTVELKPFSREEAIEFLRRGFQEADIDFKDYEVVYEKIGGIPGWLTYFGFIYLDNKNLDFAINQTLEYAKKLILKEFENFLHGREIARKRYLNIURTLSKCGKWSDVKRALELEEGIEISDSEIYNYLTQLTKHSWIIKEGEKYCPSEPLISLAFS

221211111222211122222222222222222222211111111222222222222222222222222222222222222222222222222222222222222222222222222222222222222222222222222222222222222222222222222222222222222222222222222222222222222222222212222222122222222222222222222222221122122222222222222222222222222222222222222222222222222222222222222222222222222222222222222222222222222222222222222

>2JFGA

MADYQGKNVVIIGLGLTGLSCVDFFLARGVTPRVMDTRMTPPGLDKLPEAVERHTGSLNDEWLMAADLIVASPGIALAHPSLSAAADAGIEIVGDIELFCREAQAPIVAITGSNGKSTVTTLVGEMAKAAGVNVGVGGNIGLPALMLLDDECELYVLELSSFQLETTSSLQAVAATILNVTEDHMDRYPFGLQQYRAAULRIYENAKVCVVNADDALTMPIRGADERCVSFGVNMGDYHLNHQQGETWLRVKGEKVLNVKEMKLSGQHNYTNALAALALADAAGLPRASSLKALTTFTGLPHRFEVVLEHNGVRWINDSKATNVGSTEAALNGLHVDGTLHLLLGGDGKSADFSPLARYLNGDNVRLYCFGRDGAQLAALRPEVAEQTETMEQAMRLLAPRVQPGDMVLLSPACASLDQFKNFEQRGNEFARLAKELGSHHHHHH

2222222222222222222222222222222222222222222222222222222222222222222222222222222222222222222222222222222222222222111111222222222222222222222222222222222222222122222222222222222222122222222222222222222222222222222222222222222222222222222222222222222222222222222222222221222122222222222222222222222222222212222222222222212112222122122222222222222222222222222222222222222222222222222222222222222222222222222222222222222222222222222222222222222222222

>2QBYB

GAMEVIKNPKVFIDPLSVFKEIPFREDILRDAAIAIRYFVKNEVKFSNLFLGLTGTGKTFVSKYIFNEIEEVKKEDEEYKDVKQAYVNCREVGGTPQAVLSSLAGKLTGFSVPKHGINLGEYIDKIKNGTRNIRAIIYLDEVDTLVKRRGGDIVLYQLLRSDANISVIMISNDINVRDYMEPRVLSSLGPSVIFKPYDAEQLKFILSKYAEYGLIKGTYDDEILSYIAAISAKEHGDARKAVNLLFRAAQLASGGGIIRKEHVDKAIVDYEQERLIEAVKALPFHYKLALRSLIESEDVMSAHKMYTDLCNKFKQKPLSYRRFSDIISELDMFGIVKIRIINRGRAGGVKKYALVEDKEKVLRALNETFEDSISIGDFDDVGEN
[truncated: 431,806 more chars]
