# Supplementary material for: A New Supervised Over-Sampling Algorithm with Application to Protein-Nucleotide Binding Residue Prediction
Source: PLoS One. 2014 Sep 17;9(9):e107676. doi: 10.1371/journal.pone.0107676 (PMC4168127; doi:10.1371/journal.pone.0107676)
Supplement: Supporting Information S2 — Performance comparisons between different over-sampling techniques on the ADP, AMP, GTP, GDP sub-datasets in NUC5. (DOC) [file pone.0107676.s002.doc]

Supporting Information S2: Table S1

**A New Supervised Over-Sampling Algorithm with Application to Protein-Nucleotide Binding Residue Prediction**

Jun Hu^1^, Xue He^1^, Dong-Jun Yu^1, 3,*^, Xi-Bei Yang^1, 4^, Jing-Yu Yang^1^, and Hong-Bin Shen^2,*^

^1^ School of Computer Science and Engineering, Nanjing University of Science and Technology, Xiaolingwei 200, Nanjing, China, 210094

^2^ Institute of Image Processing and Pattern Recognition, Shanghai Jiao Tong University, Dongchuan Road 800, Shanghai, China, 200240

^3^ Changshu Institute, Nanjing University of Science and Technology, Changshu 215513, PR China

^4^ School of Computer Science and Engineering, Jiangsu University of Science and Technology, Huanchenlu 200, Zhenjiang, China, 212003

* Address correspondence to D.J. Yu [njyudj@njust.edu.cn](mailto:njyudj@njust.edu.cn) or H.B. Shen at [hbshen@sjtu.edu.cn](mailto:hbshen@sjtu.edu.cn)

Tel: +86-21-34205320

Fax: +86-21-34204022

*Table S1.* *Performance comparisons between different over-sampling techniques on the ADP, AMP, GTP and GDP sub-datasets in NUC5 over five-fold cross-validation under MaxMCC Evaluation.*

| Ligand Type | Sampling Method | *Sen* (%) | *Spe* (%) | *Acc* (%) | *MCC* | *AUC* |
| --- | --- | --- | --- | --- | --- | --- |
| ADP | SOS | **60.5** | 99.1 | **97.7** | **0.653** | **0.914** |
|  | ADASYN | 59.8 | **99.2** | **97.7** | 0.652 | 0.912 |
|  | SMOTE | 59.7 | 99.1 | **97.7** | 0.645 | 0.910 |
|  | ROS | 58.6 | **99.2** | 97.6 | 0.641 | 0.909 |
| AMP | SOS | 35.4 | **99.1** | **96.7** | **0.453** | **0.850** |
|  | ADASYN | 37.2 | 98.9 | 96.6 | 0.448 | 0.849 |
|  | SMOTE | 37.2 | 98.9 | 96.5 | 0.445 | 0.847 |
|  | ROS | **38.1** | 98.8 | 96.4 | 0.440 | 0.846 |
| GTP | SOS | 47.3 | **99.5** | **97.4** | **0.598** | **0.850** |
|  | ADASYN | 47.2 | **99.5** | **97.4** | 0.597 | 0.848 |
|  | SMOTE | **49.9** | 99.2 | 97.3 | 0.585 | 0.847 |
|  | ROS | 47.1 | 99.3 | 97.3 | 0.575 | 0.846 |
| GDP | SOS | 66.1 | 99.5 | **98.2** | **0.744** | **0.923** |
|  | ADASYN | 64.6 | **99.6** | **98.2** | **0.744** | 0.922 |
|  | SMOTE | **67.1** | 99.3 | 98.1 | 0.737 | 0.920 |
|  | ROS | 66.4 | 99.3 | 97.9 | 0.720 | 0.921 |
